# Synbiotic Therapy Prevents Nosocomial Infection in Critically Ill Adult Patients: A Systematic Review and Network Meta-Analysis of Randomized Controlled Trials Based on a Bayesian Framework

**DOI:** 10.3389/fmed.2021.693188

**Published:** 2021-07-15

**Authors:** Cong Li, Ling Liu, Zhiwei Gao, Junwei Zhang, Hui Chen, Shaolei Ma, Airan Liu, Min Mo, Changde Wu, Dongyu Chen, Songqiao Liu, Jianfeng Xie, Yingzi Huang, Haibo Qiu, Yi Yang

**Affiliations:** ^1^Jiangsu Provincial Key Laboratory of Critical Care Medicine, Department of Critical Care Medicine, School of Medicine, Zhongda Hospital, Southeast University, Nanjing, China; ^2^Department of Critical Care Medicine, School of Medicine, Zhongda Hospital, Southeast University, Nanjing, China; ^3^Emergency Medicine Department of the Affiliated Hospital of Xuzhou Medical University, Xuzhou, China; ^4^Jiangsu Provincial Institute of Health Emergency, Xuzhou Medical University, Xuzhou, China; ^5^Department of Emergency, The Affiliated Huaian NO.1 People's Hospital of Nanjing Medical University, Huai'an, China; ^6^Department of Critical Care Medicine, The First Affiliated Hospital of Soochow University, Soochow University, Suzhou, China; ^7^Department of Intensive Care Medicine, Yancheng City NO.1 People' Hospital, Yancheng, China

**Keywords:** critical illness, synbiotic, nosocomial infection, network meta-analysis, Bayesian

## Abstract

**Background:** The efficacy of synbiotics, probiotics, prebiotics, enteral nutrition or adjuvant peripheral parenteral nutrition (EPN) and total parenteral nutrition (TPN) in preventing nosocomial infection (NI) in critically ill adults has been questioned. We conducted a systematic review and network meta-analysis (NMA) of randomized controlled trials (RCTs) to evaluate and rank the effectiveness of these therapies on NI amongst critically ill adults.

**Methods:** Four electronic databases were systematically searched up to June 30, 2019 for RCTs comparing the administration of probiotics, prebiotics, synbiotics, EPN and TPN in critically ill adults. The primary outcome was NI. The relative efficacy of all outcomes was determined by a Bayesian framework with random effects NMA. We estimated the odds ratio (OR) and mean difference (MD) and ranked the comparative effects of all regimens with the surface under the cumulative ranking probabilities. The study has been registered on PROSPERO (CRD42019147032).

**Results:** Fifty-five RCTs (7,119 patients) were identified. Primary outcome showed that synbiotics had the best effect in preventing NI than EPN (OR 0.37; 95% CrI 0.22–0.61), probiotics followed (OR 0.52; 95% CrI 0.34–0.77), whereas TPN significantly increased NI (OR 2.29; 95% CrI 1.48–3.67). Subgroup analysis showed that TPN significantly increased NI in intensive care unit (ICU) patients (OR 1.57; 95% CrI 1.01–2.56) and severe acute pancreatitis (SAP) patients (OR 3.93; 95% CrI 1.74–9.15). Secondary outcomes showed that synbiotics were more effective in preventing hospital-acquired pneumonia (HAP) (OR 0.34; 95% CrI 0.11–0.85), catheter-related bloodstream infection (OR 0.08; 95% CrI 0.01–0.80), urinary tract infection (OR 0.27; 95% CrI 0.08–0.71) and sepsis (OR 0.34; 95% CrI 0.16–0.70) than EPN. Amongst the treatments, probiotics were most effective for shortening the mechanical ventilation duration (MD −3.93; 95% CrI −7.98 to −0.02), prebiotics were most effective for preventing diarrhea (OR 0.24; 95% CrI 0.05–0.94) and TPN was the least effective in shortening hospital length of stay (MD 4.23; 95% CrI 0.97–7.33).

**Conclusions:** Amongst the five therapies, synbiotics not only prevented NI in critically ill adults but also demonstrated the best treatment results. By contrast, TPN did not prevent NI and ranked last, especially in ICU and SAP patients.

**Take-Home Message:** Nosocomial infection is a leading cause of mortality in critically ill patients in the ICU. However, the efficacy of synbiotics, probiotics, prebiotics, enteral nutrition or adjuvant peripheral parenteral nutrition and total parenteral nutrition in preventing nosocomial infection in critically ill adults has been questioned. The network meta-analysis provides evidence that amongst the five therapies, synbiotics not only prevented NI in critically ill adults but also demonstrated the best treatment results. By contrast, TPN did not prevent NI and ranked last, especially in ICU and SAP patients. The results of this study will provide a new scientific basis and a new idea for the debate on the efficacy of synbiotics and other treatments in the improvement of prognosis in critically ill adult patients.

**Tweet:** Synbiotic prevents nosocomial infection in critically ill adults, while total parenteral nutrition has the adverse curative.

## Introduction

Nosocomial infection (NI) is a common and serious complication in patients with critical illness (1, 2). Patients admitted to the intensive care unit (ICU) are especially susceptible to NI because of their critical illnesses and conditions, such as mechanical ventilation (MV) (3), intracranial hemorrhage (1), severe trauma, severe acute pancreatitis (SAP), complex surgery (2), and extracorporeal membrane oxygenation (ECMO) (4). Intestinal microbiota dysbiosis suggested that gastrointestinal dysfunction plays an important role in the pathogenesis of NI in critically ill patients (5–9). It can result in an increase in susceptibility to NI and significantly affect clinical outcomes (10–15).

Probiotics are live microorganisms that exert beneficial effects by protecting against pathogens, improving intestinal barrier function and inducing host immunomodulation (16). Prebiotics are a substrate that are selectively utilized by host microorganisms maintaining gut homeostasis and improving health outcomes (17–23). Enteral nutrition or adjuvant peripheral parenteral nutrition (EPN) and total parenteral nutrition (TPN) have the functions of protecting the intestinal barrier and providing adequate nutrient substrates, respectively (24). Therefore, all above therapies can partially improve intestinal microbiota dysbiosis, and are widely used in the treatment of NI in critically ill adults (17, 25).

Nonetheless, the advantages of probiotics, prebiotics, synbiotics, EPN and TPN on preventing NI in critically ill patients have been a topic of major debate. Majority of randomized controlled trials (RCTs) performed in critically ill adults have failed to show significant improvement in NI with probiotics, prebiotics and synbiotics therapies (26–34) or have even showed an increased risk of mortality (35). Moreover, RCTs have highlighted the higher risk of bacteremia and fungemia infection resulting from probiotics and synbiotics in immuno-compromised critical patients (33, 35–37).

Many previous conventional meta-analyses have already examined the risks and benefits of probiotics or synbiotics compared with EPN in critically ill adults (38–42). However, all these meta-analyses were restricted to pairwise comparisons, and only the pooled risk ratio (RR) or odds ratio (OR) were calculated. There was heterogeneity between the included trials, and the relative merit of candidate therapies could not be informed through a direct comparison. Network meta-analyses (NMAs) can not only address this limitation but also improve precision by combining direct and indirect estimates (43). Therefore, this systematic review and NMA aimed to evaluate and rank probiotics, prebiotics, synbiotics, EPN and TPN to determine their effects on improving NI of critically ill adult patients. The results of this study will provide a new scientific basis for the debate on the efficacy of synbiotics and other treatments in the improvement of prognosis in critically ill adult patients.

## Methods

### Approval

This literature was written according to the Preferred Reporting Items for Systematic Review and Meta-analyses (PRISMA) Statement Extension Statement (44). This study was registered on the international prospective register of systematic reviews (PROSPERO CRD42019147032).

### Inclusion Criteria

Participants: critically ill patients (≥16 years). If the study population was unclear, we considered a mortality rate higher than 5% in the control group to be consistent with critical illness (42). Interventions: probiotics, prebiotics, synbiotics, EPN and TPN. Primary outcome: NI. Secondary outcomes: hospital-acquired pneumonia (HAP), ventilator-associated pneumonia (VAP), bloodstream infections (BSIs), catheter-related bloodstream infection (CRBSI), urinary tract infection (UTI), sepsis, diarrhea, ICU and hospital mortality, ICU and hospital LOS and duration of MV. Study design: RCT.

### Exclusion Criteria

The trial did not report outcome variables. The trial was a duplicate publication.

### Search Strategy and Study Selection

We conducted a systematic literature search for clinical trials in Pubmed, Embase, Cochrane (CENTRAL) and Web of Science electronic medical databases until June 30, 2019. There was no language restriction. The specific search terms were used for each database, and the details of the search strategy were modified with a combination of relevant terms as proposed by Cochrane for systematic reviews of RCTs (45). The following MeSH terms were used to search for relevant literature: “critically ill” OR “synbiotic” OR “probiotic” OR “prebiotic” OR “enteral nutrition” OR “parenteral nutrition” OR “nosocomial infection” combined with RCTs.

Five reviewers selected studies for inclusion by screening the titles and abstracts of the literature independently. Thereafter, they reviewed the full texts carefully according to the inclusion and exclusion criteria to determine the final inclusion of articles. Any discrepancies between reviewers were resolved by a consensus after a discussion with a sixth reviewer.

### Definition of Interventions

Probiotics are live microorganisms that may confer health benefits on the host when administered in adequate amounts (16, 17). Prebiotics are substrates that are selectively utilized by host microorganisms and confer a health benefit (16, 18). By contrast, synbiotics are composed of probiotics and prebiotics (Supplementary File 3). The US Centers for Disease Control and Prevention (CDC) National Healthcare Safety Network (NHSN) criteria (46) were used to diagnose NI including HAP, VAP, BSIs, CRBSI, UTI, intraabdominal infection, gastroenteritis system infection and surgical site infection (Supplementary Table 2.3). We used definitions of diarrhea as defined by the authors in their original articles. From all trials, we combined hospital mortality where reported. If the mortality time frame was not specified as either ICU or hospital, it was presumed to be the latter.

### Data Extraction

For duplicate studies, we included only the research with the most informative and complete data. Five investigators extracted independently all the available data from each study. These data included characteristics of study, details of patients enrolled, type and dose of intervention and details of primary and secondary outcomes. Disagreements among the three investigators were resolved by a consensus after discussing with a sixth reviewer.

### Assessment of Risk of Bias (ROB) and Quality

We assessed each included studies' ROB in accordance with the Cochrane collaboration risk of bias tool (45). A summary of the ROB was documented as low, unclear or high. Studies were classified as having low ROB if none was rated as high ROB, and three or less were rated as unclear risk. Studies had moderate ROB if one was rated as high ROB or none was rated as high ROB but four or more were rated as unclear risk. All other cases were assumed to pertain to high ROB.

Publication bias was assessed using the comparison-adjusted funnel plots (47, 48).

Additionally, we assessed the certainty of evidence contributing to network estimates with the Grading of Recommendations Assessment, Development and Evaluation (GRADE) system (high, moderate, low and very low) (49).

### Quantitative Data Statistical Analysis

All data were conducted according to the Cochrane Handbook. In pairwise meta-analysis and NMA, dichotomous and continuous variables were analyzed using OR and mean differences (MD), respectively.

The study effect sizes were assessed using a Bayesian framework with a random effects NMA model (50, 51). Dichotomous outcomes used the binomial likelihood, and continuous outcomes used the normal likelihood. Four Markov chains were adopted for initial value setting. The initial update iteration number of the model and the continuous update iteration number were set as 20,000 and 50,000, respectively. The first 20,000 annealing times were used to eliminate the influence of the initial value, and sampling was started from 20,001 times. The initial and continuous iteration numbers of the model increased if the convergence of models was not satisfactory. A potential scale reduction factor approaching 1 indicated that the model convergence was satisfactory (52).

The treatment for each outcome was ranked by using the surface under the cumulative ranking curve (SUCRA) (53).

Heterogeneity variance was considered to measure the extent of a cross-sectional study and within-comparison variability on treatment effects. *I*^2^ < 25% and *I*^2^ > 75% indicate low and high heterogeneity, respectively (54–56). Statistically significant heterogeneity was set at *I*^2^ > 50%, and the sources of heterogeneity were discussed.

A statistical evaluation of inconsistency was assessed by the design-by-treatment test (55, 57) and node splitting (52). Inconsistencies were found between direct and indirect comparison evidence when *P* < 0.05.

The transitivity assumption underlying NMA was evaluated by comparing the distribution of clinical and methodological variables that could act as effect modifiers across treatment comparisons (53, 58).

This study evaluated whether treatment effects for the primary outcome are robust in subgroup analyses by using ICU patients, MV patients, SAP patients, trauma patients, initial time of nutrition therapy, doses, study year, and quality. In view of the fact that European Society for Clinical Nutrition and Metabolism (ESPEN), Society of Critical Care Medicine (SCCM), and American Society for Parenteral and Enteral Nutrition (A.S.P.E.N.) recommend that the initial time of early EN therapy is within 48 h (24, 25), we divided the subgroup of initial nutritional therapy into two groups: within 48 h and beyond 48 h. The average number of obligate anaerobes of normal people was around 10 [log10 colony-forming units (CFUs)/g of feces] (59–61). Therefore, we defined the dose of probiotics that was >2 × 10^10^ CFU per day as high dose and the rest as moderate to low doses.

The sensitivity of our conclusions was evaluated by analyzing only datasets of studies with high quality.

All statistical analyses were performed with Review Manager 5.3, stata (version 14.0) and R software (version 3.6.1). Network plots and comparison-adjusted funnel plots of NMA were drawn by Stata. NMAs of all outcomes were duplicated using the Netmeta 1.1-0 package in R. Bayesian MCMC simulations were performed by means of JAGS software (gemtc 0.8-2 and rjags 4-10 package) in R. Graphs of SUCRA were obtained using the ggplot2 3.2.1 package in R.

## Results

### Search Results and Characteristics of the Studies

**Figure 1:**
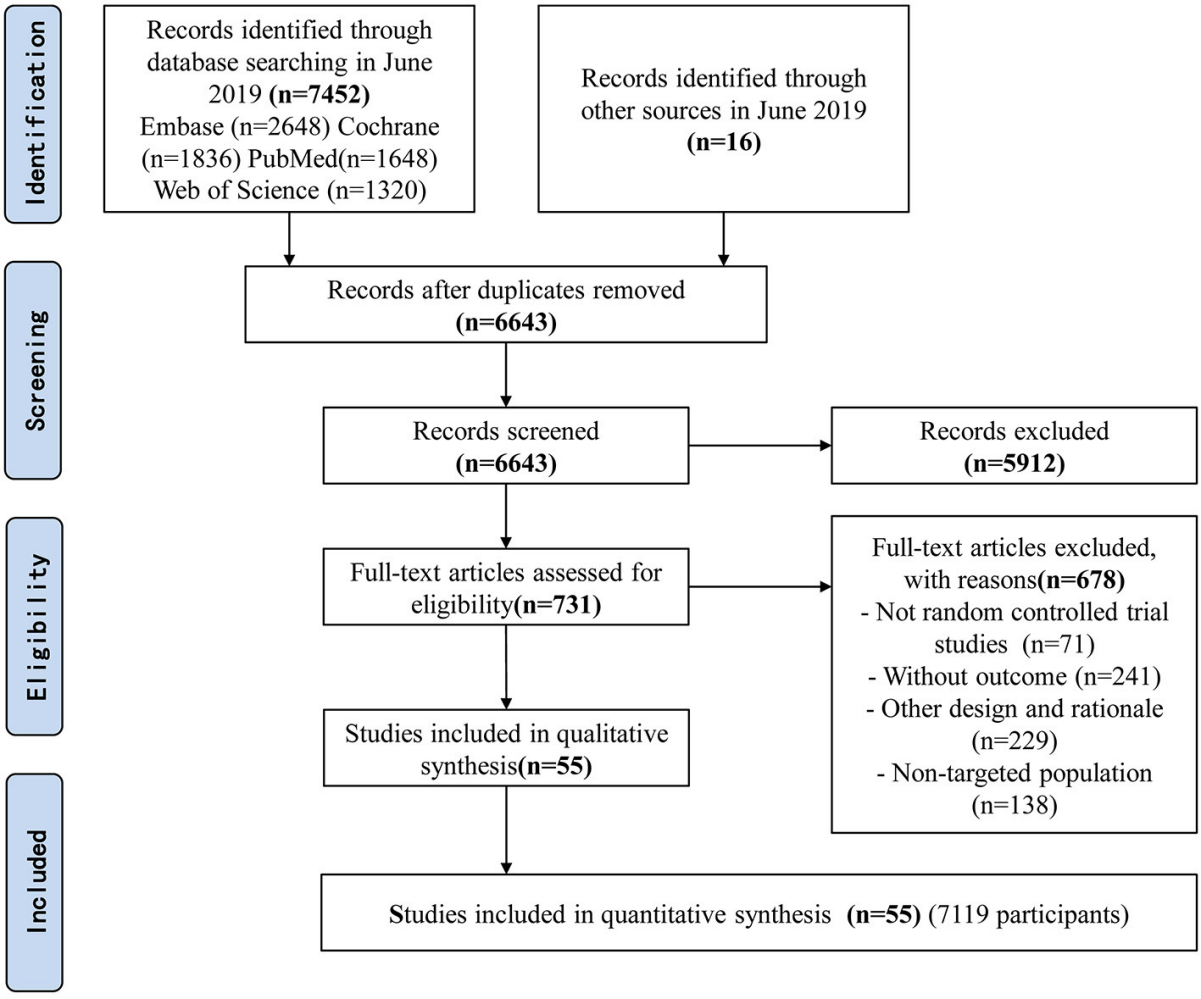
Flow diagram of included studies.

**Figure 2:**
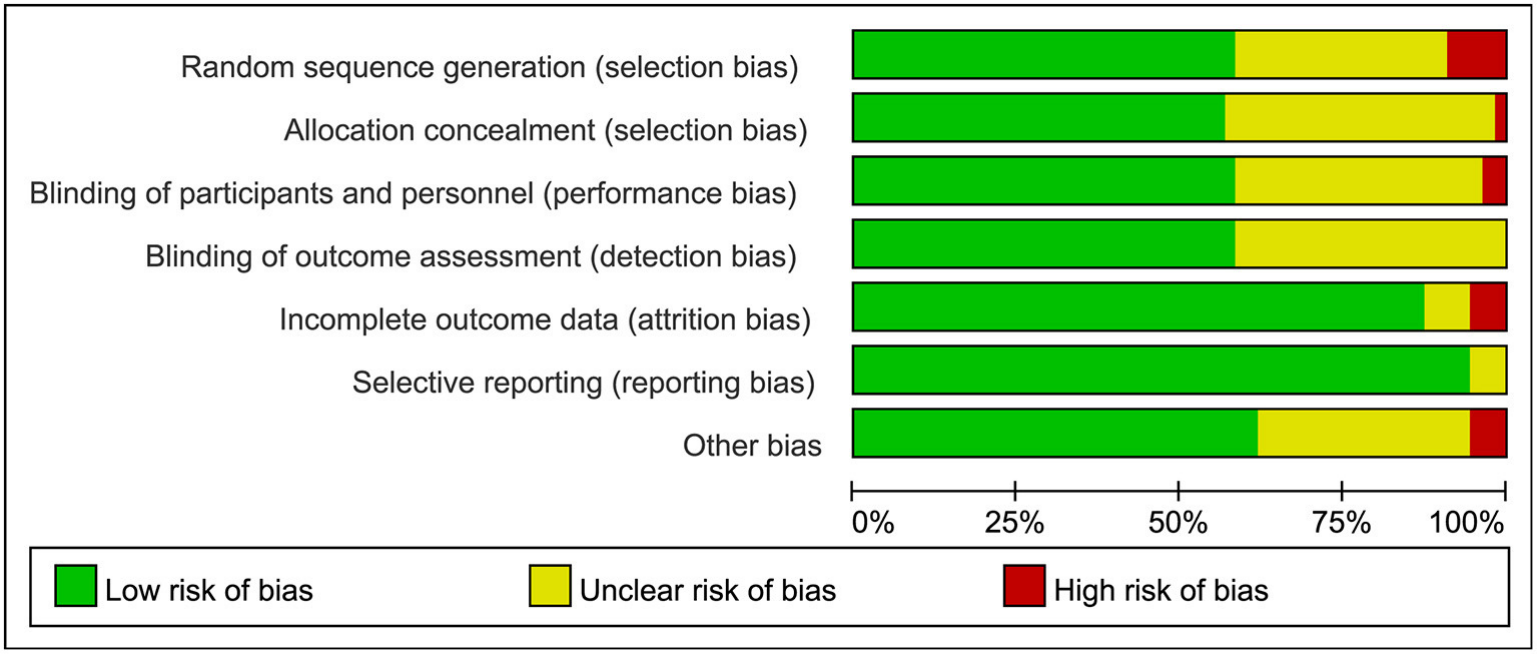
Risk bias assessment graph for included studies.

**Figure 3:**
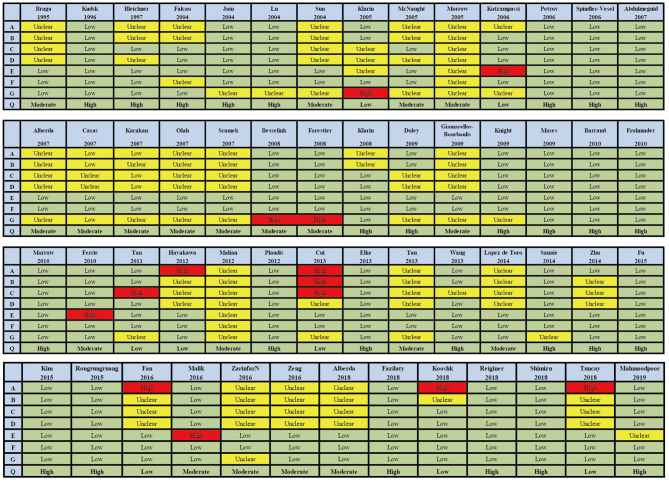
Summary of risk bias assessment for included studies. Studies were classified as having low ROB if none was rated as high ROB, and three or less were rated as unclear risk. Studies had moderate ROB if one was rated as high ROB or none was rated as high ROB but four or more were rated as unclear risk. All other cases were assumed to pertain to high ROB. A = Random sequence generation, B = Allocatin concealment, C = Blinding of participants and personnel, D = Bliding of outcomes assessment, E = Incomplete outcome data, F = Selective reporting, G = Other bias, Q = Quality.

The searches identified 7,468 articles, and 731 potentially eligible articles were retrieved in full text. Overall, 55 RCTs (comprising 7,119 patients) from 24 countries all over the world carried out between 1995 and 2019 were included (Figure 1). A total of 49 articles were published in English, 5 were in Chinese and 1 was in Spanish. Twenty-four (45%) of 55 trials recruited patients from Europe, 23 (42%) from Asia, 6 (15%) from the America and 2 (3%) from Oceania. Sample sizes varied greatly from 17 to 2410, with a mean of 60 participants (SD = 53). The mean age was 53 years old (SD = 12) for both men and women. Of these participants, 4,358 (61%) of 7,119 of the sample population were male. Eleven (20%) of 55 studies randomly assigned participants to three or more groups. Nine (16%) of 55 studies were multi-center studies, 32 (58%) of 55 studies were double-blind studies and 21 (38%) were open-label studies. Mixed diseases in ICU were the most included diseases, followed by MV support, patients with SAP, severe multiple trauma, victims of brain trauma alone and severe burns. Twenty seven (49%) of 55 studies were of high quality. Nineteen (35%) of 55 studies were of moderate quality (Figures 2, 3). A description of the included studies, interventions, and outcomes is presented in Tables 1–3. The details of the design, management description and antibiotics are shown in Supplementary File 2.

**Table 1:** Description of included studies.

**ID**	**Author**	**Year**	**Country**	**Diseases**	**Design**	***N***	**Mean age (SD)**	**Male (%)**	**APACHE II Score**	**SOFA Score**	**Intervention**
1	Braga et al. (62)	1995	Italy	SICU patients undergoing curative surgery for gastric or pancreatic cancer	SC/OP	50	60.3 (7.8)	NR	NR	NR	EN
						27	59.8 (7.1)		NR	NR	TPN
2	Kudsk et al. (63)	1996	America	ICU patients with severe trauma	SC/OP	33	33 (3)	61	NR	NR	EN
						19	35.7 (2.8)	53	NR	NR	TPN
3	Bleichner et al. (64)	1997	France	Critical patients in ICU	MC/DB	64	61.6 (12.3)	70	NR	NR	Probiotics+EN
						64	64.9 (14.1)	72	NR	NR	Placebo+EN
4	Falcão De Arruda and De Aguilar-Nascimento (65)	2004	Brazil	ICU patients with TBI	SC/DB	10	27 (20)	100	NR	NR	Synbiotics+EN
						10	26 (22.22)	90	NR	NR	EN
5	Jain et al. (27)	2004	United of kingdom	Critical patients in ICU	SC/DB	45	72 (11.11)	58	NR	NR	Synbiotics+EN
						45	73 (11.11)	60	NR	NR	Placebo+EN
6	Lu et al. (66)	2004	China	Critical patients with severe burns	SC/DB	20	36.05 (5.16)	85	NR	NR	Synbiotics+EN
						20	37.4 (2.95)	80	NR	NR	Prebiotics+EN
7	Sun et al. (67)	2004	China	Severe acute pancreatitis patients with organ failure	SC/OP	50	46.7 (16.25)	56	NR	NR	EN
						50			NR	NR	TPN
8	Klarin et al. (68)	2005	Sweden	Critical patients in ICU	SC/OP	8	70.9 (34.81)	33	17 (11.9)	NR	Probiotics+EN
						7	57.5 (31.11)	63	19 (16.3)	NR	EN
9	McNaught et al. (28)	2005	United of kingdom	Critical patients in ICU	SC/DB	52	71 (45.93)	63	12 (5.2)	NR	Probiotics+EN
						51	71 (43.7)	51	12 (6.7)	NR	EN
10	Morrow et al. (69)	2005	America	MV patients	SC/DB	19	NR	NR	NR	NR	Probiotics+EN
						21	NR	NR	NR	NR	Placebo+EN
11	Kotzampassi et al. (70)	2006	Greece	SICU patients with severe multiple trauma	MC/DB	35	52.9 (19)	80	19.36 (2.7)	NR	Synbiotics+EN
						30	55.9 (18)	83	19.36 (2.1)	NR	Placebo+EN
12	Petrov et al. (71)	2006	Russia	Severe acute pancreatitis patients with organ failure	SC/OP	35	51 (18.5)	80	12.0 (3.0)	NR	EN
						34	52 (21.5)	71	12.5 (3.7)	NR	TPN
13	Spindler-Vesel et al. (72)	2006	United of kingdom	SICU patients with severe multiple trauma	SC/DB	26	48 (22.59)	78	13.5 (5.6)	NR	Synbiotics+EN
						29	36 (21.48)	NR	14 (5.2)	NR	Prebiotics+EN
						58	35 (20.8)	NR	12 (8.4)	NR	EN
14	Abdulmeguid and Hassan (73)	2007	Greece	MV > 2 days critical patients in ICU	SC/OP	40	NR	NR	NR	NR	EN
						40	NR	NR	NR	NR	TPN
15	Alberda et al. (74)	2007	Canada	ICU patients	SC/DB	10	60.4 (17.9)	50	18.2 (4.2)	NR	Probiotics+EN
						18	64.9 (16.92)	44	15.9 (4.2)	NR	EN
16	Casas et al. (75)	2007	Spain	Severe acute pancreatitis patients with organ failure	SC/OP	11	61.2 (16.6)	77	NR	NR	EN
						11	55.6 (15.6)	77	NR	NR	TPN
17	Karakan et al. (76)	2007	Turkey	Severe acute pancreatitis patients with organ failure	SC/DB	15	47.3 (16.8)	40	9.4 (3.7)	NR	Prebiotics+EN
						15	44.9 (11.2)	53	9.6 (3.8)	NR	EN
18	Olah et al. (77)	2007	Ireland	Severe acute pancreatitis patients with organ failure	SC/DB	33	47.5 (43.7)	82	NR	NR	Synbiotics+EN
						29	46.0 (45.19)	17	NR	NR	Prebiotics+EN
19	Sramek et al. (78)	2007	Czech	Critical patients in ICU	SC/OP	15	55 (19.26)	69	24 (4.44)	NR	Synbiotics+EN
						11				NR	Prebiotics+EN
						144	59.0 (15.5)	57	8.4 (4.5)	1.9 (1.6)	EN
20	Besselink et al. (33)	2008	Netherlands	Patients with predicted severe acute pancreatitis	MC/DB	152	60.4 (16.5)	59	8.6(4.4)	2.1(2.0)	Probiotic+EN
						144	59.0 (15.5)	57	8.4(4.5)	1.9(1.6)	EN
21	Forestier et al. (79)	2008	France	Critical patients in ICU	SC/DB	102	60 (54.07)	64	NR	NR	Probiotics+EN
						106	57 (45.93)	76	NR	NR	Placebo+EN
22	Klarin et al. (80)	2008	Sweden	Critical patients in ICU	MC/DB	22	65.5 (44.44)	59	22 (16.3)	NR	Synbiotics+EN
						22	64 (50.37)	59	11 (20)	NR	Prebiotics+EN
23	Doley et al. (81)	2009	India	Severe acute pancreatitis patients with organ failure	SC/OP	25	38.4 (13.8)	NR	≥8	NR	EN
						25	41.1 (11.3)	NR	≥8	NR	TPN
24	Giamarellos-Bourboulis et al. (82)	2009	Greece	SICU patients with severe multiple injuries	MC/DB	36	52.9	NR	19.36	NR	Synbiotics+EN
						36	55.9	NR	19.36	NR	EN
25	Knight et al. (26)	2009	United of kingdom	MV patients	SC/DB	130	49.5 (19.6)	62	17 (8.1)	NR	Synbiotics+EN
						129	50.0 (18.5)	62	17 (7.4)	NR	Placebo+EN
26	Moses et al. (83)	2009	India	ICU patients with acute organophosphate poisoning needing invasive mechanical ventilatory support	SC/OP	29	29.41 (11.8)	76	NR	NR	EN
						30	30.83 (12.4)	73	NR	NR	TPN
27	Barraud et al. (84)	2010	France	MV patients	SC/DB	87	59.1 (15.9)	39	NR	9 (4.6)	Probiotics+EN
						80	61.8 (15.5)	44	NR	9.7 (4.8)	Placebo+EN
28	Frohmader et al. (85)	2010	Australia	Critical patients in ICU	SC/DB	20	60.8 (15.6)	65	22.2 (8.9)	NR	Probiotics+EN
						25	65.5 (9.8)	28	23.8 (10.2)	NR	Placebo+EN
29	Morrow et al. (29)	2010	America	MV patients	SC/DB	73	67.5 (31.11)	33	22.7 (7.5)	NR	Probiotics+EN
						73	61.5 (26.67)	46	23.7 (8.0)	NR	Prebiotics+EN
30	Ferrie and Daley (86)	2011	Australia	Critically ill patients with diarrhea	SC/SB	18	56.2 (19.4)	44	27.7 (6.3)	NR	Synbiotics+EN
						18	61.7 (17.5)	44	29.6 (6.1)	NR	Prebiotics+EN
31	Tan et al. (87)	2011	China	ICU patients with severe TBI	SC/DB	26	40.5 (13.0)	73	14.8 (3.6)	6.5 (1.4)	Probiotics+EN
						26	40.8 (12.8)	81	14.3 (3.6)	6.3 (1.4)	EN
32	Hayakawa et al. (88)	2012	Japan	MV patients	SC/OP	31	74 (14)	45	NR	NR	Synbiotics+EN
						16	75 (7)	75	NR	NR	EN
33	Malian et al. (89)	2012	America	Critical patients in SICU	SC/DB	36	60	59	16.7	NR	Probiotics+EN
						33				NR	Placebo+EN
34	Plaudis et al. (90)	2012	Latvia	Severe acute pancreatitis patients with organ failure	SC/OP	30	NR	37	8.8 (3.6)	NR	Synbiotics+EN
						28	NR		8.6 (4.9)	NR	Prebiotics+EN
						32	NR		6.8 (4.3)	NR	EN
35	Cui et al. (91)	2013	China	Severe acute pancreatitis patients with organ failure	SC/OP	23	44.9 (19.3)	70	≥8	NR	Probiotics+EN
						25			≥8	NR	EN
						22			≥8	NR	PN
36	Elke et al. (92)	2013	Germany	ICU patients with severe sepsis or septic shock	MC/OP	328	66 (12.7)	62	20 (5.8)	7 (3.6)	EN
						25	61 (10.4)	68	16 (4.4)	6 (2.2)	TPN
37	Tan et al. (93)	2013	China	SICU patients with severe TBI	SC/DB	26	40.5 (13.0)	73	14.8 (3.6)	6.5 (1.4)	Probiotics+EN
						26	40.8 (12.8)	81	14.3 (3.6)	6.3 (1.4)	EN
38	Wang et al. (94)	2013	China	ICU patients with severe acute pancreatitis	SC/DB	62	42.6 (13.8)	52	12.88 (3.19)	NR	Probiotics+EN
						61	43.7 (13.7)	52	13.27 (2.86)	NR	EN
						60	41.7 (11.4)	57	14.63 (3.67)	NR	TPN
39	Lopez de Toro et al. (95)	2014	Spain	ICU patients with multi-organ failure	SC/DB	46	68.5 (19.26)	68.5	20 (8.1)	9 (3.0)	Synbiotics+EN
						43	70 (14.07)		22 (5.9)	9 (3.0)	EN
40	Sanaie et al. (96)	2014	Iran	Critical patients in ICU	SC/DB	20	33.60 (5.50)	65	22.8 (4.73)	12.25 (2.57)	Probiotics+EN
						20	35.60 (5.03)	70	22.45 (4.57)	12.55 (2.6)	EN
41	Zhu et al. (34)	2014	China	Severe acute pancreatitis patients with organ failure	SC/DB	20	43.5 (17.5)	55	≥8	NR	Probiotics+EN
						19	42.0 (16.5)	53	≥8	NR	Placebo+EN
42	Fu et al. (97)	2015	China	Patients with severe acute pancreatitis	SC/OP	36	48.9 (12.2)	NR	11.4 (4.9)	NR	Probiotics+EN
						36	51.3 (13.6)	NR	12.3 (5.1)	NR	TPN
43	Kim et al. (98)	2015	South Korea	ICU patients after living donor liver transplantation	SC/OP	17	52 (7)	88	NR	NR	EN
						19	52 (5.5)	95	NR	NR	TPN
44	Rongrungruang et al. (99)	2015	Thailand	MV patients	SC/OP	75	68.95 (18.45)	60	19.88 (6.89)	NR	Probiotics+EN
						75	73.09 (13.16)	57	19.41 (7.04)	NR	EN
45	Fan et al. (100)	2016	China	NICU patients with severe TBI	SC/OP	80	41.22 (16.77)	51	NR	NR	EN
						40	41.56 (15.10)	53	NR	NR	TPN
46	Malik et al. (101)	2016	Malaysia	Critical patients in ICU	SC/DB	24	60 (14.4)	67	22.12 (6.0)	NR	Probiotics+EN
						25	55 (17.7)	68	23 (8.9)	NR	Placebo+EN
47	Zarinfar et al. (102)	2016	Iran	MV patients	SC/DB	30	NR	NR	NR	NR	Probiotics+EN
						30	NR	NR	NR	NR	Placebo+EN
48	Zeng et al. (32)	2016	China	MV patients	MC/OP	118	50.2 (18.2)	62	14.7 (3.9)	NR	Probiotics+EN
						117	54.6 (17.9)	56	16.6 (4.3)	NR	EN
49	Alberda et al. (103)	2018	Canada	Critical patients in ICU	SC/OP	16	59.9 (15.6)	75	25.5 (5.39)	NR	Probiotics+EN
						16	57.5 (15.0)	63	25.9 (9.70)	NR	EN
50	Fazilaty et al. (104)	2018	Iran	ICU patients with multiple trauma	SC/DB	20	NR	90	62 (8)	5 (1.3)	Prebiotics+EN
						20	NR	90	62 (8.5)	9 (3.0)	Placebo + EN
51	Kooshki et al. (105)	2018	Iran	MV patients	SC/DB	30	54.37 (19.18)	40	22.7 (7.5)	NR	Prebiotics+ EN
						30	59.53 (17.37)	63	23.7 (8)	NR	EN
52	Reiginer et al. (106)	2018	French	MV patients	MC/OP	1,202	66 (14)	67	NR	11 (3)	EN
						1,208	66 (14)	67	NR	11 (3)	TPN
53	Shimizu et al. (107)	2018	Japan	Patients MV for ≥72 h and diagnosed sepsis	SC/SB	35	74 (13.33)	71	19 (7.4)	NR	Synbiotics+EN
						37	74 (12.59)	59	20 (8.9)	NR	EN
54	Tuncay et al. (108)	2018	Turkey	Critical patients in NICU	SC/DB	23	73.9 (15.3)	39	NR	NR	Prebiotics+EN
						23	71.8 (20.0)	61	NR	NR	EN
55	Mahmoodpoor et al. (31)	2019	Iran	MV patients	MC/DB	48	59.1 (12.9)	54	24.1 (6.2)	NR	Probiotics+EN
						54	57.5 (14.5)	54	22.8 (4.7)	NR	Placebo+EN

*DB, double-blind; EN, enteral nutrition; GCS, Glasgow coma scale; MC, multi-center; MV, mechanical ventilation; NICU, neurological intensive care unit; NR, not reported, OP, open study; RCT, randomized controlled trials; SB, single-blind; SC, single-center; SD, mean difference; SICU, Surgical intensive care unit; TBI, traumatic brain injuries; TPN, total parenteral nutrition*.

**Table 2:** Description of included studies.

	**Author**	**Diseases**	***N***	**Intervention**	**Details of intervention**	**Dose or volume of intervention**
1	Braga et al. (62)	SICU patients undergoing curative surgery for gastric or pancreatic cancer	50	EN	Impart+standard formula	25 kcal/kg.day^−1^
			27	TPN	Isonitrogenous isocaloric	
2	Kudsk et al. (63)	ICU patients with severe trauma	33	EN	Impart, Immun-Aid	Mean 1,400 kcal/day
			19	TPN	NR	NR
3	Bleichner et al. (64)	Critical patients in ICU	64	Probiotics+EN	**Probiotics:** S. boulardii **EN:** Intact protein standard diet without fiber or lactose	500 mg QID
			64	Placebo+EN	**Placebo:** Powder was indistinguishable from the S. boulardii powder **EN:** Intact protein standard diet without fiber or lactose	500 mg QID
4	Falcão De Arruda and De Aguilar-Nascimento (65)	ICU patients with TBI	10	Synbiotics+EN	Fermented milk (Lactobacillus johnsonii)	Fermented milk 240 ml QD
			10	EN	Standard formula	NR
5	Jain et al. (27)	Critical patients in ICU	45	Synbiotics+EN	**Probiotics (Trevis**^**TM**^**)**: L. acidophilus La5, L. bulgaricus, Bifidobacterium lactis Bb-12, Streptococcus thermophilus **Prebiotics**: oligofructose EN: NR	Probiotic 4 × 10^9^ cfu TID Prebiotic 7.5 g BID
			45	Placebo+EN	**Placebo:** Sucrose powder **EN:** NR	Powdered sucrose capsules TID
6	Lu et al. (66)	Critical patients with severe burns	20	Synbiotics+EN	**Probiotics**: Pediococcus pentosaceus, Leuconostoc mesenteroides, Lactobacillus paracasei subsp paracasei, Lactobacillus plantarum **Prebiotics**: Betaglucan, Inulin, Pectin, Resistant starch EN: Nutrison Fibre	Probiotic 4 × 10^10^ cfu QD Prebiotic 10 g QD
			20	Prebiotics+EN	**Prebiotics**: Betaglucan, Inulin, Pectin, Resistant starch **EN:** Nutrison Fibre	10 g QD
7	Sun et al. (67)	Critical patients with severe burns	50	EN	Flicare	NR
			50	TPN	Harris-Benedict formula	125–146 kJ/kg
8	Klarin et al. (68)	Critical patients in ICU	8	Probiotics+EN	**Probiotics**: Lactobacillus plantarum 299v	Probiotics: 5 × 10^10^ cfu Q6h 3 days
			7	EN	NR	NR
9	McNaught et al. (28)	Critical patients in ICU	52	Probiotics+EN	**Probiotics**: Proviva (L. plantarum 299 v)	Probiotics:2.5 × 10^9^ cfu QD
			51	EN	EN	EN
10	Morrow et al. (69)	MV patients	19	Probiotics+EN	Lactobacillus GG	1 × 10^9^ cfu BID
			21	Placebo+EN	Inactive plant starch inulin	BID
11	Kotzampassi et al. (70)	SICU patients with severe multiple trauma	35	Synbiotics+EN	Synbiotic 2000 Forte **Probiotics**: Pediococcus pentoseceus 5–33:3, Leuconostoc mesenteroides 32–77:1, L. paracasei ssp 19, L. plantarum 2,362 **Prebiotics**: inulin, oat bran, pectin, resistant starch	Probiotic 4 × 10^9^ cfu QD Prebiotic 10 g QD
			30	Placebo+EN	**Placebo:** Maltodextrin	QD
12	Petrov et al. (71)	SICU patients with severe multiple trauma	35	EN	Peptamen	Daily 30 kcal/kg and 1.5 g/kg of protein (ideal body weight)
			34	TPN	10% dextrose solution, 10% amino acid solution, and 10% fat emulsion	
13	Spindler-Vesel et al. (72)	SICU patients with severe multiple trauma	26	Synbiotics+EN	Synbiotic 2000 **Probiotics**: Lactobacillus: Pediococcus pentosaceus 5–33:3, Lactococcus raffinolactis 32–77:1, Lactobacillus paracasei subsp paracasei 19, Lactobacillus plantarum 2362 **Prebiotics**: Glucan, inulin, pectin, resistant starch	Probiotic 4 × 10^10^ cfu QD Prebiotic 10 g QD
			29	Prebiotics+EN	Nova Source: fermentable fibers	2.2 g per 100 mL
			58	EN	**Nutricomp peptide Alitraq**: Glutamine, arginine, α-linolenic acid	1.55 g glutamine, 446 mg arginine, 154 mg α-linolenic acid per 100 mL
14	Spindler-Vesel et al. (72)	MV > 2 days critical patients in ICU	40	EN	NR	NR
			40	TPN	Identical amounts of fat, carbohydrate, and protein.	NR
15	Alberda et al. (74)	Critial patients in ICU	10	Probiotics+EN	**VSL#3**: Lactobacillus, Bifidobacterium, Streptococcus salivarius subsp. Thermophilus	Probiotics: 4.5 × 10^11^ cfu BID EN: 25–30 kcal/kg, 1.2–1.5 g/kg protein
			18	EN	**Jevity Plus**	25–30 kcal/kg, 1.2–1.5 g/kg protein
16	Casas et al. (75)	Severe acute pancreatitis patients with organ failure	11	EN	PEPTISORB	1.5–2 g proteins/kg/day and 30–35 kcal/kg/day
			11	TPN	NR	1.5–2 g proteins/kg/day and 30–35 kcal/kg/day
17	Karakan et al. (76)	Severe acute pancreatitis patients with organ failure	15	Prebiotics+EN	**Multifiber:** Soluble fibers and insoluble fibers	24 g per day
			15	EN	**EN:** No prebiotics, no placebo	2,000 kcal/d
18	Olah et al. (77)	Severe acute pancreatitis patients with organ failure	33	Synbiotics+EN	Synbiotic 2000 Forte **Probiotics**: Pediococcus pentoseceus 5–33:3, Leuconostoc mesenteroides 32–77:1, L. paracasei ssp 19, L. plantarum 2,362 **Prebiotics**: Inulin, oat bran, pectin, resistant starch	Probiotic 4 × 10^10^ cfu QD Prebiotic 10 g QD
			29	Prebiotics+EN	Plant fibers (Betaglucan, inulin, pectin, resistant starch)	10 g QD
19	Sramek et al. (78)	Critical patients in ICU	15	Synbiotics+EN	Synbiotic 2000 Forte **Probiotics**: Pediococcus pentoseceus 5–33:3, Leuconostoc mesenteroides 32–77:1, L. paracasei ssp 19, L. plantarum 2,362 **Prebiotics**: Inulin, oat bran, pectin, resistant starch, inulin, oat bran, pectin, resistant starch	Probiotic 4 × 10^10^ cfu QD Prebiotic 10 g QD
			11	Prebiotics+EN	Tea	NR
20	Besselink et al. (33)	Patients with predicted severe acute pancreatitis	152	Probiotic+EN	**Probiotic** (Ecologic 641): six different strains of freeze-dried, viable bacteria: Lactobacillus acidophilus, Lactobacillus casei, Lactobacillus salivarius, Lactococcus lactis, Bifidobacterium bifidum, Bifidobacterium lactis) EN: Nutrison Multi Fibre	Probiotic 10^10^ cfu totally daily
			144	EN	Nutrison Multi Fibre	NR
21	Forestier et al. (79)	Critical patients in ICU	102	Probiotics+EN	**Probiotics**: Lactobacillus casei rhamnosus	10^9^ cfu BID
			106	Placebo+EN	**Placebo:** Growth medium without bacteria	NR
22	Klarin et al. (80)	Critical patients in ICU	22	Synbiotics+EN	**Probiotics**: 299 Lactobacillus plantarum 8 × 10^8^ cfu /ml **Prebiotics**: Oatmeal	Probiotics: given as 6 × 100 ml doses every 12 h and after 50 ml given BID
			22	Prebiotics+EN	**Prebiotics**: Oatmeal	Same oatmeal gruel mixed with lactic acid
23	Doley et al. (81)	Severe acute pancreatitis patients with organ failure	25	EN	NR	2,500–2,700 kcal/day, 120–130 g/day of protein
			25	TPN	NR	2,500–2,700 kcal/day, 120–130 g/day of protein
24	Giamarellos-Bourboulis et al. (82)	SICU patients with severe multiple injuries	36	Synbiotics+EN	Synbiotic 2000 Forte **Probiotics**: Pediococcus pentoseceus 5–33:3, Leuconostoc mesenteroides 32–77:1, L. paracasei ssp 19, L. plantarum 2,362 **Prebiotics**: Inulin, oat bran, pectin, resistant starch **EN**: Intestamin	Probiotic: 4 × 10^10^ cfu QD Prebiotic:10 g QD
			36	EN	Intestamin	NR
25	Knight et al. (26)	MV patients	130	Synbiotics+EN	Synbiotic 2000 Forte **Probiotics**: Pediococcus pentoseceus 5–33:3, Leuconostoc mesenteroides 32–77:1, L. paracasei ssp 19, L. plantarum 2,362 **Prebiotics**: Inulin, oat bran, pectin, resistant starch **EN**: Nutrison Energy	Probiotic 4 × 10^10^ cfu BID Prebiotic 10 g BID
			129	Placebo+EN	**Placebo:** Crystalline cellulose **EN:** Nutrison Energy	10 g BID
26	Moses et al. (83)	ICU patients with acute organophosphate poisoning needing invasive mechanical ventilatory support	29	EN	Hypocaloric EN	Maximum of 1,000 cal/d and protein 28.32 g
			30	TPN	Glucose and electrolyte	Maximum of 1,000 cal/d and protein 28.32 g
27	Barraud et al. (84)	MV patients	87	Probiotics+EN	**Probiotics:** Ergyphilus Lactobacillus rhamnosus GG, Lactobacillus casei, Lactobacillus acidophilus, Bifidobacterium bifidum **EN:** Fresubin	Probiotics: 2 × 10^10^ cfu QD EN: 30–35 kcal/kg
			80	Placebo+EN	**Placebo:** Excipient **EN:** Fresubin	Placebo: NR EN: 30–35 kcal/kg
28	Frohmader et al. (85)	Critical patients in ICU	20	Probiotics+EN	**Probiotics (VSL#3):** Lactobacillus, Bifidobacterium, Streptococcus salivarius subsp. Thermophilus **EN:** Isosource or Renal or Diabetic Resource (Novartis, Melbourne, Australia)	Probiotics: 4.5 × 10^11^ cfu BID EN: 25 to 35 cal/kg per day and 0.8 to 1.5 g protein per kilogram per day
			25	Placebo+EN	**Placebo:** Free of fiber and prebiotic additives **EN:** Isosource or Renal or Diabetic Resource (Novartis, Melbourne, Australia)	Placebo: BID EN: 25 to 35 cal/kg per day and 0.8 to 1.5 g protein per
29	Morrow et al. (29)	MV patients	73	Probiotics+EN	**Probiotics**: Lactobacillus rhamnosus GG EN: NR	Probiotics: 2 × 10^9^ cfu BID
			73	Prebiotics+EN	**Prebiotics**: Inulin **EN**: NR	BID
30	Ferrie and Daley (86)	Critically ill patients with diarrhea	18	Synbiotics+EN	**Probiotics**: Lactobacillus rhamnosus GG **Prebiotics**: inulin powder **EN:** standard feeding formula, which is a 1-calorie per mL oat fiber–containing formula	Probiotic: 10^10^ cfu QD Prebiotic:280 mg QD
			18	Prebiotics+EN	**Prebiotics**: Inulin powder **EN:** standard feeding formula, which is a 1-calorie per mL oat fiber–containing formula	Prebiotic:280 mg QD
31	Tan et al. (87)	ICU patients with severe TBI	26	Probiotics+EN	**Probiotics:** Golden Bifid: 0.5 × 10^8^ cfu Bifidobacterium longum, 0.5 × 10^7^ cfu Lactobacillus bulgaricus,0.5 × 10^7^ cfu Streptococcus thermophilus **EN:** (3.8 g protein, 13.8 g carbohydrate, 3.4 g fat/100 ml, osmolarity 250 mOsm/l, no fibers)	Probiotics:10^9^ cfu per day EN: 30 kcal/kg body weight/day
			26	EN	**EN:** (3.8 g protein, 13.8 g carbohydrate, 3.4 g fat/100 ml, osmolarity 250 mOsm/l, no fibers)	30 kcal/kg body weight/day
32	Hayakawa et al. (88)	MV Patients	31	Synbiotics+EN	**Probiotics** (Yakult): 1 × 10^8^ cfu /g Bifidobacterium breve strain Yakult, 1 × 10^8^ cfu /g Lactobacillus casei strain Shirota **Prebiotics**: galactooligosaccharides **EN:** Medief (100 kcal, protein 4.5 g, fat 2.8 g, carbohydrate 14.2 g, dietary fiber 1.2 g in 100 ml) (Ajinomoto)	Probiotics: 1 g TID Prebiotics: 5 g TID EN: According to the patient's requirements
			16	EN	Medief (100 kcal, protein 4.5 g, fat 2.8 g, carbohydrate 14.2 g, dietary fiber 1.2 g in 100 ml) (Ajinomoto)	According to the patient's requirements
33	Malian et al. (89)	Critical patients in SICU	36	Probiotics+EN	**Probiotics:** Lactobacillus GG **EN:** NR	NR
			33	Placebo+EN	**Placebo:** NR **EN:** NR	NR
34	Plaudis et al. (90)	Severe acute pancreatitis patients with organ failure	30	Synbiotics+EN	Synbiotic 2000 Forte **Probiotics**: Pediococcus pentoseceus 5–33:3, Leuconostoc mesenteroides 32–77:1, L. paracasei ssp 19, L. plantarum 2,362 **Prebiotics**: inulin, oat bran, pectin, resistant starch EN: Nutrison, standard whole protein feeding formula	Probiotic 4 × 10^9^ cfu BID Prebiotic 10 g BID EN 2,500 kcal/day
			28	Prebiotics+EN	**Prebiotics**: Inulin, oat bran, pectin, resistant starch EN: Nutrison, standard whole protein feeding formula	Prebiotic 10 g BID EN 2,500 kcal/day
			32	EN	Nutrison, standard whole protein feeding formula	2,500 kcal/day
35	Cui et al. (91)	Severe acute pancreatitis patients with organ failure	23	Probiotics+EN	**Protiotics:** Bifidobacterium **EN:** Peptisorb, Nutrison Fibre	**Protiotics:**10.416 × 10^9^ cfu Q12h, **EN: NR**
			25	EN	**EN:** Peptisorb, Nutrison Fibre	**EN:** NR
			22	PN	Glucose, electrolyte, fat emulsion, amino acid	**EN:** NR
36	Elke et al. (92)	ICU patients with severe sepsis or septic shock	328	EN	NR	NR
			25	TPN	NR	NR
37	Tan et al. (93)	SICU patients with severe TBI	26	Probiotics+EN	**Protiotics:** Golden Bifid: 0.5 × 10^8^ cfu Bifidobacterium longum, 0.5 × 10^7^ cfu Lactobacillus bulgaricus,0.5 × 10^7^ cfu Streptococcus thermophilus **EN:** Standard formula	**Protiotics:**10^9^ cfu per day **EN:** NR
			26	EN	Standard formula	NR
38	Wang et al. (94)	ICU patients with severe acute pancreatitis	62	Probiotics+EN	**Protiotics:** Bacillus subtilis 1.8 × 10^9^ cfu /g, Enterococcus faecium 2.0 × 10^8^ cfu /g **EN:** PEPTISORB	**Protiotics:** 0.5 g TID **EN:** 2 g proteins/kg/d and 35 kcal/kg/d
			61	EN	**EN:** PEPTISORB	**EN:**2 g proteins/kg/d and 35 kcal/kg/d
			60	TPN	TPN	2 g proteins/kg/d and 35 kcal/kg/d, A ratio of 120:1 of non-protein calories-to-nitrogen
39	Lopez de Toro et al. (95)	ICU patients with multi-organ failure	46	Synbiotics+EN	**Probiotics (**Drink Simbiotic): streptococcus Thermophilus, lactobacillus bulgaricus, Lactobacilluscasei, lactobacillus acidophilus, bifidobacterium, Escherichia coli, coliformes **Prebiotics**: NR	Max 4.8 × 10^9^ cfu /ml
			43	EN	NR	NR
40	Sanaie et al. (96)	Critical patients in ICU	20	Probiotics+EN	**Probiotics** (VSL#3): Lactobacillus acidophilus, Bifidobacterium longus, Bifidobacterium bifidum &Bifidobacterium infantalis **EN:** Fresubin original fibre	Probiotics:9.0 × 10^9^ cfu BID EN: Energy requirements 25–30 kcal/kg and protein 1.2–1.5 g/kg.
			20	EN	**EN:** Fresubin original fibre	Energy requirements 25–30 kcal/kg and protein 1.2–1.5 g/kg.
41	Zhu et al. (34)	Severe acute pancreatitis patients with organ failure	20	Probiotics+EN	**Probiotics:** Clostridium Butyricum (miyarisan) **EN:** NR	0.7 × 10^6^ cfu BID
			19	Placebo+EN	**Placebo:** Starch **EN:** NR	The same capsule type and amount
42	Fu et al. (97)	Patients with severe acute pancreatitis	36	Probiotics+EN	**Probiotics:** live combined bacillus subtilis and enterococcusfaecium **EN:** Peptisorb, Nutrison Fibre	NR
			36	TPN	NR	1.0–1.5 g proteins/kg/day and 25–30 kcal/kg/day
43	Kim et al. (98)	ICU patients after living donor liver transplantation	17	EN	Mediwell RTH 500	NR
			19	TPN	NR	NR
44	Rongrungruang et al. (99)	MV patients	75	Probiotics+EN	**Probiotics:** Lactobacillus casei (Yakult) (Shirota strain) **EN:** NR	8 × 10^9^ cfu for oral care after standard oral care QD. 8 × 10^9^ cfu enteral feeding QD
			75	EN	NR	NR
45	Fan et al. (100)	NICU patients with severe TBI	80	EN	Nutrison Fibre	105–126 KJ/d
			40	TPN	2:1 for carbohydrates to lipids and 100:1 for calorie nitrogen ratio	105–126 KJ/d
46	Malik et al. (101)	Critical patients in ICU	24	Probiotics+EN	**Probiotics:** Lactobacillus acidophilus, Lactobacillus casei, Lactobacillus lactis, Bifidobacterium bifidum, Bifidobacterium longum, Bifidobacterium infantis **EN:** Osmolite 1 cal (standard formula), Glucerna (glucose intolerance formula), Peptamen (semielemental formula), and Novasource Renal (electrolyte and fluid restriction).	**Probiotics:**3 × 10^9^ cfu BID **EN:**25 kcal kg^−1^ d^−1^
			25	Placebo+EN	**Placebo:** Similar appearance and taste, **EN:** Osmolite 1 cal (standard formula), Glucerna (glucose intolerance formula), Peptamen (semielemental formula), and Novasource Renal (electrolyte and fluid restriction).	**Placebo:** 3 g BID **EN:**25 kcal kg^−1^ d^−1^
47	Zarinfar et al. (102)	MV patients	30	Probiotics+EN	**Probiotics:** Lactobacillus GG	TID
			30	Placebo+EN	**Placebo:** NR	TID
48	Zeng et al. (32)	MV patients	118	Probiotics+EN	**Probiotics:** Medilac-S: Bacillus subtilis 4.5 × 10^9^ cfu /0.25 g and Enterococcus faecalis 0.5 × 10^9^ cfu /0.25 g **EN:** NR	**Probiotics:**0.5 g TID EN: NR
			117	EN	NR	NR
49	Alberda et al. (103)	Critical patients in ICU	16	Probiotics+EN	**Probiotics:** Lactobacillus casei (Danactive)	1 × 10^10^ cfu BID
			16	EN	No prebiotics, no placebo	NR
50	Fazilaty et al. (104)	ICU patients with multiple trauma	20	Prebiotics+EN	**Prebiotics:** b-glucan **EN:** high-protein enteral diet (20% protein, 30% lipid, and 50% carbohydrate)	3 g QD 25–30 kcal/kg
			20	Placebo + EN	**Placebo:** Maltodextrin **EN:** high-protein enteral diet (20% protein, 30% lipid, and 50% carbohydrate)	3 g QD 25–30 kcal/kg
51	Kooshki et al. (105)	MV patients	30	Prebiotics+ EN	**Prebiotics:** Fenugreek seed powder **EN:** NR	3 g BID
			30	EN	NR	NR
52	Reiginer et al. (106)	MV patients	1,202	EN	Isosmotic, isocaloric, normal-protein, polymeric preparations	Daily calorie target in kcal/kg of actual bodyweight was 20–25 during the first 7 days then 25–30 from day 8 to extubation.
			1,208	TPN	Three groups of macronutrients	Daily calorie target in kcal/kg of actual bodyweight was 20–25 during the first 7 days then 25–30 from day 8 to extubation
53	Shimizu et al. (107)	Patients MV for ≥72 h and diagnosed sepsis	35	Synbiotics+EN	**Probiotics** (Yakult BL Seichoyaku): 1 × 10^8^ cfu /g B. breve strain /g and 1 × 10^8^ cfu /g L. casei strain Shirota **Prebiotics**: galactooligosaccharides (Oligomate S-HP) **EN:** Standard polymeric diet Glucerna^®^-Ex 1 kcal/mL; 51:17:32 ratio of carbohydrate, protein, and fat; 370 mOsm/L; fiber 1.4 g/100 mL	Probiotics: 3 g QD Prebiotics: 10 g QD EN: 25–30 kcal/kg ideal body weight per day as the calorie goal
			37	EN	Standard polymeric diet Glucerna^®^-Ex 1 kcal/mL; 51:17:32 ratio of carbohydrate, protein, and fat; 370 mOsm/L; fiber 1.4 g/100 mL	25–30 kcal/kg ideal body weight per day as the calorie goal
54	Tuncay et al. (108)	Critical patients in NICU	23	Prebiotics+EN	**Prebiotics:** Fructo-oligosaccharides (Jevity, 1 kcal/1 ml) **EN:** Standard formula (Osmolite, 1 kcal/1 ml)	Prebiotics:5.3 g QD 1 g/kg/ day EN:30–40 ml/kg/day
			23	EN	Standard formula (Osmolite, 1 kcal/1 ml)	1 g/kg/ day and 30–40 ml/kg/day
55	Mahmoodpoor et al. (31)	MV patients	48	Probiotics+EN	**Probiotics:** Lactocare: Lactobacillus species (casei, acidophilus, rhamnosus, bulgaricus), Bifidobacterium species (breve, longum), Streptococcus thermophilus. **EN:** Standard formula (1 kcal/mL; Ensure)	Probiotics:10^10^ cf u BID EN:25 kcal/kg
			54	Placebo+EN	**Placebo:** Sterile maize starch powder **EN:** Standard formula (1 kcal/mL;Ensure)	Placebo: BID EN:25 kcal/kg

*CFU, colony forming units; EN, enteral nutrition; GCS, Glasgow coma scale; MV, mechanical ventilation; NG, nasogastric; NJ, nasojejunal; NR, not reported; OG, orogastric; PN, parenteral nutrition; TBI, traumatic brain injuries; TPN, total parenteral nutrition*.

**Table 3:** Reported clinical outcomes of included studies.

	**Intervention**	**Nosocomial Infection (n/N)**							**Diarrhea**	**Mortality (n/N)**		**Mean LOS (SD)**		
		**Total**	**HAP**	**VAP**	**BI**	**CRBIS**	**UTI**	**Sepsis**		**Hospital**	**ICU**	**Hospital**	**ICU**	**MV**
1	EN	6/50	NR	NR	NR	NR	NR	NR	NR	NR	NR	14.3 (5.0)	NR	NR
	TPN	4/27	NR	NR	NR	NR	NR	NR	NR	NR	NR	19.3 (7.3)	NR	NR
2	EN	16/33	2/33	NR	5/33	NR	8/33	NR	NR	2/33	NR	25.7 (8.8)	7.7 (2.8)	3.9 (2.3)
	TPN	13/19	4/19	NR	8/19	NR	4/19	NR	NR	0/19	NR	34.9 (6.0)	15.7 (4.9)	9.0 (4.2)
3	Probiotics+EN	NR	NR	NR	NR	NR	NR	NR	18/64	NR	NR	NR	NR	NR
	Placebo+EN	NR	NR	NR	NR	NR	NR	NR	24/64	NR	NR	NR	NR	NR
4	Synbiotics+EN	5/10	NR	NR	NR	NR	NR	0/10	NR	NR	NR	NR	11.11 (10)	7 (10.37)
	EN	10/10	NR	NR	NR	NR	NR	3/10	NR	NR	NR	NR	22 (37.04)	14 (37.04)
5	Synbiotics+EN	33/45	NR	NR	NR	NR	NR	26/45	NR	22/45	NR	14 (14.81)	7 (9.63)	NR
	Placebo+EN	26/45	NR	NR	NR	NR	NR	33/45	NR	20/45	NR	15 (12.59)	5 (8.148)	NR
6	Synbiotics+EN	8/20	NR	NR	3/20	4/20	NR	NR	NR	2/20	NR	NR	NR	NR
	Prebiotics+EN	11/20	NR	NR	5/20	7/20	NR	NR	NR	1/20	NR	NR	NR	NR
7	EN	NR	NR	NR	NR	NR	NR	NR	18/50	7/50	NR	24.5	NR	NR
	TPN	NR	NR	NR	NR	NR	NR	NR	3/50	10/50	NR	30.2	NR	NR
8	Probiotics+EN	6/8	5/8	NR	0/8	3/8	2/8	NR	NR	2/8	1/8	NR	12 (24.44)	NR
	EN	5/7	2/7	NR	3/7	3/7	1/7	NR	NR	2/7	2/7	NR	11 (33.33)	NR
9	Probiotics+EN	21/52	NR	NR	NR	NR	NR	NR	NR	18/52	NR	NR	5 (5.158)	NR
	EN	22/51	NR	NR	NR	NR	NR	NR	NR	18/51	NR	NR	4 (3.704)	NR
10	Probiotics+EN	2/19	NR	5/19	NR	NR	NR	NR	NR	NR	NR	NR	NR	NR
	Placebo+EN	7/21	NR	10/21	NR	NR	NR	NR	NR	NR	NR	NR	NR	NR
11	Synbiotics+EN	17/35	19/35	NR	NR	13/35	6/35	6/35	5/35	5/35	5/35	NR	27.7 (15.2)	16.7 (9.5)
	Placebo+EN	23/30	24/30	NR	NR	20/30	13/30	12/30	10/30	9/30	9/30	NR	41.3 (20.5)	29.7 (16.15)
12	EN	7/35	2/35	NR	NR	0/35	2/35	NR	6/35	2/35	NR	NR	NR	NR
	TPN	25/34	2/34	NR	NR	5/34	4/34	NR	1/34	12/34	NR	NR	NR	NR
13	Synbiotics+EN	5/26	4/26	NR	0/26	0/26	0/26	NR	NR	2/26	2/26	NR	12 (9.481)	11 (8.37)
	Prebiotics+EN	17/29	12/29	NR	2/29	0/29	0/29	NR	NR	2/29	2/29	NR	16 (8.148)	12 (5.185)
	EN	29/58	22/58	NR	2/58	2/58	1/58	NR	NR	3/58	3/58	NR	12.9 (10.6)	9.1 (7.7)
14	EN	14/40	NR	NR	NR	NR	NR	NR	NR	7/40	NR	10.82	7.6	6.25
	TPN	20/40	NR	NR	NR	NR	NR	NR	NR	11/40	NR	12.95	10.32	8.65
15	Probiotics+EN	0/10	NR	NR	NR	NR	NR	0/10	1/10	NR	1/10	NR	NR	NR
	EN	0/18	NR	NR	NR	NR	NR	0/18	3/18	NR	2/18	NR	NR	NR
16	EN	1/11	NR	NR	0/11	0/11	1/11	2/11	NR	0/11	NR	30.2	NR	NR
	TPN	5/11	NR	NR	3/11	2/11	0/11	2/11	NR	2/11	NR	30.7	NR	NR
17	Prebiotics+EN	3/15	NR	NR	NR	NR	NR	1/15	NR	2/15	NR	10 (4.44)	6 (2.22)	NR
	EN	3/15	NR	NR	NR	NR	NR	2/15	NR	4/15	NR	15 (14.07)	6 (1.481)	NR
18	Synbiotics+EN	9/33	2/33	NR	NR	NR	3/33	3/33	NR	2/33	NR	14.9	NR	NR
	Prebiotics+EN	15/33	4/29	NR	NR	NR	3/29	5/29	NR	6/29	NR	19.7	NR	NR
19	Synbiotics+EN	9/15	NR	NR	NR	NR	NR	NR	NR	0/15	NR	NR	14 (16.3)	NR
	Prebiotics+EN	4/10	NR	NR	NR	NR	NR	NR	NR	1/11	NR	NR	10 (9.63)	NR
20	Probiotic+EN	46/152	24/152	NR	32/152	NR	1/152	1/152	25/152	24/152	NR	28.9 (41.5)	6.6 (17.1)	NR
	EN	41/144	16/144	NR	22/144	NR	2/144	2/144	28/144	9/144	NR	23.5 (25.9)	3 (9.3)	NR
21	Probiotics+EN	24/102	NR	24/102	NR	NR	NR	NR	NR	NR	NR	NR	NR	NR
	Placebo+EN	24/106	NR	24/106	NR	NR	NR	NR	NR	NR	NR	NR	NR	NR
22	Synbiotics+EN	11/22	7/22	NR	2/22	1/22	2/22	NR	NR	3/22	2/22	NR	5.5 (14.44)	4.4 (12.07)
	Prebiotics+EN	16/22	9/22	NR	3/22	3/22	1/22	NR	NR	2/22	2/22	NR	8.8 (48.81)	7.3 (14.52)
23	EN	16/25	NR	NR	5/25	NR	NR	4/25	NR	5/25	NR	42 (23.3)	10 (11)	NR
	TPN	15/25	NR	NR	8/25	NR	NR	3/25	NR	4/25	NR	36 (14.3)	15 (15)	NR
24	Synbiotics+EN	NR	NR	15/36	5/36	NR	6/36	5/36	NR	5/36	NR	NR	NR	NR
	EN	NR	NR	16/36	13/36	NR	11/36	13/36	NR	10/36	NR	NR	NR	NR
25	Synbiotics+EN	12/130	NR	12/130	NR	NR	NR	NR	7/130	35/130	28/130	19 (20.74)	6 (5.926)	5 (5.185)
	Placebo+EN	17/129	NR	17/129	NR	NR	NR	NR	9/129	42/129	34/129	18 (18.52)	7 (8.148)	5 (5.926)
26	EN	17/29	NR	12/29	NR	3/29	2/29	NR	0/29	3/29	NR	15 (7.8)	10.5 (5.2)	12 (6.3)
	TPN	19/30	NR	10/30	NR	4/30	5/30	NR	1/30	3/30	NR	12 (5.6)	8 (5.6)	10 (5.9)
27	Probiotics+EN	30/87	NR	23/87	NR	3/87	4/87	NR	48/87	27/87	21/87	26.6 (22.3)	18.7 (12.4)	NR
	Placebo+EN	30/80	NR	15/80	NR	11/80	4/80	NR	42/80	24/80	21/80	28.9 (26.4)	20.2 (20.8)	NR
28	Probiotics+EN	NR	NR	NR	NR	NR	NR	NR	NR	5/20	NR	NR	7.3 (5.7)	6 (5.2)
	Placebo+EN	NR	NR	NR	NR	NR	NR	NR	NR	3/25	NR	NR	8.1 (4)	6.71 (5.25)
29	Probiotics+EN	13/73	NR	13/73	NR	NR	NR	NR	46/73	12/73	NR	21.7 (17.4)	14.8 (11.8)	9.6 (7.2)
	Prebiotics+EN	28/73	NR	28/73	NR	NR	NR	NR	57/73	15/73	NR	21.4 (14.9)	14.6 (11.6)	9.5 (6.3)
30	Synbiotics+EN	NR	NR	NR	NR	NR	NR	NR	NR	2/18	NR	54.5 (31.26)	32.04 (24.46)	NR
	Prebiotics+EN	NR	NR	NR	NR	NR	NR	NR	NR	2/18	NR	59.04 (33.92)	29.75 (18.81)	NR
31	Probiotics+EN	9/26	2/10	7/16	0/26	NR	0/26	0/26	NR	3/26	NR	NR	6.8 (3.8)	NR
	EN	15/26	1/7	13/19	1/26	NR	2/26	0/26	NR	5/26	NR	NR	10.7 (7.3)	NR
32	Synbiotics+EN	5/31	5/31	NR	NR	NR	NR	NR	NR	NR	NR	NR	NR	NR
	EN	3/16	3/16	NR	NR	NR	NR	NR	NR	NR	NR	NR	NR	NR
33	Probiotics+EN	NR	NR	NR	NR	NR	NR	NR	NR	NR	NR	NR	18	9
	Placebo+EN	NR	NR	NR	NR	NR	NR	NR	NR	NR	NR	NR	21	17
34	Synbiotics+EN	2/30	NR	NR	2/30	NR	NR	2/30	NR	0/30	NR	NR	NR	NR
	Prebiotics+EN	2/28	NR	NR	2/28	NR	NR	2/28	NR	1/28	NR	NR	NR	NR
	EN	12/32	NR	NR	7/32	NR	NR	1/32	NR	5/32	NR	NR	NR	NR
35	Probiotics	2/23	NR	NR	NR	NR	NR	NR	NR	1/23	NR	10.4 (3.9)	NR	NR
	EN	5/25	NR	NR	NR	NR	NR	NR	NR	1/25	NR	13.4 (5.2)	NR	NR
	TPN	12/22	NR	NR	NR	NR	NR	NR	NR	3/22	NR	25.8 (6.4)	NR	NR
36	EN	193/328	NR	NR	NR	NR	NR	NR	NR	70/328	NR	NR	29 (27.2)	NR
	TPN	17/25	NR	NR	NR	NR	NR	NR	NR	4/25	NR	NR	12 (25.9)	NR
37	Probiotics+EN	NR	NR	NR	NR	NR	NR	NR	NR	3/26	NR	NR	6.8 (3.8)	NR
	EN	NR	NR	NR	NR	NR	NR	NR	NR	5/26	NR	NR	10.7 (7.3)	NR
38	Probiotics+EN	8/62	NR	NR	NR	NR	NR	8/62	NR	5/62	NR	NR	NR	NR
	EN	13/61	NR	NR	NR	NR	NR	13/61	NR	6/61	NR	NR	NR	NR
	TPN	24/60	NR	NR	NR	NR	NR	24/60	NR	7/60	NR	NR	NR	NR
39	Synbiotics+EN	9/46	NR	NR	NR	NR	NR	NR	NR	19/46	15/46	18.5 (19.26)	9 (4)	10 (3.75)
	EN	13/43	NR	NR	NR	NR	NR	NR	NR	18/43	14/43	24.5 (20.74)	8 (3.5)	8.5 (3.625)
40	Probiotics+EN	2/20	NR	NR	NR	NR	NR	2/20	NR	NR	NR	NR	NR	NR
	EN	5/20	NR	NR	NR	NR	NR	5/20	NR	NR	NR	NR	NR	NR
41	Probiotics+EN	NR	5/20	NR	11/20	NR	2/20	NR	NR	NR	NR	NR	1.21	NR
	Placebo+EN	NR	6/19	NR	13/19	NR	1/19	NR	NR	NR	NR	NR	1.01	NR
42	Probiotics+EN	2/36	NR	NR	NR	NR	NR	NR	NR	1/36	NR	15.4 (8.5)	NR	NR
	TPN	15/36	NR	NR	NR	NR	NR	NR	NR	2/36	NR	23.2 (9.7)	NR	NR
43	EN	1/17	NR	2/17	NR	0/17	NR	NR	NR	0/17	NR	23 (25.3)	6 (4)	NR
	TPN	5/19	NR	5/19	NR	2/19	NR	NR	NR	0/19	NR	24 (16)	6 (1.3)	NR
44	Probiotics+EN	18/75	NR	18/75	NR	NR	NR	NR	19/75	18/75	NR	20 (26)	30.5 (23.5)	NR
	EN	22/75	NR	22/75	NR	NR	NR	NR	14/75	17/75	NR	19 (42)	19 (6.25)	NR
45	EN	NR	13/80	NR	NR	NR	NR	13/80	32/80	16/80	NR	NR	29.52 (7.01)	10.48 (5.80)
	TPN	NR	19/40	NR	NR	NR	NR	19/40	6/40	17/40	NR	NR	36.33 (8.61)	18.63 (5.39)
46	Probiotics+EN	NR	NR	NR	NR	NR	NR	NR	NR	NR	NR	NR	10.9 (3.9)	8.4 (3.5)
	Placebo+EN	NR	NR	NR	NR	NR	NR	NR	NR	NR	NR	NR	15.8 (7.8)	14 (8)
47	Probiotics+EN	7/30	NR	7/30	NR	NR	NR	NR	1/30	5/30	NR	24.1 (5.6)	14.2 (4.7)	NR
	Placebo+EN	15/30	NR	15/30	NR	NR	NR	NR	6/30	16/30	NR	27.4 (6.6)	17.6 (6.5)	NR
48	Probiotics+EN	NR	NR	48/118	NR	NR	NR	NR	NR	26/118	15/118	13.5 (12.4)	18 (13.33)	12 (9.63)
	EN	NR	NR	62/117	NR	NR	NR	NR	NR	25/117	9/117	10.6 (10.2)	22 (33.33)	17 (11.11)
49	Probiotics+EN	1/16	NR	NR	NR	NR	NR	NR	11/16	2/16	1/16	79.56 (116.8)	11.38 (7.4)	NR
	EN	2/16	NR	NR	NR	NR	NR	NR	10/16	2/16	2/16	39.38 (54.74)	15.31 (12.96)	NR
50	Prebiotics+EN	5/20	NR	4/20	NR	0/20	0/20	0/20	NR	1/20	NR	NR	27.55 (7.8)	15 (9.3)
	Placebo + EN	11/20	NR	4/20	NR	3/20	4/20	2/20	NR	4/20	NR	NR	31.2 (15.8)	28 (21.3)
51	Prebiotics+ EN	7/30	NR	7/30	NR	NR	NR	NR	1/30	2/30	NR	24.1 (5.6)	14.2 (4.8)	16.06 (4.81)
	EN	15/30	NR	15/30	NR	NR	NR	NR	10/30	6/30	NR	27.4 (6.6)	17.6 (6.7)	20.26 (6.05)
52	EN	173/1,202	NR	113/1,202	38/1,202	29/1,202	18/1,202	NR	432/1,202	498/1,202	429/1,202	NR	9 (8.1)	NR
	TPN	194/1,208	NR	118/1,208	55/1,208	27/1,208	16/1,208	NR	393/1,208	479/1,208	405/1,208	NR	10 (8.9)	NR
53	Synbiotics+EN	10/35	NR	5/35	5/35	NR	NR	NR	NR	3/35	NR	NR	28 (20.74)	NR
	EN	25/37	NR	18/37	5/37	NR	NR	NR	NR	4/37	NR	NR	23 (22.22)	NR
54	Prebiotics+EN	NR	NR	NR	NR	NR	NR	NR	2/23	NR	NR	NR	NR	NR
	EN	NR	NR	NR	NR	NR	NR	NR	12/23	NR	NR	NR	NR	NR
55	Probiotics+EN	NR	NR	NR	NR	NR	NR	NR	7/48	NR	5/48	14.2 (8.6)	11.6 (8)	8.75 (4.79)
	Placebo+EN	NR	NR	NR	NR	NR	NR	NR	15/54	NR	6/54	21.1 (5.7)	18.6 (6.3)	12.08 (7.125)

*BI, Bloodstream infection; CRBIS, Catheter-related bloodstream infection; EN, enteral nutrition; HAP, hospital acquired pneumonia; LOS, length of stay; MV, Mechanical ventilation; NR, not reported; SD, standard deviation; TPN, Total parenteral nutrition; UTI, Urinary tract infection; VAP, Ventilator-associated Pneumonia*.

### Primary Outcome

**Figure 4:**
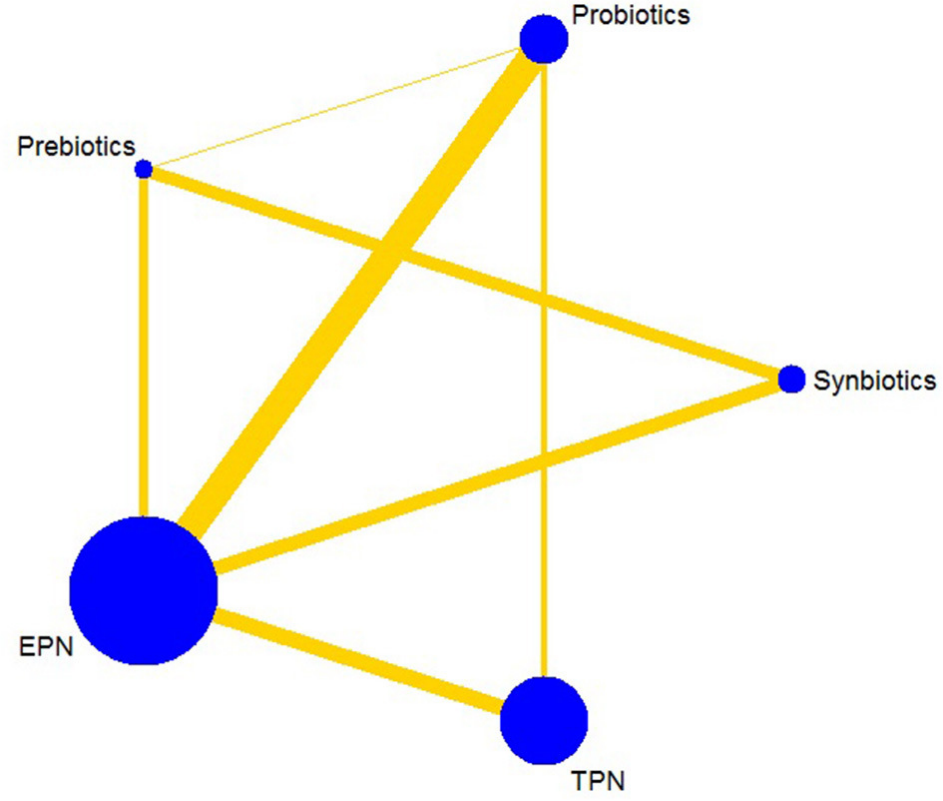
Network plot of all intervention comparisons for nosocomial infection. The size of the nodes corresponds to the total number of participants that study the treatments. The (directly) comparable treatments are linked with a line. The thickness of the line corresponds to the standard error of trials that study this comparison. The colors of the line correspond to the quality of trials that study this comparison. Low risk of bias [green], moderate risk of bias [yellow]. EPN, Enteral nutrition or adjuvant peripheral parenteral nutrition; TPN, Total parenteral nutrition.

**Figure 5:**
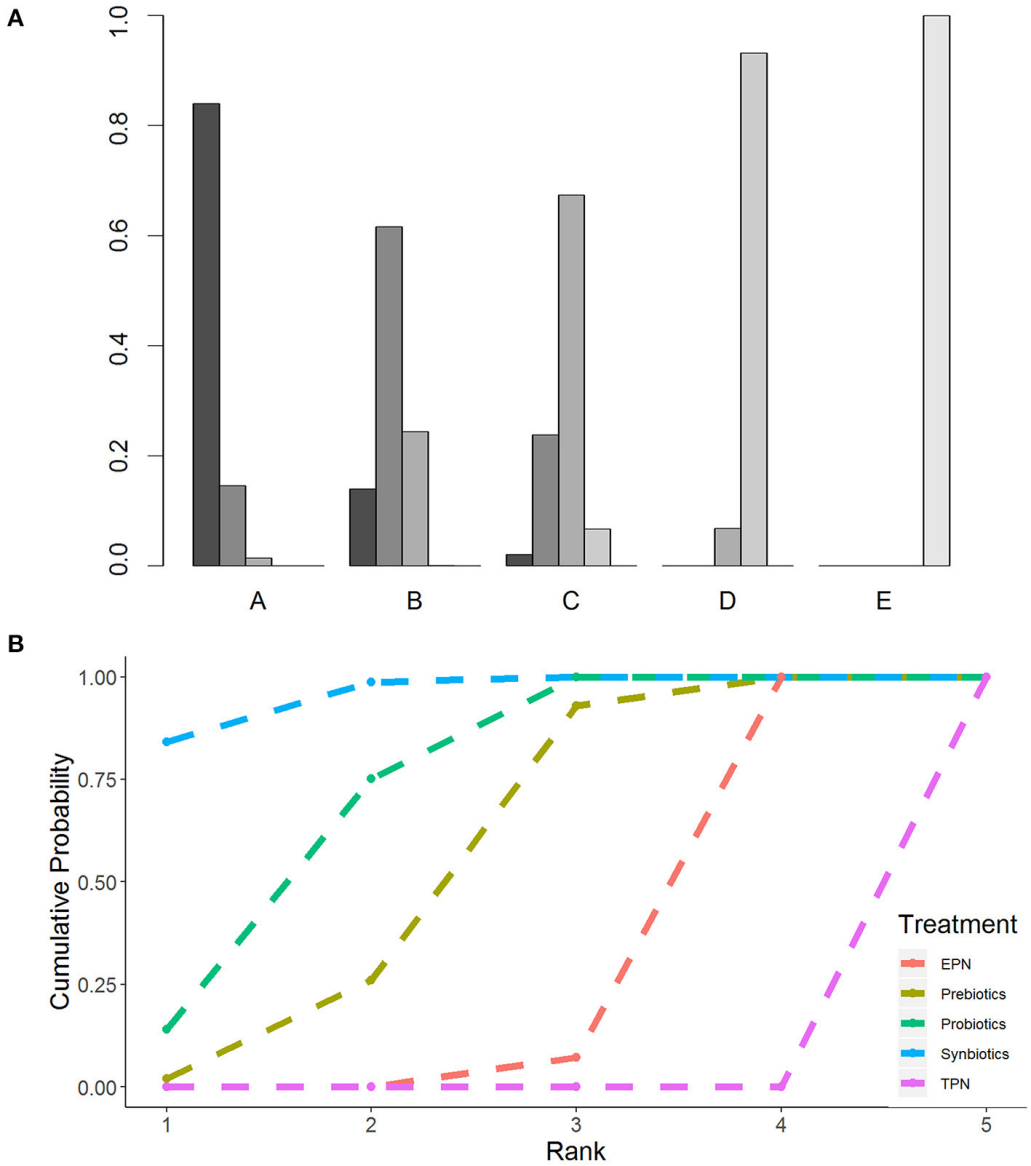
Rankogram and SUCRA ranking curve for nosocomial infection. **(A)** Rankogram for nosocomial infection. A = Synbiotics. B = Probiotics. C = Probiotics. D = EPN. E = TPN. **(B)** SUCRA ranking for nosocomial infection. The number on the X-axis represents the rank. As the number goes up, the rating goes down. EPN, Enteral nutrition or adjuvant peripheral parenteral nutrition; TPN, Total parenteral nutrition.

The primary analysis was based on the 43 studies comprising 6,215 patients. Figure 4 displays the network of eligible comparisons for NI. All treatment had at least one EPN-controlled trial. Only synbiotic therapy was not directly compared with probiotic and TPN therapy in the network. Table 4 shows the results of NMA for NI. In terms of preventing the efficacy of NI, synbiotic (OR 0.37; 95% CrI 0.22–0.61) and probiotic (OR 0.52; 95% CrI 0.34–0.77) therapy were associated with lower morbidity than EPN. By contrast, TPN was worse than EPN (OR 2.29; 95% CrI 1.48–3.67). Figure 5 shows the SUCRA ranking curve of NI. Synbiotic treatment was the best choice in preventing NI, whereas TPN was the worst.

**Table 4:** Results from pairwise meta-analyses and network meta-analyses on nosocomial infection.

**Synbiotics**	–	1.90 (0.94, 3.90)	**2.50 (1.50, 4.60)**	–
0.71 (0.38, 1.34)	**Probiotics**	2.90 (0.79, 11.11)	**1.60 (1.10, 2.40)**	**8.30 (2.90, 25.21)**
0.57 (0.32, 1.01)	0.84 (0.44, 1.60)	**Prebiotics**	2.10 (1.00, 4.70)	–
**0.37 (0.22, 0.61)**	**0.52 (0.34, 0.77)**	0.65 (0.35, 1.15)	**EPN**	**2.00 (1.30, 3.30)**
**0.16 (0.08, 0.31)**	**0.23 (0.12, 0.39)**	**0.28 (0.13, 0.58)**	**0.44 (0.27, 0.68)**	**TPN**

*Data are the ORs (95% CrI) in the column-defining treatment compared with the row-defining treatment. With treatment as the boundary, the lower left part of the table is the result of network meta-analyses, and the upper right part of the table is the result of pairwise meta-analyses. For network meta-analyses, ORs lower than 1 favor the column-defining treatment (e.g., column 1 vs. row 4 in the lower left part of the table (synbiotics vs. EPN) is the result of network meta-analyses (OR 0.37 95% CrI 0.22–0.61), so is favor the synbiotics). For pairwise meta-analyses, ORs higher than 1 favor the row-defining treatment. (e.g., column 4 vs. row 1 in the upper right part of the table (EPN vs. synbiotics) is the result of pairwise meta-analyses (OR 2.50 95% CrI 1.50–4.60), so is favor the synbiotics). To obtain ORs for comparisons in the opposite direction, reciprocals should be taken. Significant results are in bold and underscored. OR, odds ratio; CrI, credible interval; EPN, Enteral nutrition or adjuvant peripheral parenteral nutrition; TPN, Total parenteral nutrition*.

### Secondary Outcomes

**Figure 6:**
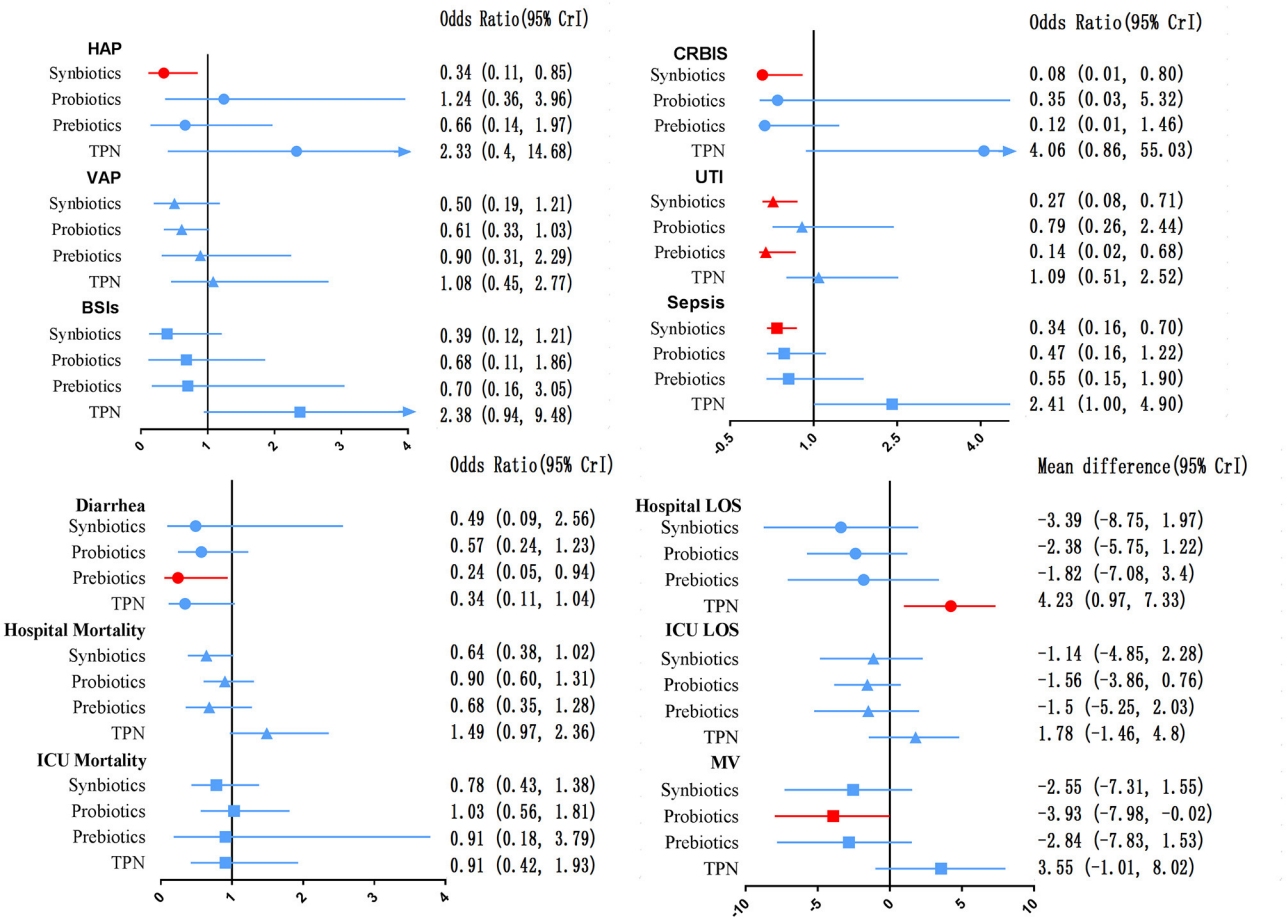
Forest plot of the effect estimate for each active intervention vs. EPN on secondary outcomes. Estimates are presented as odds ratios (OR) and 95% CrI. OR < 1 favor the treatment. BSIs, Bloodstream infections; CrI, credible interval; CRIBS, Catheter-related bloodstream infection; EPN, Enteral nutrition or adjuvant peripheral parenteral nutrition; HAP, Hospital acquired pneumonia; TPN, Total parenteral nutrition; LOS, length of stay; MV, Duration of Mechanical ventilation; UTI, urinary tract infection; VAP, Ventilator-associated pneumonia.

The network of eligible comparisons for secondary outcomes is presented in Supplementary Files 5, 6. Figure 6 presents the results of NMA for secondary outcomes. In terms of improving the efficacy of HAP, CRBIS, UTI and sepsis, synbiotic therapy was more effective than EPN, and the results of the network were OR 0.34; 95% CrI 0.11–0.85, OR 0.08; 95% CrI 0.01–0.80, OR 0.27; 95% CrI 0.08–0.71 and OR 0.34; 95% CrI 0.16–0.70, respectively. In terms of shortening the duration of MV, probiotics were more effective than EPN (MD −3.93; 95% CrI −7.98 to −0.02). In terms of preventing the efficacy of diarrhea, prebiotics were more effective than EPN (OR 0.24; 95% CrI 0.05–0.94). By contrast, TPN was worse than EPN on shortening of hospital LOS (MD 4.23; 95% CrI 0.97–7.33). No regimen significantly improved other secondary outcomes. Details of network plot graph, results of the consistent model and forest plot of the effect estimate are shown in Supplementary File 6. The SUCRA ranking curve showed that synbiotic therapy was the best choice for HAP, VAP, BSIs, CRBIS, sepsis, hospital mortality, ICU mortality and hospital LOS, while TPN was the worst choice for all secondary outcomes except diarrhea (Supplementary File 12).

### Direct Meta-Analysis

The forest plot of the pairwise and network effect estimate on NI is shown in Figure 5. The detailed results of all outcomes in pairwise meta-analysis are shown in Supplementary Files 5, 6.

### Network Heterogeneity, Inconsistency, and Transitivity

The analysis of heterogeneity (Supplementary File 7) revealed moderate-to-high global heterogeneity in NI (*I*^2^ = 62.02%), VAP (*I*^2^ = 54.33%), CRBIS (*I*^2^ = 79.14%), diarrhea (*I*^2^ = 91.11%), hospital LOS (*I*^2^ = 98.56%), ICU LOS (*I*^2^ = 79.47%) and duration of MV (*I*^2^= 86.10%).

In the analysis of inconsistency (Supplementary File 8), there was no global inconsistency in all outcomes except diarrhea (*p* = 0.0018). Inconsistencies were found between direct and indirect comparisons of probiotic therapy and EPN for NI (*p* = 0.04143), synbiotic and prebiotic therapy for CRBIS (*p* = 0.03569), synbiotic therapy and EPN for CRBIS (*p* = 0.04404), prebiotic therapy and EPN for CRBIS (*p* = 0.02783), synbiotic and prebiotic therapy for UTI (*p* = 0.04033), synbiotic therapy and EPN for UTI (*p* = 0.03591), prebiotic therapy and EPN for UTI (*p* = 0.04071), probiotic and prebiotic therapy for diarrhea (*p* = 0.01030), probiotic therapy and EPN for diarrhea (*p* = 0.01008), prebiotic therapy and EPN for diarrhea (*p* = 0.01060), and probiotic therapy and TPN for hospital LOS (*p* = 0.04520).

In the assessment of transitivity (Supplementary File 9), most of the comparisons had similar mean age, but there were a few comparisons with relatively low or high age. Meta-regressions of mean age did not show that they affected the network estimates, although results from such analyses might suffer from ecological bias.

### Subgroup and Sensitivity Analyses for Primary Outcome

Subgroup analysis of the diseases (Table 5) revealed a significant effect on the therapeutic effect of synbiotic therapy except MV patients and patients with initial time of nutrition therapy beyond 48 h, while TPN was shown to increase the morbidity of NI in different disease subgroups except MV patients (OR 1.31 95% CrI 0.51–3.87). In addition, we found that the heterogeneity and consistency in different disease subgroups were not statistically significant. Amongst RCTs over the last 10 years, high-quality studies and doses were used in our NMA. They were found to have no material impact on the relative treatment effects (Supplementary File 13).

**Table 5:** Subgroup analyses for nosocomial infection in different populations.

	**Overall patients**		**ICU patients**		**MV patients**		**SAP patients**		**Trauma patients**		**Nutrition therapy within 48 h**		**Nutrition therapy beyond 48 h**	
	**OR (95% CrI)**	**Rank**	**OR (95% CrI)**	**Rank**	**OR (95% CrI)**	**Rank**	**OR (95% CrI)**	**Rank**	**OR (95% CrI)**	**Rank**	**OR (95% CrI)**	**Rank**	**OR (95% CrI)**	**Rank**
Synbiotics	**0.37 (0.22, 0.61)**	**1**	**0.45 (0.26, 0.71)**	**1**	0.41 (0.15, 1.07)	2	**0.12 (0.02, 0.81)**	**1**	**0.13 (0.013, 0.81)**	**1**	**0.40 (0.23, 0.68)**	**1**	0.18 (0.01, 2.50)	1
Probioticsn	**0.52 (0.34, 0.77)**	**2**	**0.54 (0.36, 0.78)**	**2**	**0.49 (0.24, 0.90)**	**1**	0.63 (0.20, 1.61)	3	0.38 (0.01, 12.54)	2	**0.52 (0.33, 0.77)**	**2**	0.52 (0.07, 2.99)	2
Prebiotics	0.65 (0.35, 1.15)	3	0.76 (0.41, 1.34)	3	0.70 (0.22, 1.80)	3	0.32 (0.06, 1.59)	2	0.66 (0.05, 5.99)	3	0.67 (0.35, 1.19)	3	1.00 (0.04, 22.95)	3
EPN	Reference	4	Reference	4	Reference	4	Reference	4	Reference	4	Reference	4	Reference	4
TPN	**2.29 (1.48, 3.67)**	**5**	**1.57 (1.01, 2.56)**	**5**	1.31 (0.51, 3.87)	5	**3.93 (1.74, 9.15)**	**5**	–	–	**1.78 (1.04, 3.16)**	**5**	**3.70 (1.16, 13.52)**	**5**
Number of studies	42		32		12		11		5		34		8	
Participants	6,215		5,414		3,726		996		290		5,641		601

*CrI, credible interval; EPN, Enteral nutrition or adjuvant peripheral parenteral nutrition; MV, Mechanical ventilation; OR, odds ratio; SAP, Severe acute pancreatitis; TPN, Total parenteral nutrition. Bold indicate statistical significance*.

The sensitivity analysis was evaluated based on high-quality studies, and the results did not change substantially (Supplementary File 14).

### Risk of Bias Assessments and Grade for the Primary Outcome

In summary (Supplementary File 4), 1 (2%) of 55 rials was rated as high risk of bias, 23 (42%) trials were deemed moderate and 31 (56%) were considered low. We did not find publication bias for the network of outcomes, except duration of MV, hospital and ICU LOS (Supplementary File 10).

GRADE judgments for primary outcome were assessed and reported in Table 6. The certainty of evidence for the relative treatment effects of NI varied. It was high and moderate for most of the comparisons involving synbiotics, probiotics and prebiotics and low for most comparisons involving EPN and TPN. When subgroup analysis was performed, the GRADE between all comparisons and ranking of treatment was raised to at least moderate. Details of GRADE for secondary outcomes are presented in Supplementary File 11.

**Table 6:** Result of GRADE for nosocomial infection.

	**Nature of the evidence**	**Study limitations**	**Imprecision**	**Inconsistency**	**Indirectness**	**Publication bias**	**Confidence**	**Downgrading due to**
A vs. B	Indirect estimated	No downgrade	No downgrade	No downgrade	No downgrade	No downgrade	High	–
A vs. C	Mixed estimated	Downgrade because >70% contribution from moderate Rob comparisons	No downgrade	No downgrade	No downgrade	No downgrade	Moderate	Study limitations
A vs. D	Mixed estimated	Downgrade because >70% contribution from moderate Rob comparisons	No downgrade	Downgrade because pair heterogeneity *I*^2^ = 68.7%	No downgrade	No downgrade	Low	Study limitations Inconsistency
A vs. E	Indirect estimated	Downgrade because >70% contribution from moderate Rob comparisons	Downgrade because point estimate >1.0 but lower limit <0.80	No downgrade	No downgrade	No downgrade	Low	Study limitations Imprecision
B vs. C	Mixed estimated	No downgrade	No downgrade	No downgrade	No downgrade	No downgrade	High	Inconsistency
B vs. D	Mixed estimated	No downgrade	No downgrade	No downgrade Downgrade because sidesplitting *p* = 0.04143	No downgrade	No downgrade	Moderate	Inconsistency
B vs. E	Mixed estimated	No downgrade	No downgrade	No downgrade	No downgrade	No downgrade	High	–
C vs. D	Mixed estimated	Downgrade because >70% contribution from moderate Rob comparisons	Downgrade because point estimate >1.0 but lower limit <0.80	Downgrade because pair heterogeneity *I*^2^ = 57.4%	No downgrade	No downgrade	Very low	Study limitations Imprecision Inconsistency
C vs. E	Indirect estimated	Downgrade because >70% contribution from moderate Rob comparisons	No downgrade	No downgrade	No downgrade	No downgrade	Moderate	Study limitations
D vs. E	Mixed estimated	No downgrade	No downgrade	Downgrade because pair heterogeneity *I*^2^ = 76.4%	No downgrade	No downgrade	Moderate	Inconsistency
Ranking of treatments		Downgrade because >70% contribution from moderate Rob comparisons	No downgrade	Downgrade because global heterogeneity *I*^2^ = 62.02%	No downgrade	No downgrade	Low	Study limitations Inconsistency

*A, Synbiotic; B, Probiotic; C, Prebiotic; D, Enteral nutrition or adjuvant peripheral parenteral nutrition; E, Total parenteral nutrition*.

## Discussion

This study was based on the analysis of 55 RCTs enrolling 7,119 patients. Results indicated that synbiotic therapy was the best regimen in preventing NI in critically ill patients, while TPN exerted adverse curative effects amongst all the studied treatments. The sensitivity analyses for NI were consistent with the previous conclusions. Subgroup analysis based on diseases did not show significant heterogeneity between the included trials, and GRADE was moderate or high. These results further confirmed that the model was relevant and robust, making it applicable for use in clinical practice. Moreover, this analysis found that synbiotic therapy was the best regimen in improving HAP, CRBIS, UTI and sepsis. Probiotic and prebiotic treatments were the best regimens in shortening the duration of MV and preventing diarrhea, respectively. TPN was the worst in prolonging the hospital LOS.

Notably, this study differed from others in that it found no evidence that synbiotic therapies could reduce hospital and ICU mortality in critical patients (109). The mortality of critically ill patients was influenced by several complex risk factors (110). Probiotic and prebiotic therapy could not be fully absorbed by critically ill patients, so they may not have strong enough effects to reduce hospital and ICU mortality. Moreover, probiotic therapy did not significantly influence other clinical endpoints such as CRBIS, diarrhea and hospital LOS.

Results of subgroup analysis for the primary outcome were as follows. Firstly, subgroup analysis in different diseases showed that synbiotic therapy was the best treatment to improve NI in ICU patients. Conversely, TPN aggravated NI in ICU and SAP patients. These findings were consistent with the conclusions from NMA, thereby eliminating the effect of disease heterogeneity on the NMA outcome. Here, we focused on whether ICU patients can benefit from synbiotics. In addition, previous double-blind RCT and meta-analysis showed that TPN was associated with NI in ICU and SAP patients, which was consistent with the findings of this study. TPN therapy in ICU and SAP patients should be shortened as much as possible (25). Secondly, subgroup analysis in studies over the last 10 years and high quality showed that synbiotic therapy prevented NI, while TPN did not. These results were consistent with the standard analysis, including all studies in NMA, further confirming the robustness of the model and avoiding heterogeneity of the model. Thirdly, subgroup analysis in dosages of synbiotics showed no difference in the prevention of NI between the different doses. However, administered excessive synbiotic therapy not only failed to improve NI but also led to more infectious complications (16, 17). Hence, administered synbiotics in accordance with physiological requirements should be advocated to reduce the incidence of infectious complications. Fourthly, the subgroup of MV patients analysis showed that probiotic therapy can prevent NI. Only 3 out of a total of 12 studies administer synbiotics as the main intervention, and the patients involved were <10% of the total patients in this subgroup. Therefore, the power did not suggest that synbiotics can prevent NI. Finally, by adjusting the risk of NI and mortality through the initial nutrition therapy time, we found that synbiotics were associated with a reduction in NI among patients who were administered nutrition therapy within 48 h, and TPN were not associated with a reduction in NI, regardless of the time of nutrition therapy. This result suggests that we should administer initial enteral nutrition therapy within 48 h for critically ill adult patients (24, 25).

The primary finding of this study was inconsistent with results of previous studies. Many previous clinical trials, systematic reviews and meta-analysis efforts focused on whether symbiotics can improve NI in critically ill patients, and they rarely included probiotics. Moreover, those studies focused on the outcome of VAP (40, 111). In spite of promising data for probiotic use in reducing overall infections, the role of probiotics as a strategy to prevent VAP has been controversial (112). Recently, the results of the largest and most updated systematic review and meta-analysis demonstrated that probiotics are associated with a significant reduction in ICU-acquired infections and in the incidence of VAP. In addition, probiotics appeared to be more effective in reducing NI in patients at high risk of death than in patients at low and medium risk. However, such findings were limited by clinical heterogeneity and potential publication bias (42).

Although the mechanisms synbiotics were more effective than prebiotics and probiotics in preventing NI have not yet been clarified, the underlying mechanism areas discussed as follows: Firstly, synbiotics improve gut microbiota. Synbiotics not only increase the number of administered bacteria but also increase their genus groups and other microbiota, which could lead to the maintenance of gut microbiota (107). Secondly, synbiotics generate nutritional support for host epithelial cells. Synbiotic therapy had significantly increased levels of short-chain fatty acids are utilized mainly by intestinal epithelial cells as energy sources, The increased levels of short-chain fatty acids, especially acetate which might attenuate inflammation to reduce NI (60, 113). Thirdly, synbiotics maintain gut epithelial barrier. Increased levels of acetate and lactate might inhibit intraluminal toxins and maintain tight junctions (109). Finally, synbiotics regulate immune system function. Synbiotics regulates the innate and adaptive immune systems to reduce systemic inflammation and promote extra-intestinal organ function (109). These changes indicated that synbiotic therapy could have beneficial effects on reduce the development of NI (114, 115).

There were several strengths in this study. Firstly, this study was the first analysis using NMA to examine the effectiveness and determine the best choice of symbiotic regimen in improving NI in critically ill patients. This work helped us better assess the relative effects of treatment comparators in the absence of head-to-head trials. Secondly, our study is the most updated evaluation of the overall effects of symbiotic therapy in critically ill patients. It contained new suitable trials published on this topic since 1995 by focusing on NI. Thirdly, our study is the largest assessment of symbiotic therapy that included 55 RCTs published in both English and non-English languages from 24 countries, enrolling 7,119 patients. Fourthly, this study examined several relevant clinical outcomes in a heterogenous ICU patient population, including mixed ICU patients, MV patients, trauma patients, SAP patients and postoperative patients. Therefore, the results of this study helped reduce heterogeneity and potential publication bias and could be applied to a broad group of critically ill patients. Overall, all these factors increased the validity and robustness of our results.

Several limitations were still present in drawing strong treatment inferences. Firstly, the definitions of some diarrhea included our study were inconsistent because they are based on criteria of frequency, consistency (116), weight, duration and a combination of frequency and consistency. Such variations are rather vague and subject to different interpretations. There are at least 14 different definitions (117). Making those different definitions consistent is difficult. We were also unable to perform further grouping analysis because of the limited number of studies. Analogously, the definition of prebiotics more or less overlapped with the definition of dietary fiber. In addition, some studies did not provide the accurate definitions of study outcomes. We acknowledge potential misclassification and inconsistency, which is one of the reasons why we downgraded the GRADE of those secondary outcomes. Moreover, the variety of synbiotic strains and length of administration of therapy amongst the different trials weakened any possible clinical conclusions and recommendations. Given the limited number of studies evaluating each endpoint, we were unable to perform subgroup analysis for all clinical outcomes. A further limitation is that the quality of many comparisons was assessed as low or very low level of evidence for hospital LOS, ICU LOS, and duration of MV. Hence, the inferences from current findings were weakened. Lastly, the generalizability of results was limited to other populations as nearly 90% of all studies came from Asia and Europe countries. In addition to the above limitations, we acknowledge potential heterogeneity among critically ill patients in different trials. We have conducted subgroup analysis from many aspects such as different diseases populations, initial time of nutrition therapy, and strive to minimize heterogeneity.

A multicentre, concealed, randomized, stratified, blinded, controlled trial (111) to evaluate the effect of probiotics on VAP and other ICU-acquired infections in 2,650 critically ill patients is ongoing in Canada, USA and Saudi Arabia (clinical trials. gov. registration NCT02462590). REVISE Trials are also ongoing in North America, Australia and Saudi Arabia. The results of these trials will provide further information about the curative effect on symbiotics in the ICU.

## Conclusion

This systematic review and NMA provide evidence that synbiotic therapy ranked first over probiotics, prebiotics, EPN and TPN to prevent NI in critically ill adult patients. Conversely, TPN therapy significantly increased NI in the critically ill compared with other therapies. Physicians in critical care and related disciplines should consider the use of synbiotics as an adjunctive therapy to improve NI amongst critically ill adult patients. At the same time, the duration of TPN alone should be reduced to decrease NI, especially in ICU and SPA patients. However, on the basis of current data, there is not currently sufficient evidence to make a final strong recommendation for synbiotic therapy to be utilized in the improvement of NI in the critically ill. Numerous questions remain unanswered about a variety of synbiotic strains, wide range of daily doses and duration of therapy; such topics can be addressed in future work.

## Data Availability Statement

The original contributions presented in the study are included in the article/Supplementary Material, further inquiries can be directed to the corresponding author/s.

## Author Contributions

CL, YY, and HQ had the idea for and designed the study. YH, LL, SL, and JX supervised the study. CL, ZG, JZ, HC, SM, AL, MM, DC, and CW did search clinical trials, study select, data extract, and statistical analysis. CL wrote the manuscript. All authors contributed to acquisition, analysis, interpretation of data, revised the report, and approved the final version before submission.

## Conflict of Interest

The authors declare that the research was conducted in the absence of any commercial or financial relationships that could be construed as a potential conflict of interest.

## Abbreviations

BSIs: bloodstream infection
CENTRAL: Cochrane Central Register for Controlled Trials
CFU: Colony-forming units
CRBSI: Catheter-related bloodstream infection
CrI: Credible interval
DB: Double-blind
ECMO: Extracorporeal membrane oxygenation
EN: Enteral nutrition
EPN: Enteral nutrition or adjuvant peripheral parenteral nutrition
GCS: Glasgow coma scale
GRADE: Grades of Recommendation, Assessment, Development and Evaluation
HAP: Hospital acquired pneumonia
ICU: Intensive care unit
LOS: Length of stay
MC: Multi-center
MD: Mean difference
MV: Mechanical ventilation
NI: Nosocomial infection
NMA: Network meta-analysis
NR: Not reported
OP: Open study
OR: Odds ratio
PN: Parenteral nutrition
PRISMA: Preferred Reporting Items for Systematic Review and Meta-analyses
PROSPERO: Prospective register of systematic reviews
RCTs: Randomized controlled trial studies
RR: Risk ratio
SAP: severe acute pancreatitis
SB: Single-blind
SC: single-center
SD: Standard deviation
SUCRA: Surface under the cumulative ranking curve
TPN: Total parenteral nutrition
UTI: Urinary tract infection
VAP: Ventilator-associated pneumonia.

## References

[1] Jaradat RW Lahlouh AB Alshogran OY Aldabbour BA Balusha AA. Nosocomial infections among patients with intracranial hemorrhage: a retrospective data analysis of predictors and outcomes. Clin Neurol Neurosurg. (2019) 182:158–66. 10.1016/j.clineuro.2019.05.016 31151044

[2] Baviskar AS Khatib KI Rajpal D Dongare HC Nosocomial infections in surgical intensive care unit: a retrospective single-center study. Int J Crit Illn Inj Sci. (2019) 9:16–20. 10.4103/IJCIIS.IJCIIS_57_18 30989063 PMC6423928

[3] Spalding MC Cripps MW Minshall CT. Ventilator-associated pneumonia: new definitions. Crit Care Clin. (2017) 33:277–92. 10.1016/j.ccc.2016.12.009 28284295 PMC7127414

[4] MacLaren G Schlapbach LJ Aiken AM. Nosocomial infections during extracorporeal membrane oxygenation in neonatal, pediatric, and adult patients: a comprehensive narrative review. Pediatr Crit Care Med. (2019) 21:283–90. 10.1097/PCC.0000000000002190 31688809

[5] Reintam Blaser A Preiser JC Fruhwald S Wilmer A Wernerman J Benstoem C. Gastrointestinal dysfunction in the critically ill: a systematic scoping review and research agenda proposed by the Section of Metabolism, Endocrinology and Nutrition of the European Society of Intensive Care Medicine. Critical Care. (2020) 24:224. 10.1186/s13054-020-02889-4 32414423 PMC7226709

[6] Latorre M Krishnareddy S Freedberg DE. Microbiome as mediator: Do systemic infections start in the gut? World J Gastroenterol. (2015) 21:10487–10492. 10.3748/wjg.v21.i37.10487 26457009 PMC4588071

[7] Asrani VM Brown A Huang W Bissett I Windsor JA. Gastrointestinal dysfunction in critical illness: a review of scoring tools. JPEN J Parenteral Enteral Nutr. (2020) 44:182–96. 10.1002/jpen.1679 31350771

[8] Wischmeyer PE McDonald D Knight R. Role of the microbiome, probiotics, and 'dysbiosis therapy' in critical illness. Curr Opin Crit Care. (2016) 22:347–53. 10.1097/MCC.0000000000000321 27327243 PMC5065053

[9] Alverdy JC Chang EB. The re-emerging role of the intestinal microflora in critical illness and inflammation: why the gut hypothesis of sepsis syndrome will not go away. J Leukoc Biol. (2008) 83:461–6. 10.1189/jlb.0607372 18160538

[10] Vincent JL Rello J Marshall J Silva E Anzueto A Martin CD. International study of the prevalence and outcomes of infection in intensive care units. JAMA. (2009) 302:2323–9. 10.1001/jama.2009.1754 19952319

[11] Kallel H Dammak H Bahloul M Ksibi H Chelly H Ben Hamida C. Risk factors outcomes of intensive care unit-acquired infections in a Tunisian ICU. Med Sci Monit. (2010) 16:69–75. 10.1016/j.mehy.2009.07.02-6 20671622

[12] Vincent JL Sakr Y Sprung CL Ranieri VM Reinhart K Gerlach H. Sepsis in European intensive care units: results of the SOAP study. Crit Care Med. (2006) 34:344–53. 10.1097/01.CCM.0000194725.48928.3A 16424713

[13] Siegel T Mikaszewska-Sokolewicz M Mayzner-Zawadzka E. Epidemiology of infections at the intensive care unit. Pol Merkur Lekarski. (2006) 20:309–14. 16780263

[14] Rosenthal VD Guzman S Orellano PW. Nosocomial infections in medical-surgical intensive care units in Argentina: attributable mortality and length of stay. Am J Infect Control. (2003) 31:291–5. 10.1067/mic.2003.1 12888765

[15] Bercault N Boulain T. Mortality rate attributable to ventilator-associated nosocomial pneumonia in an adult intensive care unit: a prospective case-control study. Crit Care Med. (2001) 29:2303–9. 10.1097/00003246-200112000-00012 11801831

[16] Suez J Zmora N Segal E Elinav E. The pros, cons, and many unknowns of probiotics. Nat Med. (2019) 25:716–29. 10.1038/s41591-019-0439-x 31061539

[17] Hill C Guarner F Reid G Gibson GR Merenstein DJ Pot B. The International Scientific Association for Probiotics and Prebiotics consensus statement on the scope and appropriate use of the term probiotic. Nat Rev Gastroenterol Hepatol. (2014) 11:506–14. 10.1038/nrgastro.2014.66 24912386

[18] Gibson GR Hutkins R Sanders ME Prescott SL Reimer RA Salminen SJ. Expert consensus document: the International Scientific Association for Probiotics and Prebiotics (ISAPP) consensus statement on the definition and scope of prebiotics. Nat Rev Gastroenterol Hepatol. (2017) 14:491–502. 10.1038/nrgastro.2017.75 28611480

[19] Canfora EE Jocken JW Blaak EE. Short-chain fatty acids in control of body weight and insulin sensitivity. Nat Rev Endocrinol. (2015) 11:577–91. 10.1038/nrendo.2015.128 26260141

[20] Koh A De Vadder F Kovatcheva-Datchary P Backhed F. From dietary fiber to host physiology: short-chain fatty acids as key bacterial metabolites. Cell. (2016) 165:1332–45. 10.1016/j.cell.2016.05.041 27259147

[21] Roberfroid M Gibson GR Hoyles L McCartney AL Rastall R Rowland I. Prebiotic effects: metabolic and health benefits. Br J Nutr. (2010) 104:S1-63. 10.1017/S0007114510003363 20920376

[22] O'Keefe SJ. Diet, microorganisms and their metabolites, and colon cancer. Nat Rev Gastroenterol Hepatol. (2016) 13:691–706. 10.1038/nrgastro.2016.165 27848961 PMC6312102

[23] Pluznick JL. Gut microbiota in renal physiology: focus on short-chain fatty acids and their receptors. Kidney Int. (2016) 90:1191–8. 10.1016/j.kint.2016.06.033 27575555 PMC5123942

[24] McClave SA Taylor BE Martindale RG Warren MM Johnson DR Braunschweig C. Guidelines for the Provision and Assessment of Nutrition Support Therapy in the Adult Critically Ill Patient: Society of Critical Care Medicine (SCCM) and American Society for Parenteral and Enteral Nutrition (A.S.P.E.N.). JPEN J Parenteral Enteral Nutr. (2016) 40:159–211. 10.1177/0148607115621863 26773077

[25] Singer P Blaser AR Berger MM Alhazzani W Calder PC Casaer MP. ESPEN guideline on clinical nutrition in the intensive care unit. Clin Nutr. (2019) 38:48–79. 10.1016/j.clnu.2018.08.037 30348463

[26] Knight DJ Gardiner D Banks A Snape SE Weston VC Bengmark S. Effect of synbiotic therapy on the incidence of ventilator associated pneumonia in critically ill patients: a randomised, double-blind, placebo-controlled trial. Intens Care Med. (2009) 35:854–61. 10.1007/s00134-008-1368-1 19083199

[27] Jain PK McNaught CE Anderson AD MacFie J Mitchell CJ. Influence of synbiotic containing Lactobacillus acidophilus La5, Bifidobacterium lactis Bb 12, Streptococcus thermophilus, Lactobacillus bulgaricus and oligofructose on gut barrier function and sepsis in critically ill patients: a randomised controlled trial. Clin Nutr. (2004) 23:467–75. 10.1016/j.clnu.2003.12.002 15297081

[28] McNaught CE Woodcock NP Anderson AD MacFie J. A prospective randomised trial of probiotics in critically ill patients. Clin Nutr. (2005) 24:211–9. 10.1016/j.clnu.2004.08.008 15784480

[29] Morrow LE Kollef MH Casale TB. Probiotic prophylaxis of ventilator-associated pneumonia: a blinded, randomized, controlled trial. Am J Respir Crit Care Med. (2010) 182:1058–64. 10.1164/rccm.200912-1853OC 20522788 PMC2970846

[30] Sharma B Srivastava S Singh N Sachdev V Kapur S Saraya A. Role of probiotics on gut permeability and endotoxemia in patients with acute pancreatitis: a double-blind randomized controlled trial. J Clin Gastroenterol. (2011) 45:442–8. 10.1097/MCG.0b013e318201f9e2 21135704

[31] Mahmoodpoor A Hamishehkar H Asghari R Abri R Shadvar K Sanaie S. Effect of a probiotic preparation on ventilator-associated pneumonia in critically ill patients admitted to the intensive care unit: a prospective double-blind randomized controlled trial. Nutr Clin Pract. (2019) 34:156–62. 10.1002/ncp.10191 30088841

[32] Zeng J Wang CT Zhang FS Qi F Wang SF Ma S. Effect of probiotics on the incidence of ventilator-associated pneumonia in critically ill patients: a randomized controlled multicenter trial. Intens Care Med. (2016) 42:1018–28. 10.1007/s00134-016-4303-x 27043237

[33] Besselink MGH van Santvoort HC Buskens E Boermeester MA van Goor H Timmerman HM. Probiotic prophylaxis in predicted severe acute pancreatitis: a randomised, double-blind, placebo-controlled trial. Lancet. (2008) 371:651–9. 10.1016/S0140-6736(08)60207-X 18279948

[34] Zhu YM Lin S Dang XW Wang M Li L Sun RQ. Effects of probiotics in treatment of severe acute pancreatitis. World Chinese J Digestol. (2014). 22:5013–17. 10.11569/wcjd.v22.i32.5013

[35] Didari T Solki S Mozaffari A Nikfar S Abdollahi M. A systematic review of the safety of probiotics. Expert Opin Drug Saf. (2014) 13:227–39. 10.1517/14740338.2014.872627 24405164

[36] Carvour ML Wilder SL Ryan KL Walraven C Qeadan F Brett M. Predictors of *Clostridium difficile* infection and predictive impact of probiotic use in a diverse hospital-wide cohort. Am J Infect Control. (2019) 47:2–8. 10.1016/j.ajic.2018.07.014 30205907 PMC6321775

[37] Van den Nieuwboer M Brummer RJ Guarner F Morelli L Cabana M Claasen E. The administration of probiotics and synbiotics in immune compromised adults: is it safe? Benef Microbes. (2015) 6:3–17. 10.3920/BM2014.0079 25304690

[38] Barraud D Bollaert PE Gibot S. Impact of the administration of probiotics on mortality in critically ill adult patients: a meta-analysis of randomized controlled trials. Chest. (2013) 143:646–55. 10.1378/chest.12-1745 23460153

[39] Arumugam S Lau CS Chamberlain RS. Probiotics and synbiotics decrease postoperative sepsis in elective gastrointestinal surgical patients: a meta-analysis. J Gastrointest Surg. (2016) 20:1123–31. 10.1007/s11605-016-3142-y 27073082

[40] Bo L Li J Tao T Bai Y Ye X Hotchkiss RS. Probiotics for preventing ventilator-associated pneumonia. Cochrane Database Syst Rev. (2004) 2014:CD009066. 10.1002/14651858.CD009066.pub2 PMC4283465 25344083

[41] Watkinson PJ Barber VS Dark P Young JD. The use of pre- pro- and synbiotics in adult intensive care unit patients: systematic review. Clin Nutr. (2007) 26:182–92. 10.1016/j.clnu.2006.07.010 17011083

[42] Manzanares W Lemieux M Langlois PL Wischmeyer PE. Probiotic and synbiotic therapy in critical illness: a systematic review and meta-analysis. Crit Care. (2016) 19:262. 10.1186/s13054-016-1434-y 27538711 PMC4991010

[43] Lu G Ades AE. Combination of direct and indirect evidence in mixed treatment comparisons. Stat Med. (2004) 23:3105–24. 10.1002/sim.1875 15449338

[44] Moher D Liberati A Tetzlaff J Altman DG. Preferred reporting items for systematic reviews and meta-analyses: the PRISMA statement. Plos Med. (2009) 6:e1000097. 10.1371/journal.pmed.1000097 19621072 PMC2707599

[45] Higgins JPT Green S. Cochrane Handbook for Systematic Reviews of Interventions Version 5.1.0 [updated March 2011]. Available online at: http://handbook.cochrane.org(accessed December 15, 2012).

[46] Horan TC Andrus M Dudeck MA. CDC/NHSN surveillance definition of health care-associated infection and criteria for specific types of infections in the acute care setting. Am J Infect Control. (2008) 36:309–32. 10.1016/j.ajic.2008.03.002 18538699

[47] Higgins JP Altman DG Gotzsche PC Juni P Moher D Oxman AD. The Cochrane Collaboration's tool for assessing risk of bias in randomised trials. BMJ. (2011) 343:d5928. 10.1136/bmj.d5928 22008217 PMC3196245

[48] Chaimani A Higgins JPT Mavridis D Spyridonos P Salanti G. Graphical tools for network meta-analysis in STATA. PloS ONE. (2013) 8:e76654. 10.1371/journal.pone.0076654 24098547 PMC3789683

[49] Salanti G Del Giovane C Chaimani A Caldwell DM Higgins JP. Evaluating the quality of evidence from a network meta-analysis. PloS ONE. (2014) 9:e99682. 10.1371/journal.pone.0099682 24992266 PMC4084629

[50] Song F Harvey I Lilford R. Adjusted indirect comparison may be less biased than direct comparison for evaluating new pharmaceutical interventions. J Clin Epidemiol. (2008) 61:455–63. 10.1016/j.jclinepi.2007.06.006 18394538

[51] DerSimonian R. Meta-analysis in the design and monitoring of clinical trials. Stat Med. (1996) 15:1237–48. 8817798 10.1002/(SICI)1097-0258(19960630)15:12<1237::AID-SIM301>3.0.CO;2-N

[52] Gelman A Robin DB. Markov Chain Monte Carlo methods in biostatistics. Stat Methods Med Res. (1996) 5:339–55. 10.1177/096228029600500402 9004377

[53] Salanti G Ades AE Ioannidis JP. Graphical methods and numerical summaries for presenting results from multiple-treatment meta-analysis: an overview and tutorial. J Clin Epidemiol. (2011) 64:163–71. 10.1016/j.jclinepi.2010.03.016 20688472

[54] DerSimonian R Laird N. Meta-analysis in clinical trials. Control Clin Trials. (1986) 7:177–88. 10.1016/0197-2456(86)90046-2 3802833

[55] Higgins JP Jackson D Barrett JK Lu G Ades AE White IR. Consistency and inconsistency in network meta-analysis: concepts and models for multi-arm studies. Res Synth Methods. (2012) 3:98–110. 10.1002/jrsm.1044 26062084 PMC4433772

[56] Higgins JP Thompson SG Deeks JJ Altman DG. Measuring inconsistency in meta-analyses. BMJ. (2003) 327:557–60. 10.1136/bmj.327.7414.557 12958120 PMC192859

[57] Dias S Welton NJ Caldwellb DM Adesa AE. Checking consistency in mixed treatment comparison meta-analysis. Stat Med. (2010) 29:932–44. 10.1002/sim.3767 20213715

[58] Furukawa TA Salanti G Atkinson LZ Leucht S Ruhe HG Turner EH. Comparative efficacy and acceptability of first-generation and second-generation antidepressants in the acute treatment of major depression_ protocol for a network meta-analysis. BMJ Open. (2016) 6:e010919. 10.1136/bmjopen-2015-010919 27401359 PMC4947714

[59] Bian L Nagata S Asahara T. Effects of the continuous intake of Lactobacillus casei strain Shirota-fermented milk on risk management of long-term inpatients at health service facilities for the elderly. Int J Probiot Prebiot. (2011) 6:123–31.

[60] Kanazawa H Nagino M Kamiya S Komatsu S Mayumi T Takagi K. Synbiotics reduce postoperative infectious complications: a randomized controlled trial in biliary cancer patients undergoing hepatectomy. Langenbeck's Arch Surg. (2005) 390:104–13. 10.1007/s00423-004-0536-1 15711820

[61] Shimizu K Ogura H Hamasaki T Goto M Tasaki O Asahara T. Altered gut flora are associated with septic complications and death in critically ill patients with systemic inflammatory response syndrome. Digest Dis Sci. (2011) 56:1171–7. 10.1007/s10620-010-1418-8 20931284 PMC3059822

[62] Braga M Vignali A Gianotti L Cestari A Profili M Di Carlo V. Benefits of early postoperative enteral feeding in cancer patients. Infusionsther Transfusionsmed. (1995) 22:280–4. 10.1159/000223143 8924741

[63] Kudsk KA Minard G Croce MA Brown RO Lowrey TS Pritchard FE. A randomized trial of isonitrogenous enteral diets after severe trauma: an immune-enhancing diet reduces septic complications. Ann Surg. (1996) 224:531–43. 10.1097/00000658-199610000-00011 8857857 PMC1235418

[64] Bleichner G Blehaut H Mentec H Moyse D. *Saccharomyces boulardii* prevents diarrhea in critically ill tube-fed patients. Intens Care Med. (1997). 23:517–23. 10.1007/s001340050367 9201523

[65] Falcão De Arruda IS De Aguilar-Nascimento JE. Benefits of early enteral nutrition with glutamine and probiotics in brain injury patients. Clin Sci. (2004) 106:287–92. 10.1042/CS20030251 14558885

[66] Lu X Han CM Yu JX Fu SZ. Preliminary comparative study on the effects of early enteral supplementation of synbiotics on severely burned patients. Chin J Burns. (2004) 20:198–201. 15447816

[67] Sun B Gao Y Xu J Zhou XL Zhou ZQ Liu C. Role of individually staged nutritional support in the management of severe acute pancreatitis. Hepatobil Pancreat Dis Int. (2004) 3:458–63. 15313689

[68] Klarin B Johansson ML Molin G Larsson A Jeppsson B. Adhesion of the probiotic bacterium *Lactobacillus plantarum* 299v onto the gut mucosa in critically ill patients: a randomised open trial. Crit Care. (2005) 9:R285–93. 10.1186/cc3522 15987403 PMC1175894

[69] Morrow LE Kollef MH Bowers JB Casale TB. Probiotic manipulation of the native flora in critically ill patients: an opportunity for ventilator-associated pneumonia prophylaxis? Chest. (2005) 128:144S. 10.1378/chest.128.4_MeetingAbstracts.144S

[70] Kotzampassi K Giamarellos-Bourboulis EJ Voudouris A Kazamias P Eleftheriadis E. Benefits of a synbiotic formula (Synbiotic 2000Forte) in critically ill trauma patients: earlyresults of a randomized controlled trial. World J Surg. (2006) 30:1848–55. 10.1007/s00268-005-0653-1 16983476

[71] Petrov MS Kukosh MV Emelyanov NV. A randomized controlled trial of enteral versus parenteral feeding in patients with predicted severe acute pancreatitis shows a significant reduction in mortality and in infected pancreatic complications with total enteral nutrition. Digesti Surg. (2006) 23:336–44; discussion 344–335. 10.1159/000097949 17164546

[72] Spindler-Vesel A Bengmark S Vovk I Cerovic O Kompan L. Synbiotics, prebiotics, glutamine, or peptide in early enteral nutrition: a randomized study in trauma patients. J Parenteral Enteral Nutr. (2007) 31:119–26. 10.1177/0148607107031002119 17308252

[73] Abdulmeguid AM Hassan A. Enteral versus parenteral nutrition in mechanically ventilated patients. Neurol Croatica. (2007) 56:15–24. 21242788

[74] Alberda C Gramlich L Meddings J Field C McCargar L Kutsogiannis D. Effects of probiotic therapy in critically ill patients: a randomized, double-blind, placebo-controlled trial. Am J Clin Nutr. (2007) 85:816–23. 10.1093/ajcn/85.3.816 17344505

[75] Casas M Mora J Fort E Farré A Aracil C Busquets D. Total enteral nutrition vs. total parenteral nutrition in patients with severe acute pancreatitis. Rev Esp Enferm Dig. (2007) 99:264–9. 10.4321/S1130-01082007000500004 17650935

[76] Karakan T Ergun M Dogan I Cindoruk M Unal S. Comparison of early enteral nutrition in severe acute pancreatitis with prebiotic fiber supplementation versus standard enteral solution: a prospective randomized double-blind study. World J Gastroenterol. (2007) 21:2733–7. 10.3748/wjg.v13.i19.2733 17569144 PMC4147124

[77] Oláh A Belágyi T Pótó L Romics L Jr. Bengmark S. Synbiotic control of inflammation and infection in severe Acute Pancreatitis: a prospective, randomized, double blind study. Hepatogastroenterology. (2007) 54:590–4. 17523328

[78] Sramek V Dadak L Stouracova M Stetka P Kyr M Ticha A. Impact of addition of synbiotics (Synbiotic (2000). Forte) to enteral nutrition on the course of MODS, occurrence of sepsis, immune status and gut function in long-term critically ill patients. Anesteziol Intenzivni Med. (2007) 18:157–63.

[79] Forestier C Guelon D Cluytens V Gillart T Sirot J De Champs C. Oral probiotic and prevention of Pseudomonas aeruginosa infections: a randomized, double-blind, placebo-controlled pilot study in intensive care unit patients. Critical care. (2008) 12:R69. 10.1186/cc6907 18489775 PMC2481460

[80] Klarin B Wullt M Palmquist I Molin G Larsson A Jeppsson B. Lactobacillus plantarum 299v reduces colonisation of *Clostridium* difficile in critically ill patients treated with antibiotics. Acta Anaesthesiol Scand. (2008) 52:1096–102. 10.1111/j.1399-6576.2008.01748.x 18840110

[81] Doley RP Wig TDYJ Kochhar R Singh G Bharathy KGS Kudari A. Enteral nutrition in severe acute pancreatitis. J Pancreas. (2009) 10:157–62. 10.1115/1.1456090 19287109

[82] Giamarellos-Bourboulis EJ Bengmark S Kanellakopoulou K Kotzampassi K. Pro- and synbiotics to control inflammation and infection in patients with multiple injuries. J Trauma. (2009) 67:815–21. 10.1097/TA.0b013e31819d979e 19820590

[83] Moses V Mahendri NV John G Peter JV Ganesh A. Early hypocaloric enteral nutritional supplementation in acute organophosphate poisoning–a prospective randomized trial. Clin Toxicol. (2009) 47:419–24. 10.1080/15563650902936664 19492933

[84] Barraud D Blard C Hein F Marcon O Cravoisy A Nace L. Probiotics in the critically ill patient: a double blind, randomized, placebo-controlled trial. Intens Care Med. (2010) 36:1540–7. 10.1007/s00134-010-1927-0 20502866

[85] Frohmader TJ Chaboyer WP Robertson IK Gowardman J. Decrease in frequency of liquid stool in enterally fed critically ill patients given the multispecies probiotic VSL#3: a pilot trial. Am J Cri Care. (2010) 19:e1–11. 10.4037/ajcc2010976 20436058

[86] Ferrie S Daley M. *Lactobacillus* GG as treatment for diarrhea during enteral feeding in critical illness: randomized controlled trial. JPEN J Parenteral Enteral Nutr. (2011) 35:43–9. 10.1177/0148607110370705 20978244

[87] Tan M Zhu JC Du J Zhang LM Yin HH. Effects of probiotics on serum levels of Th1/Th2 cytokine and clinical outcomes in severe traumatic brain-injured patients: a prospective randomized pilot study. Crit Care. (2011) 15:R290. 10.1186/cc10579 22136422 PMC3388628

[88] Hayakawa M Asahara T Ishitani T Okamura A Nomoto K Gando S. Synbiotic therapy reduces the pathological gram-negative rods caused by an increased acetic acid concentration in the gut. Digest Dis Sci. (2012) 57:2642–9. 10.1007/s10620-012-2201-9 22576712

[89] Malian M Reichenbach R Peck A Pamukov N. Probiotic supplementation in critical care. Crit Care Med. (2012) 40:1–328. 10.1097/01.ccm.0000425300.89190.4b23213646

[90] Plaudis H Pupelis G Zeiza K Boka V. Early low volume oral synbiotic/prebiotic supplemented enteral stimulation of the gut in patients with severe acute pancreatitis:a prospective feasibility study. Acta Chir Belg. (2012) 112:131–8. 10.1080/00015458.2012.11680811 22571076

[91] Cui LH Xiao-hui W Li-hua P Lan Y Yun-sheng Y. The effects of early enteral nutrition with addition of probiotics on the prognosis of patients suffering from severe acute pancreatitis. Chin Crit Care Med. (2013) 25:224–8. 10.3760/cma.j.issn.2095-4352.2013.04.011 23660099

[92] Elke G Kuhnt E Ragaller M Schadler D Frerichs I Brunkhorst FM. Enteral nutrition is associated with improved outcome in patients with severe sepsis: a secondary analysis of the VISEP trial. Med Klin Intensivmed Notfallmed. (2013) 108:223–33. 10.1007/s00063-013-0224-4 23455443

[93] Tan M Xiao-lan L Jun-wei D Hua P Jing-ci Z. Effects of probiotics on blood glucose levels and clinical outcomes in patients witn severe craniocerebral trauma. Chin Crit Care Med. (2013) 25:627–30. 10.3760/cma.j.issn.2095-4352.2013.10.012 24119702

[94] Wang G Wen J Xu L Zhou S Gong M Wen P. Effect of enteral nutrition and ecoimmunonutrition on bacterial translocation and cytokine production in patients with severe acute pancreatitis. J Surg Res. (2013) 183:592–7. 10.1016/j.jss.2012.12.010 23726433

[95] Lopez de Toro I Sanchez-Casado M Pérez-Pedrero Sánchez-Belmonte MJ López-Reina Torrijos P Sánchez-Rodriguez P Raigal-Caño A. The influence of symbiotics in multi-organ failure: randomised trial. Med Clin. (2014) 143:143–9. 10.1016/j.medcli.2013.09.046 24560584

[96] Sanaie S Ebrahimi-Mameghani M Hamishehkar H Mojtahedzadeh M Mahmoodpoor A. Effect of a multispecies probiotic on inflammatory markers in critically ill patientss: a randomized, double-blind, placebo-controlled trial. J Res Med Sci. (2014) 19:827–33. 25535496 PMC4268190

[97] Fu Y-H Jian-Bo W Gui-Liang W Ping W Min G Ming H. Effect of enteral nutrition on cytokine production and plasma endotoxin in patients with severe acute pancreatitis. World Chin J Digestol. (2015) 23:1174. 10.11569/wcjd.v23.i7.117423726433

[98] Kim JM Joh JW Kim HJ Kim SH Rha M Sinn DH. Early enteral feeding after living donor liver transplantation prevents infectious complications: a prospective pilot study. Medicine. (2015) 94:e1771. 10.1097/MD.0000000000001771 26554774 PMC4915875

[99] Rongrungruang Y Krajangwittaya D Pholtawornkulchai K Tiengrim S Thamlikitkul V. Randomized controlled study of probiotics containing *Lactobacillus casei* (Shirota strain) for prevention of ventilator-associated pneumonia. J Med Assoc Thai. (2015) 98:253–9. 25920295

[100] Fan M-C Qiao-ling W Wei F Yun-xia J Lian-di L Sun P. Early enteral combined with parenteral nutrition treatment for severe traumatic brain injury: effects on immune function, nutritional status and outcomes. Chin Med Sci J. (2016) 31:213–20. 10.1016/S1001-9294(17)30003-2 28065217

[101] Malik AA Rajandram R Tah PC Hakumat-Rai VR Chin KF. Microbial cell preparation in enteral feeding in critically ill patients: a randomized, double-blind, placebo-controlled clinical trial. J Crit Care. (2016) 32:182–88. 10.1016/j.jcrc.2015.12.008 26777745

[102] Zarinfar N Sharafkhah M Amiri M Rafeie M. Probiotic effects in prevention from ventilator-associated pneumonia. Koomesh. (2016) 7:803–13.

[103] Alberda C Marcushamer S Hewer T Journault N Kutsogiannis D. Feasibility of a *Lactobacillus casei* drink in the intensive care unit for prevention of antibiotic associated diarrhea and *Clostridium difficile*. Nutrients. (2018) 10:539. 10.3390/nu10050539 PMC5986419 29701662

[104] Fazilaty Z Chenari H Shariatpanahi ZV. Effect of β-glucan on serum levels of IL-12, hs-CRP, and clinical outcomes in multiple-trauma patients: a prospective randomized study. Turkish J Trauma Emerg Surg. (2018) 24:287–93. 10.5505/tjtes.2017.34514 30028484

[105] Kooshki AZK Zarghi A Rad M Tabaraie Y. Prebiotic prophylaxis of ventilator-associated pneumonia: a randomized clinical trial. Biomed Res Ther. (2018) 5:2287–95. 10.15419/bmrat.v5i5.442

[106] Reignier J Boisramé-Helms J Brisard L Lascarrou J-B Ait Hssain A Anguel N. Enteral versus parenteral early nutrition in ventilated adults with shock: a randomised, controlled, multicentre, open-label, parallel-group study (NUTRIREA-2). Lancet. (2018) 391:133–43. 10.1016/S01406736(17)32146-3 29128300

[107] Shimizu K Yamada T Ogura H Mohri T Kiguchi T Fujimi S. Synbiotics modulate gut microbiota and reduce enteritis and ventilator-associated pneumonia in patients with sepsis: a randomized controlled trial. Crit Care. (2018) 22:239. 10.1186/s13054-018-2167-x 30261905 PMC6161427

[108] Tuncay P Arpaci F Doganay M Erdem D Sahna A Ergun H. Use of standard enteral formula versus enteric formula with prebiotic content in nutrition therapy: a randomized controlled study among neuro-critical care patients. Clin Nutr ESPEN. (2018) 25:26–36. 10.1016/j.clnesp.2018.03.123 29779815

[109] Davison JM Wischmeyer PE. Probiotic and synbiotic therapy in the critically ill: state of the art. Nutrition. (2019) 59:29–36. 10.1016/j.nut.2018.07.017 30415160

[110] Lherm T Monet C Nougiere B Soulier M Larbi D Le Gall C. Seven cases of fungemia with *Saccharomyces boulardii* in critically ill patients. Intens Care Med. (2002) 28:797–801. 10.1007/s00134-002-1267-9 12107689

[111] Johnstone J Heels-Ansdell D Thabane L Meade M Marshall J Lauzier F. Evaluating probiotics for the prevention of ventilator-associated pneumonia: a randomised placebo-controlled multicentre trial protocol and statistical analysis plan for PROSPECT. BMJ Open. (2019) 9:e025228. 10.1136/bmjopen-2018-025228 31227528 PMC6596980

[112] Morrow LE Gogineni V Malesker MA. Synbiotics and probiotics in the critically ill after the PROPATRIA trial. Curr Opin Clin Nutr Metab Care. (2012) 15:147–50. 10.1097/MCO.0b013e32834fcea8 22248590

[113] Asahara T Shimizu K Nomoto K Hamabata T Ozawa A Takeda Y. Probiotic bifidobacteria protect mice from lethal infection with Shiga toxin-producing *Escherichia coli* O157:H7. Infect Immunity. (2004) 72:2240–7. 10.1128/IAI.72.4.2240-2247.2004 15039348 PMC375161

[114] Asahara T Takahashi A Yuki N Kaji R Takahashi T Nomoto K. Protective effect of a synbiotic against multidrug-resistant *Acinetobacter baumannii* in a murine infection model. Antimicrob Agents Chemother. (2016) 60:3041–50. 10.1128/AAC.02928-15 26953197 PMC4862511

[115] Shimizu K Ogura H Goto M Asahara T Nomoto K Morotomi M. Synbiotics decrease the incidence of septic complications in patients with severe SIRS: a preliminary report. Digest Dis Sci. (2008) 54:1071–8. 10.1007/s10620-008-0460-2 18726154

[116] De Brito-Ashurst I Preiser JC. Diarrhea in critically ill patients: the role of enteral feeding. JPEN J Parenteral Enteral Nutr. (2016) 40:913–23. 10.1177/0148607116651758 27271709

[117] Bliss DZ Guenter PA Settle RG. Defining and reporting diarrhea in tube-fed patients-what a mess! Am J Clin Nutr. (1992) 55:753–9. 10.1093/ajcn/55.3.753 1550053

