# Supplementary material for: Synbiotic Therapy Prevents Nosocomial Infection in Critically Ill Adult Patients: A Systematic Review and Network Meta-Analysis of Randomized Controlled Trials Based on a Bayesian Framework
Source: Front Med (Lausanne). 2021 Jul 15;8:693188. doi: 10.3389/fmed.2021.693188 (PMC8321544; doi:10.3389/fmed.2021.693188)
Supplement: Supplementary file 1 [file Data_Sheet_1.pdf]

Appendix file

Synbiotic Therapy prevents nosocomial infection in critically ill adult patients: A Systematic Review and Network meta-analyses based on a Bayesian framework

Cong Li[1,2,3], Zhiwei Gao[1,4], Junwei Zhang[1], Shaolei Ma[1], Ming Mo[1], Changde Wu[1], Jianfeng Xie[1], Ling Liu[1], Yingzi Huang[1], Haibo Qiu[1], Yi Yang[1]*

*Joint corresponding authors

Author Affiliations:

[1]Department of Critical Care Medicine, Zhongda Hospital, School of Medicine, Southeast University, Nanjing 210009, China

[2]Emergency Medicine Department of the Affiliated Hospital of Xuzhou Medical University, Xuzhou, Jiangsu province, 221002, China

[3]Jiangsu Provincial Institute of Health Emergency, Xuzhou Medical University, Xuzhou, Jiangsu province, 221002, China

[4]Department of Emergency, The affiliated Huaian NO.1 People's Hospital of Nanjing Medical University, Huai'an, 223300, China

Correspondence to: Prof Yi Yang, Department of Critical Care Medicine, Zhongda Hospital, School of Medicine, Southeast University, Nanjing 210009, China. E-mail: yiyiyang2004@163.com.

Online Appendix Content

Appendix 1 8

Figure S 1.1 Flow diagram of studies 8

Appendix 2 9

Table S 2.1 Description of intervention in the included studies 9

Table S 2.1 Description of intervention in the included studies (Continued) 10

Table S 2.1 Description of intervention in the included studies (Continued) 11

Table S 2.1 Description of intervention in the included studies (Continued) 12

Table S 2.1 Description of intervention in the included studies (Continued) 13

Table S 2.1 Description of intervention in the included studies (Continued) 14

Table S 2.1 Description of intervention in the included studies (Continued) 15

Table S 2.1 Description of intervention in the included studies (Continued) 16

Table S 2.2 Description of antibiotic interventions in the included studies 17

Table S 2.2 Description of antibiotic interventions in the included studies (Continued) 18

Table S 2.3 Description of outcomes in studies included in network analyses 19

Table S 2.3 Description of outcomes in studies included in network analyses (Continued) 20

Table S 2.3 Description of outcomes in studies included in network analyses (Continued) 21

Table S 2.3 Description of outcomes in studies included in network analyses (Continued) 22

Appendix 3 23

Table S 3.1 General characteristics of interventions 23

Appendix 4 24

Figure S 4.1 Risk of bias summary graph: review authors' judgements (low, unclear and high) for each risk of bias item shown as percentages across all included studies. 24

Figure S 4.2 Risk of bias summary: judgements about each bias item for each study 25

Appendix 5 26

Table S 5.1 Pairwise and network estimated odds ratios (with 95% CrI) of interventions on nosocomial infection 27

Figure S 5.1 Forest plot of the pairwise and network effect estimate on nosocomial infection 27

Table S 5.2 Pairwise and network estimated odds ratios (with 95% CrI) of interventions on hospital acquired pneumonia 28

Figure S 5.2 Forest plot of the pairwise and network effect estimate on hospital acquired pneumonia 28

Table S 5.3 Pairwise and network estimated odds ratios (with 95% CrI) of interventions on ventilator-associated pneumonia 29

Figure S 5.3 Forest plot of the pairwise and network effect estimate on ventilator-associated pneumonia 29
Table S 5.4 Pairwise and network estimated odds ratios (with 95% CrI) of interventions on bloodstream infection 30
Figure S 5.4 Forest plot of the pairwise and network effect estimate on bloodstream infection ... 30
Table S 5.5 Pairwise and network estimated odds ratios (with 95% CrI) of interventions on catheter-related bloodstream infection 31
Figure S 5.5 Forest plot of the pairwise and network effect estimate on catheter-related bloodstream infection 31
Table S 5.6 Pairwise and network estimated odds ratios (with 95% CrI) of interventions on urinary tract infections 32
Figure S 5.6 Forest plot of the pairwise and network effect estimate on urinary tract infections .. 32
Table S 5.7 Pairwise and network estimated odds ratios (with 95% CrI) of interventions on sepsis 33
Figure S 5.7 Forest plot of the pairwise and network effect estimate on sepsis 33
Table S 5.8 Pairwise and network estimated odds ratios (with 95% CrI) of interventions on diarrhea 34
Figure S 5.8 Forest plot of the pairwise and network effect estimate on diarrhea 34
Table S 5.9 Pairwise and network estimated odds ratios (with 95% CrI) of interventions on hospital mortality 35
Figure S 5.9 Forest plot of the pairwise and network effect estimate on hospital mortality 35
Table S 5.10 Pairwise and network estimated odds ratios (with 95% CrI) of interventions on ICU mortality 36
Figure S 5.10 Forest plot of the pairwise and network effect estimate on ICU mortality 36
Table S 5.11 Pairwise and network estimated mean differecen (with 95% CrI) of interventions on hospital lengtn of stay 37
Figure S 5.11 Forest plot of the pairwise and network effect estimate on v hospital lengtn of stay 37
Table S 5.12 Pairwise and network estimated mean differecen (with 95% CrI) of interventions on ICU length of stay 38
Figure S 5.12 Forest plot of the pairwise and network effect estimate on ICU length of stay 38
Table S 5.13 Pairwise and network estimated mean differecen (with 95% CrI) of interventions on the duration of mechanical ventilation 39
Figure S 5.13 Forest plot of the pairwise and network effect estimate on the duration of mechanical ventilation 39
Appendix file 6 40
Figure S 6.1 Network plot of all intervention comparisons for nosocomial infection 40

Figure S 6.2 Network plot of all intervention comparisons for hospital acquired pneumonia 41
Figure S 6.3 Network plot of all intervention comparisons for ventilator-associated pneumonia .. 41
Figure S 6.4 Network plot of all intervention comparisons for bloodstream infections 42
Figure S 6.5 Network plot of all intervention comparisons for catheter-related bloodstream infection 42
Figure S 6.6 Network plot of all intervention comparisons for urinary tract infections 43
Figure S 6.7 Network plot of all intervention comparisons for sepsis 43
Figure S 6.8 Network plot of all intervention comparisons for diarrhea 44
Figure S 6.9 Network plot of all intervention comparisons for hospital mortality 44
Figure S 6.10 Network plot of all intervention comparisons for ICU mortality 45
Figure S 6.11 Network plot of all intervention comparisons for hospital lengtn of stay 45
Figure S 6.12 Network plot of all intervention comparisons for ICU length of stay 46
Figure S 6.13 Network plot of all intervention comparisons for the duration of mechanical ventilation 46
Appendix file 7 47
Figure S 7.1 Assesment of heterogeneity in networks for nosocomial infection 48
Figure S 7.2 Assesment of heterogeneity in networks for hospital acquired pneumonia 49
Figure S 7.3 Assesment of heterogeneity in networks for ventilator-associated pneumonia 50
Figure S 7.4 Assesment of heterogeneity in networks for bloodstream infection 51
Figure S 7.5 Assesment of heterogeneity in networks for catheter-related bloodstream infection 52
Figure S 7.6 Assesment of heterogeneity in networks for urinary tract infection 53
Figure S 7.7 Assesment of heterogeneity in networks for sepsis 54
Figure S 7.8 Assesment of heterogeneity in networks for diarrhea 55
Figure S 7.9 Assesment of heterogeneity in networks for hospital mortality 56
Figure S 7.10 Assesment of heterogeneity in networks for ICU mortality 57
Figure S 7.11 Assesment of heterogeneity in networks for hospital lengtn of stay 58
Figure S 7.12 Assesment of heterogeneity in networks for ICU length of stay 59
Figure S 7.13 Assesment of heterogeneity in networks for the duration of mechanical ventilation 60
Table S 7.14 Assessment of global heterogeneity in networks 61
Appendix file 8 62
Figure S 8.1 Node-splitting analysis of inconsistency in networks for nosocomial infection 63
Figure S 8.2 Node-splitting analysis of inconsistency in networks for hospital acquired pneumonia 64

Figure S 8.3 Node-splitting analysis of inconsistency in networks for ventilator-associated pneumonia 64
Figure S 8.4 Node-splitting analysis of inconsistency in networks for bloodstream infection 64
Figure S 8.5 Node-splitting analysis of inconsistency in networks for catheter-related bloodstream infection 65
Figure S 8.6 Node-splitting analysis of inconsistency in networks for urinary tract infection 65
Figure S 8.7 Node-splitting analysis of inconsistency in networks for sepsis 65
Figure S 8.8 Node-splitting analysis of inconsistency in networks for diarrhea 66
Figure S 8.9 Node-splitting analysis of inconsistency in networks for hospital mortality 66
Figure S 8.10 Node-splitting analysis of inconsistency in networks for ICU mortality 66
Figure S 8.11 Node-splitting analysis of inconsistency in networks for hospital lengtn of stay 67
Figure S 8.12 Node-splitting analysis of inconsistency in networks for ICU length of stay 67
Figure S 8.13 Node-splitting analysis of inconsistency in networks for the duration of mechanical ventilation 68
Figure S 8.14 Assesment of global inconsistency in networks using the 'design-by-treatment' interaction model 69
Appendix 9 70
Figure S 9.1 Transitivity in the network of primary outcome 70
Appendix file 10 71
Figure S 10.1 Comparison-adjusted funnel plot for the network of nosocomial infection in all comparisons 71
Figure S10.2 Comparison-adjusted funnel plot for the network of hospital acquired pneumonia in all comparisons 71
Figure S 10.3 Comparison-adjusted funnel plot for the network of ventilator-associated pneumonia in all comparisons 72
Figure S 10.4 Comparison-adjusted funnel plot for the network of bloodstream infections in all comparisons 72
Figure S 10.5 Comparison-adjusted funnel plot for the network of catheter-related bloodstream infection in all comparisons 73
Figure S 10.6 Comparison-adjusted funnel plot for the network of urinary tract infections in all comparisons 73
Figure S 10.7 Comparison-adjusted funnel plot for the network of sepsis comparisons 74
Figure S 10.8 Comparison-adjusted funnel plot for the network of diarrhea comparisons 74
Figure S 10.9 Comparison-adjusted funnel plot for the network of hospital mortality in all comparisons 75
Figure S 10.10 Comparison-adjusted funnel plot for the network of ICU mortality in all comparisons 75

Figure S 10.12 Comparison-adjusted funnel plot for the network of ICU length of stay in all comparisons........ 76
Figure S 10.13 Comparison-adjusted funnel plot for the network of the duration of mechanical ventilation in all comparisons........ 77
Appendix 11........ 78
Figure S 11.1 Contribution plot for NI and contribution of low or moderate RoB comparisons to each network estimate of NI........ 79
Table S 11.1 Result of GRADE for NI........ 80
Figure S 11.2 Contribution plot for pneumonia and contribution of low or moderate RoB comparisons to each network estimate of hospital acquired pneumonia........ 81
Table S 11.2 Result of GRADE for hospital acquired pneumonia........ 82
Figure S 11.3 Contribution plot for VAP and contribution of low or moderate RoB comparisons to each network estimate of VAP........ 83
Table S 11.3 Result of GRADE for VAP........ 84
Figure S 11.4 Contribution plot for bloodstream infection and contribution of low or moderate RoB comparisons to each network estimate of bloodstream infection........ 85
Table S 11.4 Result of GRADE for bloodstream infection........ 86
Figure S 11.5 Contribution plot for CRB and contribution of low or moderate RoB comparisons to each network estimate of CRB........ 87
Table S 11.5 Result of GRADE for CRBIS........ 88
Figure S 11.6 Contribution plot for UTI and contribution of low or moderate RoB comparisons to each network estimate of UTI........ 89
Table S 11.6 Result of GRADE for UTI........ 90
Figure S 11.7 Contribution plot for sepsis and contribution of low or moderate RoB comparisons to each network estimate of sepsis........ 91
Table S 11.7 Result of GRADE for sepsis........ 92
Figure S 11.8 Contribution plot for diarrhea and contribution of low or moderate RoB comparisons to each network estimate of diarrhea........ 93
Table S 11.8 Result of GRADE for diarrhea........ 94
Figure S 11.9 Contribution plot for hospital mortality and contribution of low or moderate RoB comparisons to each network estimate of hospital mortality........ 95
Table S 11.9 Result of GRADE for hospital mortality........ 96
Figure S 11.10 Contribution plot for ICU mortality and of low or moderate RoB comparisons to each network estimate of ICU mortality........ 97
Table S 11.10 Result of GRADE for ICU mortality........ 98
Figure S 11.11 Contribution plot for hospital LOS and of low or moderate RoB comparisons to each network estimate of hospital LOS........ 99

Table S 11.11 Result of GRADE for hospital LOS 100
Figure S 11.12 Contribution plot for ICU LOS and of low or moderate RoB comparisons to each network estimate of ICU LOS 101
Table S 11.12 Result of GRADE for ICU LOS............ 102
Figure S 11.13 Contribution plot for MV and of low or moderate RoB comparisons to each network estimate of MV 103
Table S 11.13 Result of GRADE for MV............ 104
Appendix file 12 105
Figure S 12.1 Treatment ranking and SUCRA ranking curve for nosocomial infection 105
Figure S 12.2 Treatment ranking and SUCRA ranking curve for hospital acquired pneumonia..... 106
Figure S 12.3 Treatment ranking and SUCRA ranking curve for ventilator-associated pneumonia107
Figure S 12.4 Treatment ranking and SUCRA ranking curve for bloodstream infection 108
Figure S 12.5 Treatment ranking and SUCRA ranking curve for catheter-related bloodstream infection 109
Figure S 12.6 Treatment ranking and SUCRA ranking curve for urinary tract infections 110
Figure S 12.7 Treatment ranking and SUCRA ranking curve for sepsis............ 111
Figure S 12.8 Treatment ranking and SUCRA ranking curve for diarrhea............ 112
Figure S 12.9 Treatment ranking and SUCRA ranking curve for hospital mortality 113
Figure S 12.10 Treatment ranking and SUCRA ranking curve for ICU mortality 114
Figure S 12.11 Treatment ranking and SUCRA ranking curve for hospital lengtn of stay 115
Figure S 12.12 Treatment ranking and SUCRA ranking curve for ICU length of stay 116
Figure S 12.13 Treatment ranking and SUCRA ranking curve for the duration of mechanical ventilation 117
Appendix 13 118
Table S 13.1 Subgroup analyses for nosocomial infection in different populations............ 118
Table S 13.2 Subgroup analyses for nosocomial infection in different times, quality and doses错误!未定义书签。
Appendix 14 119
Table S 14.1 Sensitivity analyses for the risk of nosocomial infection 119
Appendix 15 120
References of included studies 120

Appendix 1

Flow diagram of studies

Figure S 1.1 Flow diagram of studies

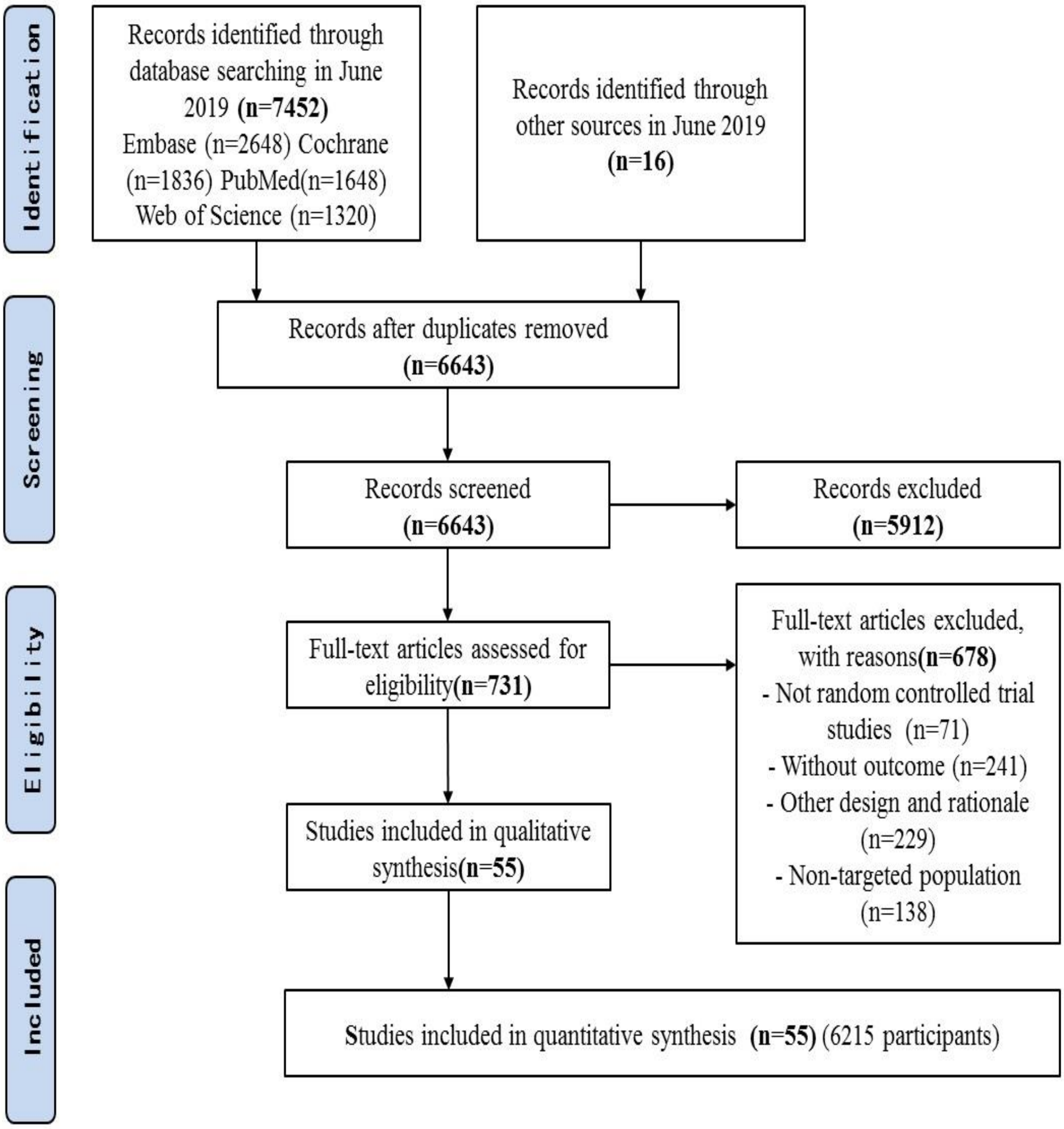

Appendix 2
Description of included studies, interventions, and outcomes

Table S 2.1 Description of intervention in the included studies

ID	Author	Intervention	Details of intervention	Dose or volume of intervention	Duration of intervention	Duration (days), mean(SD)	Drug administration	Enteral feeding/ total parenteral feeding
1	Braga	EN	Impart®+standard formual	25 kcal/kg.day^{-1}	NR	NR	Nasogastric jejunostomies tubes	Enteral feeding
		TPN	Isonitrogenous isocaloric		NR	NR	NR	Parenteral feeding
2	Kudsk	EN	Impart®, Immun-Aid®	Mean 1400 kcal/day	Until hospital discharge	NR	Jejunostomies tubes	Enteral feeding
		TPN	NR	NR	NR	NR	NR	Parenteral feeding
3	Bleichner	Probiotics+EN	**Probiotics:** S. boulardii<br>**EN:** Intact protein standard diet without fiber or lactose	500 mg QID	21 days or until EN stopped	10.1 (6.1)	Diluted in 20 ml of tepid water (NG/jejunostomy tube)	Enteral feeding
		Placebo+EN	**Placebo:** Powder was indistinguishable from the S. boulardii powder<br>**EN:** Intact protein standard diet without fiber or lactose	500 mg QID		10.7 (6.4)	NR	
4	Falcao	Synbiotics+EN	Fermented milk(Lactobacillus johnsonii) (35 kcal ·kg^{-1} of mean ideal weight ·day^{-1})	Fermented milk 240ml QD	14 days, beginning no later than 48 h after admission and continuing for a minimum of 5 days	9 (4)	Nasoenteral tube	Enteral feeding
		EN	Standard formula	NR		13 (5)	NR	
5	Jain	Synbiotics+EN	**Probiotics(Trevis™)**:L. acidophilus La5, L. bulgaricus, Bifidobacterium lactis Bb-12, Streptococcus thermophilus<br>**Prebiotics**:oligofructose<br>**EN:** NR	Probiotic 4×10^{9}cfu TID<br>Prebiotic7.5g BID	Until hospital discharge	10	Orally/NG tube	Enteral or parenteral feeding
		Placebo+EN	Placebo: Sucrose powder<br>**EN:** NR	Powdered sucrose capsules TID		10		
6	Lu	Synbiotics+EN	**Probiotics**:Pediococcus pentosaceus, Leuconostoc mesenteroides, Lactobacillus paracasei subsp paracasei, Lactobacillus plantarum<br>**Prebiotics**:Betaglucan, Inulin, Pectin, Resistant starch<br>**EN:** Nutrison Fibre	Probiotic 4×10^{10} cfu QD<br>Prebiotic10g QD	21 days after injury	NR	Diluted in 50 ml of tepid water (NG tube)	Enteral feeding
		Prebiotics+EN	Betaglucan, Inulin, Pectin, Resistant starch<br>**EN:** Nutrison Fibre	10g QD		NR	NG tube	
7	Sun	EN	Flicare	NR	Until hospital discharge	NR	NG/jejunostomy tube	Enteral or adjuvant PN
		TPN	Harris-Benedict formula	125-146 kJ/kg		NR	Central vein	Parenteral feeding

Table S 2.1 Description of intervention in the included studies (Continued)

ID	Author	Intervention	Details of intervention	Dose or volume of intervention	Duration of intervention	Duration (days), mean(SD)	Drug administration	Enteral feeding/ total parenteral feeding
8	Klarin	Probiotics+EN	**Probiotics**:Lactobacillus plantarum 299v	Probiotics: 5×10^{10} cfu Q6h 3 days	Until ICU discharge	NR	NG tube	Enteral feeding and PN
		EN	NR	NR		NR		
9	McNaught	Probiotics+EN	**Probiotics**: Proviva (L.plantarum 299 v)	Probiotics:2.5×10^{9} cfu QD	Until discharge from hospital or the patient expressed the wish to discontinue	9 (10.4)	Orally/NG tube	Enteral or adjuvant PN
		EN	EN	NR		NR		
10	Morrow	Probiotics+EN	Lactobacillus GG	1×10^{9} cfu BID	72h	NR	Orally/NG tube/Iv Oropharynx and stomach	Enteral feeding or parenteral feeding
		Placebo+EN	Inactive plant starch inulin	BID		NR		
11	Kotzampassi	Synbiotics+EN	Synbiotic 2000 Forte<br>**Probiotics**:Pediococcus pentoseceus 5-33:3, Leuconostoc mesenteroides 32-77:1, L.paracasei ssp 19, L.plantarum 2,362<br>**Prebiotics**:inulin, oat bran,pectin, resistant starch	Probiotic 4×10^{9} cfu QD<br>Prebiotic10g QD	15 days	NR	Endoscopic gastrostomy or NG tube	Enteral feeding or parenteral feeding
		Placebo+EN	Placebo: Maltodextrin	QD		NR		
12	Petrov	EN	Peptamen	daily 30 kcal/kg and 1.5 g/kg of protein (ideal body weight,)	Minimum of 7 days	NR	Nasojejunal feeding tube, distal to the ligament of Treitz	Enteral feeding
		TPN	10% dextrose solution, 10% amino acid solution and 10% fat emulsion			NR	Central venous catheter	Parenteral feeding
13	Spindler-Vesel	Synbiotics+EN	Synbiotic 2000<br>**Probiotics**: Lactobacillus: Pediococcus pentosaceus 5–33:3, Lactococcus raffinolactis 32–77:1, Lactobacillus paracasei subsp paracasei 19, Lactobacillus plantarum 2362<br>**Prebiotics**: glucan, inulin, pectin, resistant starch	Probiotic 4×10^{10} cfu QD<br>Prebiotic10g QD	7 consecutive days. Until ICU discharge or EN discontinuation	NR	NG tube	Enteral feeding
		Prebiotics+EN	Nova Source: fermentable fibers	2.2g per 100 mL		NR		
		EN	**Nutricomp peptide**<br>**Alitraq**:glutamine, arginine, α-linolenic acid	1.55 g glutamine, 446 mg arginine, 154 mg α-linolenic acid per 100 mL.		NR		
14	Abdulmeguid	EN	NR	NR	Maximum of 72 hours	NR	Naso-jejunal tube, close to Treitz's ligament.	Enteral feeding
		TPN	Identical amounts of fat, carbohydrate, and protein.	NR		NR	central venous catheter (subclavian/jugular)	Parenteral feeding
15	Alberda	Probiotics+EN	**VSL#3**: Lactobacillus ,Bifidobacterium, Streptococcus salivarius subsp. Thermophilus	Probiotics: 4.5×10^{11} cfu BID<br>EN: 25–30 kcal/kg, 1.2–1.5 g/kg protein.	NR	8 (4)	NJ tube	Enteral feeding and adjuvant peripheral parenteral feeding
		EN	**Jevity Plus**,	25–30 kcal/kg, 1.2–1.5 g/kg protein.	NR	10 (4)		

Table S 2.1 Description of intervention in the included studies (Continued)

ID	Author	Intervention	Details of intervention	Dose or volume of intervention	Duration of intervention	Duration (days), mean(SD)	Drug administration	Enteral feeding/ total parenteral feeding
16	Casas	EN	PEPTISORB®,	1.5-2 g proteins/kg/day and 30-35 kcal/kg/day.	7 day minimum	NR	NJ tube	Enteral feeding
		TPN	NR	1.5-2 g proteins/kg/day and 30-35 kcal/kg/day.		NR		
17	Karakan	Prebiotics+EN	**Prebiotic:**soluble fibers and insoluble fibers	24 g per day	7 days	NR	Dissolved in 100 mL of lukewarm sterile water, intragastric tube feeding	Enteral feeding
		EN	**EN:**No prebiotics, no placebo	2000kcal/d		NR	Intragastric tube feeding	
18	Olah	Synbiotics+EN	Synbiotic 2000 Forte **Probiotics**:Pediococcus pentoseceus 5-33:3, Leuconostoc mesenteroides 32-77:1, L.paracasei ssp 19, L.plantarum 2,362 **Prebiotics**:inulin, oat bran,pectin, resistant starch	Probiotic 4×10^{10} cfu QD Prebiotic10g QD	NR	NR	NR	NR
		Prebiotics+EN	Plant fibers (Betaglucan, inulin, pectin,resistant starch)	10g QD				
19	Sramek V	Synbiotics+EN	Synbiotic 2000 Forte **Probiotics**:Pediococcus pentoseceus 5-33:3, Leuconostoc mesenteroides 32-77:1, L.paracasei ssp 19, L.plantarum 2,362 **Prebiotics**:inulin, oat bran,pectin, resistant starch, inulin, oat bran,pectin, resistant starch	Probiotic 4×10^{10} cfu QD Prebiotic10g QD	8 days	NR	NJ tube	Enteral feeding
		Prebiotics+EN	Tea	NR		NR		
20	Besselink	Probiotic+EN	**Probiotic** (Ecologic 641): six different strains of freeze-dried, viable bacteria: Lactobacillus acidophilus, Lactobacillus casei, Lactobacillus salivarius, Lactococcus lactis, Bifidobacterium bifidum, Bifidobacterium lactis) EN: Nutrison Multi Fibre	Probiotic 10^{10} cfu totally daily	For a maximum of 28 days.	NR	Dissolved in sterilised distilled water and administered NJ tube/Oral	Enteral feeding
		EN	Nutrison Multi Fibre	NR		NR		
21	Forestier	Probiotics+EN	Lactobacillus casei rhamnosus	10^{9} cfu BID	From the third day after admission to the ICU until discharge or death.	NR	NG tube or oral after tube removal	Enteral feeding
		Placebo+EN	Growth medium without bacteria	NR		NR		
22	Klarin	Synbiotics+EN	**Probiotics**, 299 Lactobacillus plantarum 8×10^{8} cfu /ml **prebiotics**,oatmeal	Probiotics: given as 6 × 100 ml doses every 12 h and after 50 ml given BID	Until ICU discharge	5.5 (4.875)	NG tube	Enteral feeding
		Prebiotics+EN	Oatmeal	Same oatmeal gruel mixed with lactic acid		8.8 (16.48)		
23	Doley	EN	NR	2,500-2,700 kcal/day, 120-130g/day of protein.	72 hours of admission and was continued for a minimum of 14 days.	NR	Enteral tube	Enteral feeding
		TPN	NR	2,500-2,700 kcal/day, 120-130g/day of protein.		NR	Central venous catheter	Parenteral feeding

Table S 2.1 Description of intervention in the included studies (Continued)

ID	Author	Intervention	Details of intervention	Dose or volume of intervention	Duration of intervention	Duration (days), mean(SD)	Drug administration	Enteral feeding/ total parenteral feeding
24	Giamarellos-Bourboulis	Synbiotics+EN	Synbiotic 2000 Forte<br>**Probiotics**:Pediococcus pentoseceus 5-33:3, Leuconostoc mesenteroides 32-77:1, L.paracasei ssp 19, L.plantarum 2,362<br>**Prebiotics**:inulin, oat bran,pectin, resistant starch<br>EN: Intestamin	Probiotic: 4×10^{10} cfu QD<br>Prebiotic:10g QD	15 days	NR	Diluted in 100 mL of tap water<br>NG/gastrostomy tube	Enteral feeding
		EN	Intestamin	NR		NR	NG/gastrostomy tube	
25	Knight	Synbiotics+EN	Synbiotic 2000 Forte<br>**Probiotics**:Pediococcus pentoseceus 5-33:3, Leuconostoc mesenteroides 32-77:1, L.paracasei ssp 19, L.plantarum 2,362<br>**Prebiotics**:inulin, oat bran,pectin, resistant starch<br>EN: Nutrison Energy	Probiotic 4×10^{10} cfu BID<br>Prebiotic10g BID	A maximum of 28 days or ICU discharge or death	NR	Dissolved in 50-100 ml of sterile water<br>NG tube /OG	Enteral feeding
		Placebo+EN	Placebo: Crystalline cellulose<br>EN: Nutrison Energy	10g BID		NR	NG tube /OG	
26	Moses	EN	Hypocaloric EN	Maximum of 1000 cal/d and protein 28.32g	From the time of intubation to either the time of tracheostomy or extubation or transfer out of the medical ICU to the ward or death.	NR	Nasogastric feeds	Enteral feeding
		TPN	Glucose and electrolyte	Maximum of 1000 cal/d and protein 28.32g		NR	Central venous catheter	Parenteral feeding
27	Barraud	Probiotics+EN	**Probiotics:** Ergyphilus<br>Lactobacillus rhamnosus GG, Lactobacillus casei, Lactobacillus acidophilus, Bifidobacterium bifidum<br>**EN:** Fresubin®	Probiotics: 2 ×10^{10} cfu QD<br>EN: 30–35 kcal/kg	The entire period of mechanical ventilation but for a duration not exceeding 28 days	NR	Diluted in 20 mL of water<br>NG tube	Enteral feeding
		Placebo+EN	**Placebo:**Excipient<br>**EN:** Fresubin®	Placebo:NR<br>EN: 30–35 kcal/kg		NR	NG tube	
28	Frohmader	Probiotics+EN	**Probiotics(VSL#3):**Lactobacillus ,Bifidobacteri um, Streptococcus salivarius subsp. Thermophilus<br>**EN:** Isosource or Renal or Diabetic Resource (Novartis, Melbourne, Australia)	Probiotics: 4.5×10^{11} cfu BID<br>EN: 25 to 35 cal/kg per day and 0.8 to 1.5 g protein/ kilogram/day.	until hospital discharge	NR	NG/NJ tube	Enteral feeding
		Placebo+EN	**Placebo:** Free of fiber and prebiotic additives<br>**EN:** Isosource or Renal or Diabetic Resource (Novartis, Melbourne, Australia)	Placebo:BID<br>EN: 25 to 35 cal/kg per day and 0.8 to 1.5 g protein/ kilogram/day.		NR		
29	Morrow	Probiotics+EN	Probiotics: Lactobacillus rhamnosus GG<br>EN:NR	Probiotics: 2 ×10^{9} cfu BID	Until extubation, tracheostomy placement, or death	NR	Mixed with water<br>Oropharynx and NG tube	Enteral feeding
		Prebiotics+EN	Prebiotics : Inulin<br>EN:NR	BID		NR		

Table S 2.1 Description of intervention in the included studies (Continued)

ID	Author	Intervention	Details of intervention	Dose or volume of intervention	Duration of intervention	Duration (days), mean(SD)	Drug administration	Enteral feeding/ total parenteral feeding
30	Ferrie	Synbiotics+EN	**Probiotics**: Lactobacillus rhamnosus GG<br>**Prebiotics**: inulin powder<br>EN:standard feeding formula,which is a 1-calorie per mL oat fiber–containing formula	Probiotic: 10^{10} cfu QD<br>Prebiotic:280 mg QD	7 days	NR	Dispersed in 50 mL sterile water NG tube	Enteral feeding
		Prebiotics+EN	**Prebiotics**: Inulin powder<br>EN:standard feeding formula,which is a 1-calorie per mL oat fiber–containing formula	Prebiotic:280 mg QD		NR	NG tube	
31	Tan	Probiotics+EN	**Probiotics:** Golden Bifid: 0.5 $\times 10^8$ cfu Bifidobacterium longum, 0.5 $\times 10^7$ cfu Lactobacillus bulgaricus,0.5 $\times 10^7$ cfu Streptococcus thermophilus<br>**EN:** (3.8 g protein, 13.8 g carbohydrate, 3.4 g fat/100 ml, osmolarity 250 mOsm/l, no fibers)	Probiotics:10^9 cfu per day<br>EN: 30 kcal/kg body weight/day	21 days	NR	NG tube	Enteral feeding and adjuvant peripheral parenteral feeding
		EN	**EN:** (3.8 g protein, 13.8 g carbohydrate, 3.4 g fat/100 ml, osmolarity 250 mOsm/l, no fibers)	30 kcal/kg body weight/day		NR		
32	Hayakawa	Synbiotics+EN	**Probiotics**: 1×10^8 cfu /g Bifidobacterium breve strain Yakult, 1×10^8 cfu /g Lactobacillus casei strain Shirota<br>**Prebiotics**: galactooligosaccharides<br>**EN**：Medief(100 kcal, protein 4.5 g, fat 2.8 g, carbohydrate 14.2 g, dietary fiber 1.2 g in 100 ml)(Ajinomoto®)	Probiotics: 1g TID<br>Prebiotics: 5g TID<br>EN: According to the patient’s requirements	8 weeks	NR	Gastrostomy/Jejunostomy/ NG tube	Enteral feeding
		EN	Medief(100 kcal, protein 4.5 g, fat 2.8 g, carbohydrate 14.2 g, dietary fiber 1.2 g in 100 ml)(Ajinomoto®)	According to the patient’s requirements		NR		
33	Malian	Probiotics+EN	**Probiotics:** Lactobacillus GG<br>**EN:** NR	NR	Until hospital discharge	NR	Orally/feeding tube	Enteral feeding
		Placebo+EN	**Placebo:**NR<br>**EN:** NR	NR		NR		
34	Plaudis	Synbiotics+EN	Synbiotic 2000 Forte<br>**Probiotics**:Pediococcus pentoseceus 5-33:3, Leuconostoc mesenteroides 32-77:1, L.paracasei ssp 19, L.plantarum 2,362<br>**Prebiotics**:inulin, oat bran,pectin, resistant starch<br>EN: Nutrison, standard whole protein feeding formula	Probiotic 4×10^9 cfu BID<br>Prebiotic10g BID<br>EN 2500 kcal/day	NR	8(1.8)	Small intestine feeding tube.	Enteral feeding
		Prebiotics+EN	**Prebiotics**:inulin, oat bran,pectin, resistant starch<br>EN: Nutrison, standard whole protein feeding formula	Prebiotic10g BID<br>EN 2500 kcal/day		7.6(1.5)		
		EN	Nutrison, standard whole protein feeding formula	2500 kcal/day		9.4(12.5)		
35	Cui	Probiotics+EN	**Protiotics:**Bifidobacterium<br>**EN:** Peptisorb,Nutrison Fibre	Protiotics:10.416 $\times10^9$ cfu Q12h,<br>EN: NR	14 days	NR	NG tube	Enteral feeding

Table S 2.1 Description of intervention in the included studies (Continued)

ID	Author	Intervention	Details of intervention	Dose or volume of intervention	Duration of intervention	Duration (days), mean(SD)	Drug administration	Enteral feeding/ total parenteral feeding
		EN	**EN:** Peptisorb,Nutrison Fibre	EN: NR		NR		
		PN	Glucose, electrolyte, fat emulsion, amino acid	EN: NR		NR	Central venous catheter	TPN feeding
36	Elke	EN	NR	NR	21 days or discharge from the ICU or death	NR	NR	Enteral feeding
		TPN	NR	NR		NR	NR	Total parenteral feeding
37	Tan	Probiotics+EN	**Protiotics:** Golden Bifid: 0.5 $\times 10^{8}$ cfu Bifidobacterium longum, 0.5 $\times 10^{7}$ cfu Lactobacillus bulgaricus,0.5 $\times 10^{7}$ cfu Streptococcus thermophilus **EN:** Standard formula	Protiotics:10^{9} cfu per day EN: NR	7 days	NR	NG tube	Enteral feeding
		EN	Standard formula	NR		NR		Enteral feeding and adjuvant PN feeding
38	Wang	Probiotics+EN	**Protiotics:** Bacillus subtilis 1.8 $\times 10^{9}$ cfu /g,Enterococcus faecium 2.0 $\times 10^{8}$ cfu /g **EN:** PEPTISORB®	Protiotics: 0.5 g TID EN: 2 g proteins/kg/d and 35 kcal/kg/d	21 days	NR	NG tube	Enteral feeding
		EN	**EN:** PEPTISORB®	EN:2 g proteins/kg/d and 35 kcal/kg/d		NR		
		TPN	TPN	2 g proteins/kg/d and 35 kcal/kg/d, A ratio of 120:1 of nonprotein calories-to-nitrogen	NR	NR	Central venous catheter (subclavian or jugular)	Total PN feeding
39	Lopez de Toro	Synbiotics+EN	**Probiotics**(Drink Simbiotic): streptococcus Thermophilus,lactobacillus bulgaricus, Lactobacilluscasei, lactobacillus acidophilus,bifidobacterium, Escherichia coli,coliformes **Prebiotics**:NR	Max 4.8 $\times 10^{9}$ cfu /ml	7 days	NR	NG tube	Enteral feeding
		EN	NR	NR		NR		
40	Sanaie	Probiotics+EN	**Probiotics**(VSL#3): Lactobacillus acidophilus, Bifidobacterium longus, Bifidobacterium bifidum &Bifidobacterium infantalis **EN:** Fresubin original fibr	Probiotics:9.0$\times 10^{9}$ cfu BID EN: Energy requirements 25-30 kcal/kg and protein 1.2-1.5 g/kg.	7 days	NR	NG tube	Enteral feeding
		EN	**EN:** Fresubin original fibr	Energy requirements 25-30 kcal/kg and protein 1.2-1.5 g/kg.		NR		
41	Zhu	Probiotics+EN	**Probiotics:** Clostridium Butyricum (miyarisan) **EN:** NR	0.7 $\times 10^{6}$ cfu BID	14 days	NR	NG tube	Enteral feeding
		Placebo+EN	**Placeebo:**Starch **EN:** NR	The same capsule type and amount		NR		

Table S 2.1 Description of intervention in the included studies (Continued)

ID	Author	Intervention	Details of intervention	Dose or volume of intervention	Duration of intervention	Duration (days), mean(SD)	Drug administration	Enteral feeding/ total parenteral feeding
42	Fu	Probiotics+EN	**Probiotics:**live combined bacillus subtilis and enterococcusfaecium<br>**EN:** Peptisorb,Nutrison Fibre	NR	14days	NR	NR	Enteral feeding
		TPN	NR	1.0-1.5 g proteins/kg/day and 25-30 kcal/kg/day		NR	NR	Parenteral feeding
43	Kim	EN	Mediwell RTH 500®	NR	Until discharge or death	NR	Nasointestinal feeding tubes	Enteral feeding
		TPN	NR	NR		NR	Central venous catheterization	Parenteral feeding
44	Rongrungruang	Probiotics+EN	**Probiotics:** Lactobacillus casei (Yakult) (Shirota strain)<br>**EN:**NR	8 $\times 10^9$ cfu for oral care after standard oral care QD. 8 $\times 10^9$ cfu enteral feeding QD	28 days or endotracheal tubes were removed	NR	Feeding tube	Enteral feeding
		EN	NR	NR		NR		
45	Fan	EN	Nutrison Fibre	105-126KJ/d	7 days	NR	Nasointestinal feeding tubes	Enteral feeding and adjuvant PN feeding
		TPN	2:1 for carbohydrates to lipids and 100:1 for calorie nitrogen ratio	105-126KJ/d		NR	Central venous catheterization	Parenteral feeding
46	Malik	Probiotics+EN	**Probiotics:** Lactobacillus acidophilus, Lactobacillus casei, Lactobacillus lactis, Bifidobacterium bifidum, Bifidobacterium longum, Bifidobacterium infantis<br>**EN:** Osmolite 1 cal (standard formula), Glucerna (glucose intolerance formula), Peptamen (semielemental formula), and Novasource Renal (electrolyte and fluid restriction).	Probiotics:3 $\times 10^9$ cfu BID<br>EN:25 kcal $kg^{-1} d^{-1}$	7 days	NR	Diluted in 5 mL of water, NG tube	Enteral feeding
		Placebo+EN	**Placebo:**Similar appearance and taste,<br>**EN:** Osmolite 1 cal (standard formula), Glucerna (glucose intolerance formula), Peptamen (semielemental formula), and Novasource Renal (electrolyte and fluid restriction).	Placebo: 3g BID<br>EN:25 kcal $kg^{-1} d^{-1}$		NR		
47	ZarinfarN	Probiotics+EN	Lactobacillus GG	TID	NR	14.2(4.7)	NG tube	Enteral feeding
		Placebo+EN	NR	TID		17.6(6.5)		
48	Zeng	Probiotics+EN	**Probiotics:** Medilac-S: Bacillus subtilis 4.5 $\times 10^9$ cfu /0.25 g and Enterococcus faecalis 0.5 $\times 10^9$ cfu /0.25 g<br>**EN:**NR	Probiotics:0.5 g TID<br>EN:NR	Until tracheal extubation, discharge from the hospital or death, with a maximum duration of 14 days	14 (5.185)	NG tube	Enteral feeding
		EN	NR	NR		12 (0.741)		
49	Alberda	Probiotics+EN	**Probiotics:** Lactobacillus casei (Danactive)	1 $\times 10^{10}$ cfu BID	7 days	10.31 (4.2)	Feeding tube/Oral	Enteral feeding and adjuvant PN feeding
		EN	No prebiotics, no placebo	NR		11 (6.64)		

Table S 2.1 Description of intervention in the included studies (Continued)

ID	Author	Intervention	Details of intervention	Dose or volume of intervention	Duration of intervention	Duration (days), mean(SD)	Drug administration	Enteral feeding/ total parenteral feeding
50	Fazilaty	Prebiotics+EN	**Prebiotics:**b-glucan<br>**EN:**high-protein enteral diet(20% protein, 30% lipid, and 50% carbohydrate)	3g QD<br>25-30 kcal/kg	NR	NR	Feeding warm soup, NG tube	Enteral feeding
		Placebo + EN	**Placebo:** maltodextrin<br>**EN:**high-protein enteral diet(20% protein, 30% lipid, and 50% carbohydrate)	3g QD<br>25-30 kcal/kg		NR		
51	Kooshk	Prebiotics+ EN	**Prebiotics:** Fenugreek seed powder<br>**EN:**NR	3g BID	NR	NR	Feeding warm soup, NG tube	Enteral feeding
		EN	NR	NR		NR		
52	Reiginer	EN	Isosmotic,isocaloric, normal-protein, polymeric preparations	20–25 kcal/kg during the first 7 days then 25–30 kcal/kg from day 8 to extubation.	28 days	20 (19.26)	NG tube/Oral	Enteral feeding EN (Glucerna),
		TPN	Three groups of macronutrients	20–25 kcal/kg during the first 7 days then 25–30 during the first 7 days then 25–30 kcal/kg from day 8 to extubation.		NR	Central venous catheterization	Total peripheral parenteral feeding
53	Shimizu	Synbiotics+EN	**Probiotics** (Yakult BL Seichoyaku): 1 $\times 10^8$ cfu /g B. breve strain /g and 1 $\times 10^8$ cfu /g L. casei strain Shirota<br>**Prebiotics**: galactooligosaccharides(Oligomate S-HP)<br>**EN:** standard polymeric diet Glucerna®-Ex 1 kcal/mL; 51:17:32 ratio of carbohydrate, protein, and fat; 370 mOsm/L; fiber 1.4 g/100 mL	Probiotics: 3g QD<br>Prebiotics: 10g QD<br>EN: 25-30 kcal/kg ideal body weight per day as the calorie goal.	7 days	6(3.7)	NG tube	Enteral feeding
		EN	standard polymeric diet Glucerna®-Ex 1 kcal/mL; 51:17:32 ratio of carbohydrate, protein, and fat; 370 mOsm/L; fiber 1.4 g/100 mL	25-30 kcal/kg ideal body weight per day as the calorie goal.		4(2.2)		
54	Tuncay	Prebiotics+EN	**Prebiotics:** Fructo-oligosaccharides (Jevity, 1 kcal/1 ml)<br>**EN:** Standard formula (Osmolite, 1 kcal/1 ml)	Prebiotics:5.3g QD 1 g/kg/ day<br>EN:30-40 ml/kg/day	21 days	NR	NG/ NJ tube	Enteral feeding
		EN	Standard formula (Osmolite, 1 kcal/1 ml)	1 g/kg/ day and 30-40 ml/kg/day		NR		
55	Mahmoodpoor	Probiotics+EN	**Probiotics:** Lactocare: Lactobacillus species (casei, acidophilus, rhamnosus, bulgaricus), Bifidobacterium species (breve, longum), Streptococcus thermophilus.<br>**EN:** Standard formula (1 kcal/mL;Ensure)	Probiotics:10^{10} cf u BID<br>EN:25 kcal/kg	14 days	NR	Feeding tube	Enteral feeding
		Placebo+EN	**Placebo:** Sterile maize starch powder<br>**EN:** Standard formula (1 kcal/mL;Ensure)	Placebo: BID<br>EN:25 kcal/kg		NR		

Abbreviation: CFU: colony forming units; EN: enteral nutrition; NG: nasogastric; NJ: Nasojejunal; NR: not reported; OG: orogastric; PN: parenteral nutrition: TPN: total parenteral nutrition.

Table S 2.2 Description of antibiotic interventions in the included studies

ID	Author	Antibiotic intervention	Duration of intervention (days), mean(SD)
1	Braga	1 g Ceftriaxone, TID, iv	Perioperative
2	Kudsk	NR	No more than 24 hours, unless dictated by specialty services
3	Bleichner	Antibiotics	NR
4	Falcao	NR	NR
5	Jain	Antibiotics	NR
6	Lu	Cefperazone-Sulbactam, flomoxef sodium,imipenem-cilastatin	NR
7	Sun	NR	NR
8	Klarin	Erythromycin,Imipenem, Metronidazol, Cefuroxime, Meropenem, Clindamycin, Vancomycin, Ciprofloxacin, Isoniazid, Rifampicin, Penicillin G	NR
9	McNaught	Broad spectrum antibiotics	NR
10	Morrow	NR	NR
11	Kotzampassi	Antibiotics	NR
12	Petrov	Antibiotic prophylaxis (ofloxacin plus metronidazole),	NR
13	Spindler-Vesel	No preventive antibiotic treatment was given.	NR
14	Abdulmeguid	NR	NR
15	Alberda	Antibiotics	NR
16	Casas	Imipenem, piperacillin-tazobactam.	NR
17	Karakan	NR	NR
18	Olah	Antibiotics were administered only in cases of suspected or manifest infection or in severe complications. Imipenem cilastatin (Tienam,MSD)	NR
19	Sramek V	NR	NR
20	Besselink	Antibiotics were used in about half the patients. The overall rate of antibiotic use in study group was no different from in the placebo group.	NR
21	Forestier	Ceftazidime, Imipenem, Ciprofloxacin, Fluconazole	NR
22	Klarin	Cephalosporines, Carbapenems	NR
23	Doley	ciprofloxacin/metronidazole or imipenem/cilastatin	NR
24	Giamarellos-Bourboulis	NR	NR
25	Knight	Cephalosporins,Penicillins,Carbapenems,Macrolides,Gentamicin,Metronidazole,Vancomycin	1~7
26	Moses	Antibiotics	NR
27	Barraud	Antibiotics	NR
28	Frohmader	ß-lactams,Penicillins, Cephalosporins, Clindamycin, Macrolides, Ciprofloxacin, Tetracyclines, Cotrimoxazole, Gentamicin, Vancomycin, Rifampicin, Metronidazole, Antifungals, Antivirals	11.1(4.9)<br>9.4 (5.8)
29	Morrow	Antibiotic therapy as deemed necessary, under the direction of their admitting physicians throughout the study.	13.3 (10.4)<br>16.3 (14.4)
30	Ferrie	NR	NR
31	Tan	Cefmetazole, Imipenem, Vancomycin	11.9 (4.9)<br>14.1 (6.0)
32	Hayakawa	Antibiotics	NR
33	Malian	NR	NR
34	Plaudis	Fluorochinolones and metronidozole or imipenem/cilastin monotherapy.	NR
35	Cui	Antibiotics	NR
36	Elke	Antibiotics	NR
37	Tan	NR	NR
38	Wang	NR	NR

Table S 2.2 Description of antibiotic interventions in the included studies (Continued)

ID	Author	Antibiotic intervention	Duration of intervention (days), mean(SD)
39	Lopez de Toro	NR	NR
40	Sanaie	Antibiotics	NR
41	Zhu	Antibiotics	NR
42	FU	NR	NR
43	Kim	Perioperative prophylaxis consisted of intravenous cefotaxime (4 g/day) and ampicillin sulbactam (6 g/day) given 4 times/day for 2 days after surgery. If bacterial sepsis was clinically suspected, broad-spectrum antibiotics were administered empirically.	NR
44	Rongrungruang	Antibiotics	NR
45	Fan	NR	NR
46	Malik	NR	NR
47	Zarinfar N	NR	NR
48	Zeng	The physicians initiated antibiotic treatment for VAP and stopped antibiotic use according to the clinical signs—but not microbiological data.Carbapenem, Glycopeptide, Linezolid	6.8(2.0)<br>7.94 (2.9)
49	Alberda	Cephalosporins, Cefazolin, Cefuroxime, Ceftriaxone, Cefixime, Cephalexin, Penicillins, Penicillin G, Amoxicillin, Piperacillin/Tazobactam, Fluoroquinolones, Ciprofloxacin, Levofloxacin, Azithromycin, Carbapenem, Meropenem, Imipenim/cilastatin, Metronidiazole Voriconazole, Vancomycin	NR
50	Fazilaty	Antibiotics	NR
51	Kooshk	Antibiotics	NR
52	Shimizu	Antibiotics were administered under the same policy during the entire study period. Carbapenem, Ampicillin/sulbactam, Cephalosporin, Vancomycin, Quinolone, Penicillin class	13(11.85)<br>18(13.33)
53	Reiginer	Antibiotics, antiviral agents, and antifungal agents.	NR
54	Tuncay	Antibiotics	NR
55	Mahmoodpoor	Antibiotics	NR

Abbreviation: NR: not reported

Table S 2.3 Description of outcomes in studies included in network analyses

Study	Outcome
**Nosocomial infection**	
Falcao, 2004	Pneumonia, Urinary tract infection, Sepsis
Jain, 2004	Septic complication
Lu, 2004	Bloodstream infection, Catheter-related bloodstream infection
Klarin, 2005	Pneumonia, Bloodstream infection, Catheter-related bloodstream infection, Urinary tract infection
McNaught, 2005	Spetic complication(chest infections, wound infections, urinary tract infections)
Morrow, 2005	Ventilator-associated pneumonia
Kotzampassi, 2006	Bacterial pneumonia, Bacteremia, Urinary tract infection, Catheter-related sepsis,Wound
Petrov,2006	Pancreatic infection (Infected pancreatic necrosis, Pancreatic abscess)<br>Extrapancreatic infection (Pneumonia, Catheter-related bloodstream infection , Urinary tract infection)
Abdulmeguid,2007	Pneumonia, intra-abdominal abscess, empyema, line sepsis, or fasciitis with wound dehiscence
Alberda, 2007	Lactobacillus-induced sepsis
Casas, 2007	Bloodstream infection, Catheter-related bloodstream infection , Urinary tract infection, pancreatic infected necrosis, SIRS
Karakan, 2007	Sepsis,Cholangitis
Olah, 2007	Pneumonia, Urinary tract infection, Pancreas related infection
Spindler-Vesel, 2006	Pneumonia, Bloodstream infection, Catheter-related bloodstream infection, Urinary tract infection
Sramek V, 2007	Undefined
Besselink, 2008	Pneumonia, Bloodstream infection, Urosepsis, Infected necrosis, Infected ascitess
Forestier, 2008	Ventilator-associated pneumonia
Klarin, 2008	Pneumonia, Bloodstream infection, Catheter-related bloodstream infection, Urinary tract infection, Clostridium difficile
Doley, 2009	Positive culture of fine needle aspirate, blood, operative specimen and drain fluid
Evangelos, 2009	Ventilator-associated pneumonia, Bloodstream infection, Urinary tract infection, Sepsis.
Knight, 2009	Ventilator-associated pneumonia
Moses, 2009	Ventilator-associated pneumonia, Catheter-related bloodstream infection, Urinary tract infection, Sepsis.
Barraud, 2010	Ventilator-associated pneumonia, Bloodstream infection, Catheter-related bloodstream infection.
Morrow, 2010	Pneumonia, Lactobacillus bacteremia, Ventilator-associated pneumonia, Clostridium difficile diarrhea
Tan, 2011	Pneumonia,Ventilator-associated pneumonia, Bloodstream infection,Urinary tract infection, Wound, Sepsis
Hayakawa, 2012	Pneumonia
Plaudis, 2012	Primary infection – when conservative treatment failed to prevent sepsis and positive bacterial cultures were obtained during the surgical intervention or percutaneous drainage of the purulent contents ;Secondary infection (drain related infection) – contamination of the necrotic tissue and fluid collections was a consequence of the early operation or percutaneous drainage of the noninfected collections (drain related infections).
Cui, 2013	Peripancreatic infection
Elke, 2013	Pneumonia, Abdomen, Bone or soft tissue, Surgical wound, Urinary tract infection, Primary bacteremia
Lopez de Toro, 2013	Infectious disease
Wang, 2013	Pancreatic sepsis
Sanaie, 2014	Sepsis
Zhu, 2014	Pneumonia, Bloodstream infection, Urinary tract infection
Fu, 2015	Undefined
Rongrungruang, 2015	Ventilator-associated pneumonia
Zarinfar N, 2016	Ventilator-associated pneumonia
Zeng, 2016	Ventilator-associated pneumonia
Alberda, 2018	Clostridium difficile infections
Fazilaty, 2018	Ventilator-associated pneumonia, Catheter-related bloodstream infection, Urinary tract infection, Sepsis, Wound
Kooshk, 2018	Ventilator-associated pneumonia
Reiginer,2018	Ventilator-associated pneumonia, Bloodstream infection, Catheter-related bloodstream infection, Urinary tract infection, Soft-tissue infection,other infection
Shimizu, 2018	Ventilator-associated pneumonia, infection complication(bloodstream infection, enteritis), Enteritis
Mahmoodpoor, 2019	Ventilator-associated pneumonia
**Hospital acquired pneumonia**	
Kudsk, 1996	1) abnormal temperature (>101 F or <96 F); 2) leukocytosis (leukocytes > 10,000 or >10% immature forms); 3) macroscopically purulent sputum; and 4) new or changing infiltrate on chest roentgenogram; 4) quantitative cultures grew 10^5 colony-forming units/mL.
Sun, 1997	Clinical manifestations plus laboratory test results
Falcao, 2004	Respiratory infection was defined as a compatible X-ray seen by the senior radiologist, fever and yellow secretion collected from the tracheal tube with positive culture.
Klarin, 2005	Secretion collected from the tracheal tube with positive culture
Morrow, 2005	Clinical manifestations plus laboratory test results
Kotzampassi, 2006	bacterial pneumonia<br>Clinical diagnosis: new or progressive pulmonary radiological infiltrate, purulent tracheal secretions, fever (> 38.5℃), and leukocytosis or leukopenia (white blood cell count more than $12*10^9$ L^{-1} or less than 4 $*10^9$ L^{-1}. microbiologically diagnosis: the isolation of a potentially pathogenic microorganisms in bronchoalveolar lavage in concentrations of 10^5 CFU mL^{-1}) or more.
Petrov,2006	Clinical manifestations plus laboratory test results
Olah,2007	Clinical manifestations plus laboratory test results
Spindler-Vesel, 2007	Microbiological specimens were collected and nosocomial infections were recorded as recommended by the Centers for Disease Control and Prevention and consensus conference.

Table S 2.3 Description of outcomes in studies included in network analyses (Continued)

Study	Outcome
Besselink, 2008	Coughing, dyspnoea, chest film showing infiltrative abnormalities, lowered arterial blood gas with positive sputum culture. If in intensive care, a positive endotracheal culture is mandatory.
Klarin, 2008	Positive tracheal secretions culture
Tan, 2011	Clinical manifestations plus laboratory test results
Hayakawa, 2012	Clinical manifestations plus laboratory test results
Elke, 2013	Clinical manifestations plus laboratory test results
Zhu, 2014	Clinical manifestations plus laboratory test results
Kim, 2015	Clinical manifestations and causative organisms were isolated simultaneously.
Fan, 2016	Clinical manifestations plus laboratory test results
**Ventilator-associated Pneumonia**	
Morrow, 2005	Clinical manifestations plus laboratory test results
Forestier, 2008	VAP was defined according mostly to the US Centers for Disease Control and Prevention's National Healthcare Safety Network criteria. These criteria require there to be at least one positive sample (protected specimen brush or plugged telescoping catheter for bronchoalveolar minilavage [>10^3 colony-forming units (CFUs)/ml] or endotracheal aspirate with [>10^5 CFUs/ml and >25 leucocytes/high-power field]); also required is the presence of one or several new abnormal radiographical and progressive parenchymatous infiltrates and one of the following signs: purulent sputum production, fever (temperature > 38.5℃), pathogenic bacteria in blood culture without other infection source, and bronchoalveolar minilavage with more than 5% cells with intethnicityllular bacteria.
Evangelos, 2009	Ventilator-associated pneumonia (VAP) was diagnosed in patients presenting with all of the following: (a) new or persistent consolidation in lung X-ray, (b) purulent tracheobroncial secretions(Cultures of tracheobroncial secretions yielding a pathogen at a count ≥10^6 CFU/mL), and (c) clinical pulmonary infection score (CPIS) more than 6.
Knight, 2009	There were new progressive, or persistent (＞24 h) infiltration on chest radiograph plus at least two of the following: (1)Temperature ＞38.0℃, (2) Leucocytosis (WBC count＞12*10^3uL^{-1}) or leucopoenia (WBC count ＜4 9*10^3uL^{-1}), (3) Purulent tracheobronchial secretions.
Moses, 2009	The appearance of a new or progressive pulmonary infiltrate and any two of the following: (a) temperature >38.0℃ or <36.0℃, (b) WBC count >10,000 or <4,000/uL, (c) purulent tracheobronchial secretions. Quantitative endotracheal aspirates with cultures were performed in patients suspected to have VAP, with ≥ 10^5 colony-forming units taken as significant
Barraud, 2010	VAP was defined by the presence of (a) a new and persistent infiltrate on chest radiograph associated with at least one of the following: purulent tracheal secretions, temperature 38.3℃ or higher, and a leukocyte count of 10,000 uL^{-1} or higher; and (b) positive quantitative cultures of distal pulmonary secretions obtained from bronchoalveolar lavage (significant threshold more than 10^4colonyforming units/mL).
Morrow, 2010	Microbiologically confirmed VAP incidence based on quantitative bronchoalveolar lavage culture with at least 10^4cfu/ml in patients intubated for 48 hours or longer. Clinical criteria require a new and persistent infiltrate on chest radiographs with two of three supporting findings: fever (＞38.5℃ or ＜35.0℃), leukocytosis (white blood cells＞10,000/mm^3 or＜3,000/mm^3), and purulent sputum.
Tan, 2011	As pneumonia occurring more than 48 hours after endotracheal intubation, and was diagnosed by the presence of both a new or progressive radiographic infiltrate plus at least two clinical features - fever > 38.0℃, leucocytosis (white blood cells count > 12×10^9/l), leucopenia (white blood cells count < 4×10^9/l), or purulent tracheobronchial secretions -and positive semiquantitative cultures of tracheobronchial secretions
Rongrungruang, 2015	A diagnosis of VAP was made if the patient had a new, persistent, or progressive infiltrate visible on a chest radiograph in combination with at least 3 of the following 4 criteria: 1) body temperature greater than 38℃ or less than 35.5℃, 2) leukocytosis (>10,000 leukocytes/mm^3) or leukopenia (<3,000 leukocytes/mm^3), 3) purulent tracheal aspirate, and 4) a semi-quantitative culture of tracheal aspirate samples that was positive for pathogenic bacteria.
Zarinfar N, 2016	Clinical manifestations plus laboratory test results
Zeng, 2016	A clinical diagnosis of VAP was based on the presence of a new, persistent or progressive infiltrate on chest radiographs that persisted for at least 48 h combined with at least two of the following criteria: (1) a temperature of >38.0 ℃ or <35.5 ℃; (2) a blood leukocytosis count of >12 × 10^3/mm^3 or <3 × 10^3/mm^3 and/or left shift; (3)purulent tracheal aspirates. A score of 3+ or 4+ defined the presence of microbiologically confirmed VAP in the semiquantitative cultures of endotracheal aspirate.
Fazilaty, 2018	A new infiltrate on chest X-rays occurring more than 48 hours after endotracheal intubation plus two or more of the following: fever (body temperate >38.3℃), leukocytosis (white blood cell count >12×10^9/ml), leucopenia (white blood cell count <4×10^9/ml), and purulent tracheobronchial secretions
Kooshk, 2018	VAP was diagnosed based on the clinical scores, according to the American College of Chest Physicians (ACCP). Clinical scores reflect the new and persistent infiltrates on chest radiographs. The infiltrates were associated to two or three supporting factors: fever (>38.5℃ or <35.0℃), leukocytosis (white blood cells >10,000 mm^3 or <3,000 mm^3), and purulent sputum
Reiginer,2018	new and persistent or progressive lung infiltrates on the chest radiograph, combined with at least two of the following criteria: body temperature ≥38.5℃ or ≤35.5℃, peripheral leukocytosis (>10,000/mm^3) or leukopenia (<4,000/mm^3), and purulent tracheal aspirates. The diagnosis must be confirmed in each participating ICU on the basis of a positive semiquantitative bacteriological result from a distal respiratory specimen: bronchoalveolar lavage fluid (positive if there are ≥10^4 colony-forming units (cfu)/ml), protected specimen brush (positive if there are ≥10^3 cfu/ml) or tracheobronchial aspirate (positive if there are ≥10^5 cfu/ml)
Shimizu, 2018	VAP refers to pneumonia that arises more than 48–72h after endotracheal intubation.

Table S 2.3 Description of outcomes in studies included in network analyses (Continued)

Study	Outcome
Mahmoodpoor, 2019	patients with a new or persistent infiltration on chest x-ray with 2 of the following criteria of hyperthermia >38℃ or hypothermia <36℃, leukocytosis or leucopenia, or purulent sputum underwent bronchoalveolar lavage (BAL). patients were considered VAP-positive if the quantitative BAL culture had at least 104 colony-forming units/mL in patients who were mechanically ventilated for >48 hours.
**Bloodstream infection**	
Lu, 2004	Positive blood culture with clinical significance.
Klarin, 2005	Positive blood culture.
Spindler-Vesel,2007	Positive blood culture with clinical significance.
Besselink, 2008	Positive blood culture. For non-pathogens (eg, coagulase-negative staphylococci) at least two samples had to be positive.
Klarin, 2008	Positive blood culture
Doley, 2009	Positive blood culture
Evangelos, 2009	Positive blood culture with clinical significance. Primary bacteremia was defined as any case of bacteremia yielding an isolate that was not isolated from any other source and where extensive diagnostic work-out comprising lung X-ray and chest and abdominal computed tomography failed to disclose another source of infection
Tan, 2011	Positive blood culture with clinical significance.
Plaudis, 2012	Positive blood culture with clinical significance.
Elke, 2013	Positive blood culture with clinical significance.
Zhu, 2014	Positive blood culture with clinical significance.
Kim, 2015	Clinical manifestations and causative organisms were isolated simultaneously.
Fan, 2016	Positive blood culture with clinical significance.
Reiginer,2018	Positive blood culture with clinical significance.
Shimizu, 2018	Bacteremia was defined as a positive blood culture after the first 3 days.
**Catheter-related bloodstream infection**	
Lu, 2004	Catheter-related bloodstream infection (CR-BSI) was defined as a positive peripheral blood culture, regardless of the microorganism, associated with a positive catheter-tip culture growing the same microorganism as in the blood.
Kotzampassi, 2006	The patient had local signs of infection at the entry site, a temperature above 38.5℃ or below 35℃, a white blood cell count greater than $10*10^9$ L^{-1} or less than $3*10^9$ L^{-1} that resolved after catheter removal with no other infection site, the semiquantitative culture of the catheter tip showing more than 15 CFU mL^{-1}, or isolation of a pathogen from blood cultures.
Petrov,2006	Clinical manifestations plus laboratory test results
Spindler-Vesel,2007	Clinical manifestations plus laboratory test results
Klarin, 2008	Positive catheter-tip culture
Moses, 2009	Bacteremia or fungemia in a patient who had an intravascular device and both (a) at least one positive blood culture, samples being obtained from peripheral veins, (b) clinical manifestations of infection (e.g., fever, chills, and/or hypotension) and no other apparent source for blood stream infection with the exception of the intravenous catheter.
Barraud, 2010	Catheter-related bloodstream infection (CR-BSI) was defined as a positive peripheral blood culture, regardless of the microorganism, associated with a positive catheter-tip culture growing the same microorganism as in the blood.
Fazilaty, 2018	According to the Centers for Disease Control's definitions for nosocomial infections.
Reiginer,2018	Clinical manifestations plus laboratory test results
**Urinary tract infection**	
Kudsk 1996	Clinical manifestations plus laboratory test results
Klarin, 2005	Positive urine culture
Kotzampassi, 2006	The urine culture showed at least 10^5 colonies of a pathogen.
Petrov,2006	Clinical manifestations plus laboratory test results
Spindler-Vesel,2007	Clinical manifestations plus laboratory test results
Olah, 2007	Clinical manifestations plus laboratory test results
Besselink, 2008	Urosepsis—dysuria with bacteraemia on the same day, without a urinary catheter in situ.
Klarin, 2008	Positive urine culture
Evangelos, 2009	Positive urine culture (Cultures of urine samples yielding a pathogen at a concentration $\geq 10^5$ CFU/mL)with clinical significance
Moses, 2009	≥100,000 colony-forming units/mL of one or two organisms first identified after at least 48 h of stay in ICU
Barraud, 2010	Clinical manifestations plus laboratory test results
Tan, 2011	Clinical manifestations plus laboratory test results
Elke, 2013	Clinical manifestations plus laboratory test results
Zhu, 2014	Clinical manifestations plus laboratory test results
Kim, 2015	Clinical manifestations and causative organisms were isolated simultaneously.
Fazilaty, 2018	Positive urine culture
Reiginer,2018	Clinical manifestations plus laboratory test results
**Sepsis**	
Falcao, 2004	Sepsis was defined as systemic inflammatory response syndrome with bacteriological evidence of infection.
Jain, 2004	Septic complications were defined as the presence of recognised pathogens in tissue which is normally sterile, confirmed by the results of culture and supported by clinical, haematological or radiological evidence.
McNaught, 2003	Spetic complication
Kotzampassi, 2006	According to the American College of Chest Physicians Definitions and Guidelines and the American College of Chest Physicians/Society of Critical Care Medicine (ACCP/SCCM) Consensus Conference Committee.

Table S 2.3 Description of outcomes in studies included in network analyses (Continued)

Study	Outcome
Alberda, 2007	Lactobacillus-induced sepsis
Karakan, 2007	Clinical manifestations plus laboratory test results
Olah, 2007	Clinical manifestations plus laboratory test results
Besselink, 2008	Urosepsis—dysuria with bacteraemia on the same day, without a urinary catheter in situ.
Evangelos, 2009	Sepsis was defined according to the American College for Chest Physicians/Society of Critical Care Medicine criteria.
Tan, 2011	Sepsis related to Lactobacillus and bowel ischemia
Plaudis, 2012	Recurrence of SIRS, worsening of the patient's general condition, signs of recurrent organ dysfunction were considered to indicate possible infection in necrotizing SAP.
Wang, 2013	Pancreatic sepsis plus infection
Sanaie, 2014	Clinical manifestations plus laboratory test results
Fazilaty, 2018	According to a consensus panel convened by the American College of Chest Physicians and the Society of Critical Care Medicine (SCCM).
**Diarrhea**	
Bleichner, 1997	Diarrhea was defined as a daily score equal to or greater than 12. Another frequently used definition of diarrhea in the literature (i. e., three or more nonformed stools per day) has also been tested.
Sun, 2004	No accurate definition is provided
Kotzampassi, 2006	No accurate definition is provided
Petrov,2006	No accurate definition is provided
Alberda, 2007	Diarrheal episodes were measured daily by the Hart & Dobb diarrheal scale. Diarrhea was defined as a score of＞12 in a 24 h period
Besselink, 2008	No accurate definition is provided
Knight, 2009	No accurate definition is provided
Moses, 2009	Self-limiting diarrhea
Barraud, 2010	Diarrhoea episodes defined by the occurrence of at least 3 liquid stools/day.
Morrow, 2010	All patients with diarrhea (three or more loose stools per 24-h period or placement of a fecal management system for continuous liquid stool) had a C. difficile cytotoxin assay sent. Patients with diarrhea but three negative C. difficile cytotoxin assays were classified as having ''ICU-associated'' diarrhea.
Rongrungruang, 2015	No accurate definition is provided
Fan, 2016	No accurate definition is provided
Zarinfar N, 2016	Caused by clostridium difficile
Alberda, 2018	Antibiotic associated diarrhea was defined as three or more bowel movements per day or greater than 750 mL liquid stool, when diarrhea continued for greater than three consecutive days, in conjunction with antibiotic administration.
Kooshk, 2018	Clostridium difficile-associated diarrhea (three or more loose stools per 24 h period), ICU-associated diarrhea (presumablybecause of acute disorder, antibiotic administration and dietary changes).
Reiginer,2018	No accurate definition is provided
Tuncay, 2018	No accurate definition is provided
Mahmoodpoor, 2019	>3 times in a day, weight >250 g, volume >500 mL

Appendix 3
General characteristics of interventions

Table S 3.1 General characteristics of interventions

Intervention	General characteristics
Probiotics	Lactobacillus plantarum 299v, Lactobacillus acidophilus, Lactobacillus acidophilus LA-5, Lactobacillus acidophilus LA-11, Lactobacillus bulgaricus, Lactobacillus paracasei, Lactobacillus plantarum 2362, Lactobacillus plantarum,Lactobacillus plantarum CGMCC No. 1258, Lactobacillus casei, Lactobacillus salivarius, Lactococcus lactis, Lactobacillus rhamnosus, Bacillus mesentericus, Bifidobacterium lactis Bb-12, Bifidobacterium longum BL-88, Bifidobacterium breve, Bifidobacterium bifidum, Bifidobacterium infantis, Clostridium butyricum, Enterococcus faecalis T-110, Leuconostoc mesenteroides 77:1, Pediacoccus pentosaceus 5-33:3, Streptococcus thermophiles, Streptococcus faecalis, Saccharomyces boulardii 011,L. paracasei ssp 19, L. delbrueckii subsp. Bulgaricus.
Prebiotics	Bioactive fibers, inulin, pectin, resistant starch, oat fiber, beta glucan, oligofructose, galacto-oligosaccharides, maltodextrin, oatmeal.
Synbiotics	Probiotics and prebiotics combination.
Enteral nutrition	Standard enteral nutrition, Fresubin, Fresenius, Homburg, Germany; Nutrison.
Parenteral nutrition	Conventional parenteral nutrition.

Appendix 4

Risk of bias assessments

We followed the recommended approach for assessing risk of bias in studies included in Cochrane reviews. This tool addresses specific bias domains including methods for generating the random sequence, allocation concealment, blinding of participants and investigators, blinding of outcome assessment, incompleteness of outcome data and selective outcome reporting. Each item is adjudicated within each study and the results are represented in a risk of bias table. The adjudication of the risk of bias is achieved by answering pre-specified questions about the methods reported by each study in relation to the risk domain, such that the conclusion is either low risk of bias, unclear risk of bias or high risk of bias.

Figure S 4.1 Risk of bias summary graph: review authors' judgements (low, unclear and high) for each risk of bias item shown as percentages across all included studies.

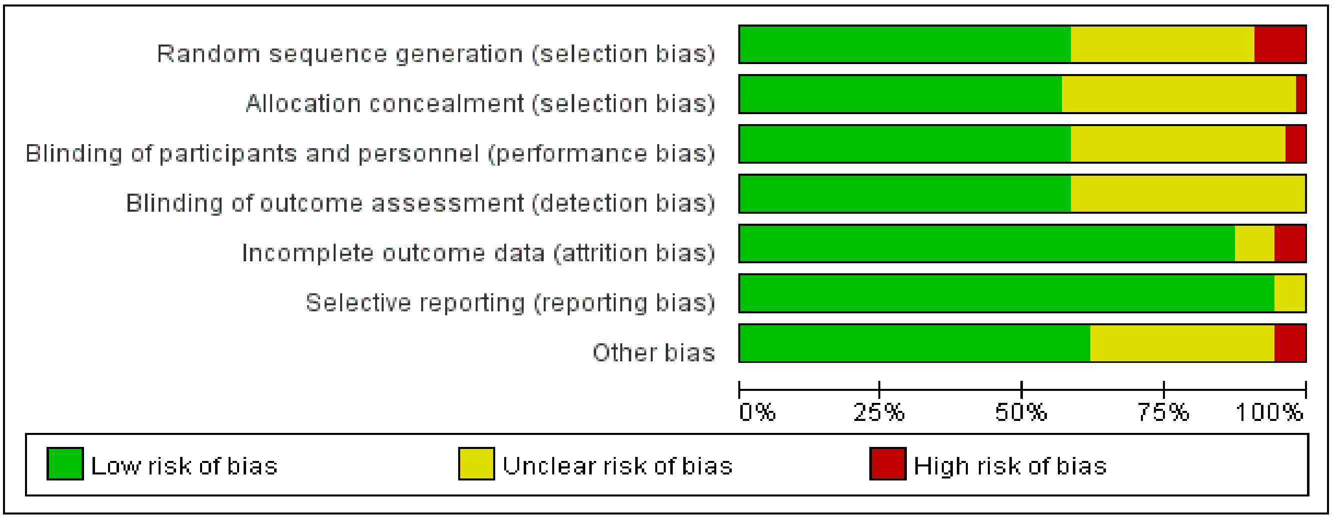

Figure S 4.2 Risk of bias summary: judgements about each bias item for each study

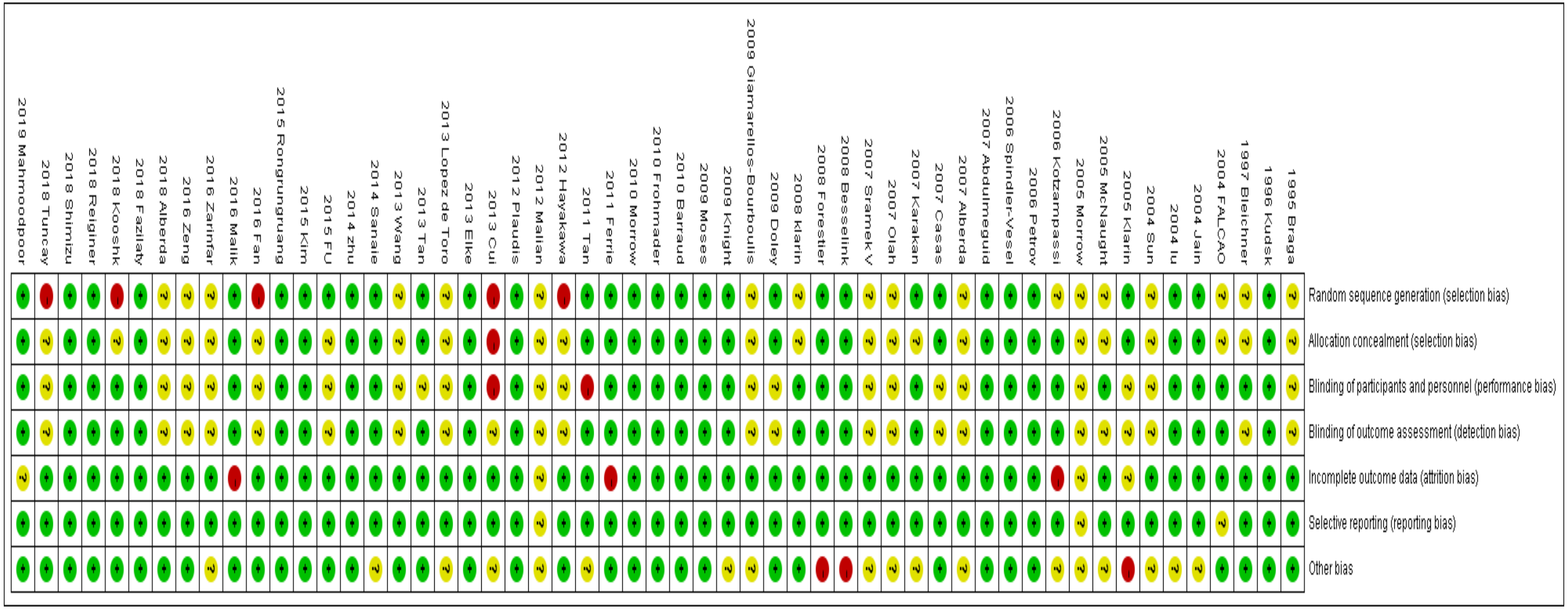

Appendix 5
Results from pairwise meta-analyses and network meta-analyses

Data are ORs (95% CrI) in the column-defining treatment compared with the row-defining treatment. With treatment as the boundary, the lower left part of the table is the result of network meta-analyses, and the upper right part of the table is the result of pairwise meta-analyses. For network meta-analyses, ORs lower than 1 favour the column-defining treatment. For pairwise meta-analyses, ORs higher than 1 favour the row-defining treatment. To obtain ORs for comparisons in the opposite direction,reciprocals should be taken. Significant results are in bold and underscored. OR=odds ratio. CrI=credible interval. EPN=Enteral nutrition. TPN= Total parenteral nutrition.

Table S 5.1 Pairwise and network estimated odds ratios (with 95% CrI) of interventions on nosocomial infection

Synbiotics	——	1.90 (0.94, 3.90)	2.50 (1.50, 4.62)	——
0.71 (0.38, 1.34)	Probiotics	2.90 (0.79, 11.42)	1.60 (1.10, 2.43)	8.30 (2.90, 25.02)
0.57 (0.32, 1.01)	0.8 (0.41, 1.57)	Prebiotics	2.10 (1.02, 4.70)	——
0.37 (0.22, 0.61)	0.52 (0.34, 0.77)	0.65 (0.35, 1.15)	EPN	2.02 (1.30, 3.30)
0.16 (0.08, 0.31)	0.23 (0.12, 0.39)	0.28 (0.13, 0.58)	0.44 (0.27, 0.68)	TPN

Figure S 5.1 Forest plot of the pairwise and network effect estimate on nosocomial infection

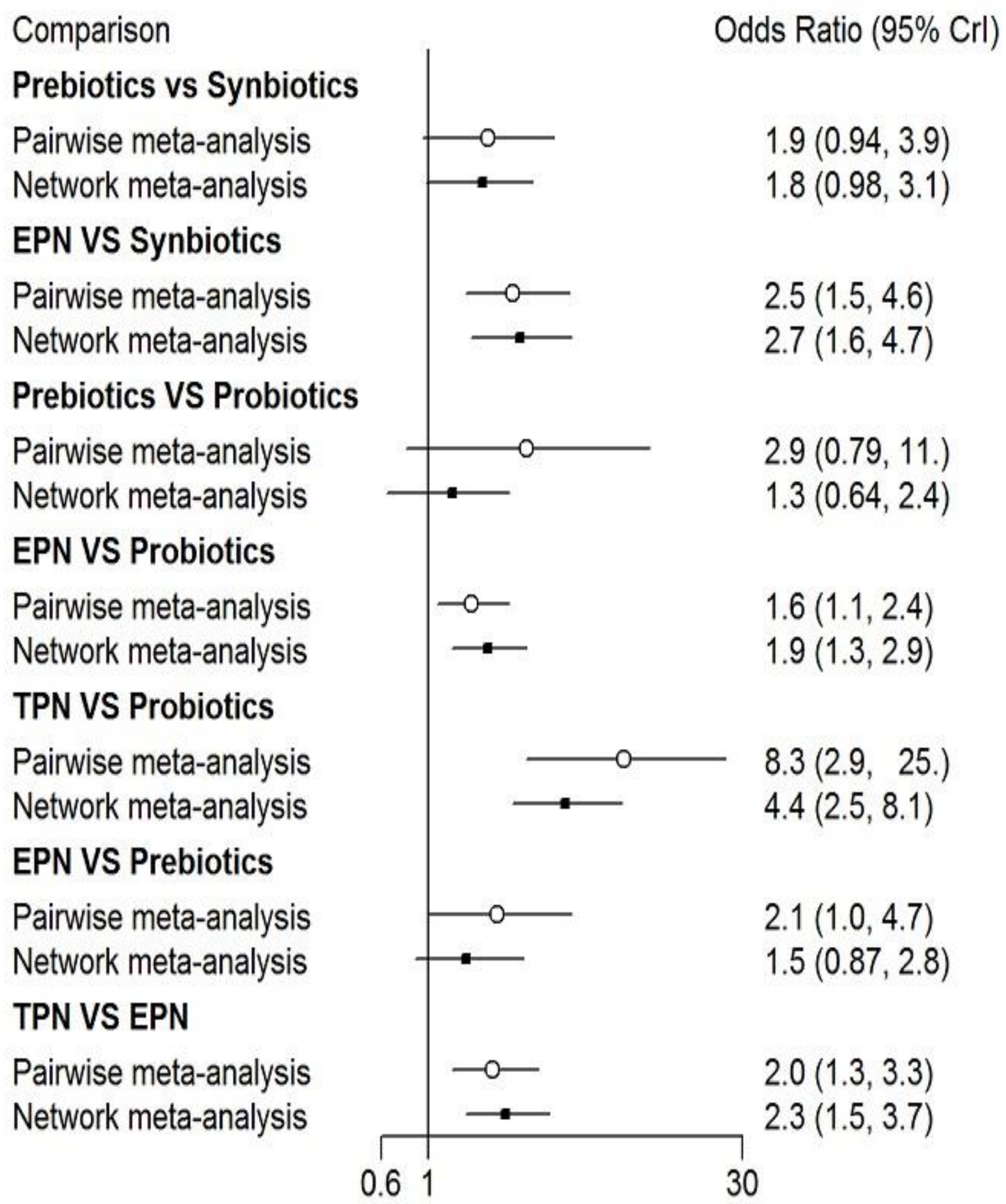

Table S 5.2 Pairwise and network estimated odds ratios (with 95% CrI) of interventions on hospital acquired pneumonia

Synbiotics	——	1.50 (0.46, 4.60)	3.60 (1.30, 12.02)	——
0.27 (0.05, 1.29)	Probiotics	——	0.80 (0.25, 2.80)	——
0.53 (0.19, 1.65)	1.93 (0.37, 13.92)	Prebiotics	0.87 (0.41, 5.60)	——
0.34 (0.11, 0.86)	1.24 (0.34, 4.17)	0.65 (0.14, 2.03)	EPN	2.40 (0.40, 15.30)
0.14 (0.02, 1.08)	0.52 (0.05, 4.57)	0.27 (0.02, 2.24)	0.43 (0.07, 2.61)	TPN

Figure S 5.2 Forest plot of the pairwise and network effect estimate on hospital acquired pneumonia

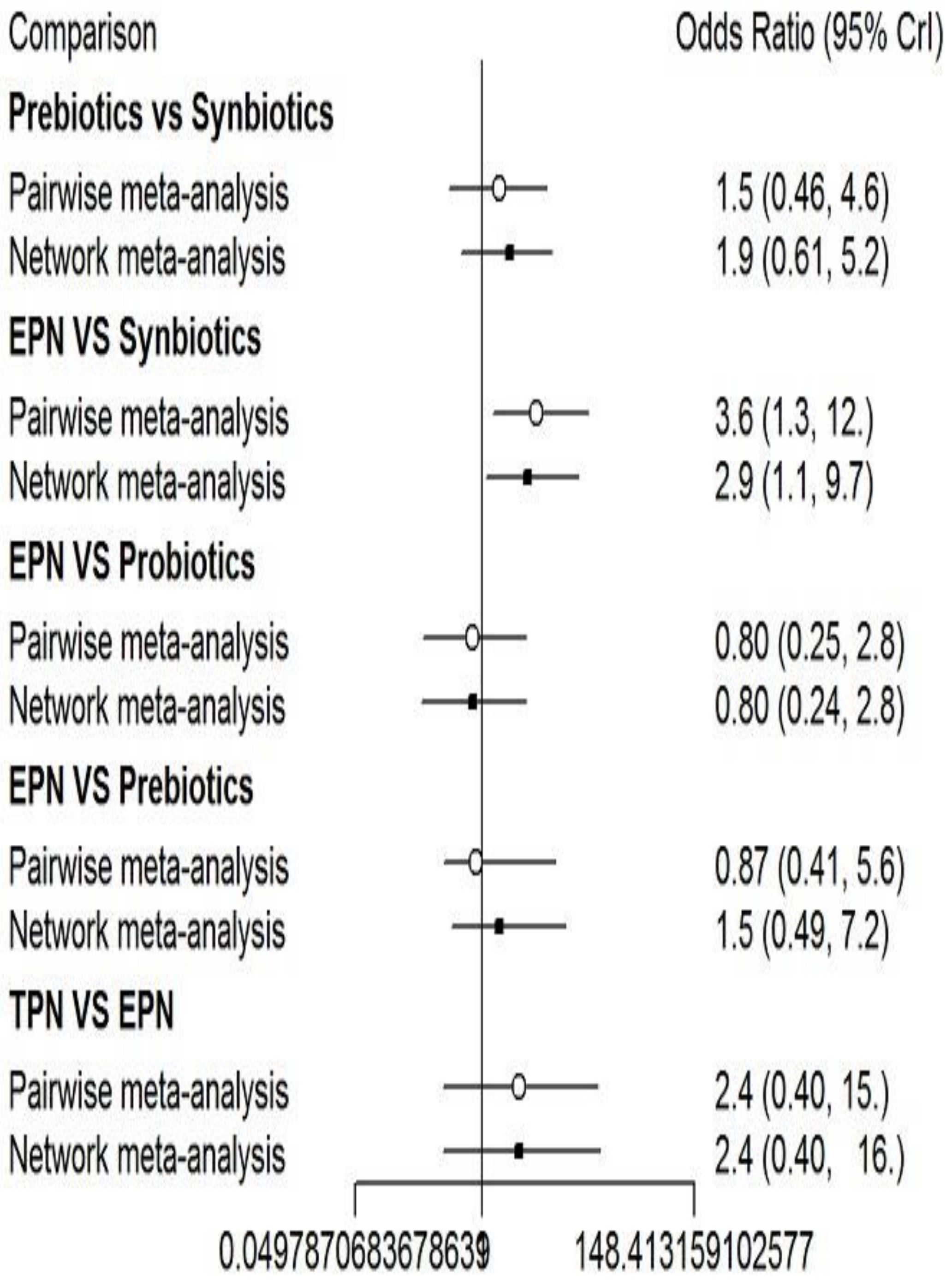

Table S 5.3 Pairwise and network estimated odds ratios (with 95% CrI) of interventions on ventilator-associated pneumonia

Synbiotics	——	——	2.00 (0.92, 4.50)	——
0.81 (0.29, 2.41)	Probiotics	2.90 (0.82, 11.00)	1.40 (0.89, 2.50)	——
0.56 (0.15, 2.24)	0.68 (0.25, 1.92)	Prebiotics	2.20 (0.64, 7.00)	——
0.50 (0.19, 1.21)	0.61 (0.33, 1.03)	0.90 (0.31, 2.29)	EPN	1.10 (0.50, 2.50)
0.46 (0.12, 1.61)	0.57 (0.18, 1.53)	0.83 (0.20, 2.95)	0.93 (0.36, 2.23)	TPN

Figure S 5.3 Forest plot of the pairwise and network effect estimate on ventilator-associated pneumonia

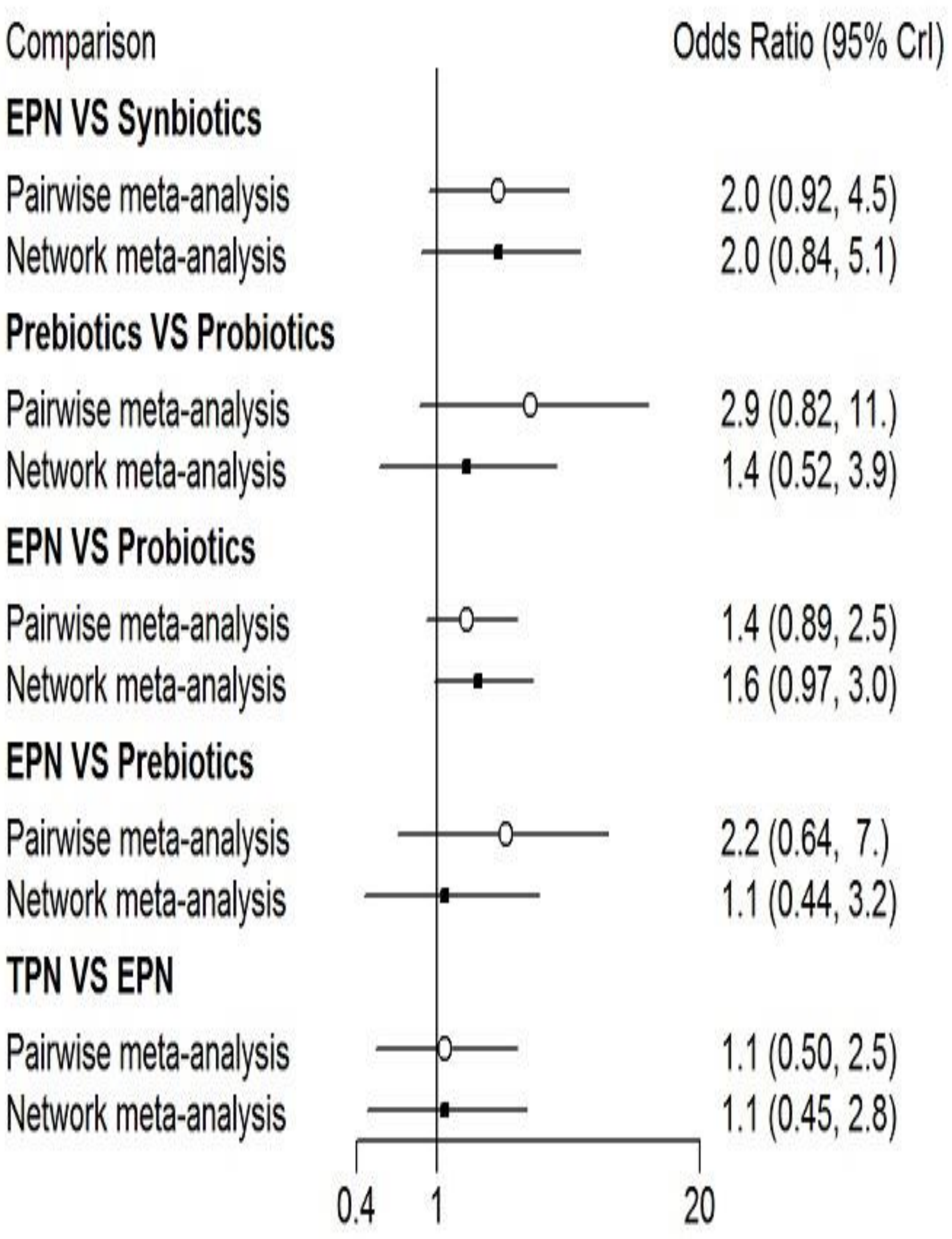

Table S 5.4 Pairwise and network estimated odds ratios (with 95% CrI) of interventions on bloodstream infection

Synbiotics	——	1.80 (0.32, 10.00)	2.50 (0.61, 11.00)	——
0.58 (0.13, 5.34)	Probiotics	——	1.60 (0.53, 12.00)	——
0.55 (0.14, 1.90)	0.96 (0.09, 5.23)	Prebiotics	1.60 (0.20, 12.00)	——
0.39 (0.12, 1.21)	0.68 (0.11, 1.86)	0.70 (0.16, 3.05)	EPN	2.40 (0.89, 11.00)
0.16 (0.02, 0.67)	0.28 (0.02, 1.11)	0.29 (0.04, 1.53)	0.42 (0.11, 1.06)	TPN

Figure S 5.4 Forest plot of the pairwise and network effect estimate on bloodstream infection

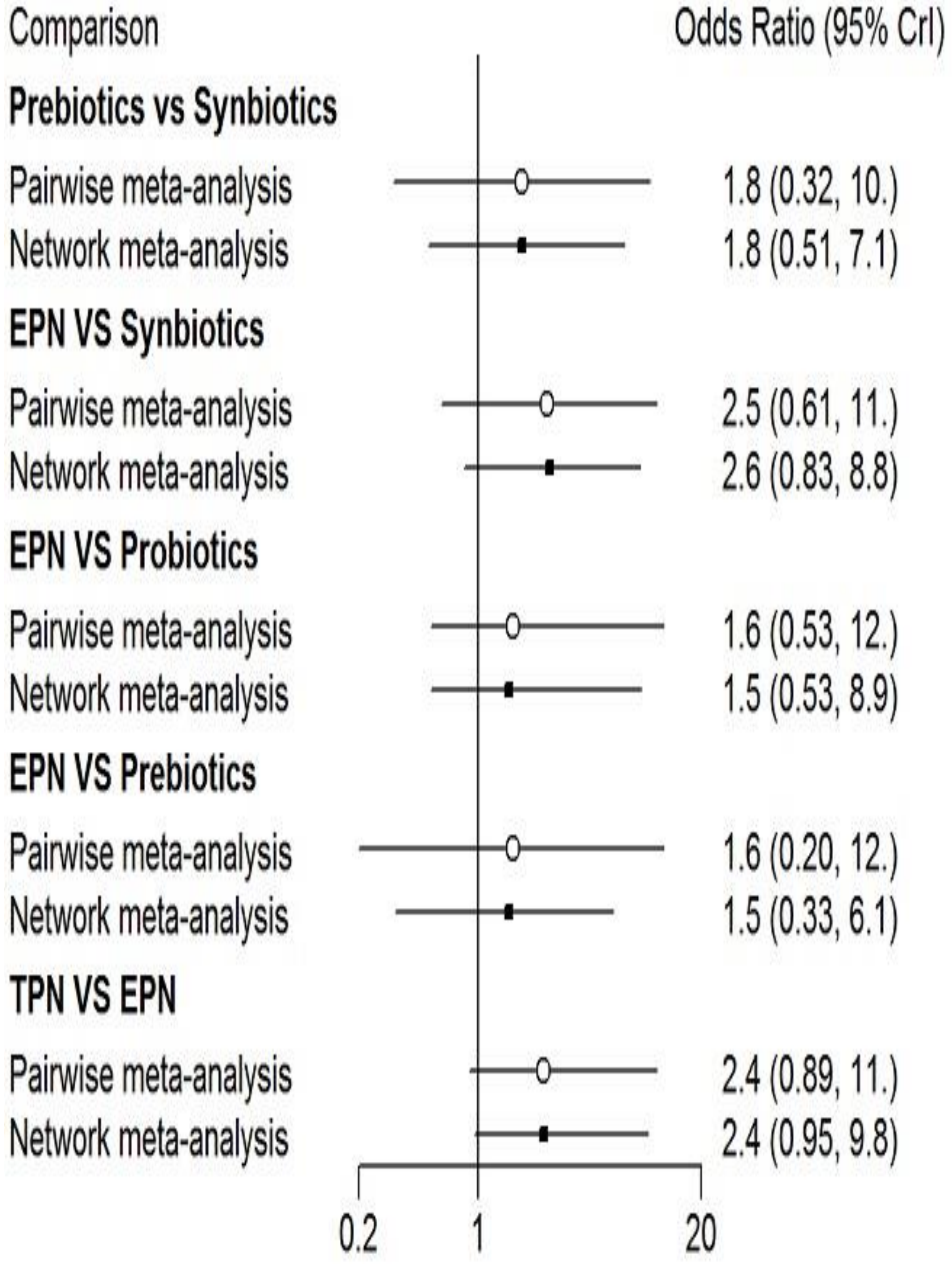

Table S 5.5 Pairwise and network estimated odds ratios (with 95% CrI) of interventions on catheter-related bloodstream infection

Synbiotics	—	2.90 (0.01, 1100)	4.90 (0.01, 2.3e+05)	—
0.24 (0.00, 6.33)	Probiotics	—	2.90 (0.01, 1200)	—
0.70 (0.07, 8.93)	2.90 (0.09, 497.91)	Prebiotics	1.0e+25(0.56, 6.0e+55)	—
**0.08 (0.01, 0.80)**	0.35 (0.03, 5.32)	0.12 (0.01, 1.46)	EPN	5.80 (0.70, 1.4e+05)
**0.02 (0.00, 0.33)**	0.09 (0.00, 1.51)	**0.03 (0.00, 0.59)**	0.25 (0.02, 1.17)	TPN

Figure S 5.5 Forest plot of the pairwise and network effect estimate on catheter-related bloodstream infection

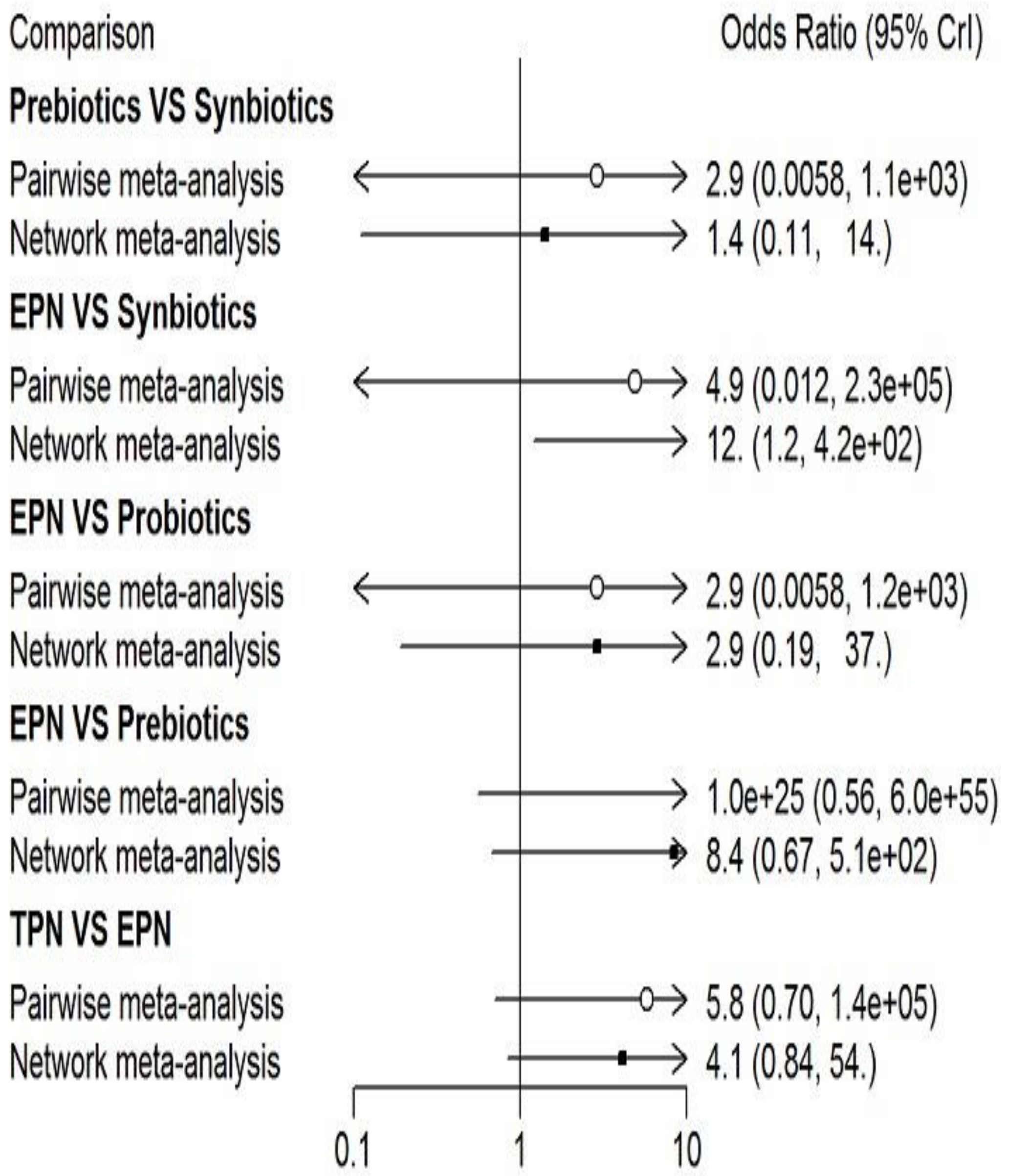

Table S 5.6 Pairwise and network estimated odds ratios (with 95% CrI) of interventions on urinary tract infections

Synbiotics	——	0.08 (0.15, 4.2)	3.00 (1.01, 9.51)	1.20 (0.42, 3.80)
0.34 (0.06, 1.44)	Probiotics	——	3.1e+11(24.50, 4.2e+70)	——
1.89 (0.46, 10.06)	5.50 (0.85, 53.75)	Prebiotics	1.10 (0.51, 2.50)	——
0.27 (0.08, 0.71)	0.79 (0.26, 2.44)	0.14 (0.02, 0.68)	EPN	——
0.25 (0.05, 0.83)	0.73 (0.18, 2.74)	0.13 (0.01, 0.72)	0.92 (0.40, 1.98)	TPN

Figure S 5.6 Forest plot of the pairwise and network effect estimate on urinary tract infections

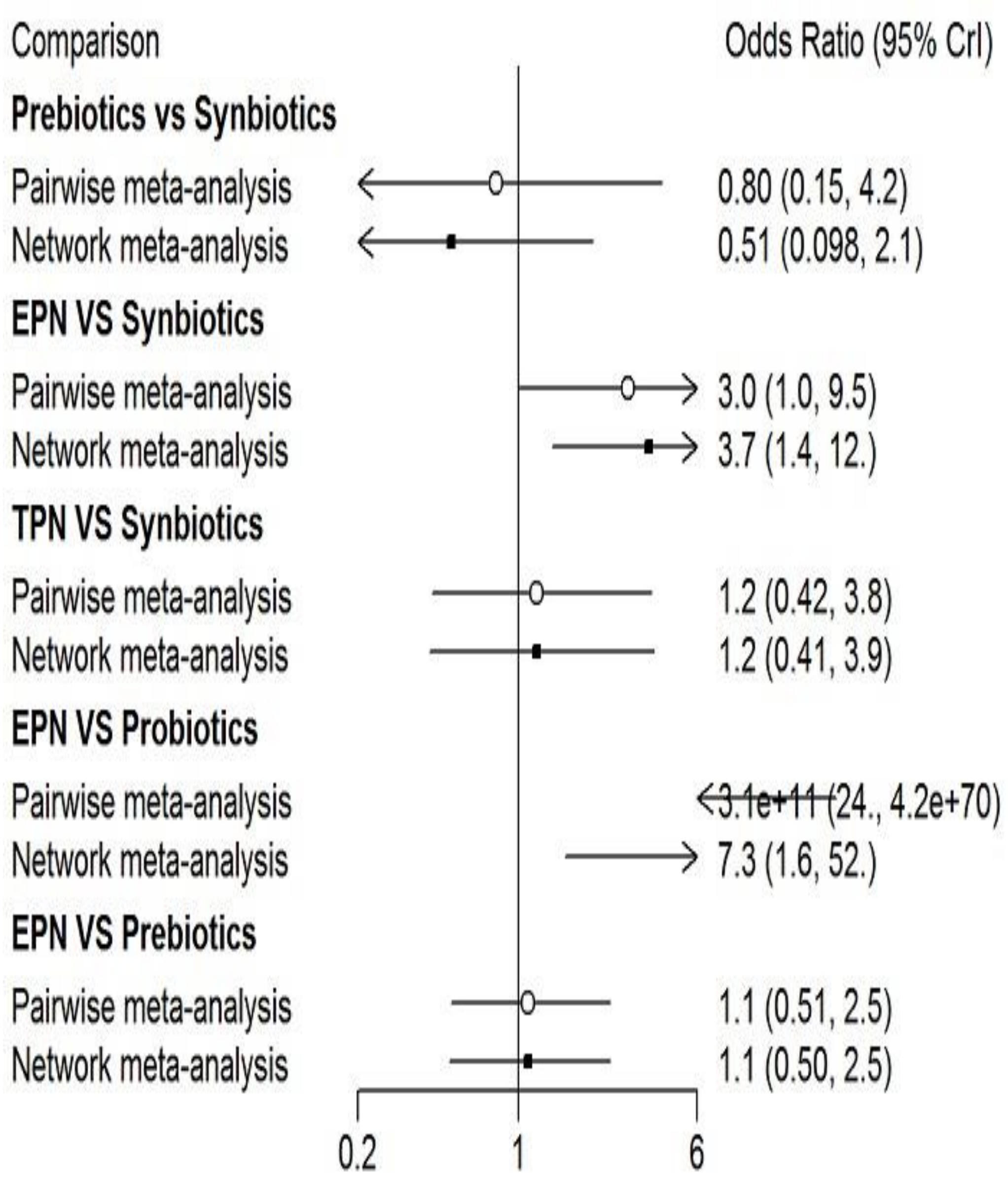

Table S 5.7 Pairwise and network estimated odds ratios (with 95% CrI) of interventions on sepsis

Synbiotics	——	1.80 (0.36, 8.40)	2.90 (1.40, 6.70)	——
0.73 (0.22, 2.66)	Probiotics	——	2.30 (0.72, 7.40)	4.80 (1.06, 22.40)
0.63 (0.17, 2.20)	0.85 (0.17, 4.25)	Prebiotics	2.70 (0.42, 25.10)	——
0.34 (0.16, 0.70)	0.47 (0.16, 1.22)	0.55 (0.15, 1.90)	EPN	2.50 (0.99, 5.10)
0.14 (0.05, 0.44)	0.19 (0.06, 0.58)	0.23 (0.05, 1.01)	0.41 (0.2, 1.00)	TPN

Figure S 5.7 Forest plot of the pairwise and network effect estimate on sepsis

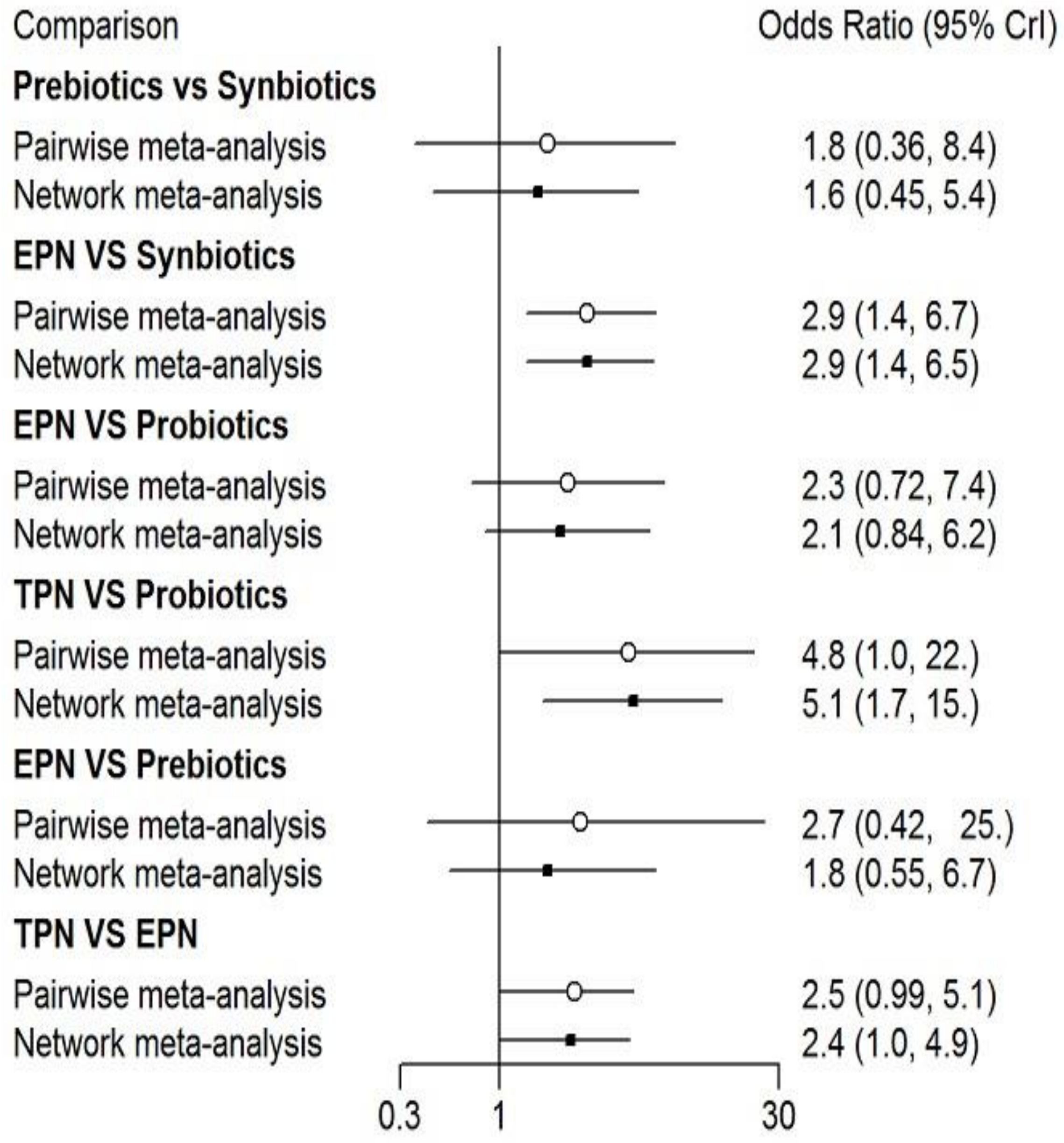

Table S 5.8 Pairwise and network estimated odds ratios (with 95% CrI) of interventions on diarrhea

Synbiotics	——	——	2.00 (0.56, 7.10)	——
0.86 (0.14, 5.62)	Probiotics	2.10 (0.43, 11.40)	1.30 (0.72, 2.50)	——
2.04 (0.24, 21.01)	2.32 (0.57, 11.87)	Prebiotics	**17.00 (3.42, 110)**	——
0.49 (0.09, 2.56)	0.57 (0.24, 1.23)	**0.24 (0.05, 0.94)**	EPN	0.38 (0.15, 0.83)
1.44 (0.19, 10.44)	1.65 (0.40, 6.72)	0.71 (0.10, 4.25)	2.90 (0.97, 9.28)	TPN

Figure S 5.8 Forest plot of the pairwise and network effect estimate on diarrhea

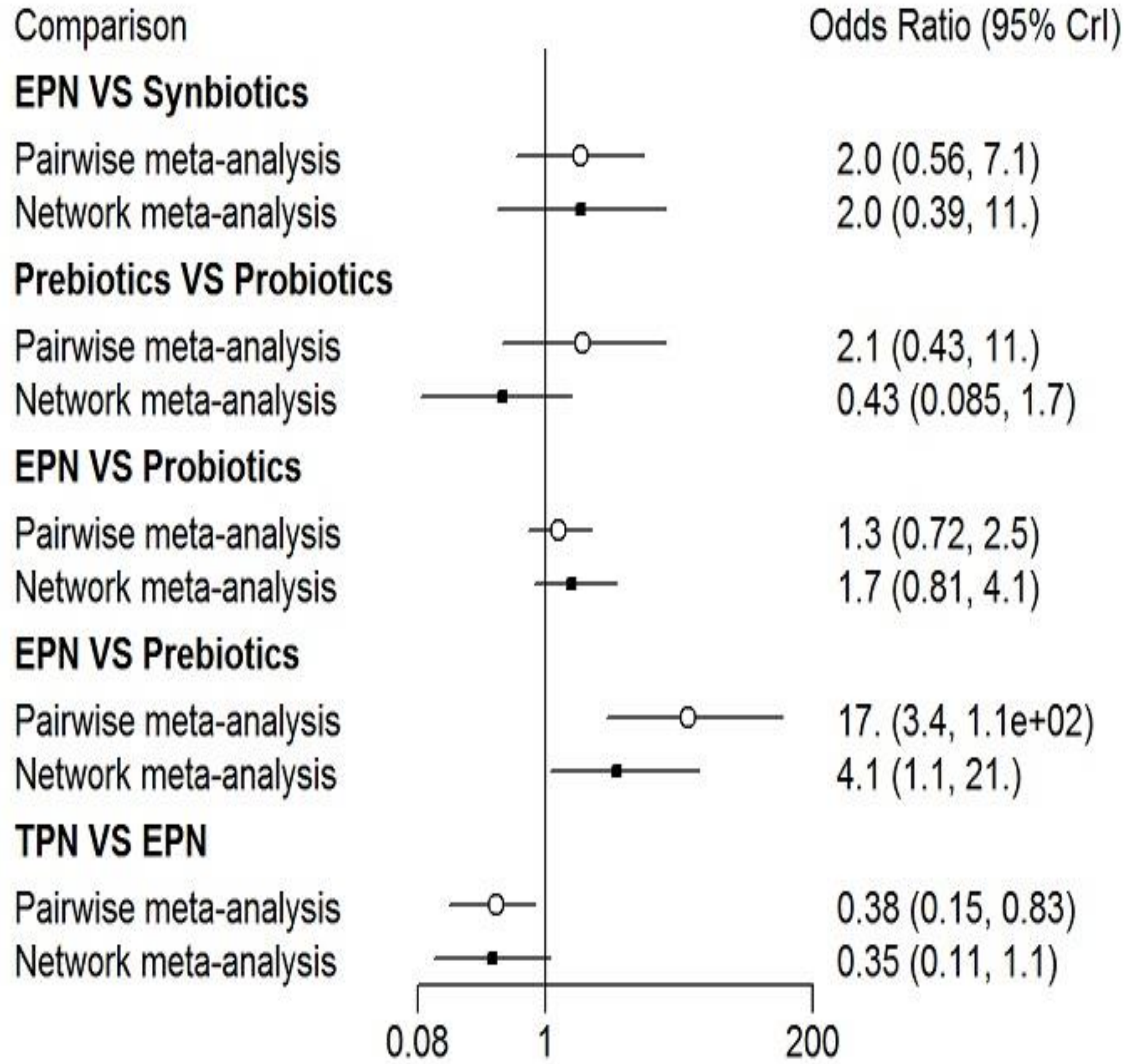

Table S 5.9 Pairwise and network estimated odds ratios (with 95% CrI) of interventions on hospital mortality

Synbiotics	— —	1.40 (0.54, 3.90)	1.30 (0.79, 2.30)	— —
0.71 (0.38, 1.30)	Probiotics	1.30 (0.38, 4.70)	1.00 (0.68, 1.60)	2.10 (0.55, 8.20)
0.93 (0.48, 1.85)	1.31 (0.66, 2.72)	Prebiotics	3.10 (1.12, 9.10)	— —
0.64 (0.38, 1.02)	0.90 (0.60, 1.31)	0.68 (0.35, 1.28)	EPN	1.40 (0.94, 2.30)
<u>0.43 (0.21, 0.80)</u>	0.60 (0.33, 1.03)	0.46 (0.20, 1.01)	0.67 (0.42, 1.03)	TPN

Figure S 5.9 Forest plot of the pairwise and network effect estimate on hospital mortality

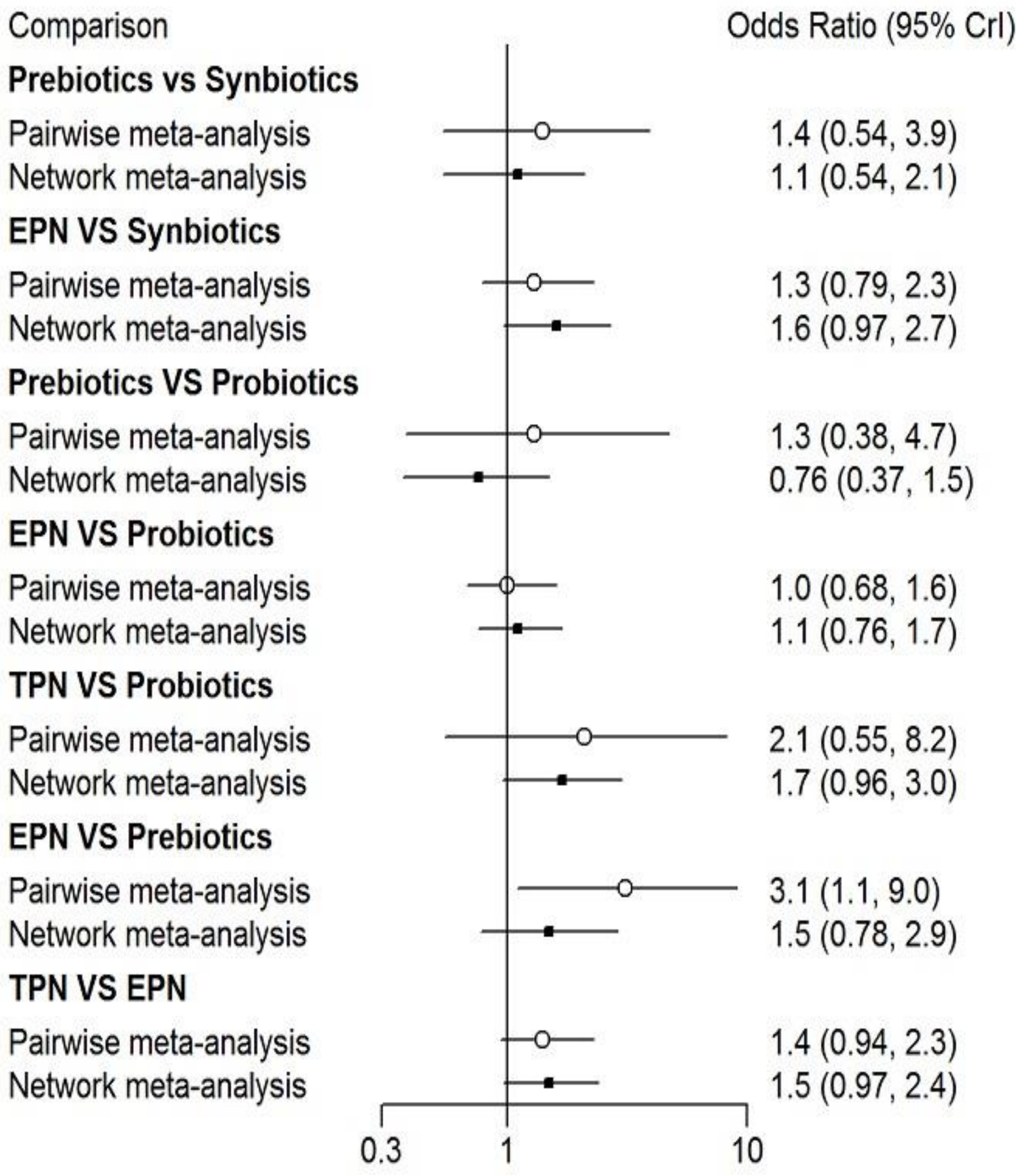

Table S 5.10 Pairwise and network estimated odds ratios (with 95% CrI) of interventions on ICU mortality

Synbiotics	——	0.94 (0.14, 6.10)	1.30 (0.73, 2.40)	——
0.76 (0.33, 1.75)	Probiotics	——	0.98 (0.54, 1.80)	——
0.85 (0.20, 4.22)	1.13 (0.23, 6.27)	Prebiotics	0.79 (0.01, 9.50)	——
0.78 (0.43, 1.38)	1.03 (0.56, 1.81)	0.91 (0.18, 3.79)	EPN	0.91 (0.42, 2.00)
0.86 (0.34, 2.18)	1.13 (0.43, 2.82)	1.01 (0.17, 4.92)	1.10 (0.52, 2.37)	TPN

Figure S 5.10 Forest plot of the pairwise and network effect estimate on ICU mortality

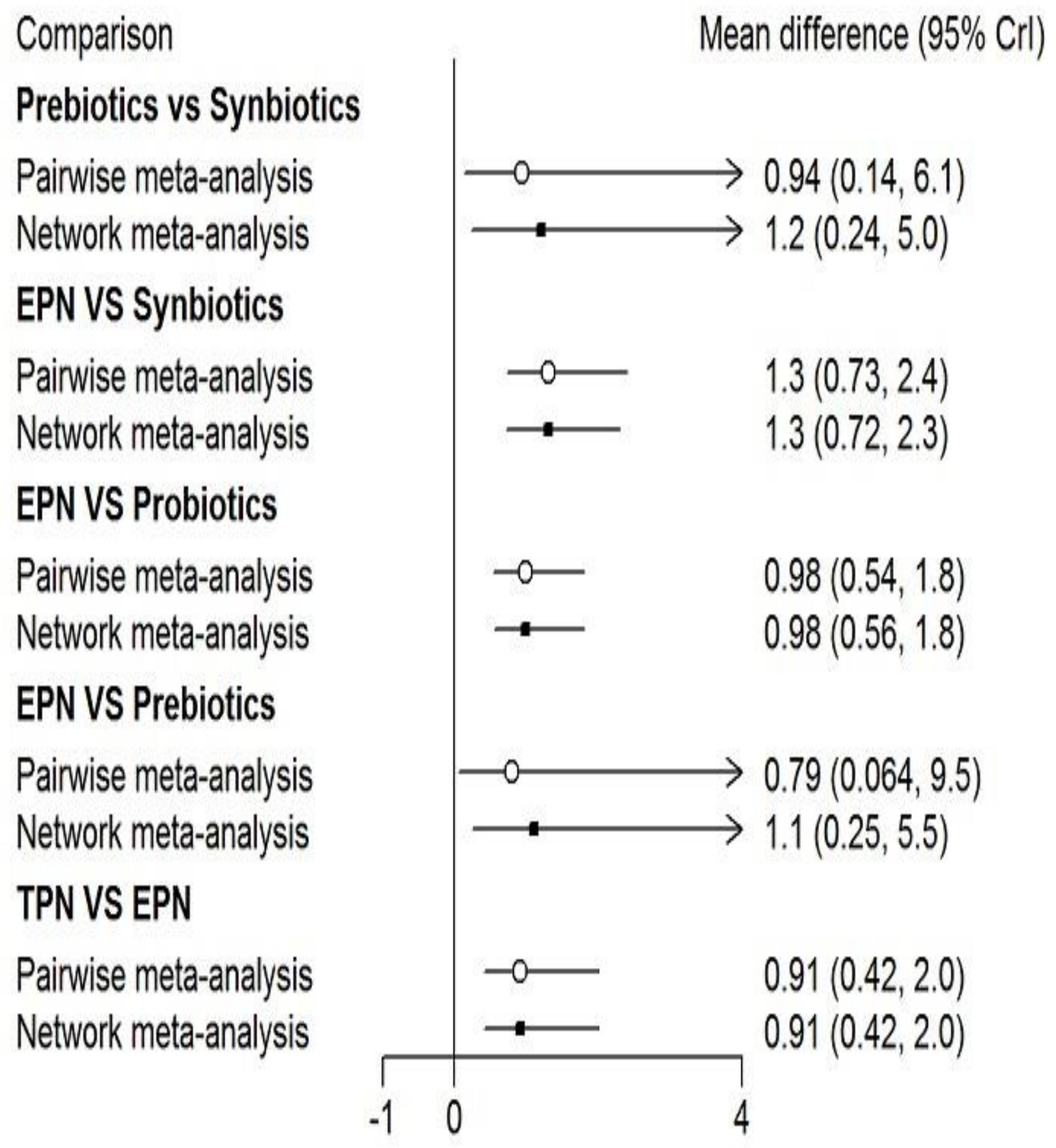

Table S 5.11 Pairwise and network estimated mean differecen (with 95% CrI) of interventions on hospital lengtn of stay

Synbiotics	--	4.80 (-3.40, 13.10)	1.50 (-4.70, 7.70)	--
-1.01 (-7.28, 5.09)	Probiotics	-0.30 (-11.08, 9.90)	1.40 (-2.70, 5.10)	12.00 (4.00, 19.10)
-1.57 (-7.47, 4.52)	-0.53 (-6.24, 5.41)	Prebiotics	3.91 (-3.20, 11.00)	--
-3.39 (-8.75, 1.97)	-2.38 (-5.75, 1.22)	-1.82 (-7.08, 3.4)	EPN	3.60 (0.25, 6.90)
-7.62 (-13.75, -1.43)	-6.6 (-10.7, -2.23)	-6.05 (-12.04, 0.04)	-4.23 (-7.33, -0.97)	TPN

Figure S 5.11 Forest plot of the pairwise and network effect estimate on v hospital lengtn of stay

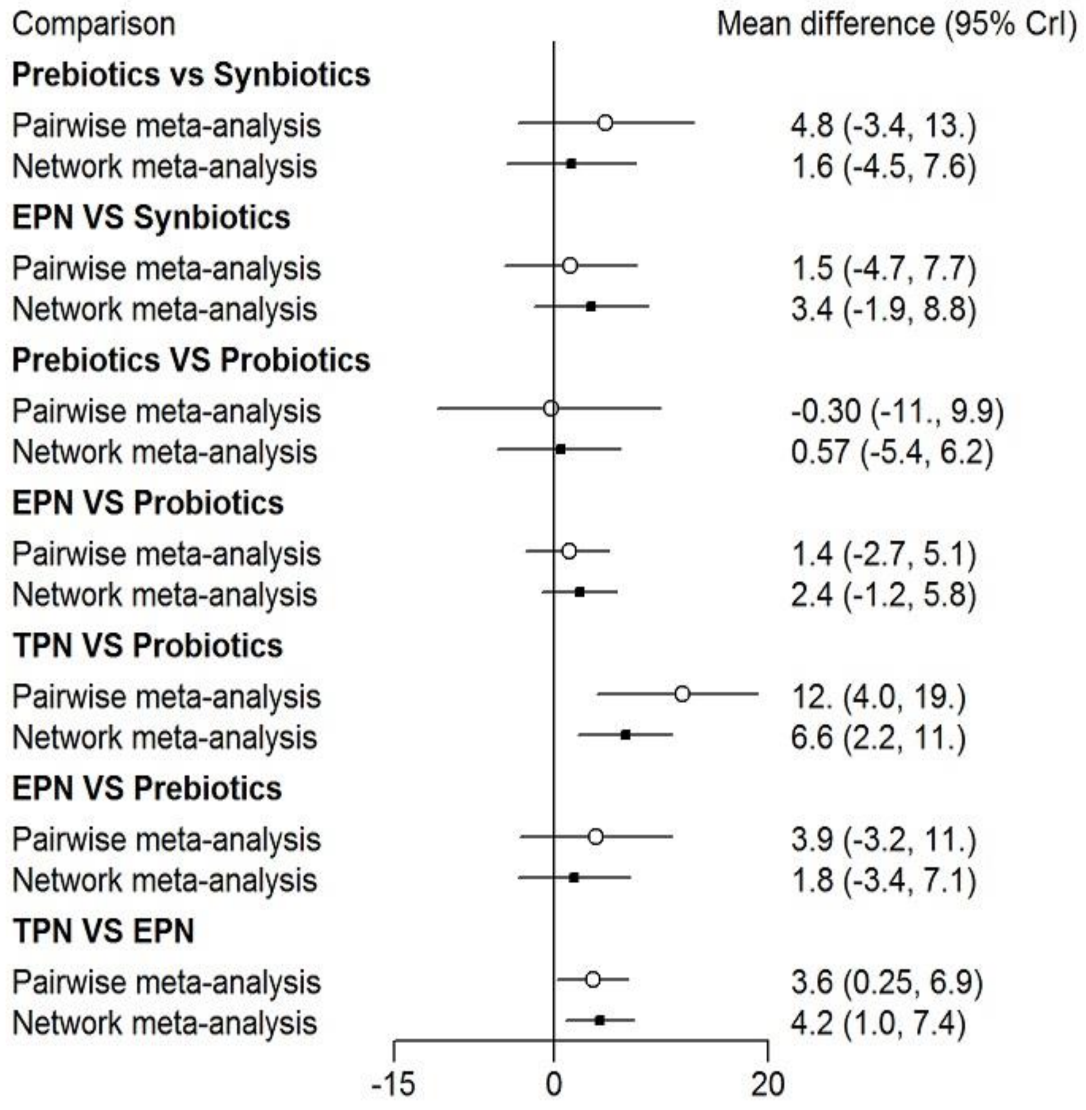

Table S 5.12 Pairwise and network estimated mean differecen (with 95% CrI) of interventions on ICU length of stay

Synbiotics	——	-0.61 (-6.70, 5.40)	1.50 (-2.50, 5.80)	——
0.41 (-3.90, 4.43)	Probiotics	-0.21 (-9.20, 8.80)	1.60 (-0.90, 4.00)	——
0.38 (-3.86, 4.49)	-0.07 (-3.99, 4.13)	Prebiotics	0.82 (-3.80, 5.40)	——
-1.14 (-4.85, 2.28)	-1.56 (-3.86, 0.76)	-1.50 (-5.25, 2.03)	EPN	1.80 (-1.40, 4.80)
-2.91 (-7.73, 1.80)	-3.34 (-7.07, 0.68)	-3.27 (-8.04, 1.48)	-1.78 (-4.80, 1.46)	TPN

Figure S 5.12 Forest plot of the pairwise and network effect estimate on ICU length of stay

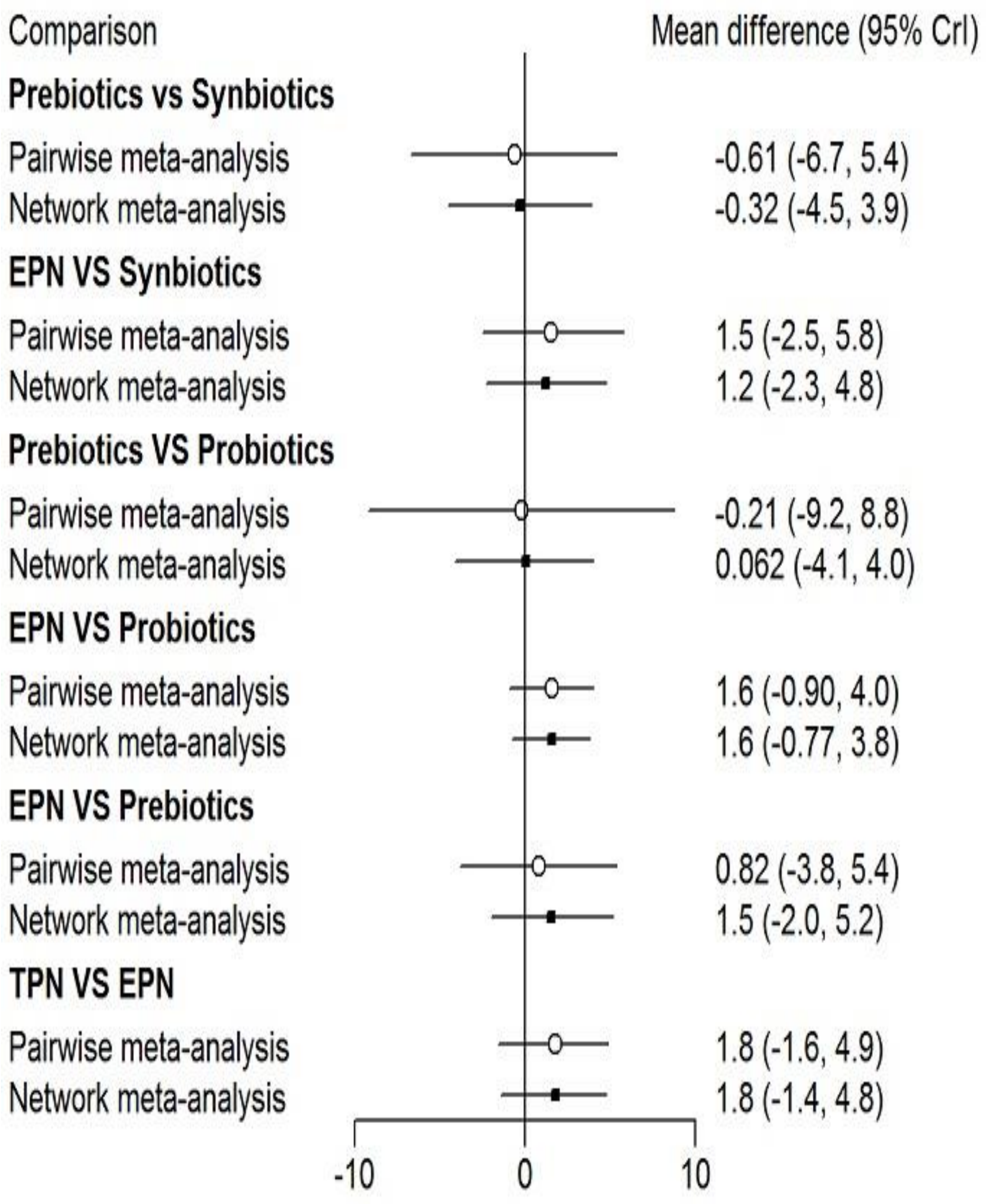

Table S 5.13 Pairwise and network estimated mean differecen (with 95% CrI) of interventions on the duration of mechanical ventilation

Synbiotics	--	1.80 (-6.20, 9.70)	1.70 (-3.10, 7.00)	--
1.39 (-4.68, 6.91)	Probiotics	-0.09 (-9.70, 9.60)	4.20 (-0.24, 8.80)	--
0.34 (-5.26, 5.62)	-1.05 (-6.39, 4.56)	Prebiotics	2.82 (-3.00, 9.50)	--
-2.55 (-7.31, 1.55)	-3.93 (-7.98, -0.02)	-2.84 (-7.83, 1.53)	EPN	3.50 (-1.40, 8.30)
-6.07 (-12.78, -0.16)	-7.45 (-13.52, -1.44)	-6.39 (-13.12, -0.15)	-3.55 (-8.02, 1.01)	TPN

Figure S 5.13 Forest plot of the pairwise and network effect estimate on the duration of mechanical ventilation

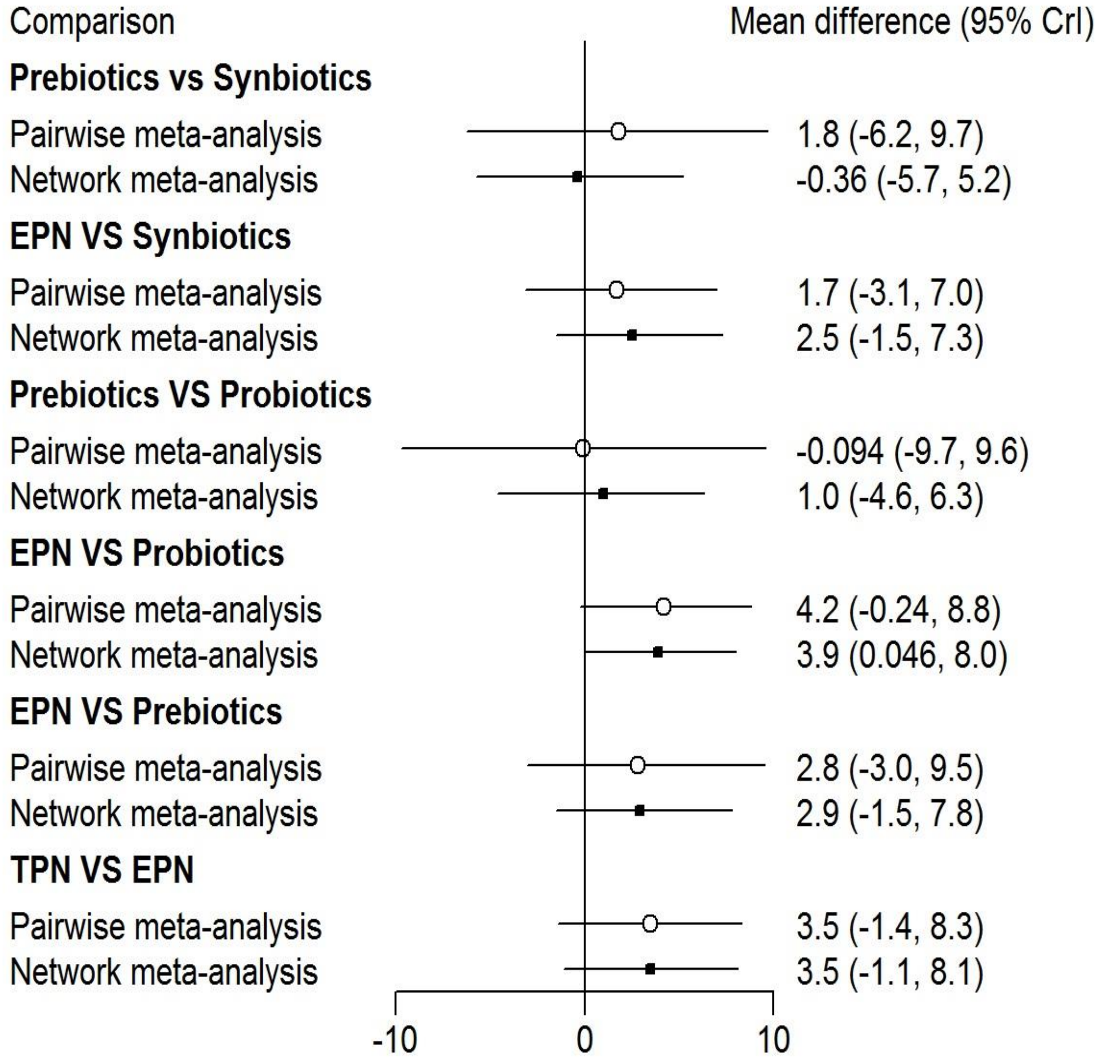

Appendix file 6

Results from network meta-analyses

The size of the nodes corresponds to the total number of participants that study the treatments. The (directly) comparable treatments are linked with a line. The thickness of the line corresponds to the standard error of trials that study this comparison. The colours of the line corresponds to the quality of trials that study this comparison. low risk of bias [green] ,moderate risk of bias [yellow].

Figure S 6.1 Network plot of all intervention comparisons for nosocomial infection

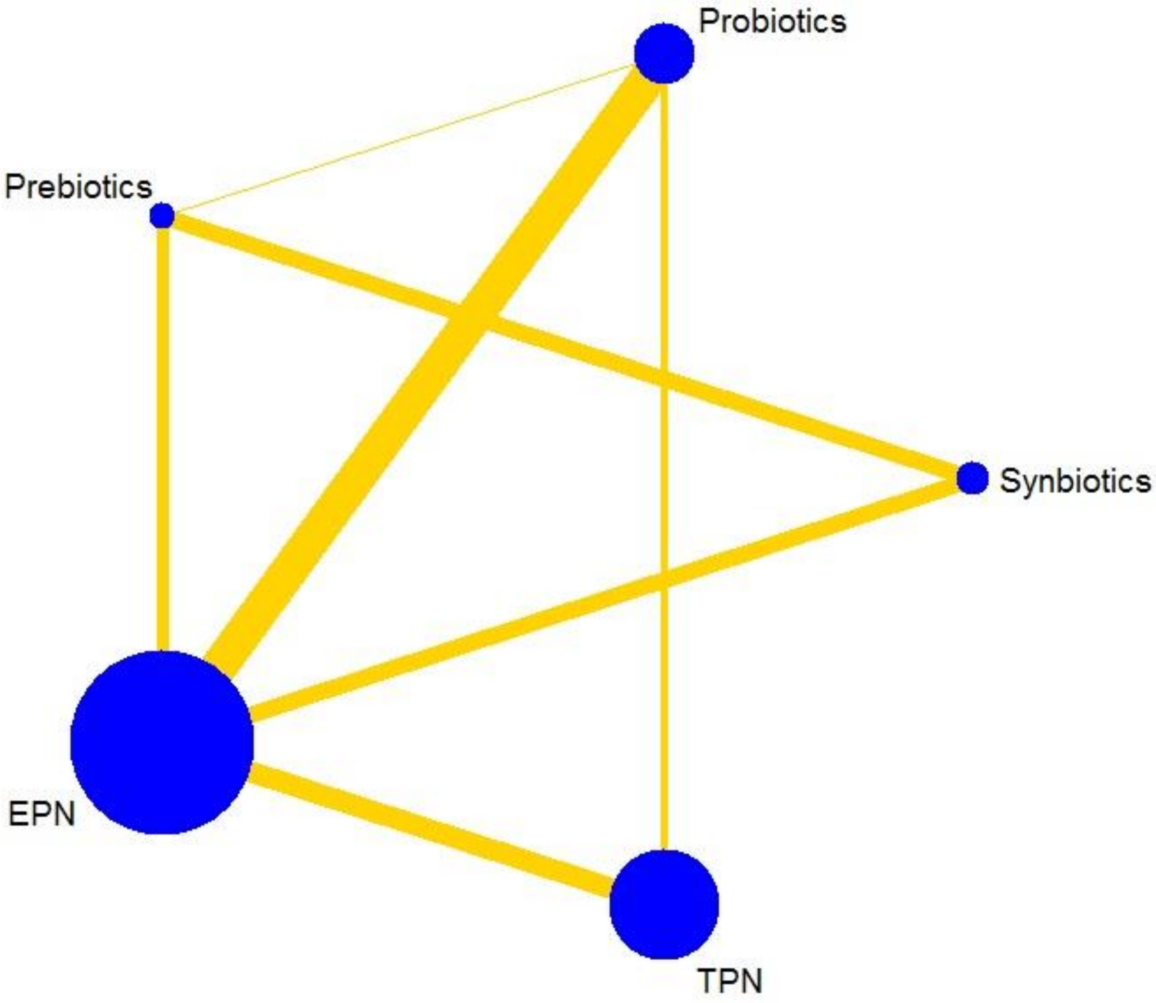

Figure S 6.2 Network plot of all intervention comparisons for hospital acquired pneumonia

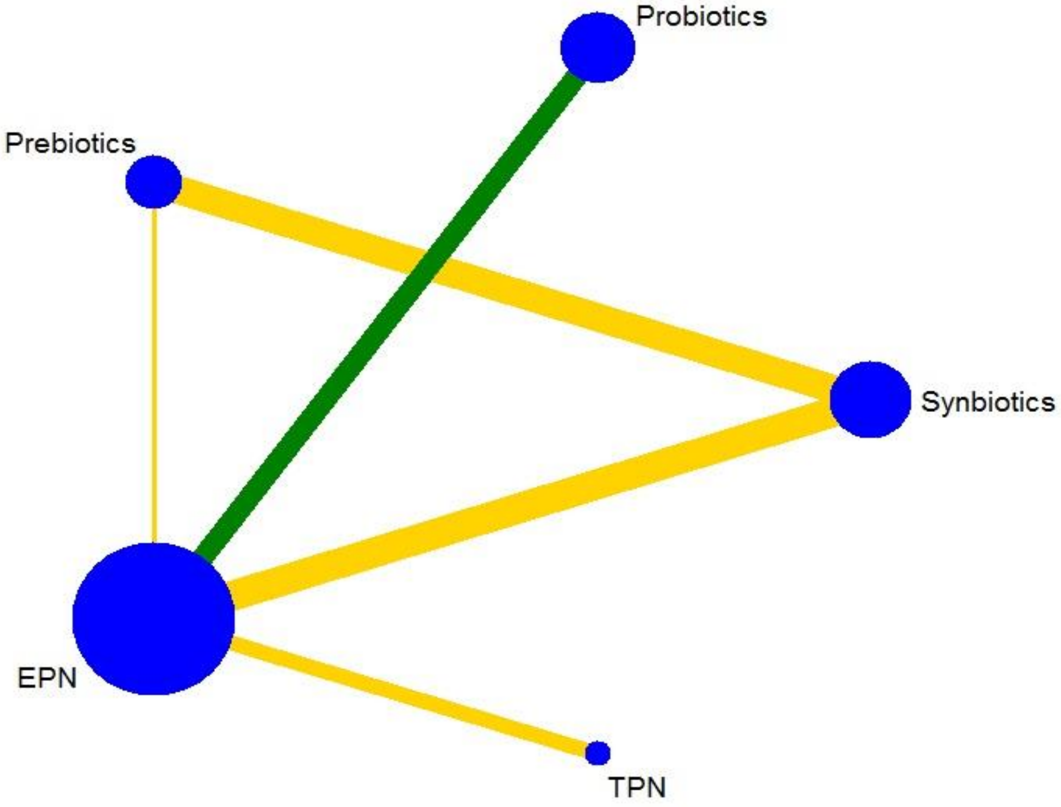

Figure S 6.3 Network plot of all intervention comparisons for ventilator-associated pneumonia

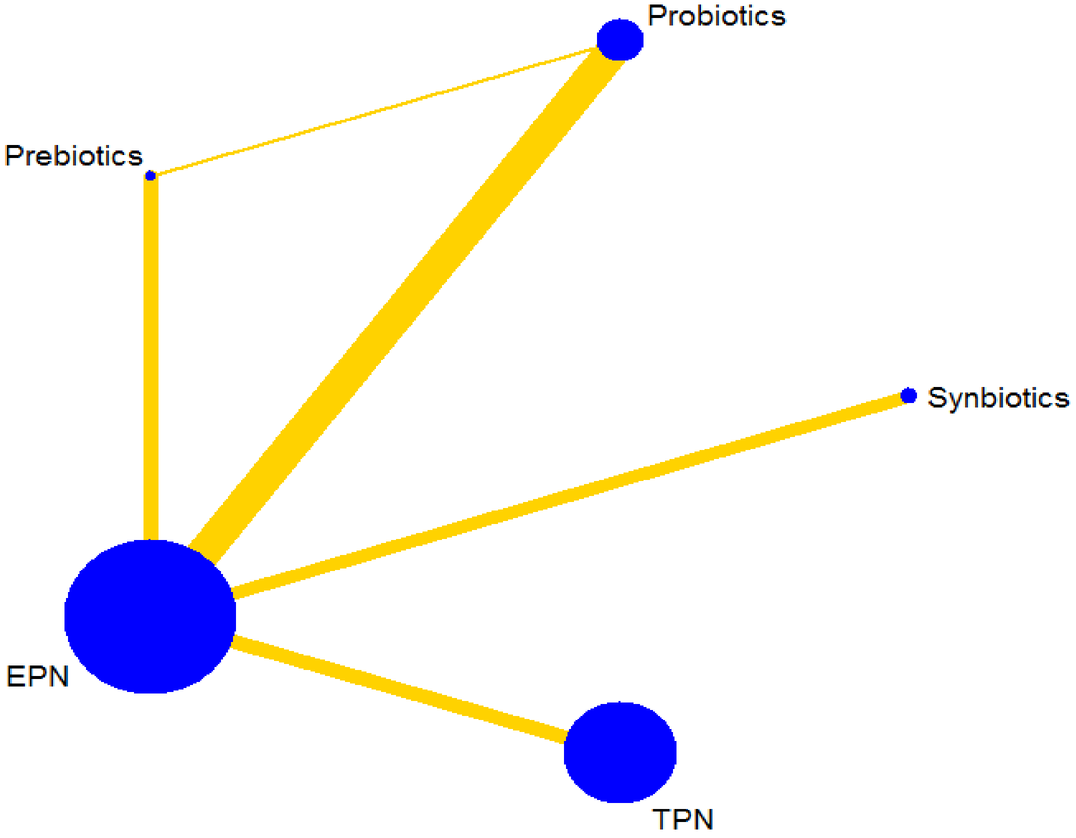

Figure S 6.4 Network plot of all intervention comparisons for bloodstream infections

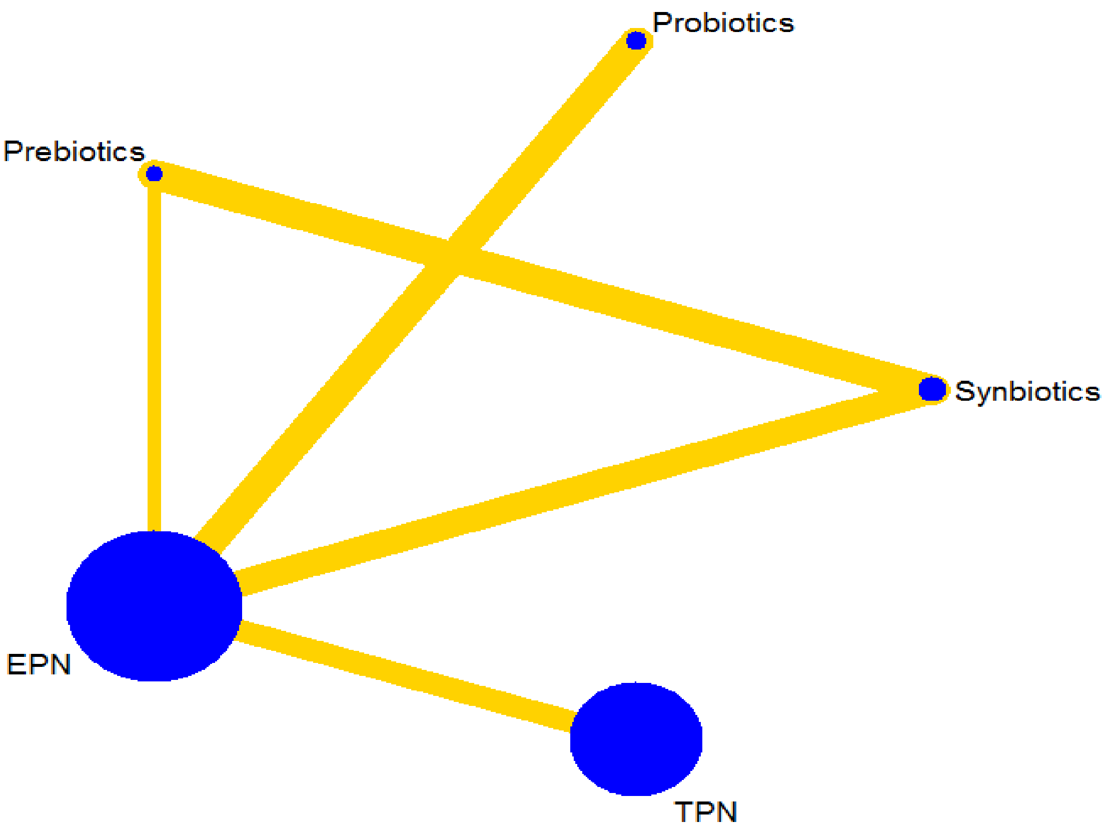

Figure S 6.5 Network plot of all intervention comparisons for catheter-related bloodstream infection

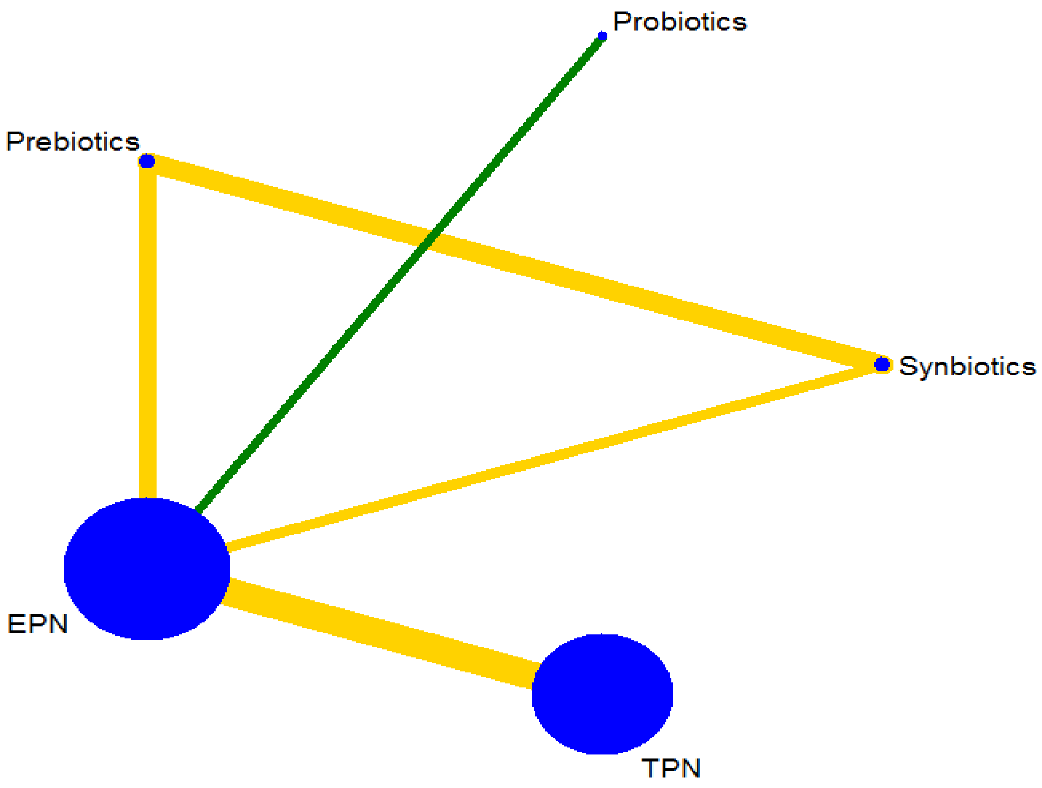

Figure S 6.6 Network plot of all intervention comparisons for urinary tract infections

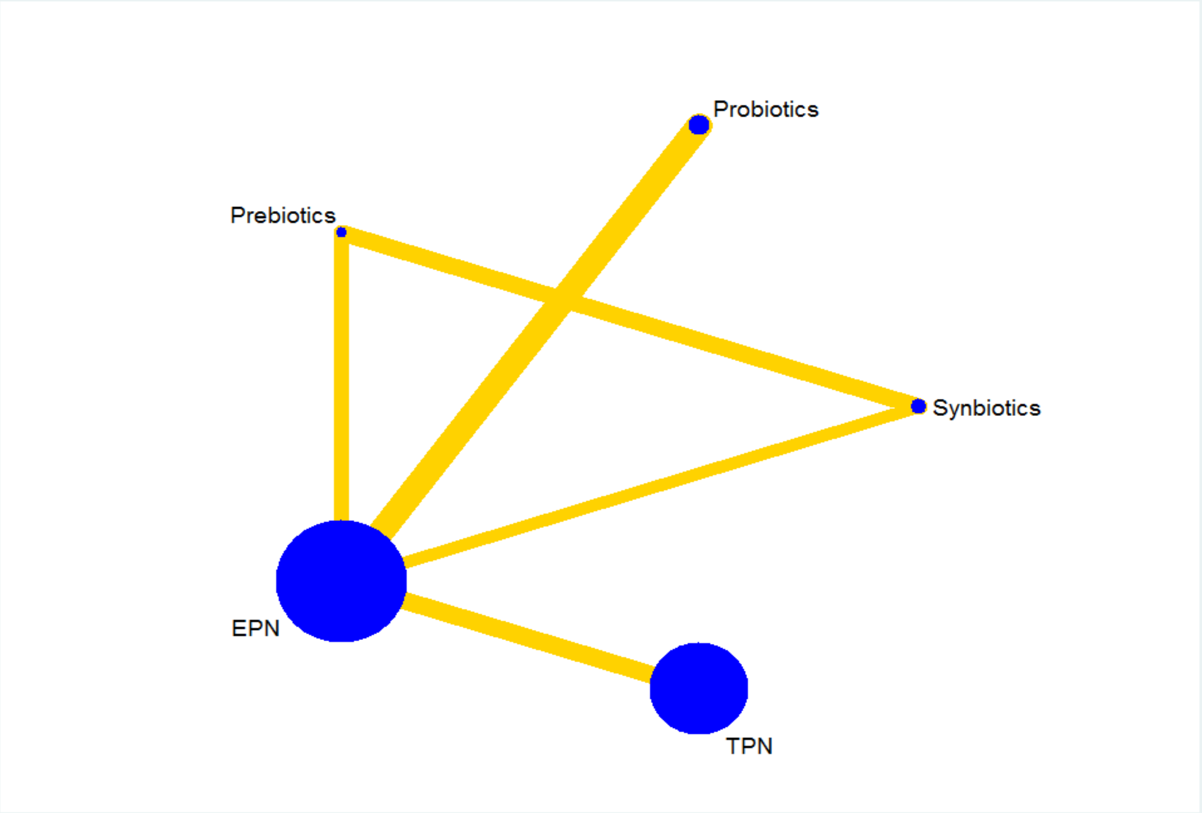

Figure S 6.7 Network plot of all intervention comparisons for sepsis

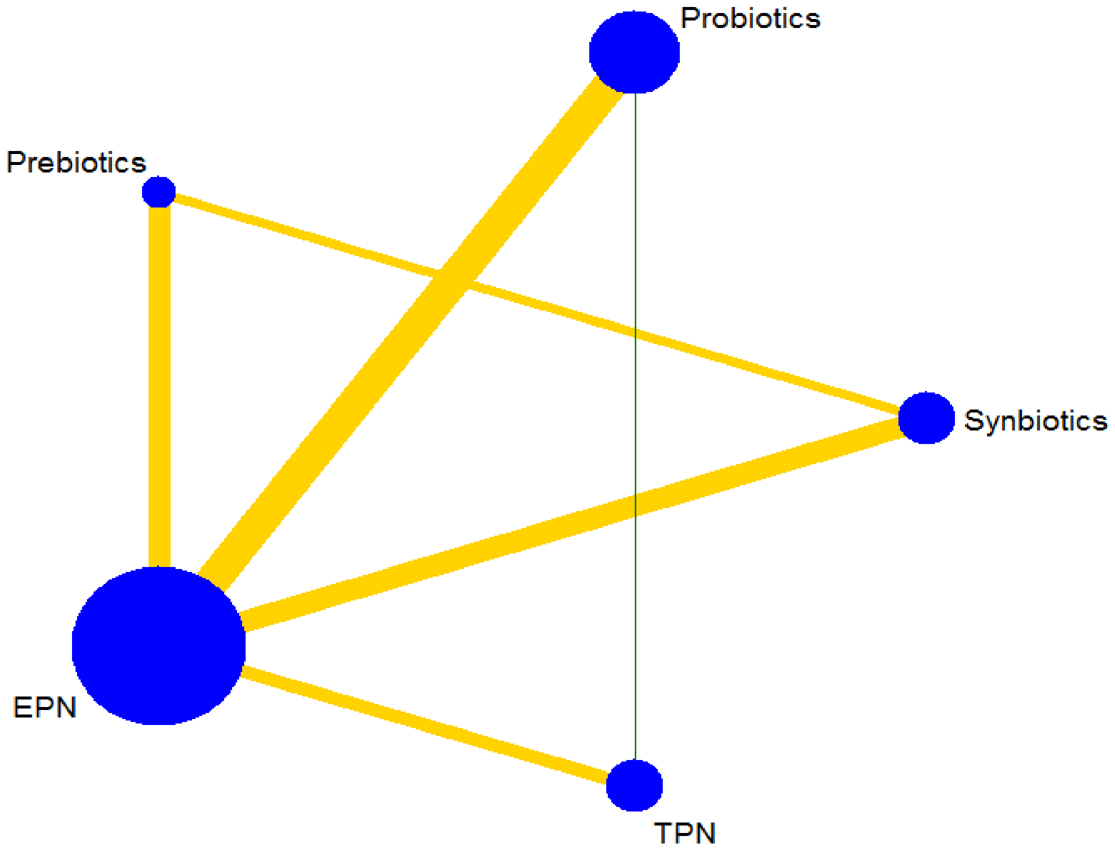

Figure S 6.8 Network plot of all intervention comparisons for diarrhea

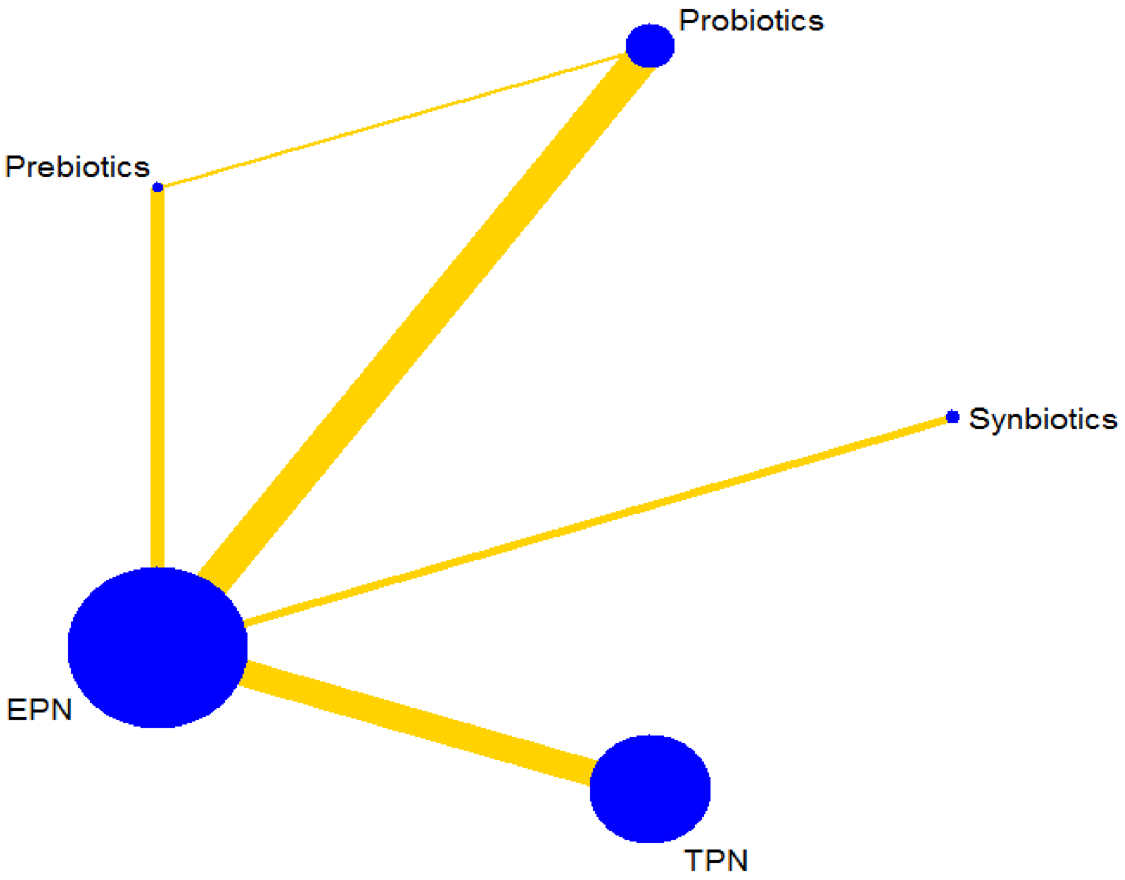

Figure S 6.9 Network plot of all intervention comparisons for hospital mortality

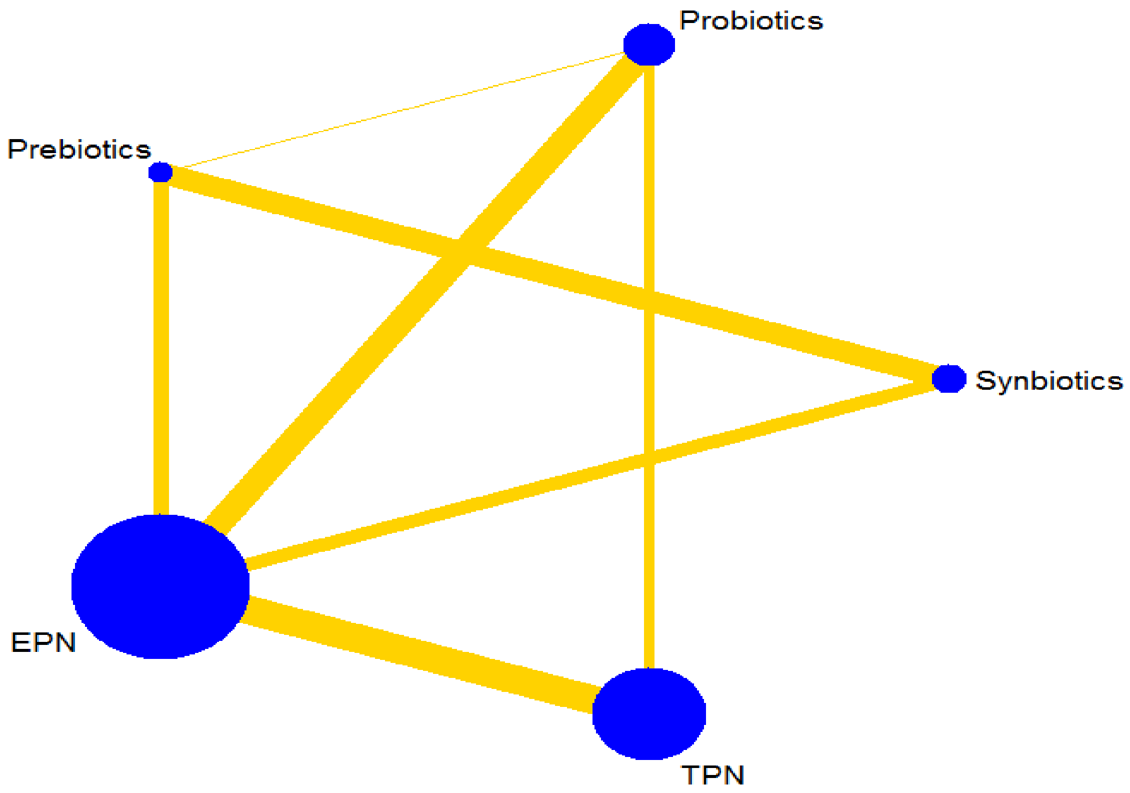

Figure S 6.10 Network plot of all intervention comparisons for ICU mortality

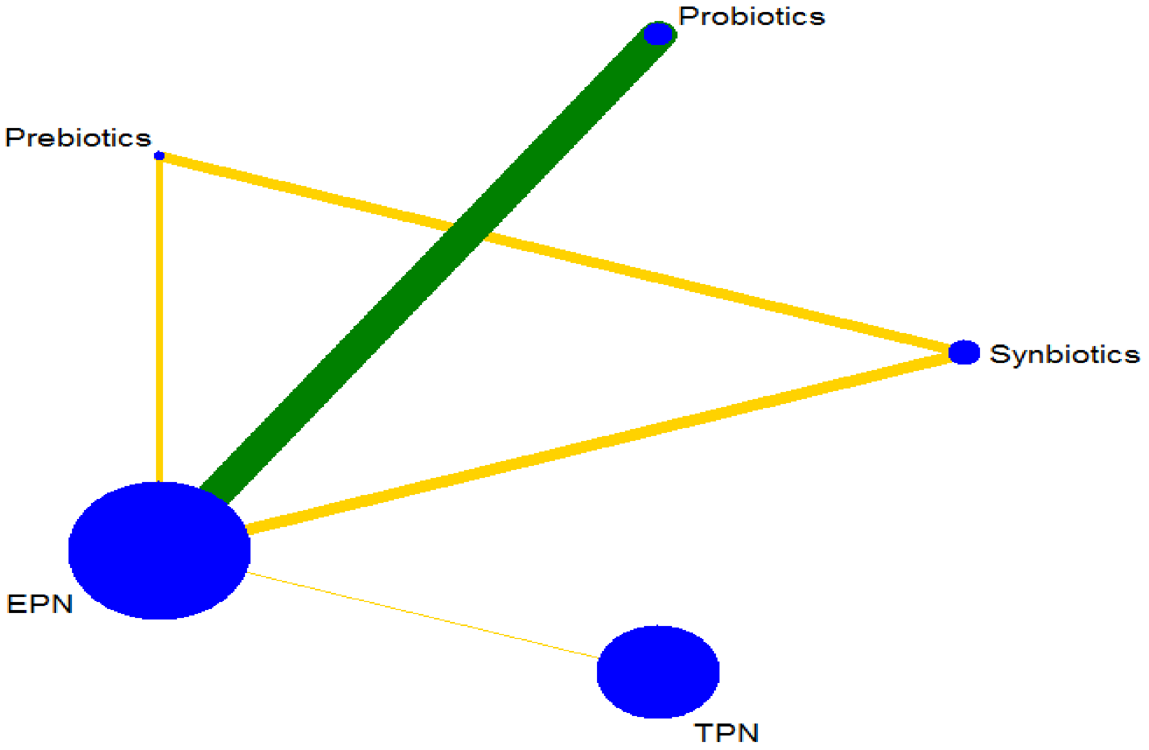

Figure S 6.11 Network plot of all intervention comparisons for hospital lengtn of stay

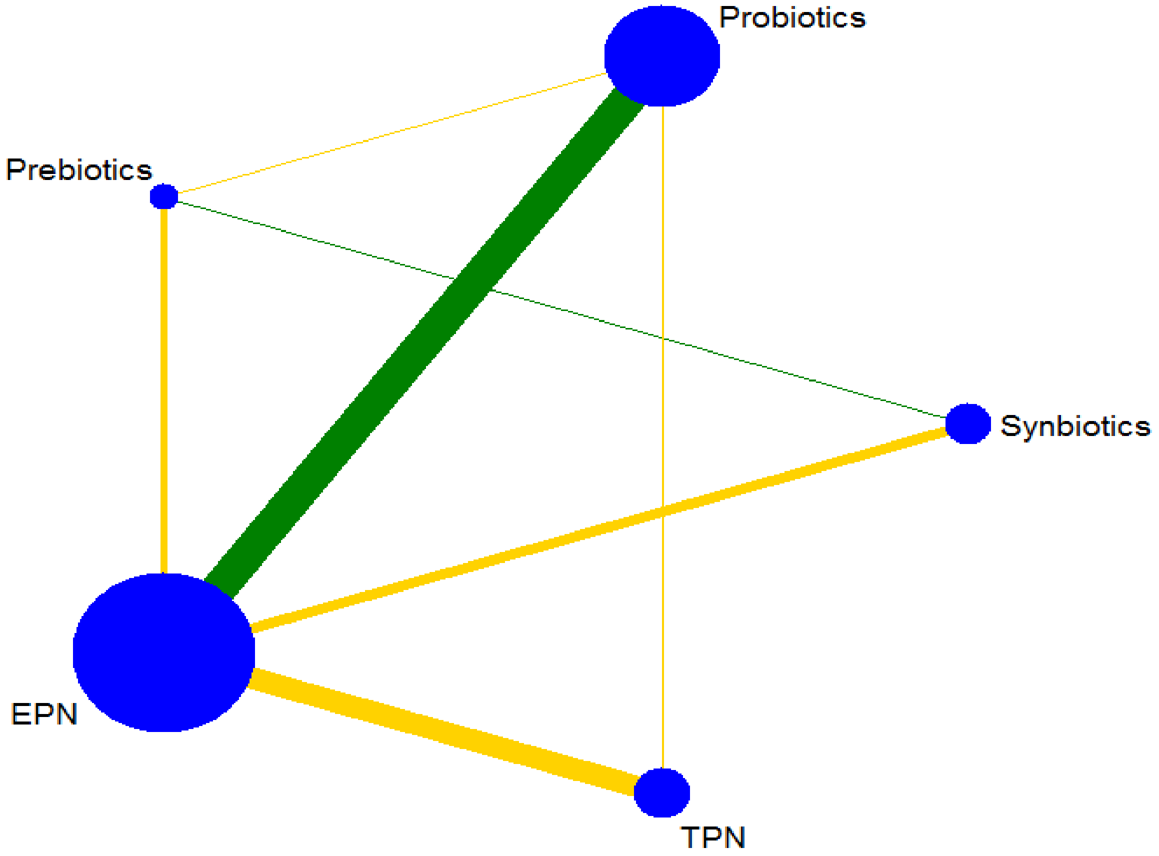

Figure S 6.12 Network plot of all intervention comparisons for ICU length of stay

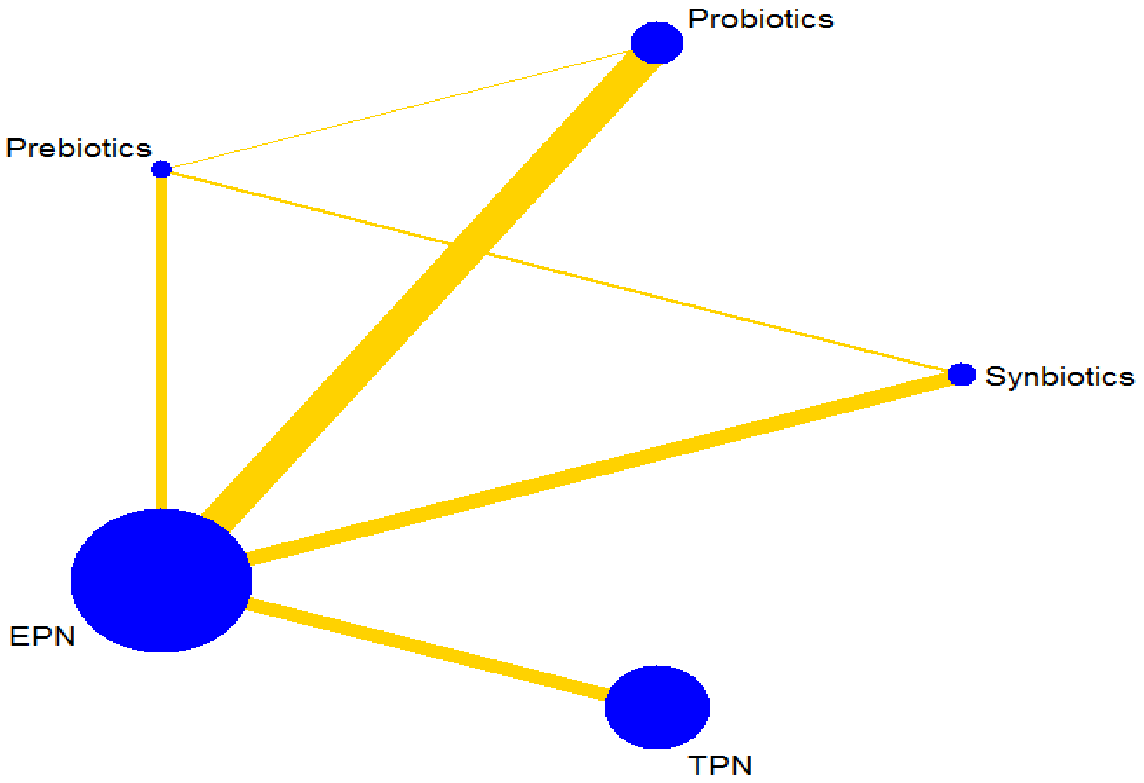

Figure S 6.13 Network plot of all intervention comparisons for the duration of mechanical ventilation

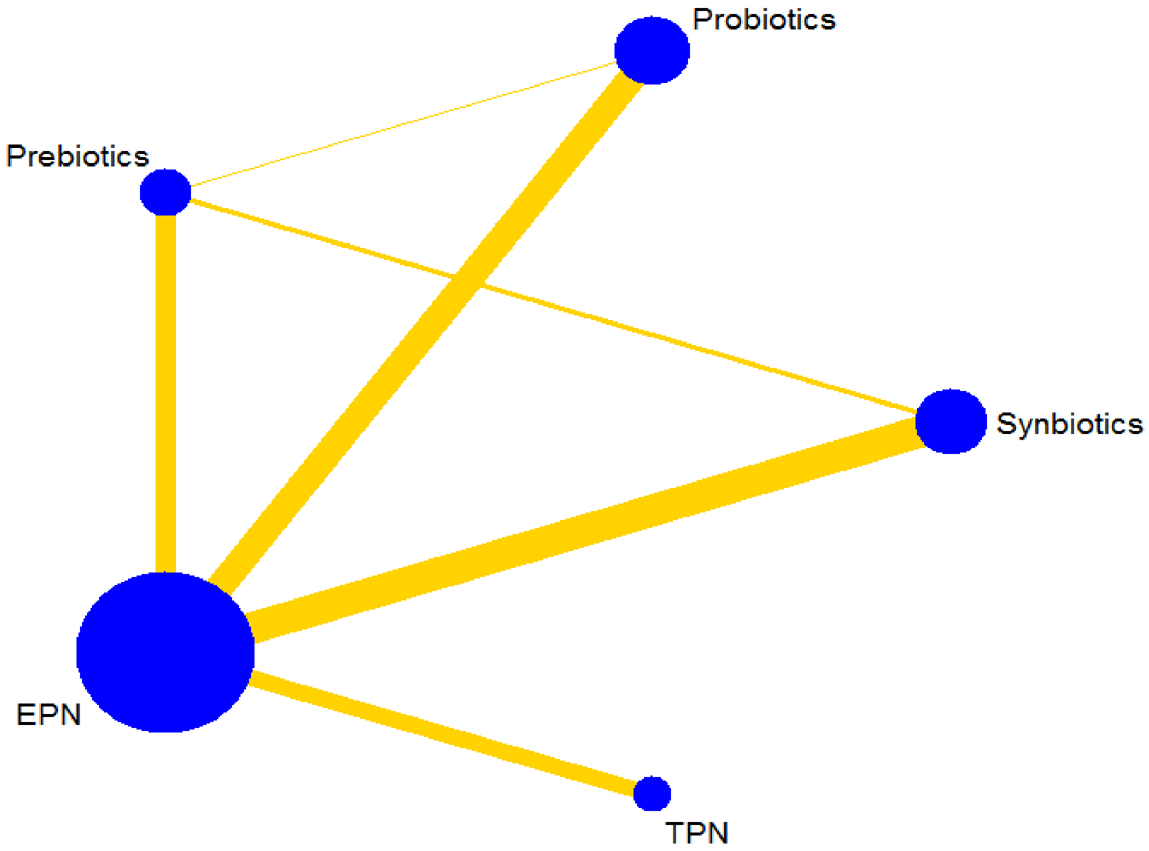

Appendix file 7
Assessment of heterogeneity in treatment for each outcome network

We assess heterogeneity between direct comparisons and global heterogeneity by looked at the I^2 weather it is high compared to the expected value (50%). The following graphs all show the results of heterogeneity between direct comparisons in each outcome network. The study number in the first column of each figure corresponds to the serial number of the references in appendix 15. A=Synbiotics, B=Probiotics, C= Probiotics, D= EPN, E= TPN.

Figure S 7.1 Assesment of heterogeneity in networks for nosocomial infection

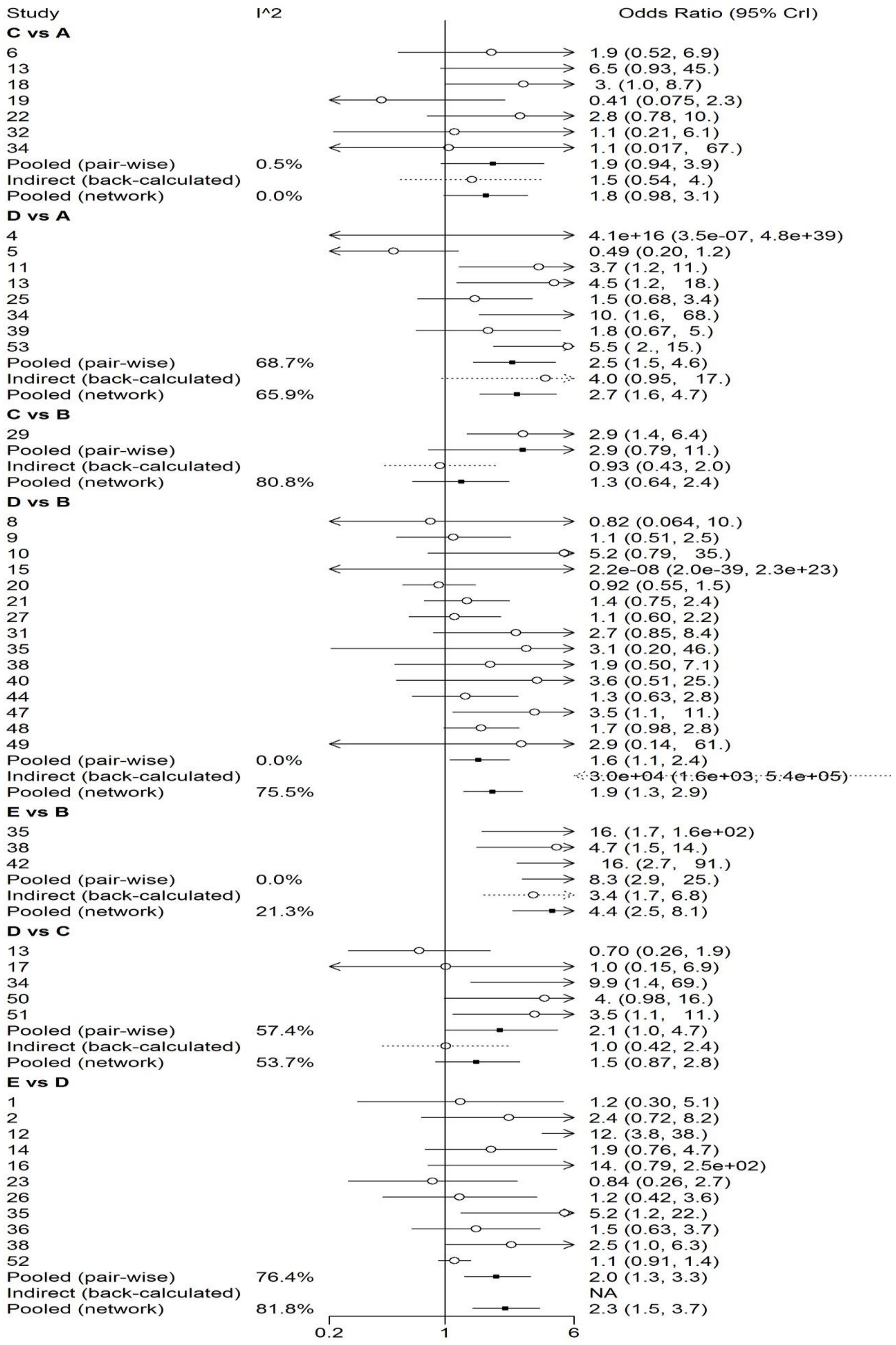

Figure S 7.2 Assesment of heterogeneity in networks for hospital acquired pneumonia

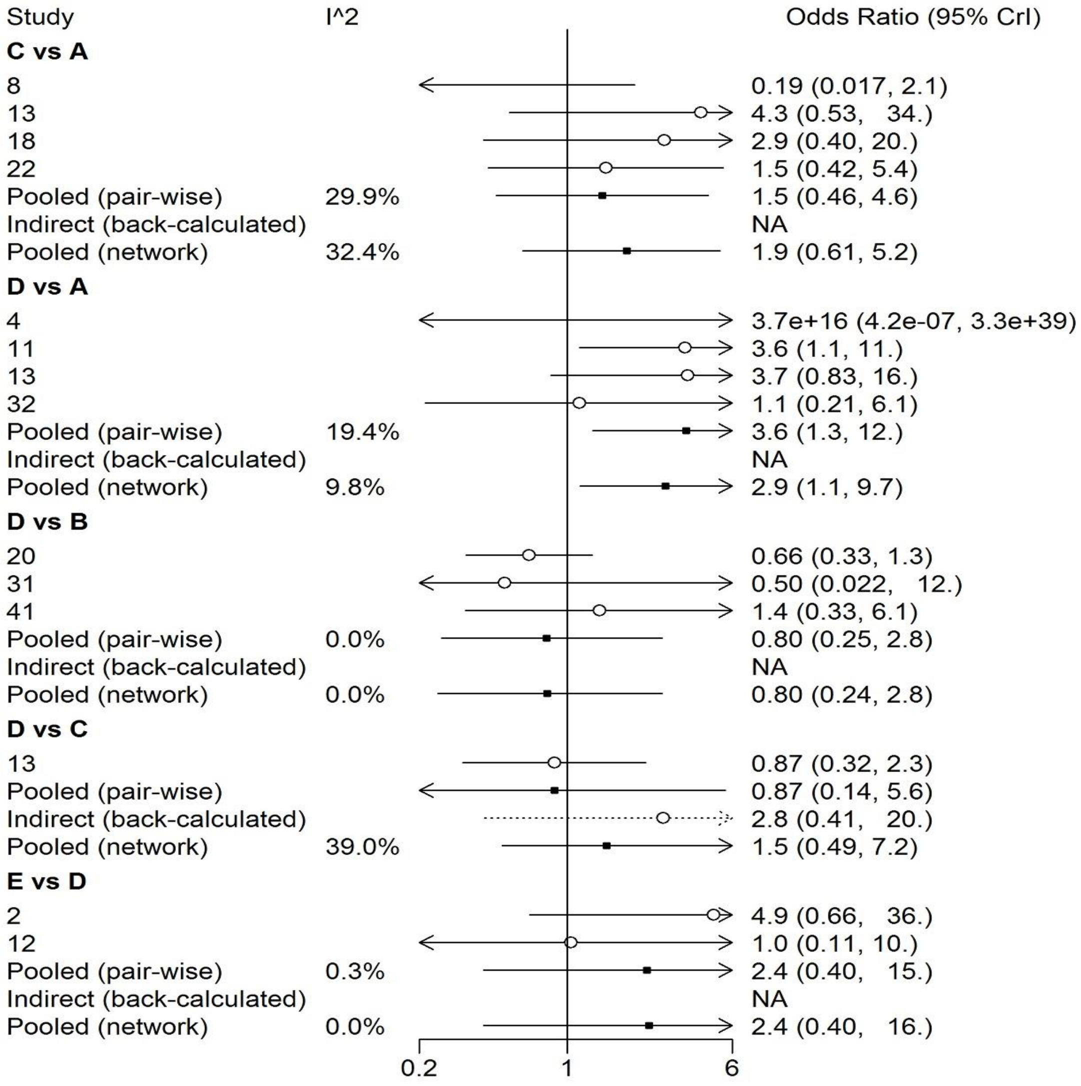

Figure S 7.3 Assesment of heterogeneity in networks for ventilator-associated pneumonia

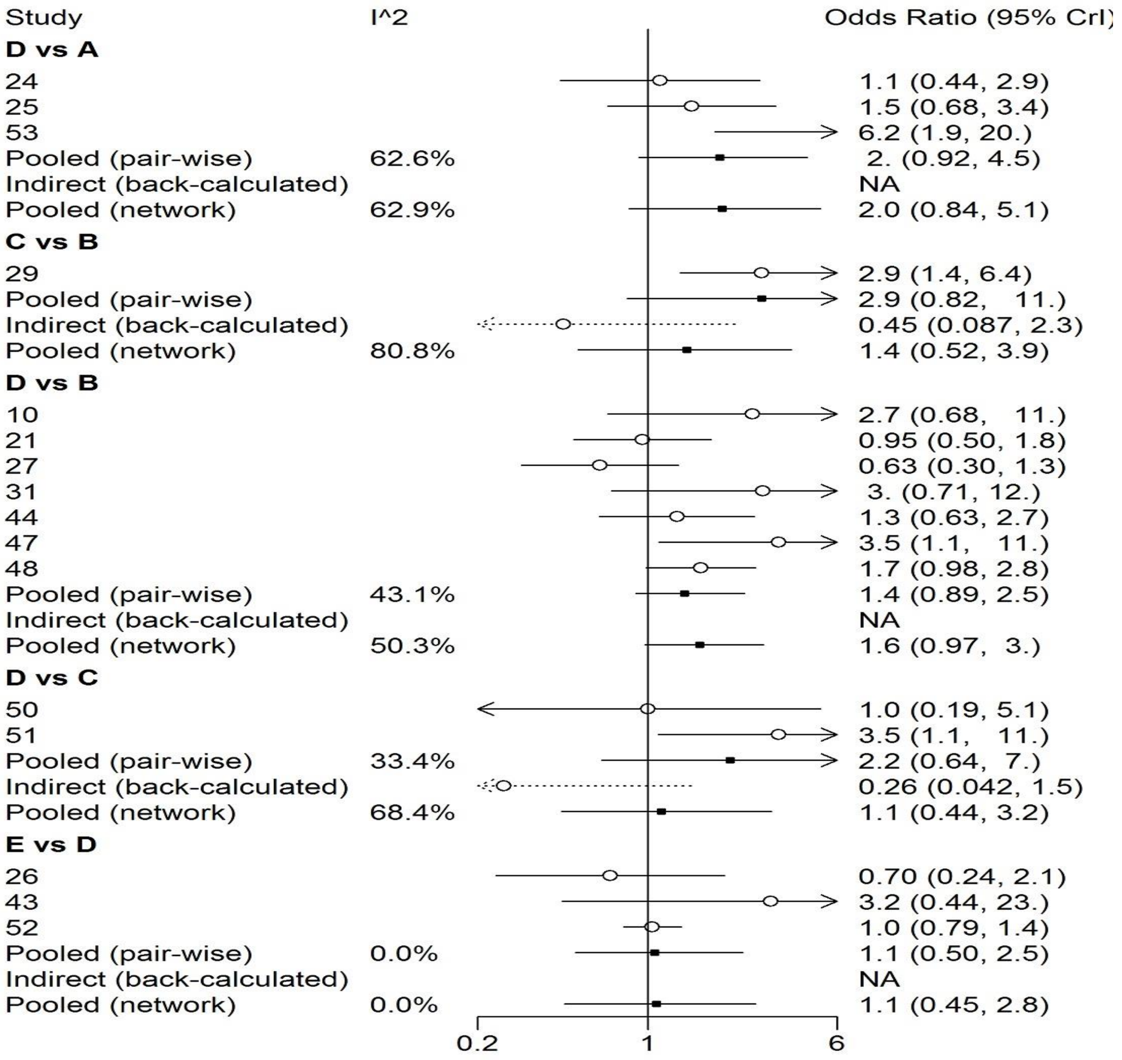

Figure S 7.4 Assesment of heterogeneity in networks for bloodstream infection

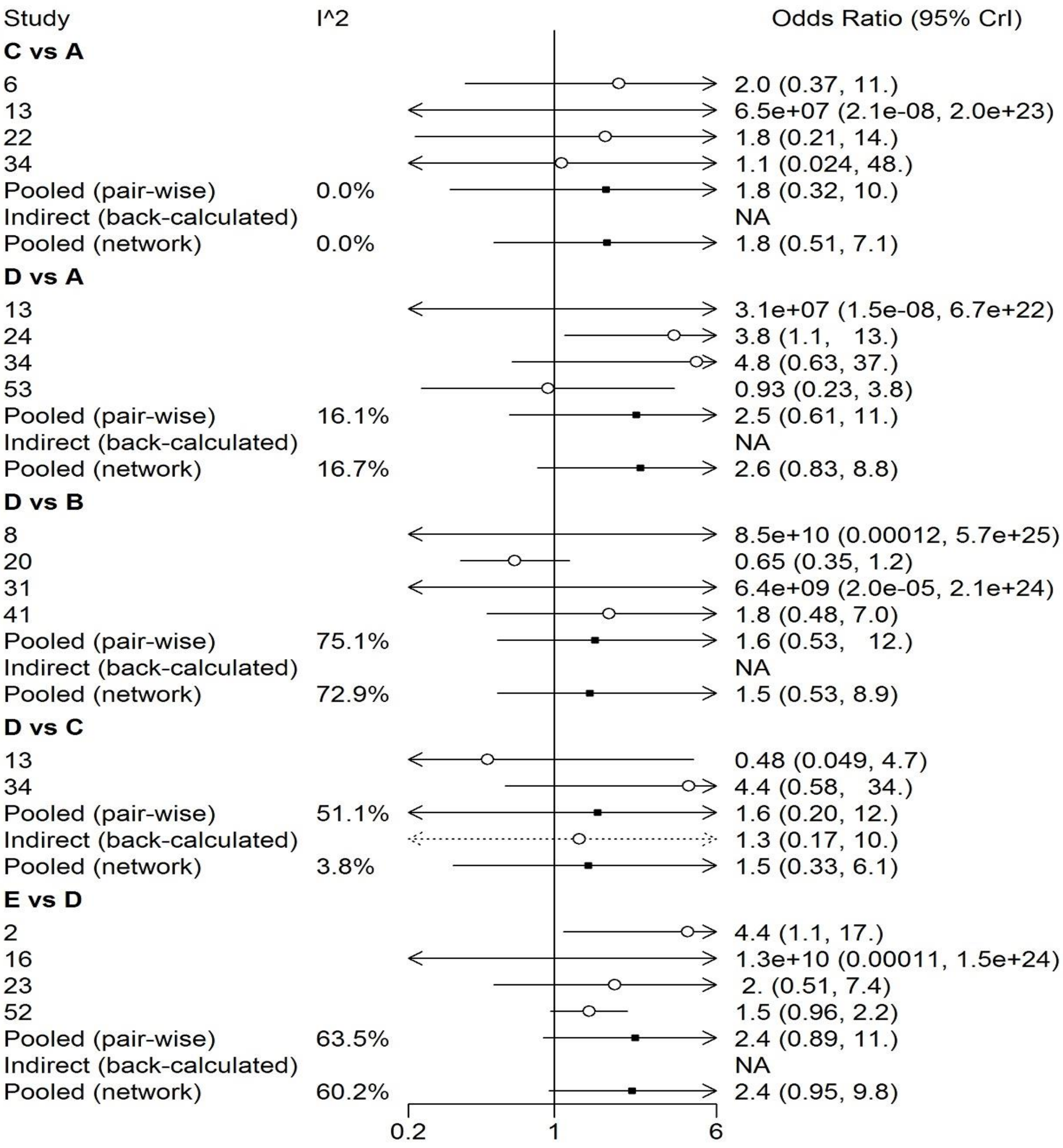

Figure S 7.5 Assesment of heterogeneity in networks for catheter-related bloodstream infection

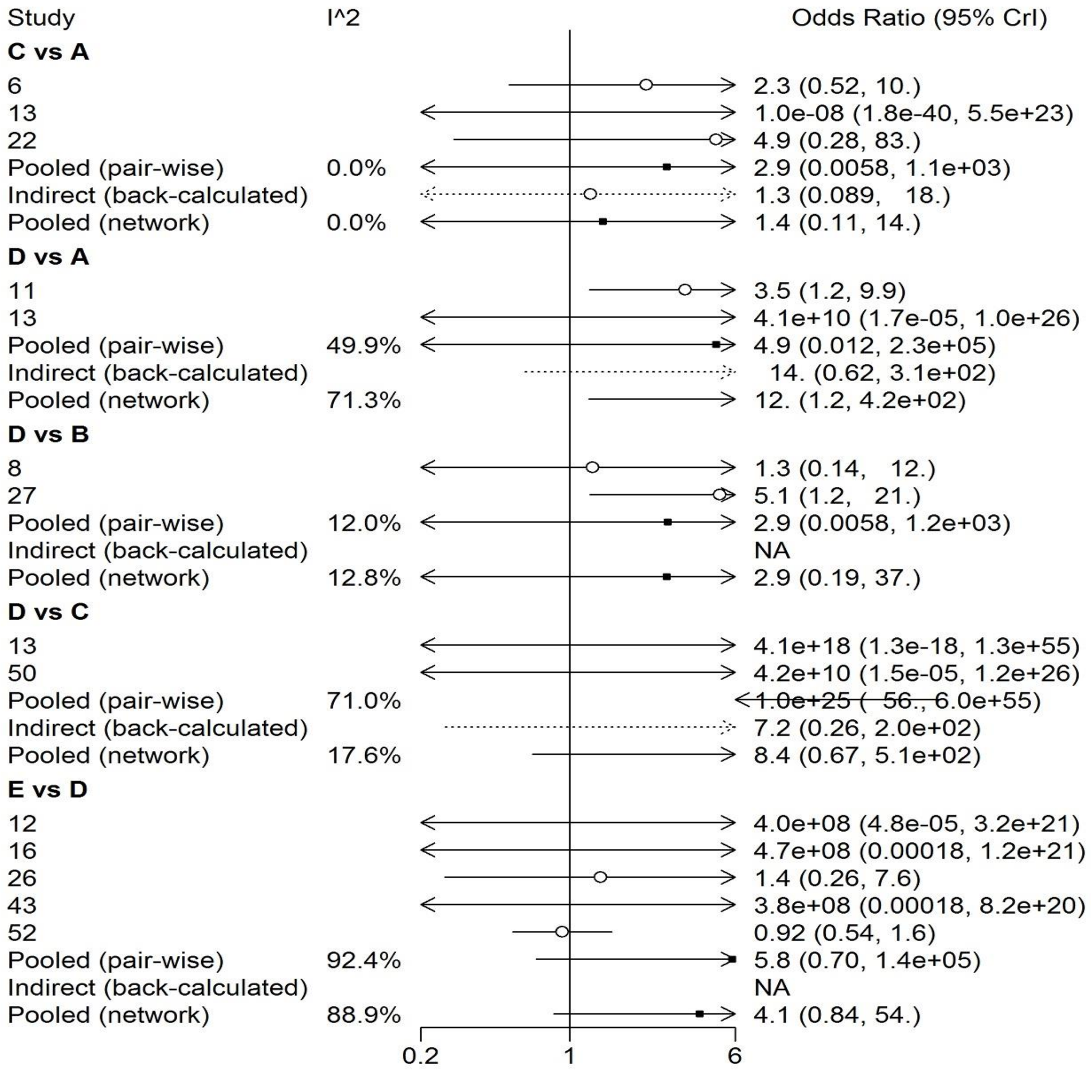

Figure S 7.6 Assesment of heterogeneity in networks for urinary tract infection

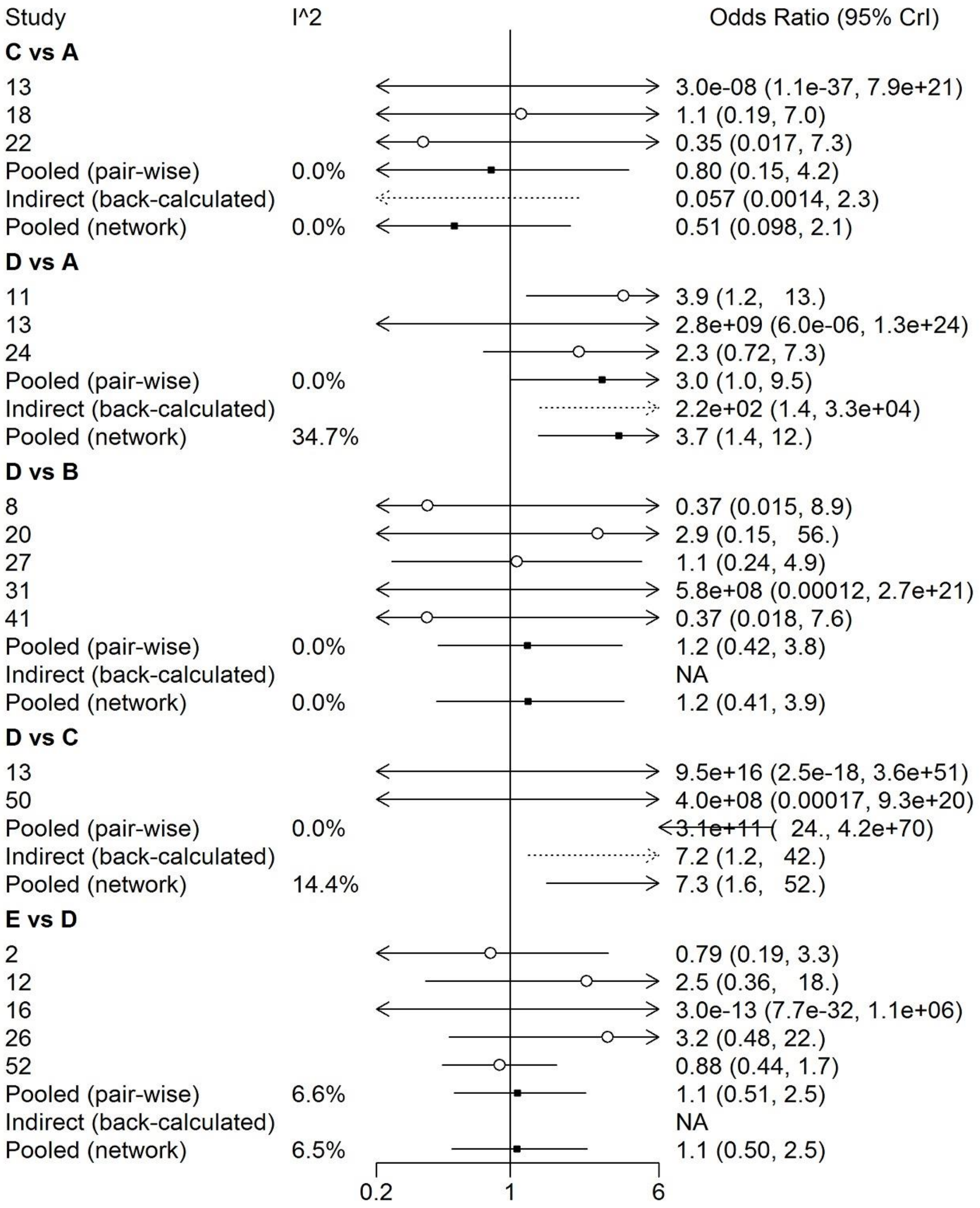

.

Figure S 7.7 Assesment of heterogeneity in networks for sepsis

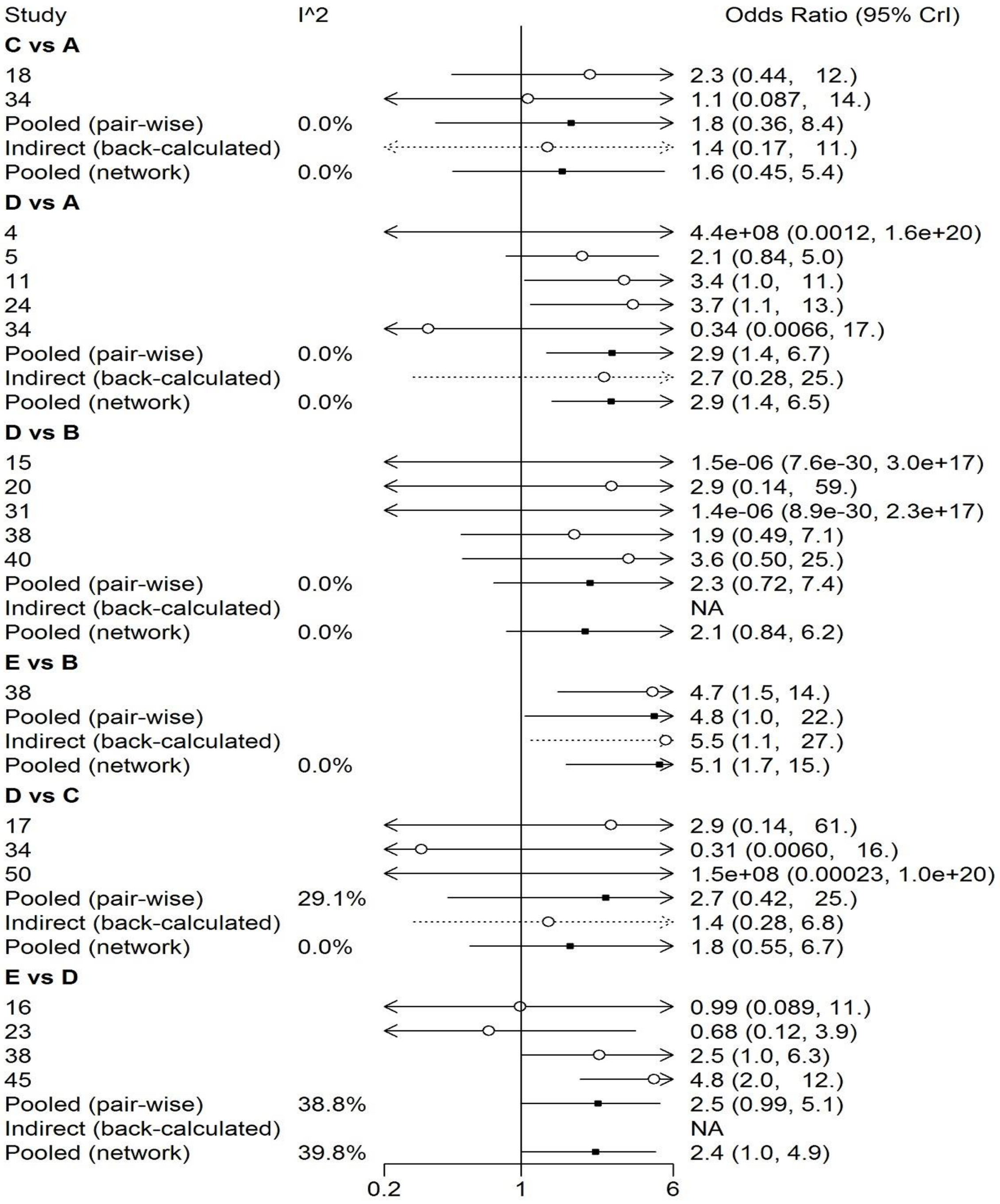

Figure S 7.8 Assesment of heterogeneity in networks for diarrhea

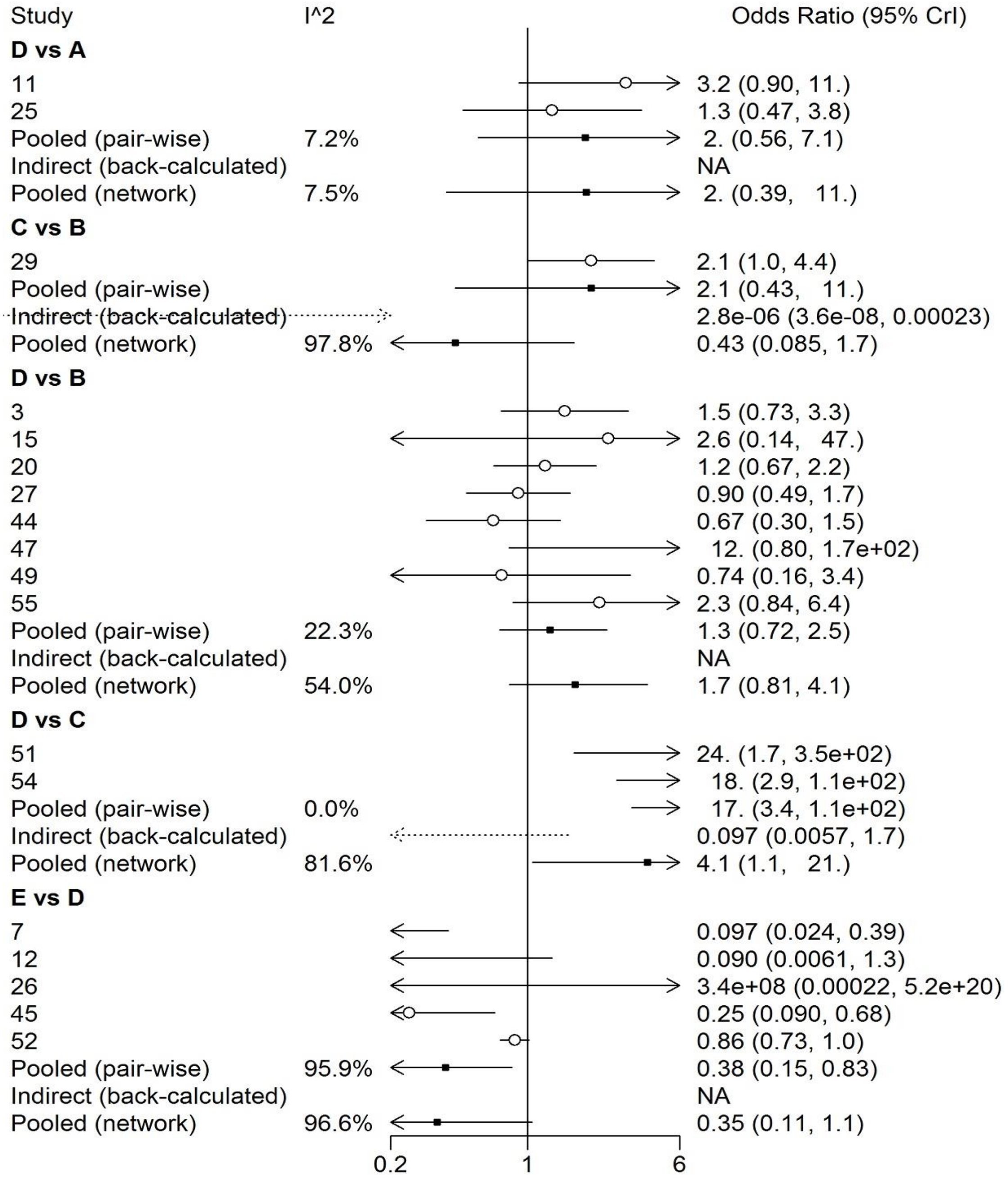

Figure S 7.9 Assesment of heterogeneity in networks for hospital mortality

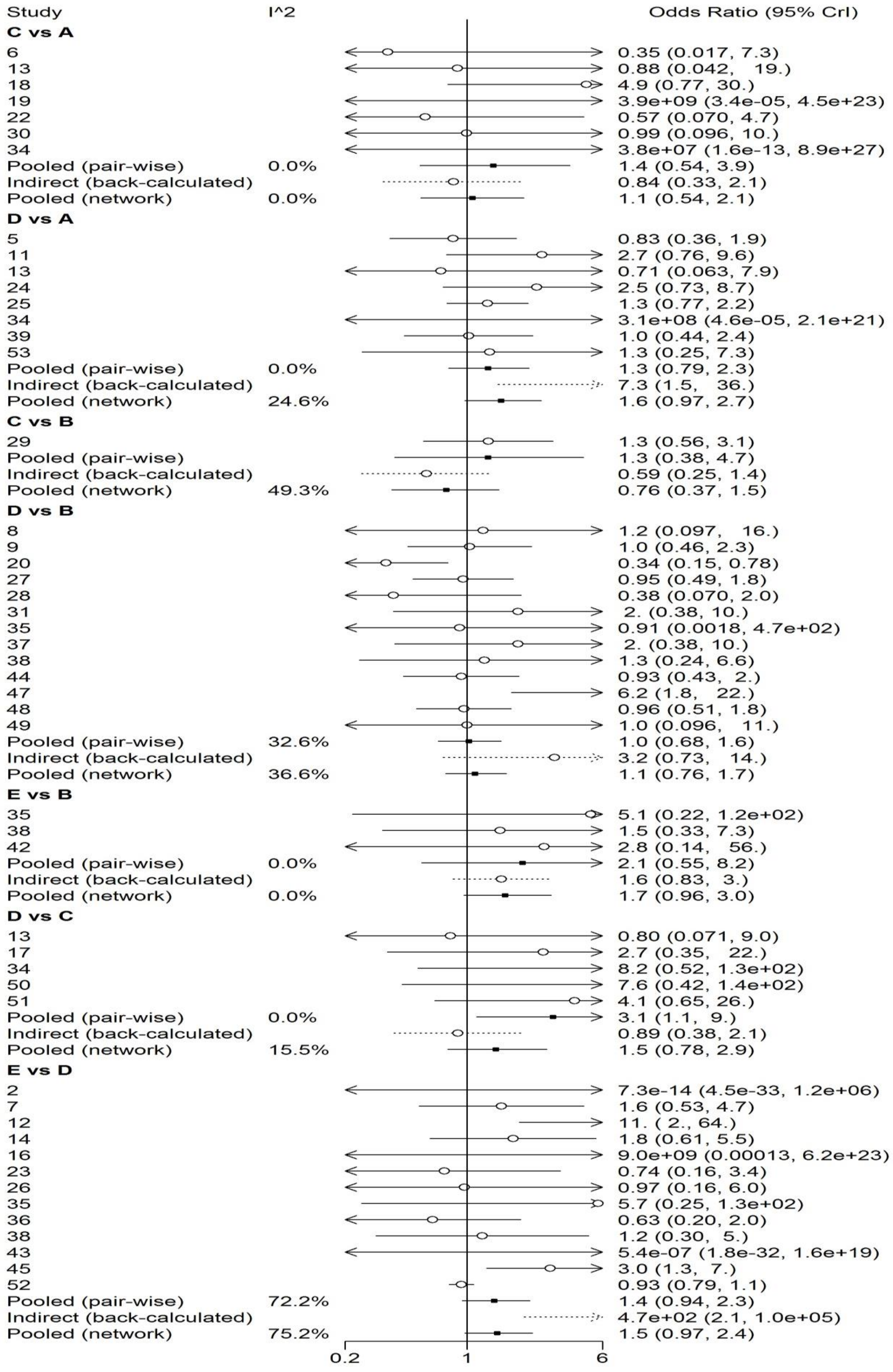

Figure S 7.10 Assesment of heterogeneity in networks for ICU mortality

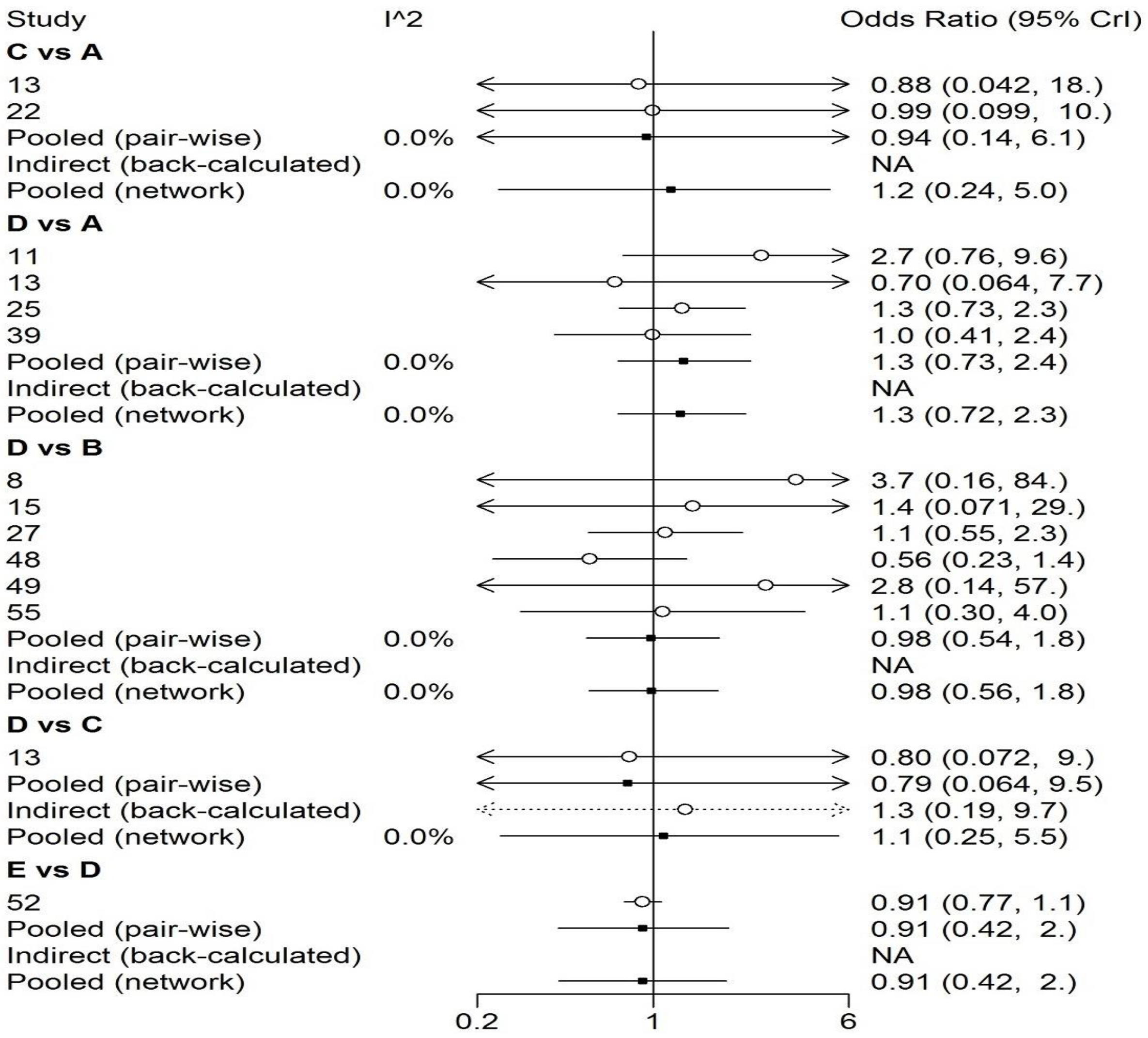

Figure S 7.11 Assesment of heterogeneity in networks for hospital lengtn of stay

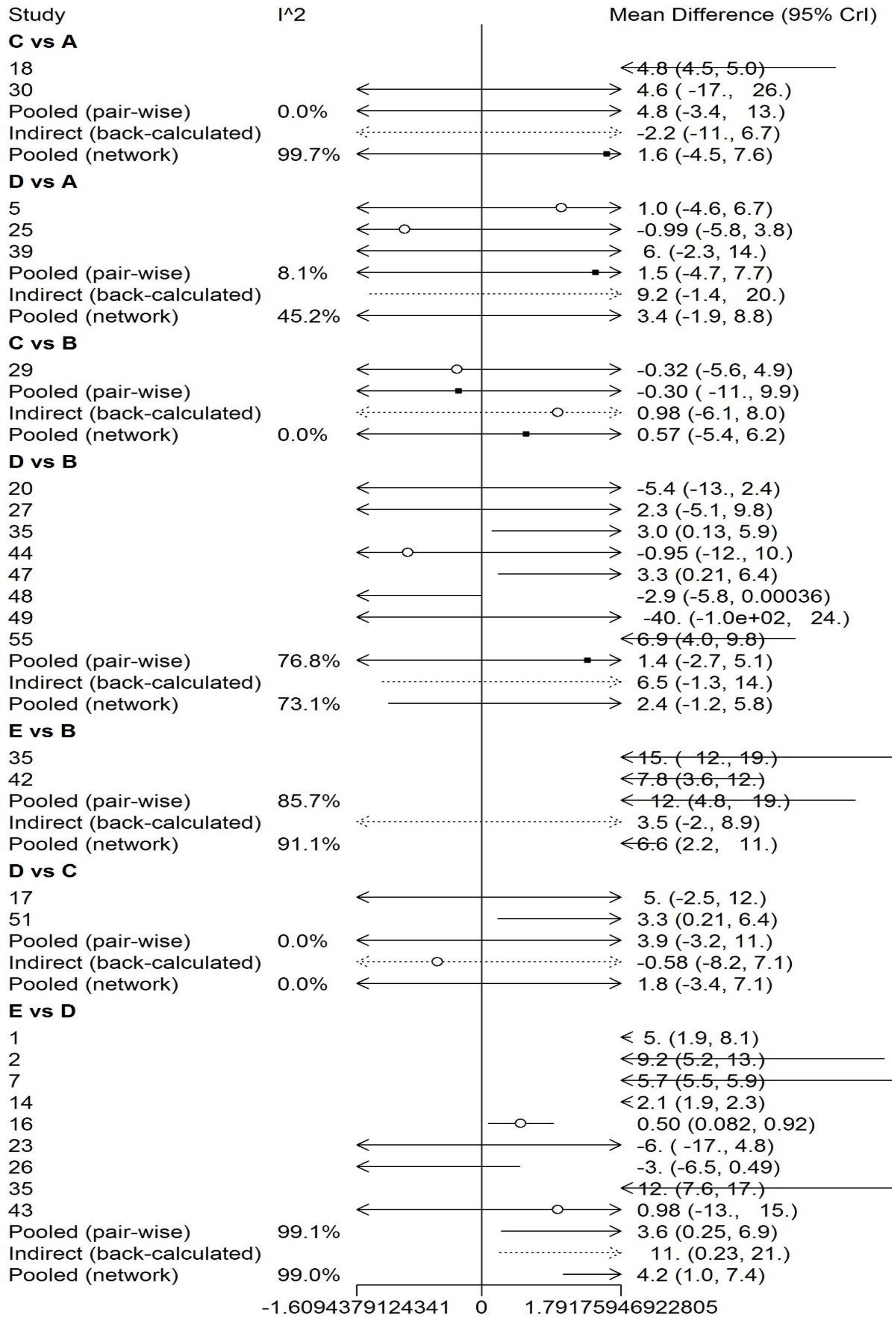

Figure S 7.12 Assesment of heterogeneity in networks for ICU length of stay

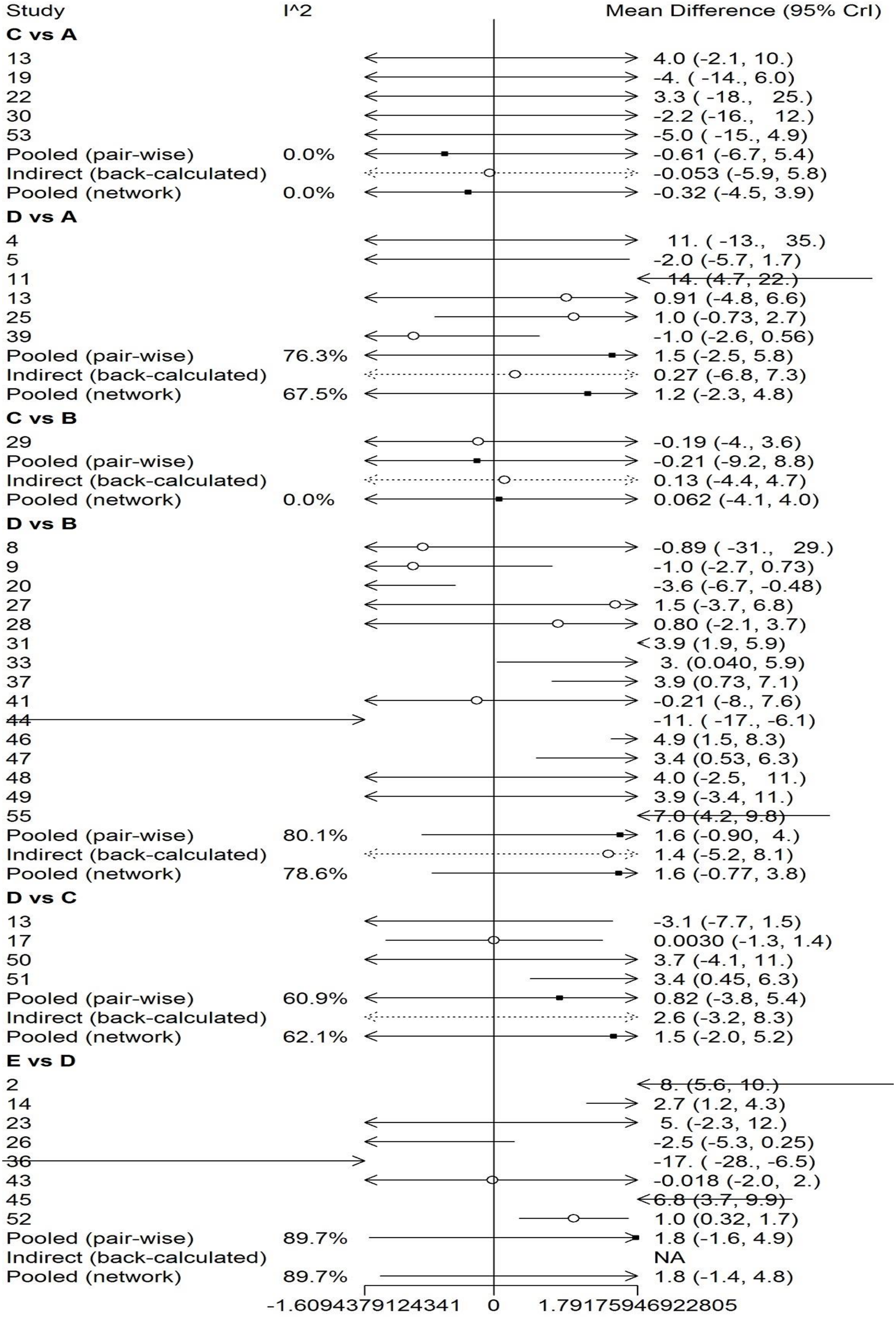

Figure S 7.13 Assesment of heterogeneity in networks for the duration of mechanical ventilation

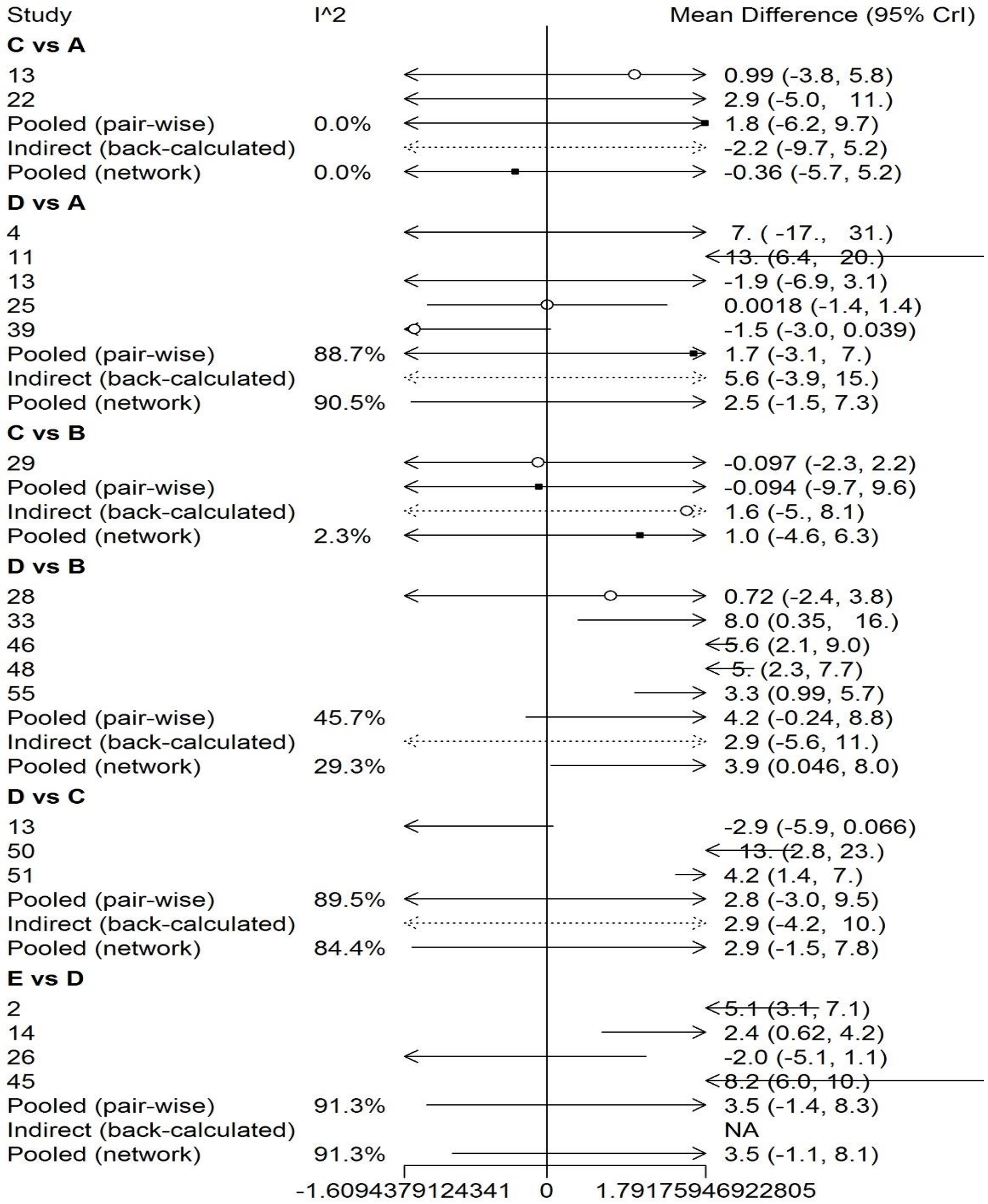

Table S 7.14 Assessment of global heterogeneity in networks

**Network outcome**	**Pair(I^2%)**	**Compairson(I^2%)**
Nosocomial infection	54.47	62.02
Hospital acquired pneumonia	9.72	8.97
Ventilator-associated pneumonia	42.67	54.33
Bloodstream infection	51.66	44.64
Catheter-related bloodstream infections	84.99	79.14
Urinary tract infections	0	0
Sepsis	0	0
Diarrhea	87.94	91.11
Hospital mortality	42.61	46.59
ICU mortality	0	0
Length of hospital stay	97.79	98.56
Length of ICU stay	80.62	79.47
Length of MV	85.52	86.10

Appendix file 8
Assessment of inconsistency in treatment for each outcome network

We use Node-splitting analysis to assess inconsistencies between direct and indirect comparisons, and‘design-by-treatment’ interaction model to assess global inconsistencies ($p<0.05$). The following graphs all show the results of Node-splitting analysis in each outcome network. A=Synbiotics, B=Probiotics, C= Probiotics, D= EPN, E= TPN.

Figure S 8.1 Node-splitting analysis of inconsistency in networks for nosocomial infection

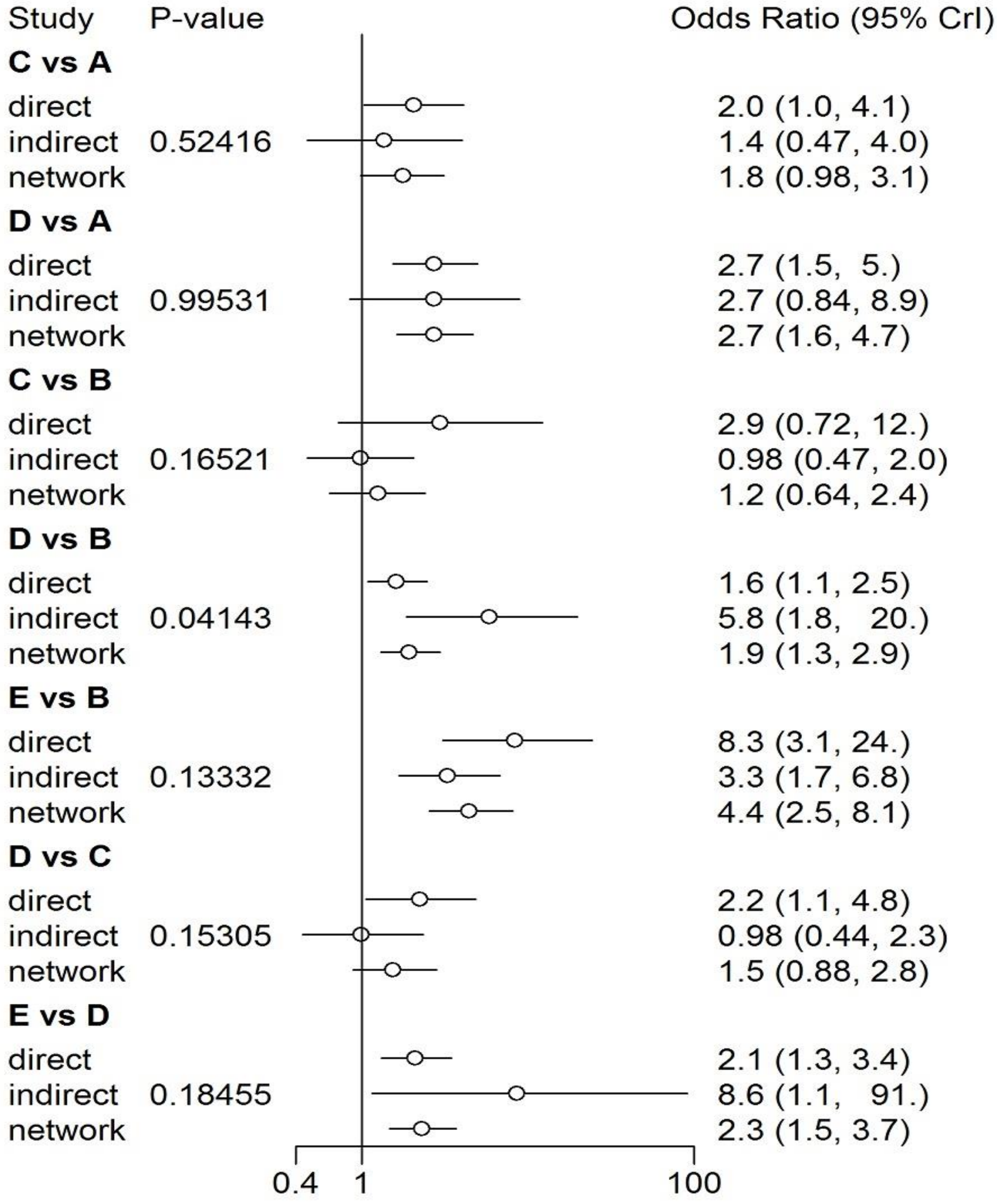

Figure S 8.2 Node-splitting analysis of inconsistency in networks for hospital acquired pneumonia

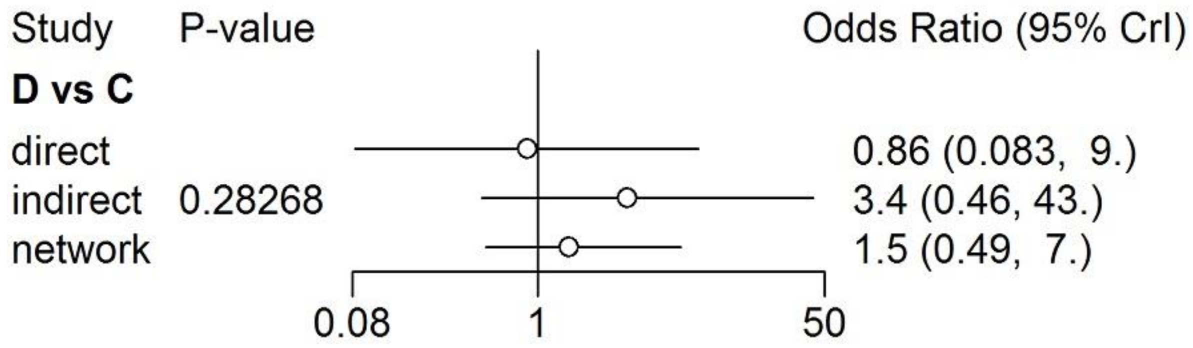

Figure S 8.3 Node-splitting analysis of inconsistency in networks for ventilator-associated pneumonia

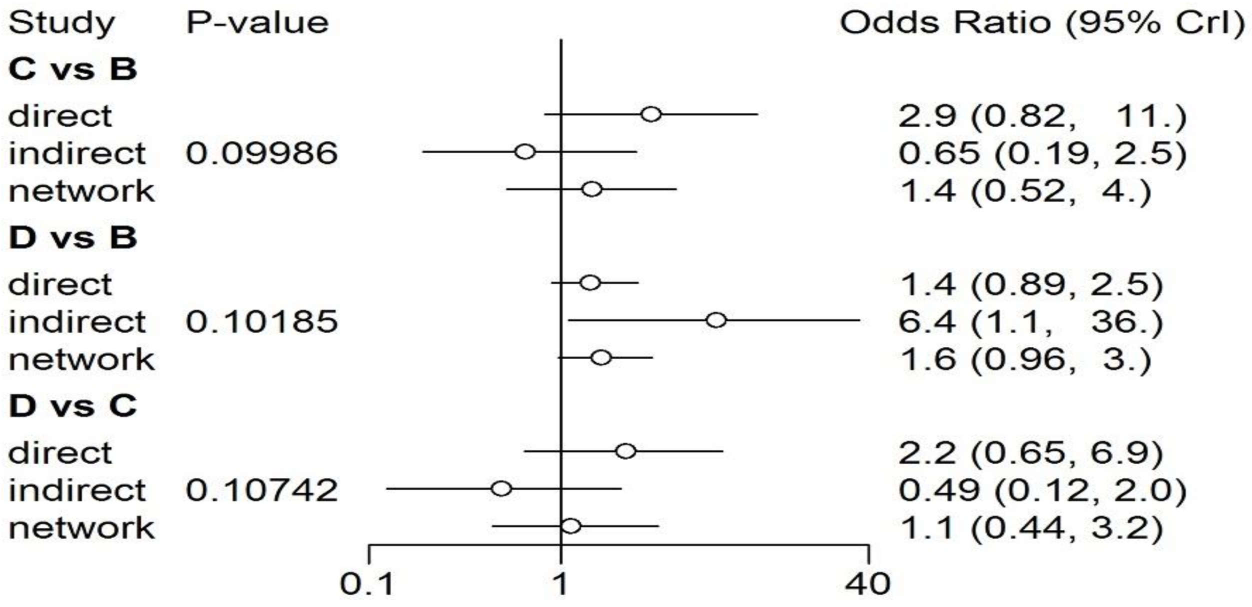

Figure S 8.4 Node-splitting analysis of inconsistency in networks for bloodstream infection

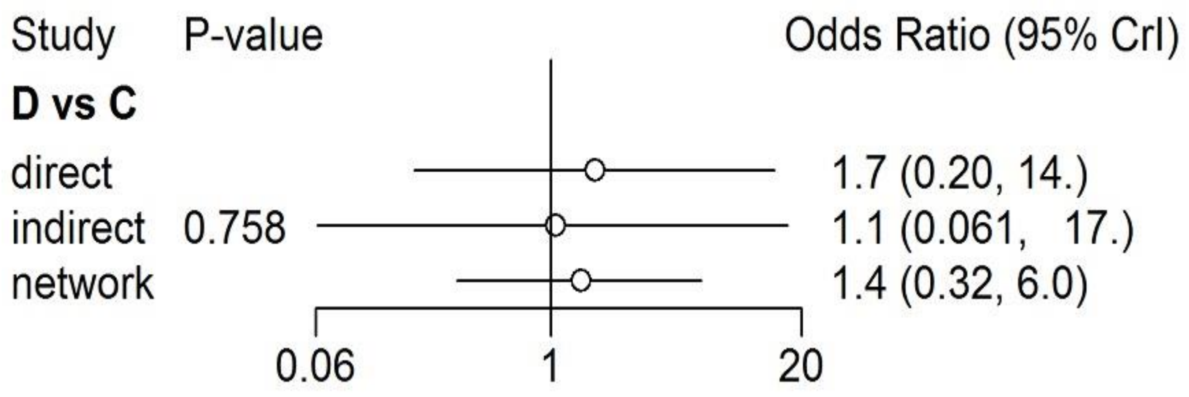

Figure S 8.5 Node-splitting analysis of inconsistency in networks for catheter-related bloodstream infection

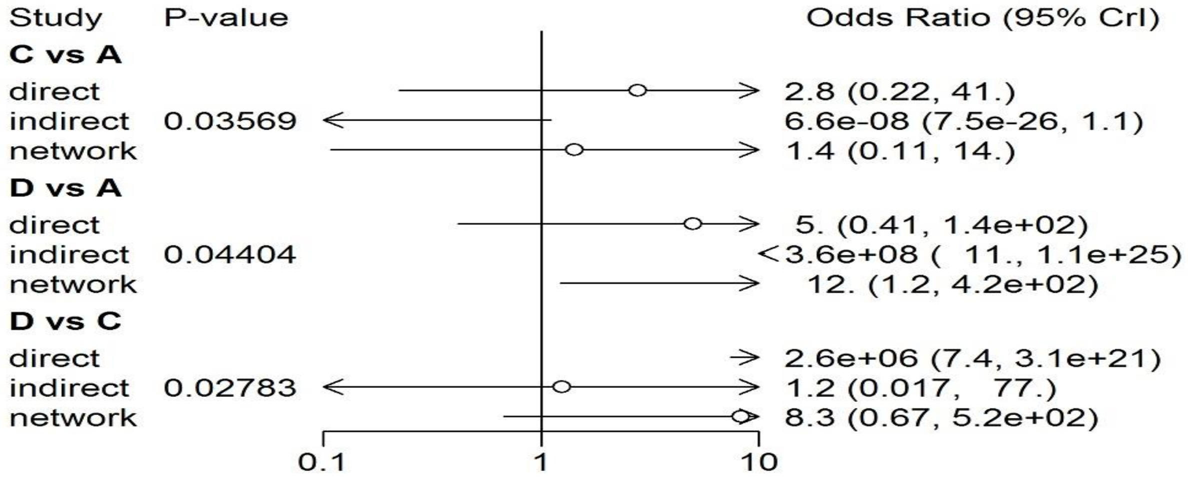

Figure S 8.6 Node-splitting analysis of inconsistency in networks for urinary tract infection

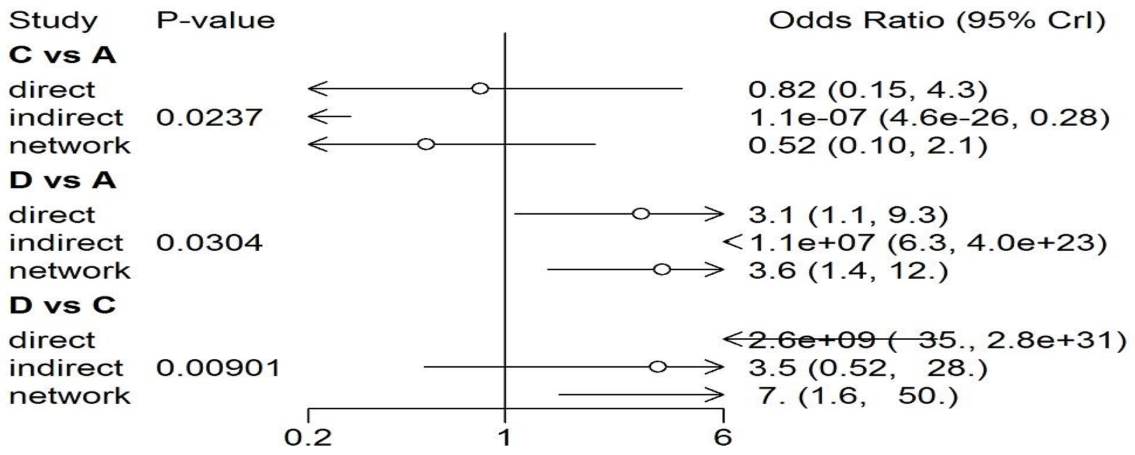

Figure S 8.7 Node-splitting analysis of inconsistency in networks for sepsis

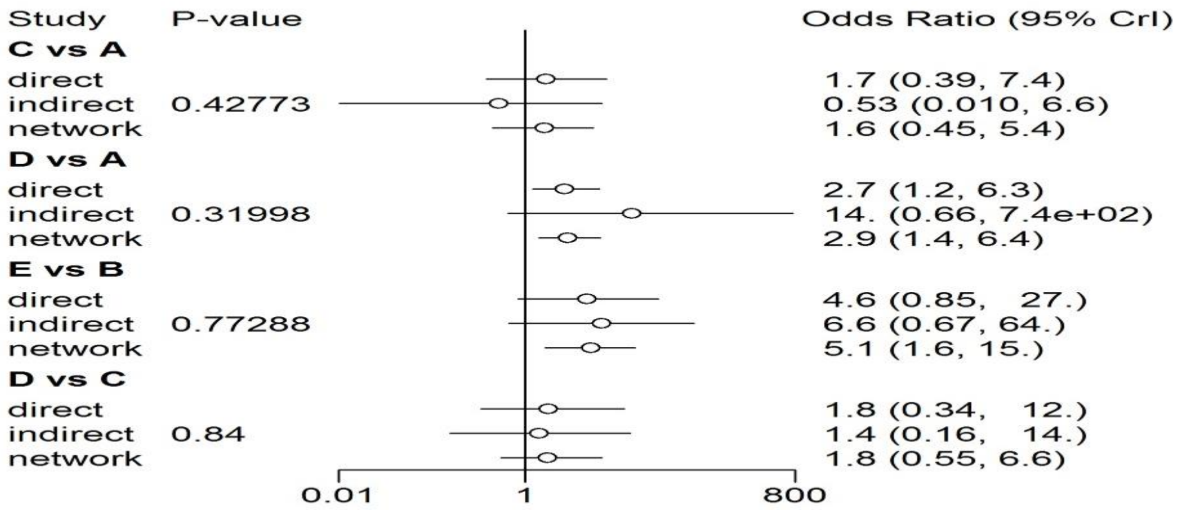

Figure S 8.8 Node-splitting analysis of inconsistency in networks for diarrhea

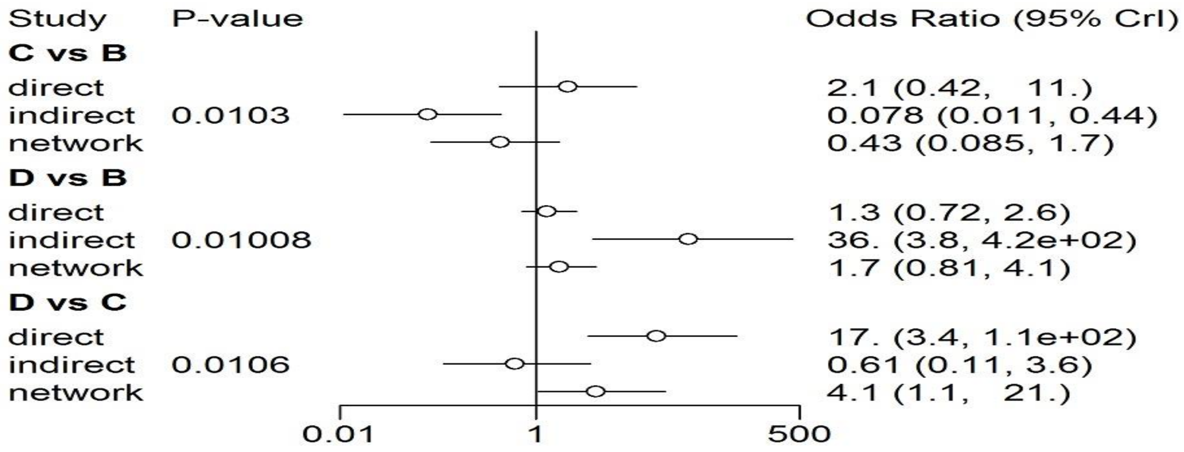

Figure S 8.9 Node-splitting analysis of inconsistency in networks for hospital mortality

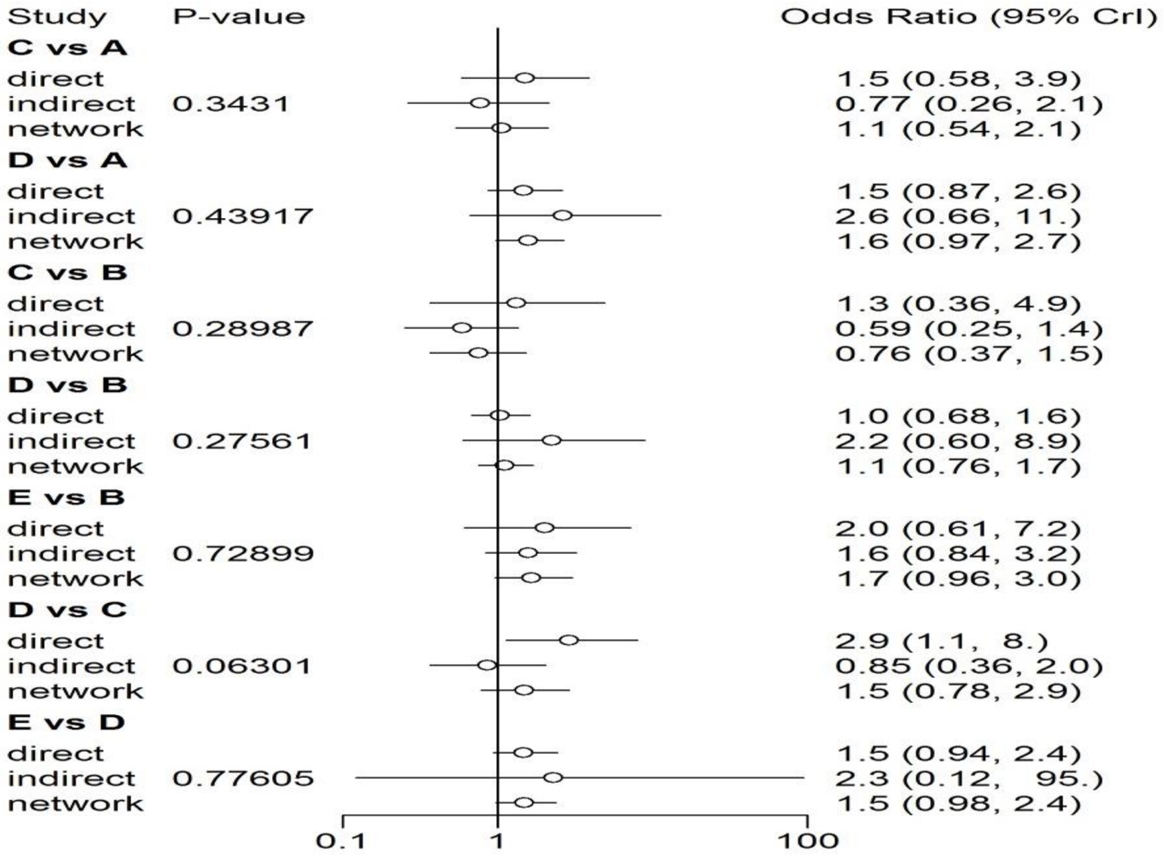

Figure S 8.10 Node-splitting analysis of inconsistency in networks for ICU mortality

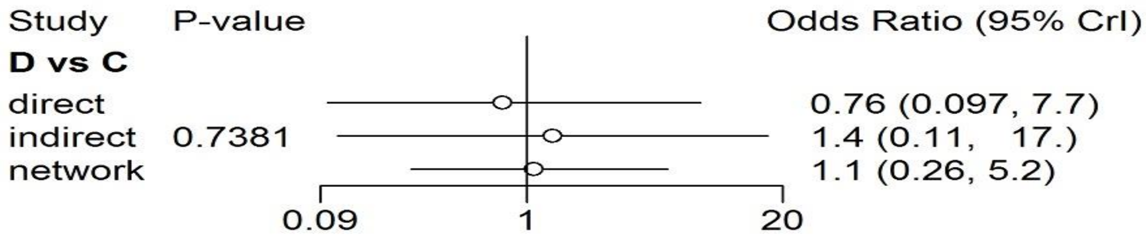

Figure S 8.11 Node-splitting analysis of inconsistency in networks for hospital lengtn of stay

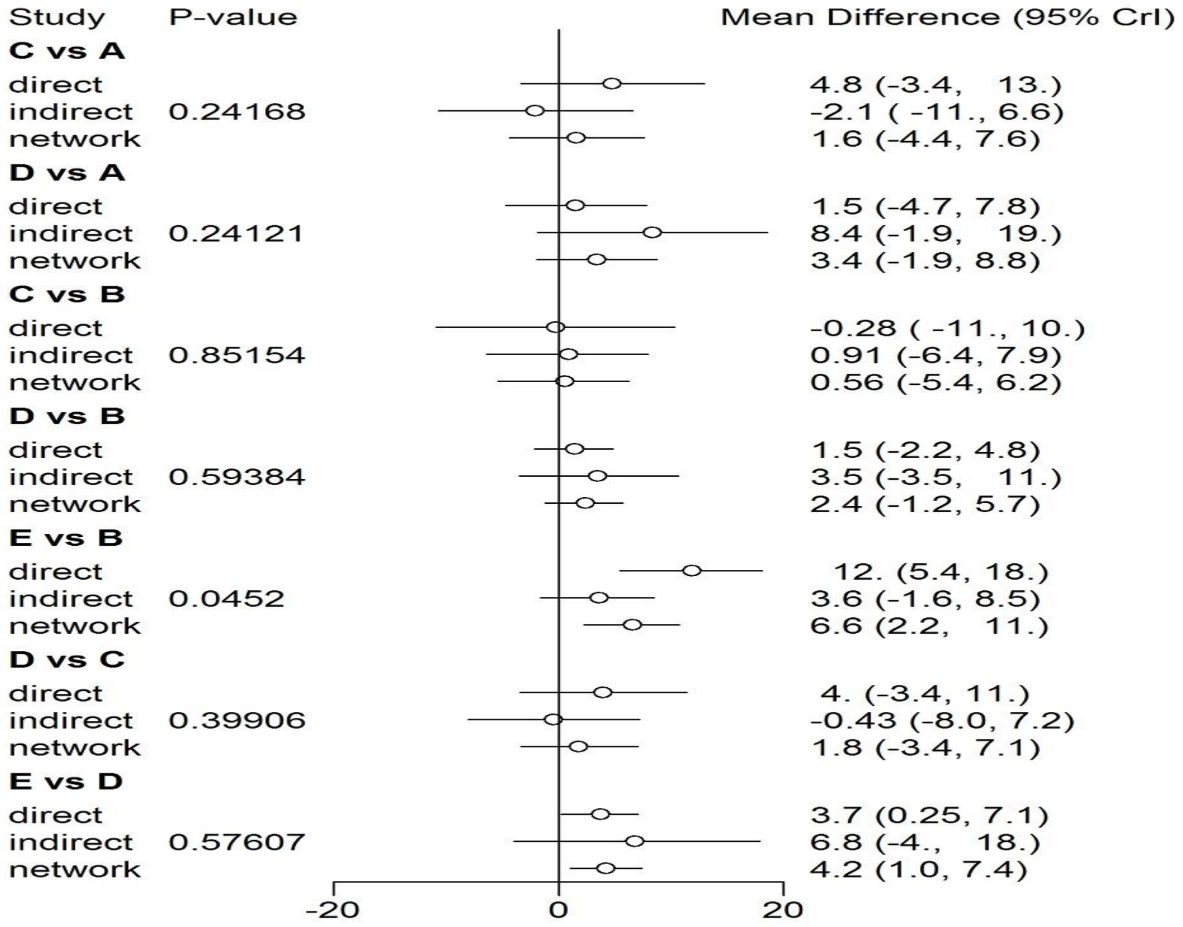

Figure S 8.12 Node-splitting analysis of inconsistency in networks for ICU length of stay

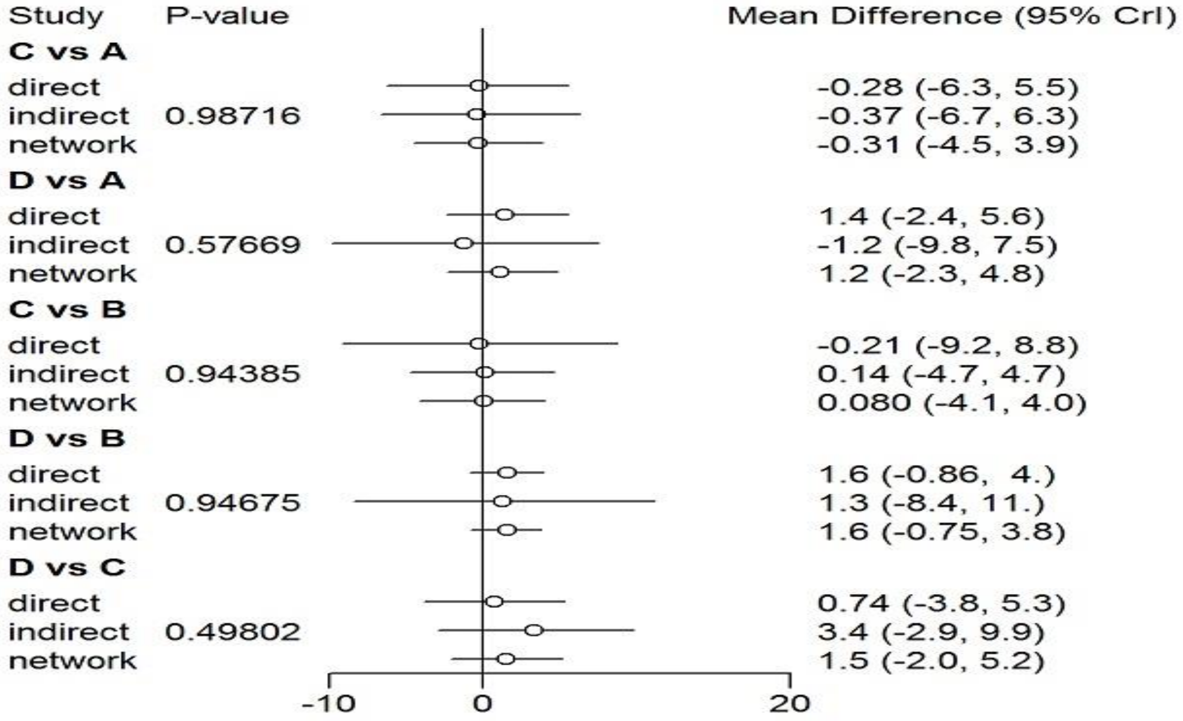

Figure S 8.13 Node-splitting analysis of inconsistency in networks for the duration of mechanical ventilation

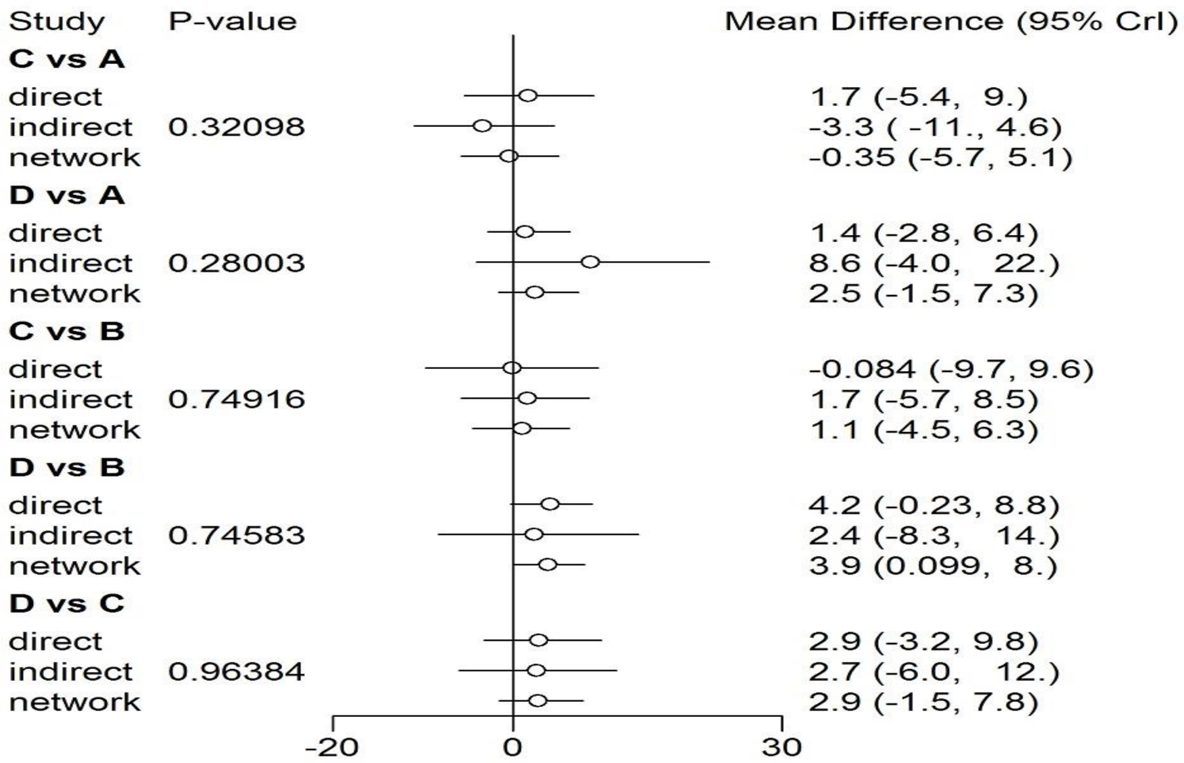

Figure S 8.14 Assesment of global inconsistency in networks using the 'design-by-treatment' interaction model

Network outcome	Chi-square	P value for test of global inconsistency
Nosocomial infection	11.26	0.1276
Hospital acquired pneumonia	2.29	0.3179
Ventilator-associated pneumonia	3.66	0.0557
Bloodstream infection	0.24	0.8872
Catheter-related bloodstream infections	1.05	0.7883
Urinary tract infections	1.76	0.6232
Sepsis	2.49	0.7774
Diarrhea	9.74	**0.0018**
Hospital mortality	3.81	0.8015
ICU mortality	0.58	0.7482
Length of hospital stay	9.83	0.0801
Length of ICU stay	2.01	0.7344
Length of MV	3.66	0.4543

Appendix 9

Assessment of transitivity

Figure S 9.1 Transitivity in the network of primary outcome

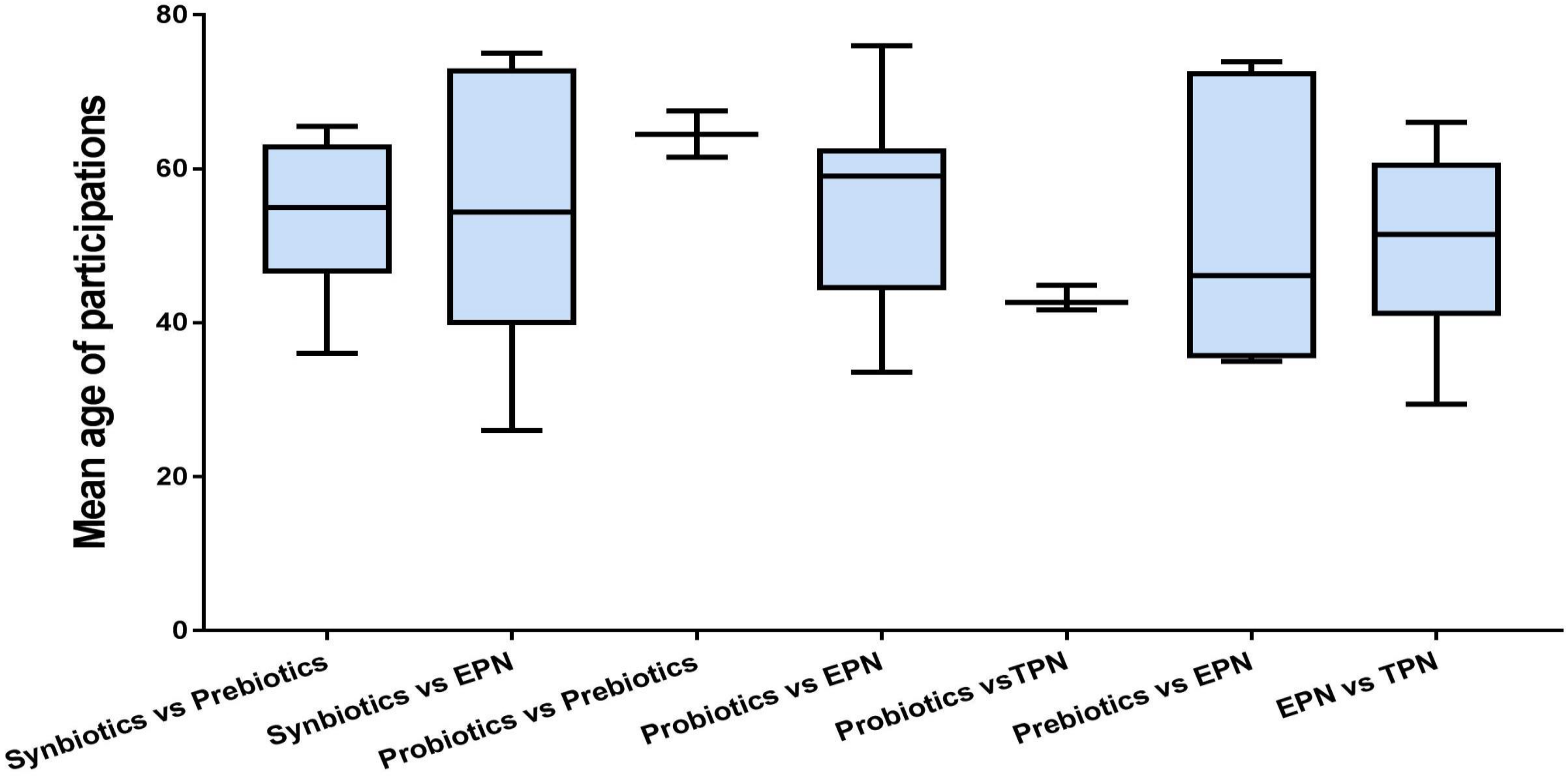

Appendix file 10

Comparison-adjusted funnel plot for each outcome form the network meta-analyses

Figure S 10.1 Comparison-adjusted funnel plot for the network of nosocomial infection in all comparisons

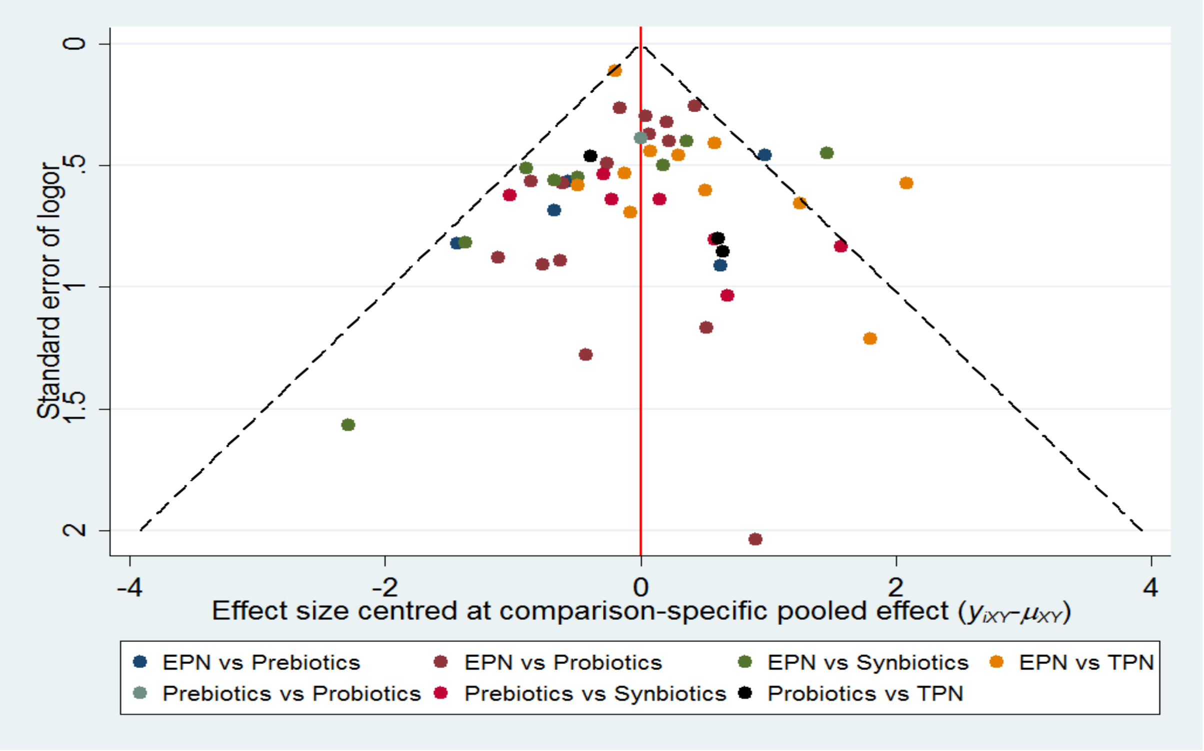

Figure S10.2 Comparison-adjusted funnel plot for the network of hospital acquired pneumonia in all comparisons

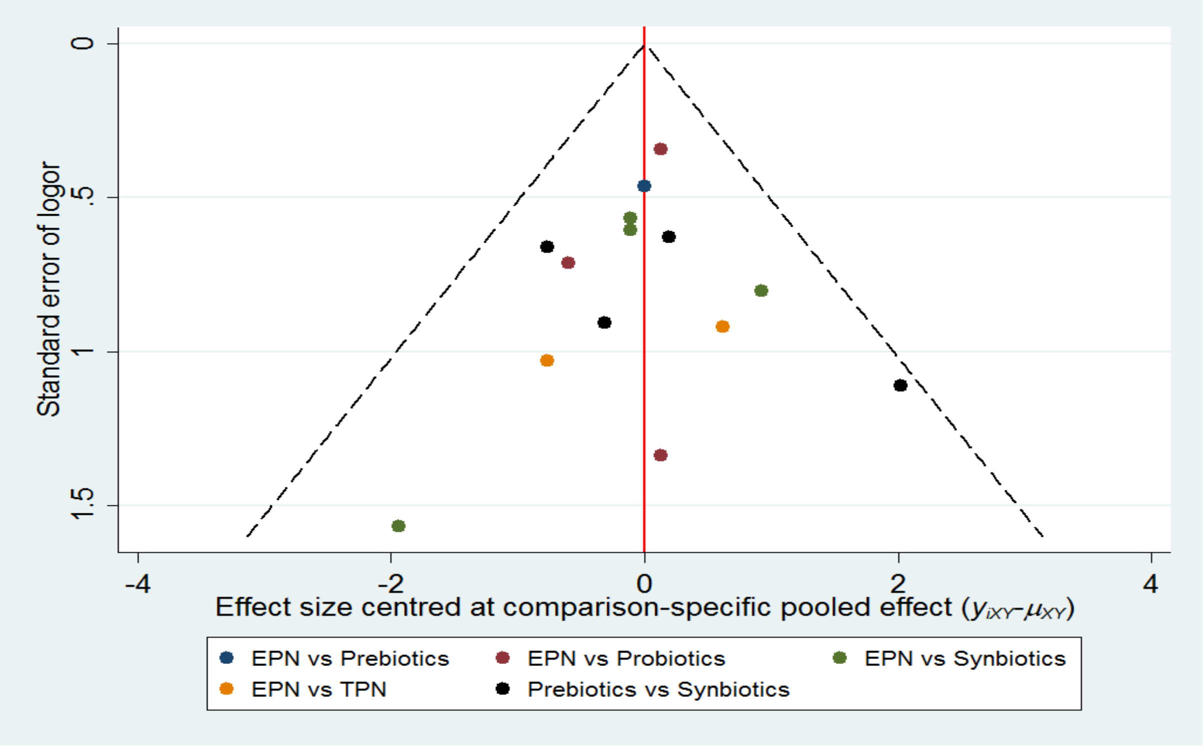

Figure S 10.3 Comparison-adjusted funnel plot for the network of ventilator-associated pneumonia in all comparisons

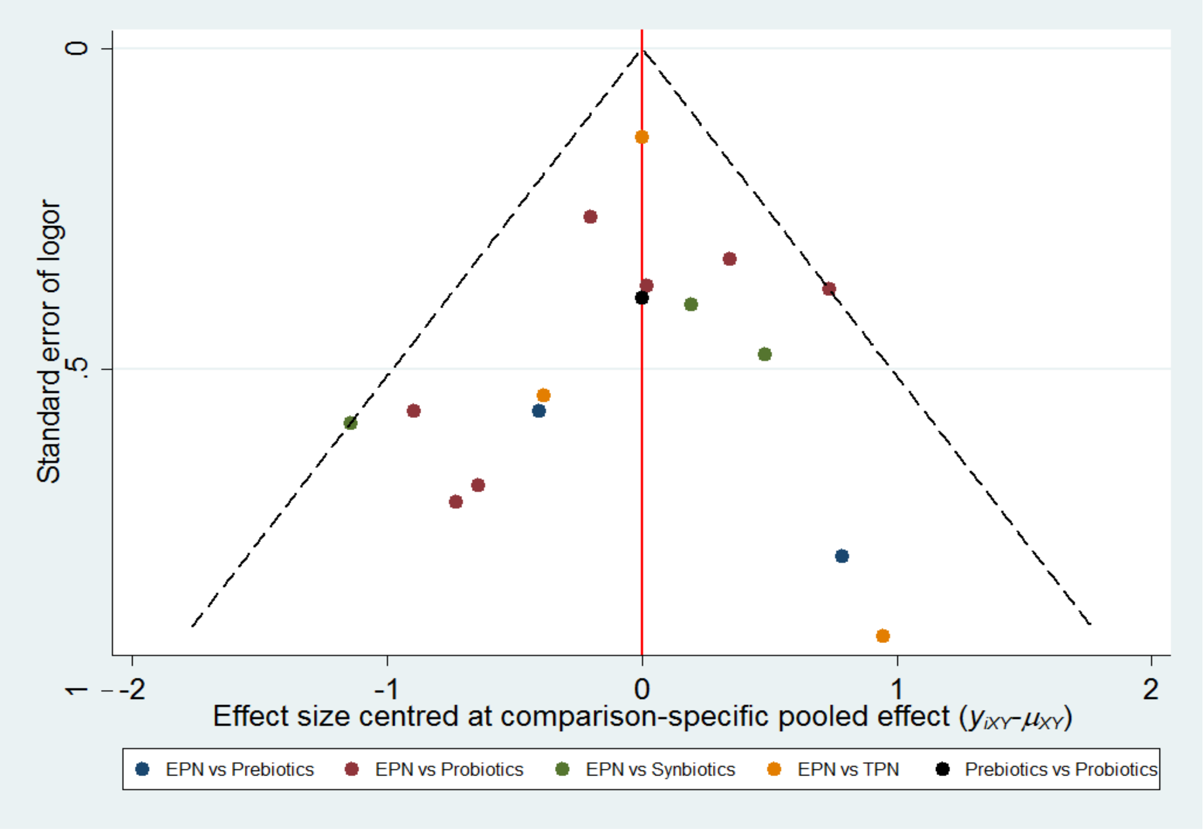

Figure S 10.4 Comparison-adjusted funnel plot for the network of bloodstream infections in all comparisons

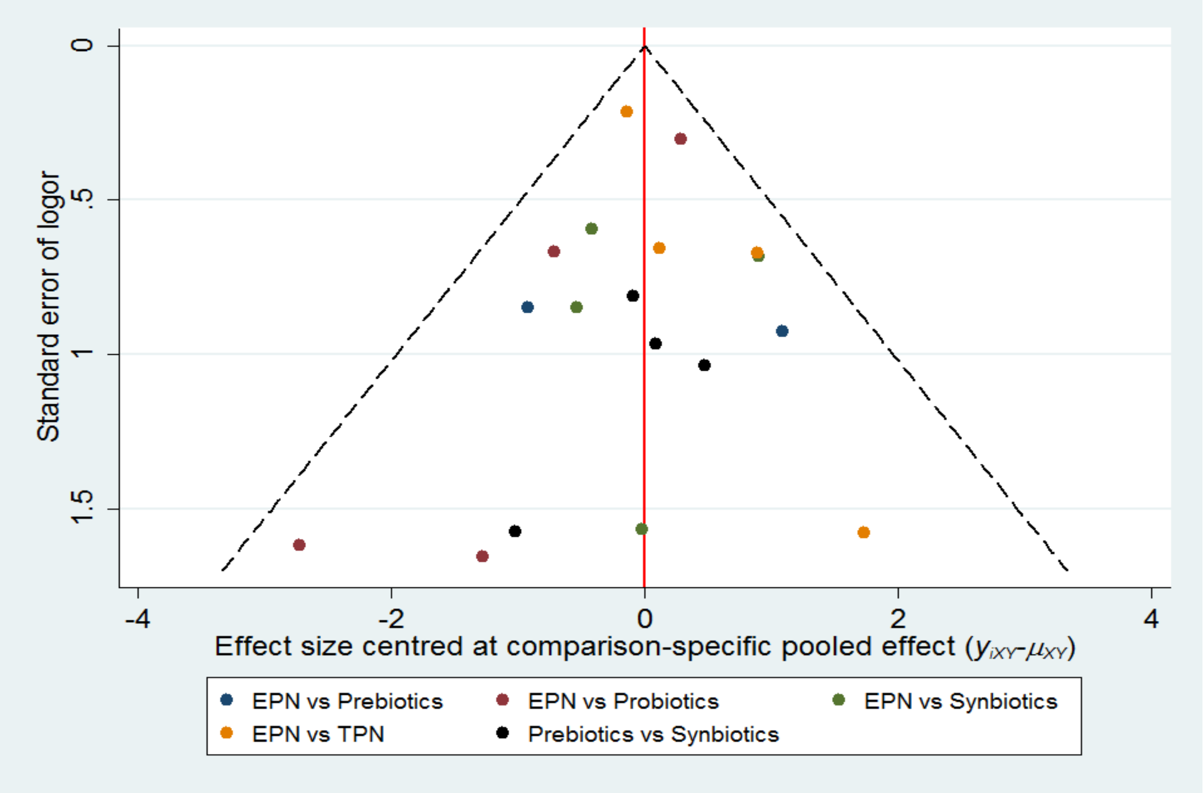

Figure S 10.5 Comparison-adjusted funnel plot for the network of catheter-related bloodstream infection in all comparisons

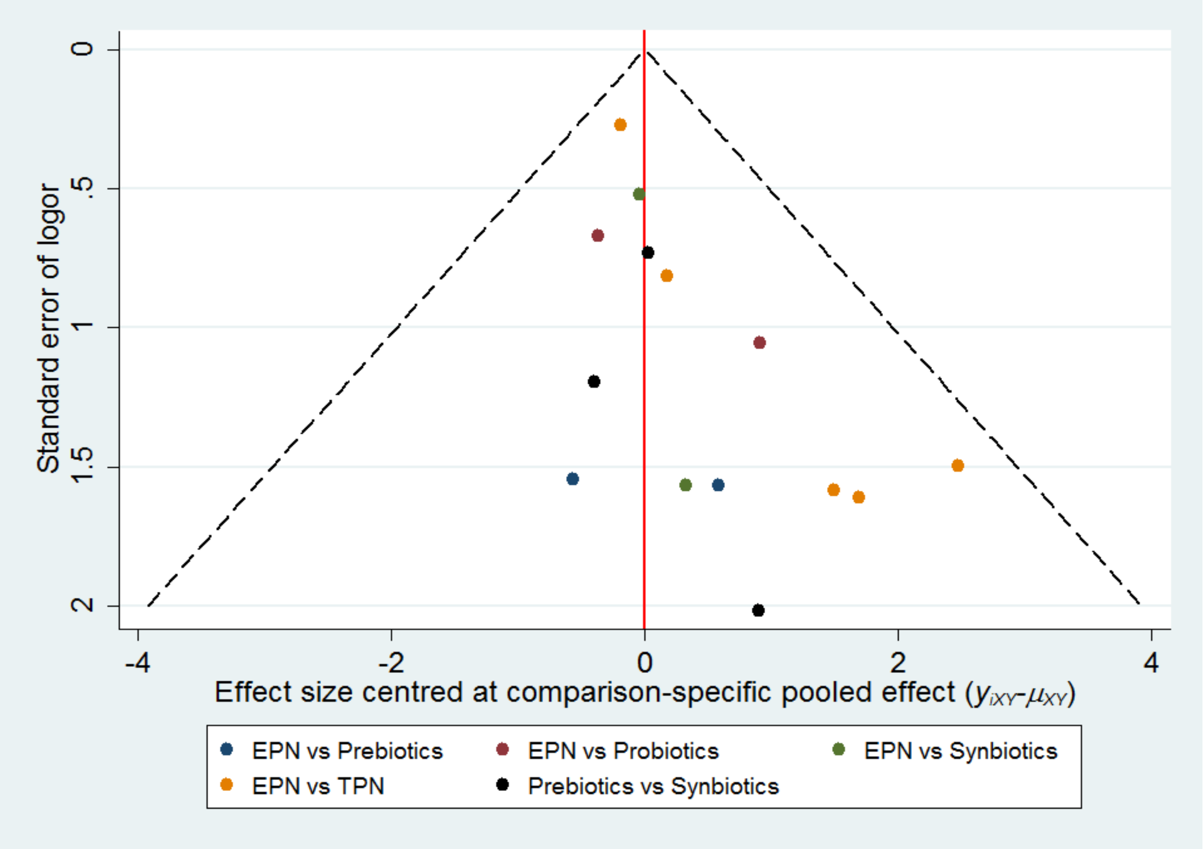

Figure S 10.6 Comparison-adjusted funnel plot for the network of urinary tract infections in all comparisons

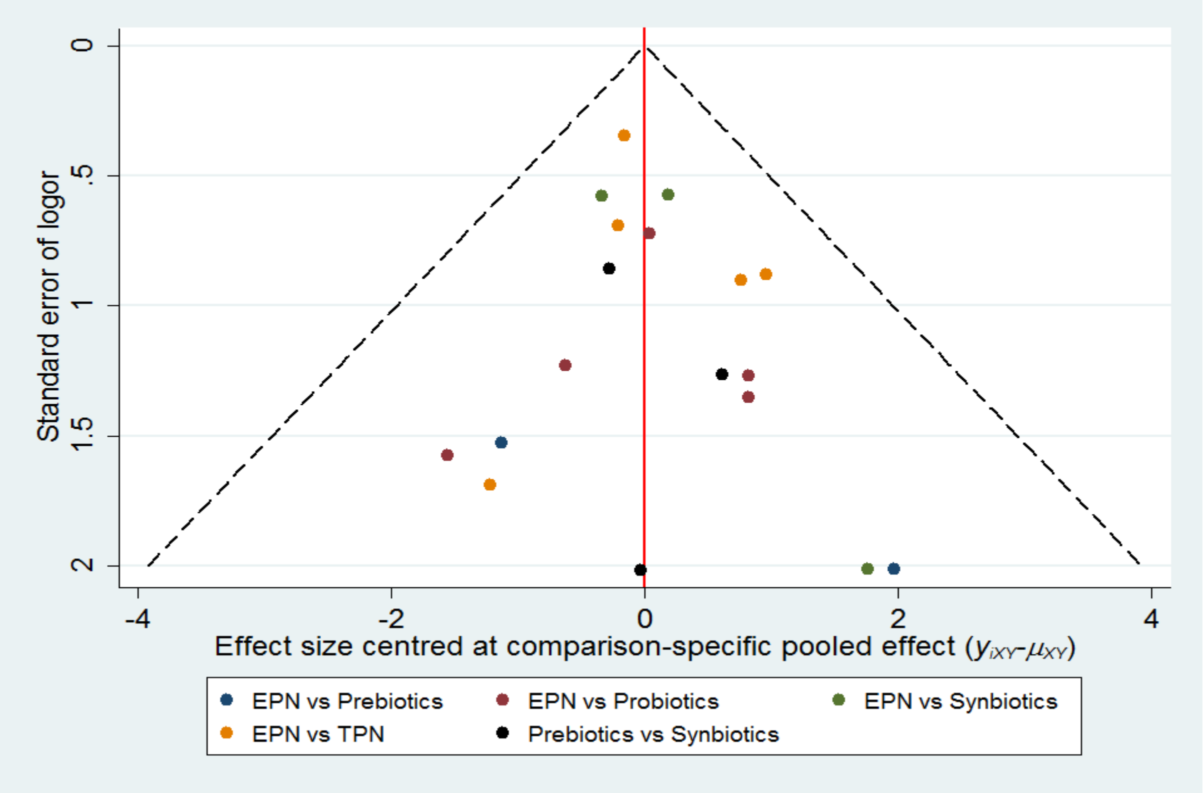

Figure S 10.7 Comparison-adjusted funnel plot for the network of sepsis comparisons

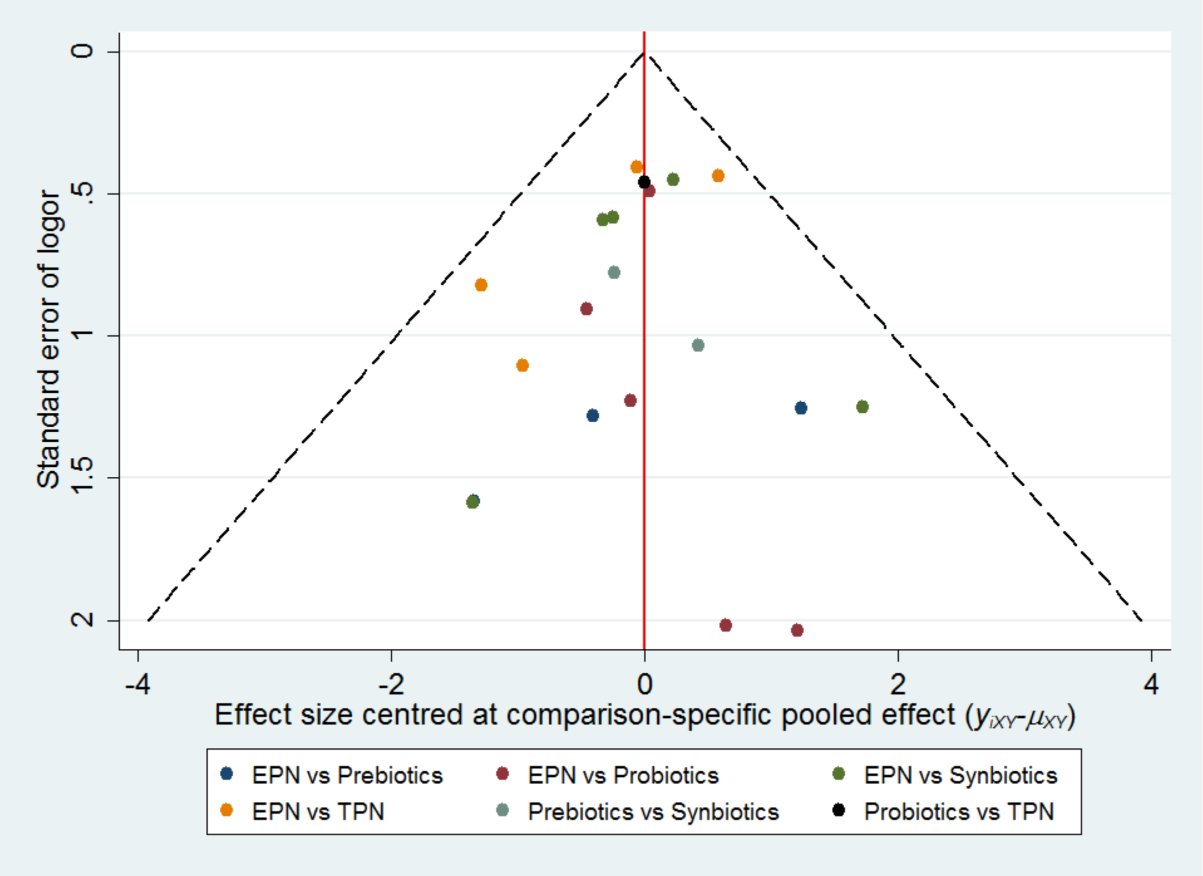

Figure S 10.8 Comparison-adjusted funnel plot for the network of diarrhea comparisons

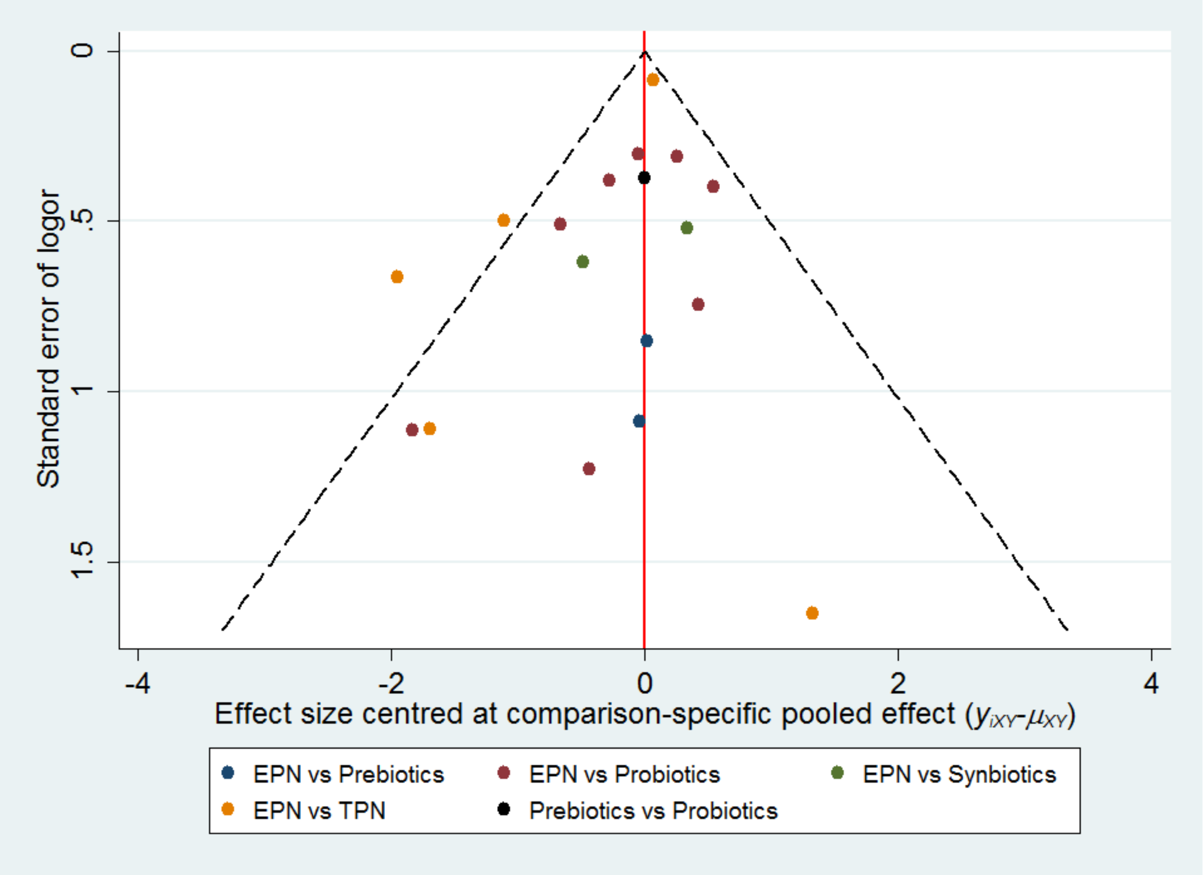

Figure S 10.9 Comparison-adjusted funnel plot for the network of hospital mortality in all comparisons

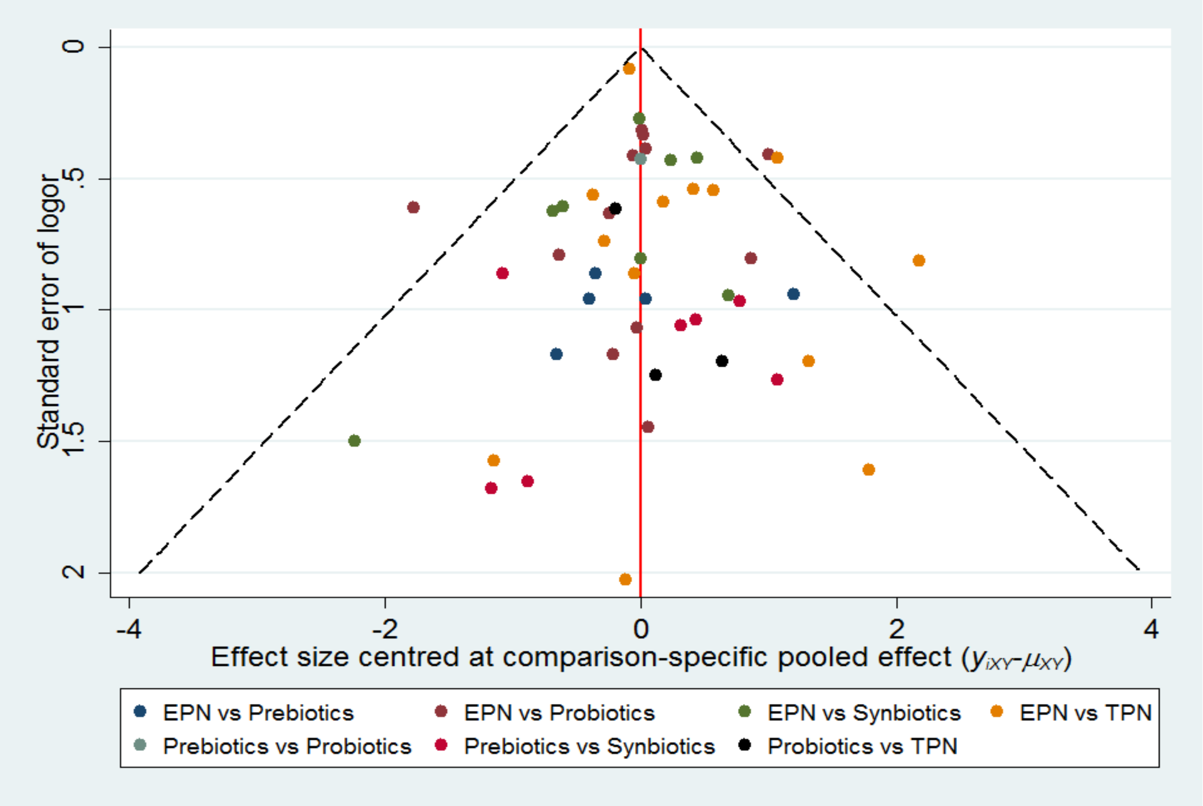

Figure S 10.10 Comparison-adjusted funnel plot for the network of ICU mortality in all comparisons

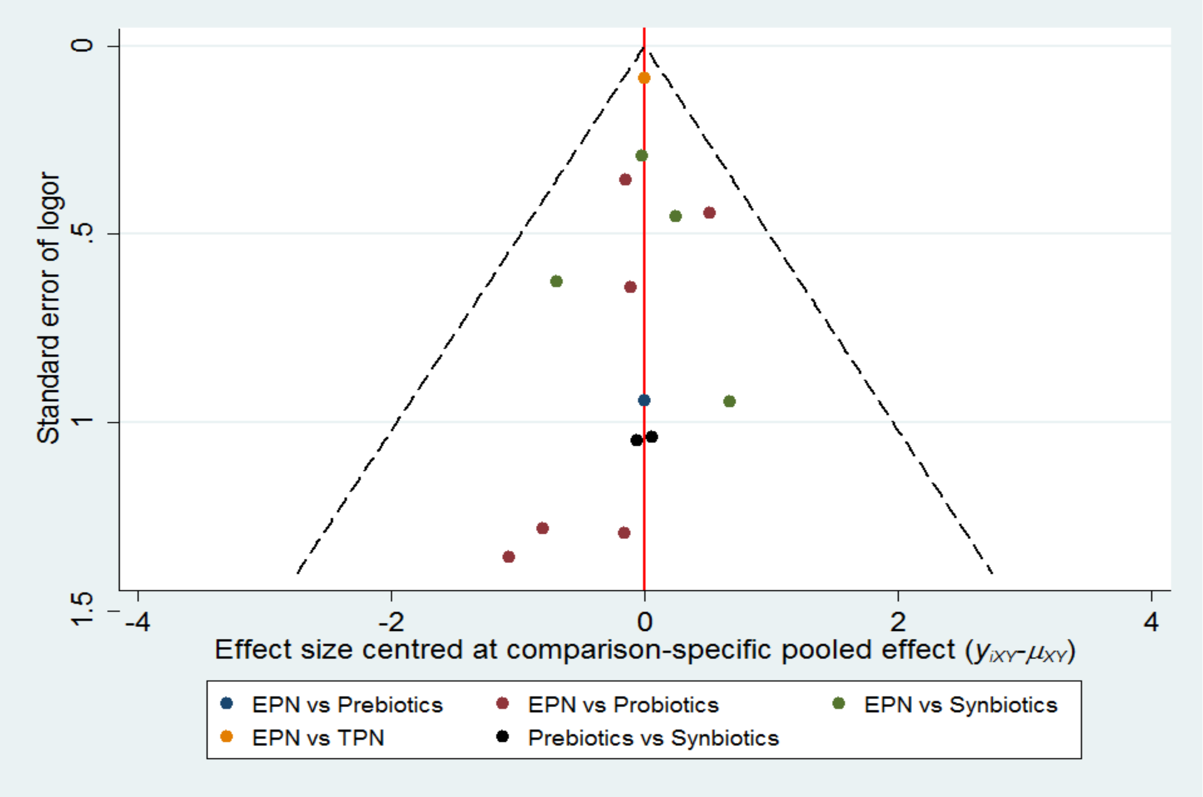

Figure S 10.11 Comparison-adjusted funnel plot for the network of hospital lengtn of stay in all comparisons

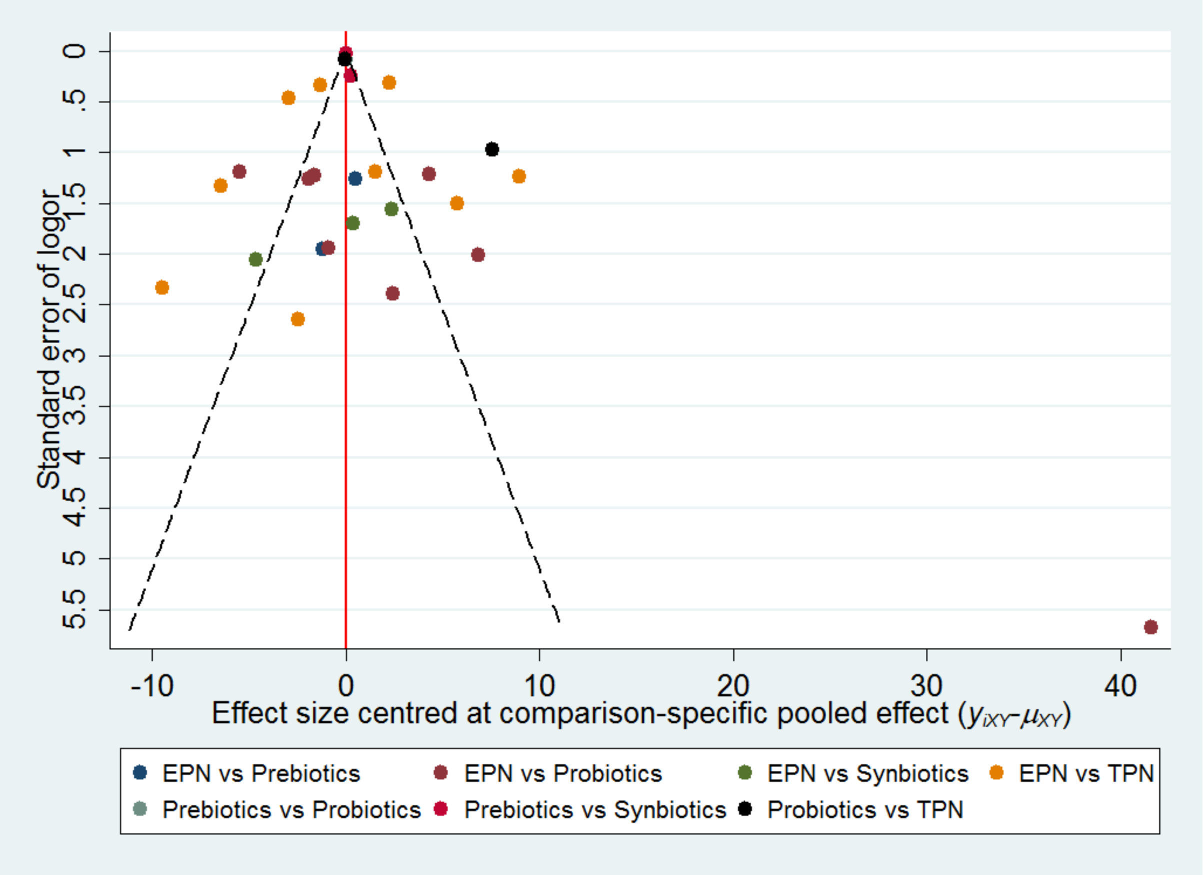

Figure S 10.12 Comparison-adjusted funnel plot for the network of ICU length of stay in all comparisons

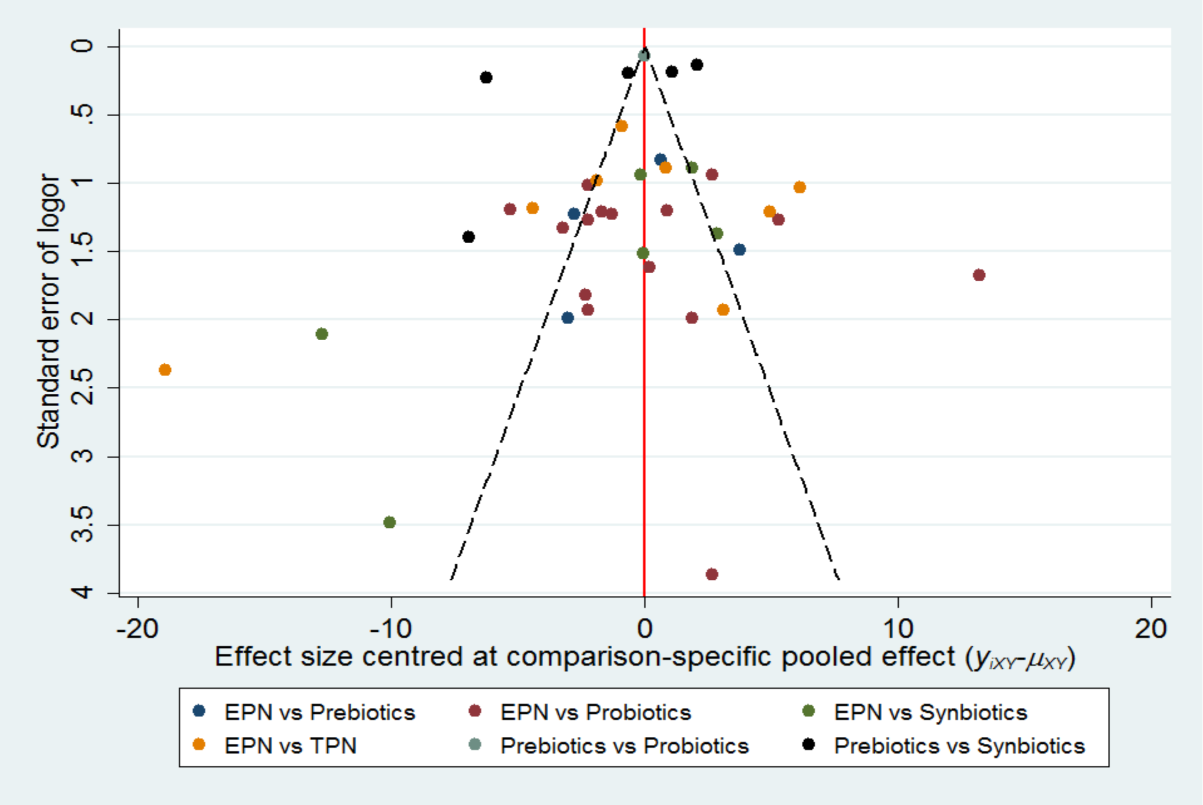

Figure S 10.13 Comparison-adjusted funnel plot for the network of the duration of mechanical ventilation in all comparisons

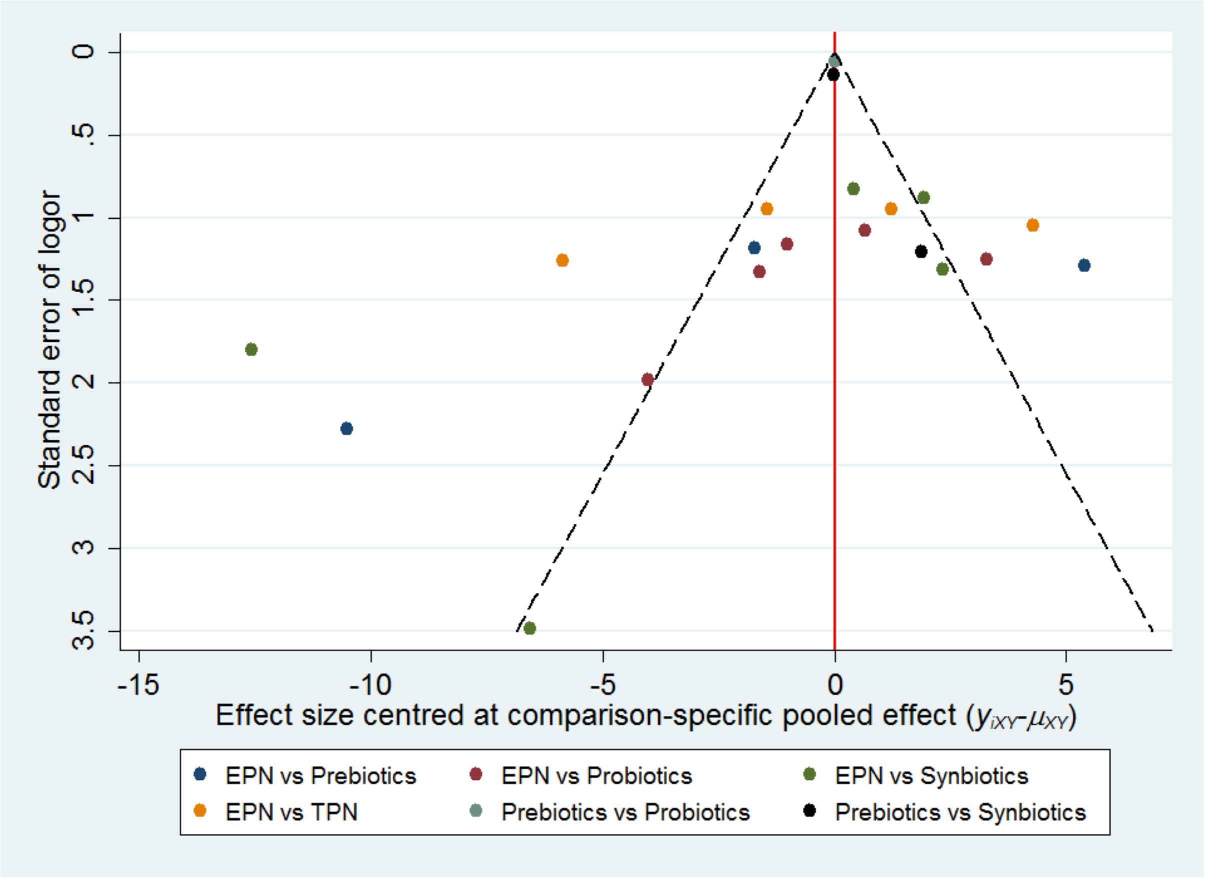

Appendix 11
GRADE for the primary and second outcomes

Based on the above assessment of RoB for each comparison and the contribution matrix detailing contribution of each direct comparison to all network estimates, the following bar graphs show the percentage of low or moderate or high RoB contributions for each network estimate.
The judgements about study limitations in each direct comparison is shown at the beginning of the graph. Each bar corresponds to a NMA relative treatment effect and shows how much information comes from comparisons at low risk of bias [green] or moderate risk of bias [yellow].

Based on all the above information, we GRADEd each network estimate according to the following criteria.
(1)**Study limitations**: We downgraded by one level when the contributions from low RoB comparisons were less than 30% and contributions from moderate RoB comparisons were 70% or greater.
(2)**Imprecision**: For dichotomous variables, we considered a clinically meaningful threshold for OR to be 0.80 or 1.25 and downgraded the estimate if the OR point estimate is 1 or more and the lower limit of its confidence interval (CI) is below 0.80; or if the OR point estimate is less than 1 and the upper limit of its CI is above 1.25. For continuous variables, we downgrade when CI crosses null value or includes values favoring either treatment.
(3)**Inconsistency**: We rated two concepts, heterogeneity and incoherence (inconsistency), in this domain. For heterogeneity, we looked at the I^2 and found weather it is high compared to the expected value (50%). For inconsistency, we looked at the results of side splitting and 'design-by-treatment' interaction model. We did downgraded the comparisons with important inconsistency ($p<0.05$) (we could downgrade the same network estimate for both heterogeneity and inconsistency).
(4)**Indirectness**: We have assured transitivity in our network by limiting the included studies to critically ill adult patients. We assured that they did not violate transitivity of the network. Hence, we did not downgraded singly-connected nodes for indirectness because evaluation of transitivity for such nodes is clear.
(5)**Publication bias**: We managed to retrieve Appendix and unpublished information included in the available systematic reviews and network meta-analyses, and we assessed publication bias using the comparison-adjusted funnel plots

Figure S 11.1 Contribution plot for NI and contribution of low or moderate RoB comparisons to each network estimate of NI

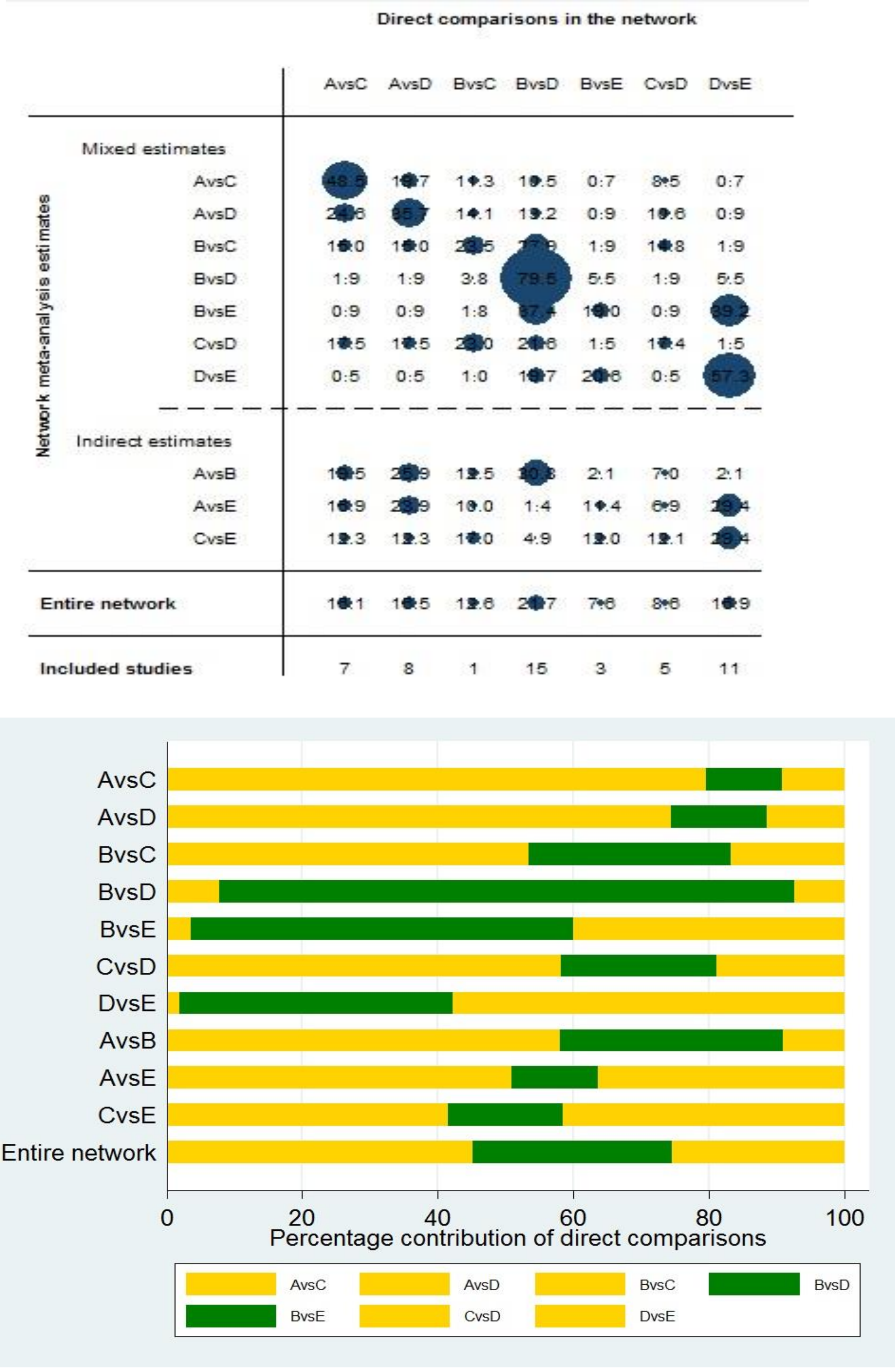

A: Synbiotics; B: Probiotics; C: Probiotics; D: EPN; E: TPN

Table S 11.1 Result of GRADE for NI

	Nature of the evidence	Study limitations	Imprecision	Inconsistency	Indirectness	Publication bias	Confidence	Downgrading due to
A vs B	Indirect estimated	No downgrade	No downgrade	No downgrade	No downgrade	No downgrade	HIGH	-
A vs C	Mixed estimated	Downgrade because >70% contribution from moderate Rob comparisons	No downgrade	No downgrade	No downgrade	No downgrade	MODERATE	Study limitations
A vs D	Mixed estimated	Downgrade because >70% contribution from moderate Rob comparisons	No downgrade	Downgrade because pair heterogeneity I^2=68.7%	No downgrade	No downgrade	LOW	Study limitations Inconsistency
A vs E	Indirect estimated	Downgrade because >70% contribution from moderate Rob comparisons	Downgrade because point estimate >1.0 but lower limit<0.80	No downgrade	No downgrade	No downgrade	LOW	Study limitations Imprecision
B vs C	Mixed estimated	No downgrade	No downgrade	No downgrade	No downgrade	No downgrade	HIGH	Inconsistency
B vs D	Mixed estimated	No downgrade	No downgrade	No downgrade Downgrade because sidesplitting p=0.04143	No downgrade	No downgrade	MODERATE	Inconsistency
B vs E	Mixed estimated	No downgrade	No downgrade	No downgrade	No downgrade	No downgrade	HIGH	-
C vs D	Mixed estimated	Downgrade because >70% contribution from moderate Rob comparisons	Downgrade because point estimate >1.0 but lower limit<0.80	Downgrade because pair heterogeneity I^2=57.4%	No downgrade	No downgrade	VERY LOW	Study limitations Imprecision Inconsistency
C vs E	Indirect estimated	Downgrade because >70% contribution from moderate Rob comparisons	No downgrade	No downgrade	No downgrade	No downgrade	MODERATE	Study limitations
D vs E	Mixed estimated	No downgrade	No downgrade	Downgrade because pair heterogeneity I^2=76.4%	No downgrade	No downgrade	LOW	Imprecision Inconsistency
Ranking of treatments		Downgrade because >70% contribution from moderate Rob comparisons	No downgrade	Downgrade because global heterogeneity I^2=62.02%	No downgrade	No downgrade	LOW	Study limitations Inconsistency

A: Synbiotics; B: Probiotics; C: Probiotics; D: EPN; E: TPN

Figure S 11.2 Contribution plot for pneumonia and contribution of low or moderate RoB comparisons to each network estimate of hospital acquired pneumonia

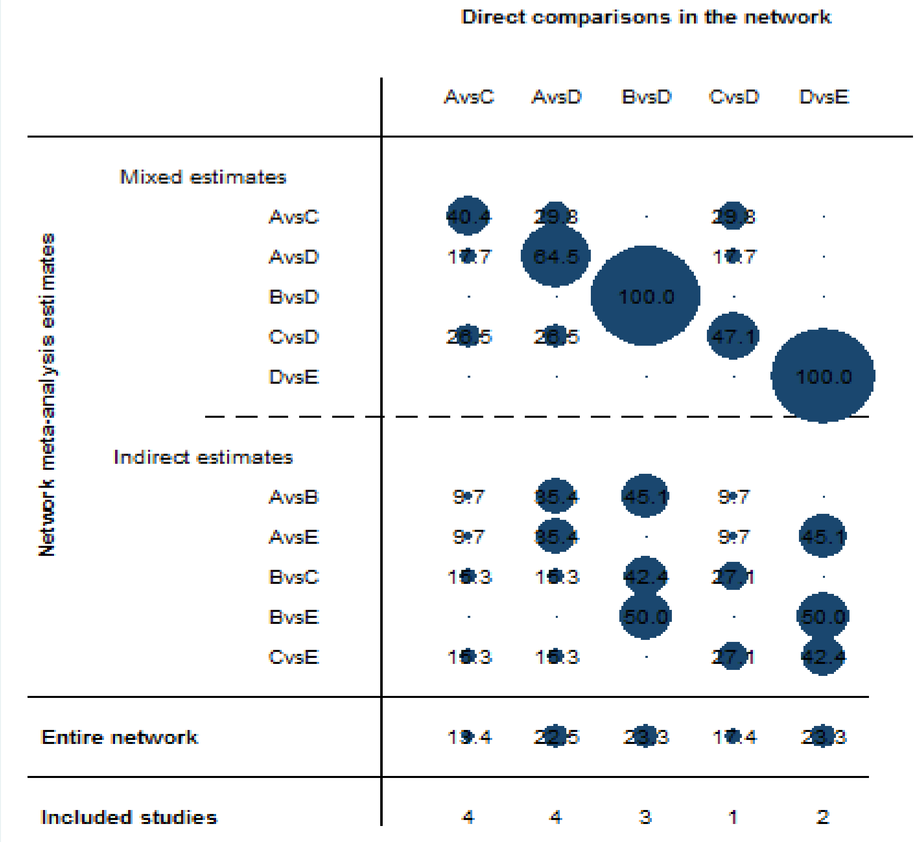

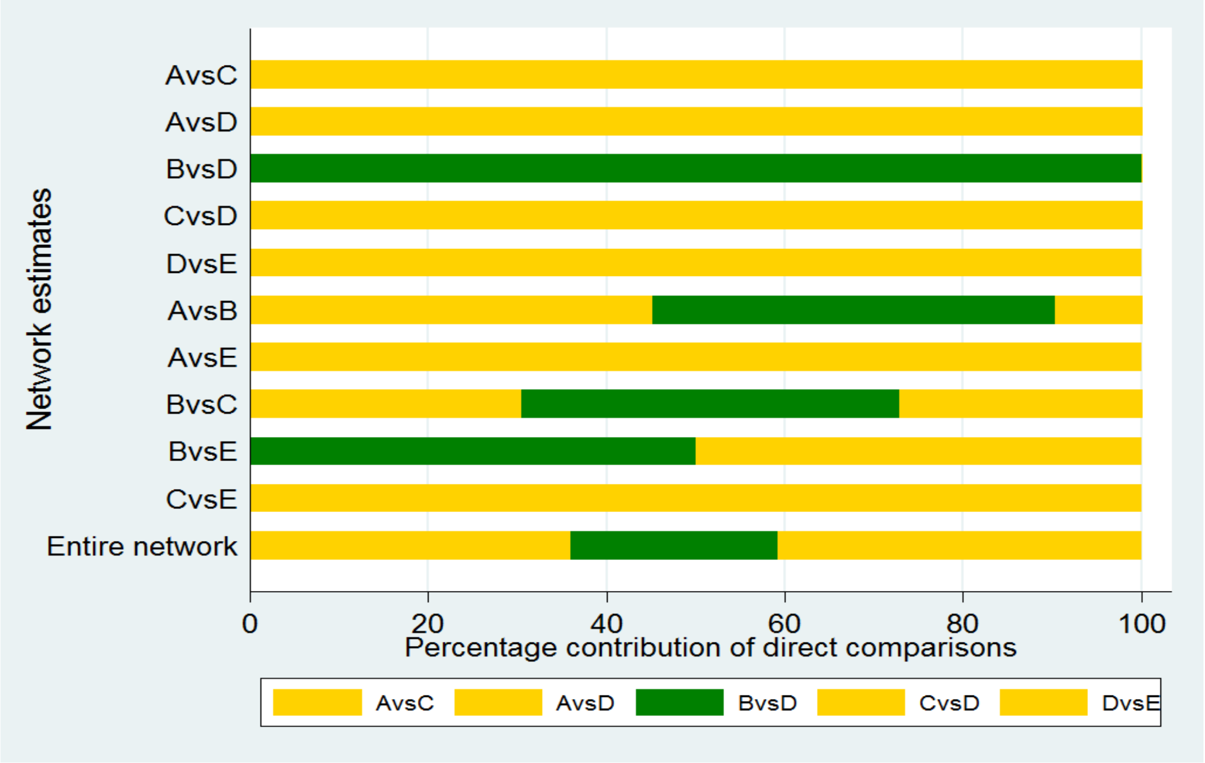

A: Synbiotics; B: Probiotics; C: Probiotics; D: EPN; E: TPN

Table S 11.2 Result of GRADE for hospital acquired pneumonia

	Nature of the evidence	Study limitations	Imprecision	Inconsistency	Indirectness	Publication bias	Confidence	Downgrading due to
A vs B	Indirect estimated	No downgrade	No downgrade	No downgrade	No downgrade	No downgrade	HIGH	-
A vs C	Mixed estimated	Downgrade because >70% contribution from moderate Rob comparisons	Downgrade because point estimate >1.0 but lower limit<0.80	No downgrade	No downgrade	No downgrade	LOW	Study limitations Imprecision
A vs D	Mixed estimated	Downgrade because >70% contribution from moderate Rob comparisons	Downgrade because point estimate < 1.0 but upper limit >1.25	No downgrade	No downgrade	No downgrade	LOW	Study limitations Imprecision
A vs E	Indirect estimated	Downgrade because >70% contribution from moderate Rob comparisons	Downgrade because point estimate >1.0 but lower limit<0.80	No downgrade	No downgrade	No downgrade	LOW	Study limitations Imprecision
B vs C	Indirect estimated	No downgrade	No downgrade	No downgrade	No downgrade	No downgrade	HIGH	-
B vs D	Mixed estimated	No downgrade	No downgrade	No downgrade	No downgrade	No downgrade	HIGH	-
B vs E	Indirect estimated	No downgrade	No downgrade	No downgrade	No downgrade	No downgrade	HIGH	-
C vs D	Mixed estimated	Downgrade because >70% contribution from moderate Rob comparisons	Downgrade because point estimate < 1.0 but upper limit >1.25	No downgrade	No downgrade	No downgrade	LOW	Study limitations Imprecision
C vs E	Indirect estimated	Downgrade because >70% contribution from moderate Rob comparisons	Downgrade because point estimate >1.0 but lower limit<0.80	No downgrade	No downgrade	No downgrade	LOW	Study limitations Imprecision
D vs E	Mixed estimated	Downgrade because >70% contribution from moderate Rob comparisons	Downgrade because point estimate >1.0 but lower limit<0.80	No downgrade	No downgrade	No downgrade	LOW	Study limitations Imprecision
Ranking of treatments		Downgrade because >70% contribution from moderate Rob comparisons	No downgrade	No downgrade	No downgrade	No downgrade	MODERATE	Study limitations

A: Synbiotics; B: Probiotics; C: Probiotics; D: EPN; E: TPN

Figure S 11.3 Contribution plot for VAP and contribution of low or moderate RoB comparisons to each network estimate of VAP

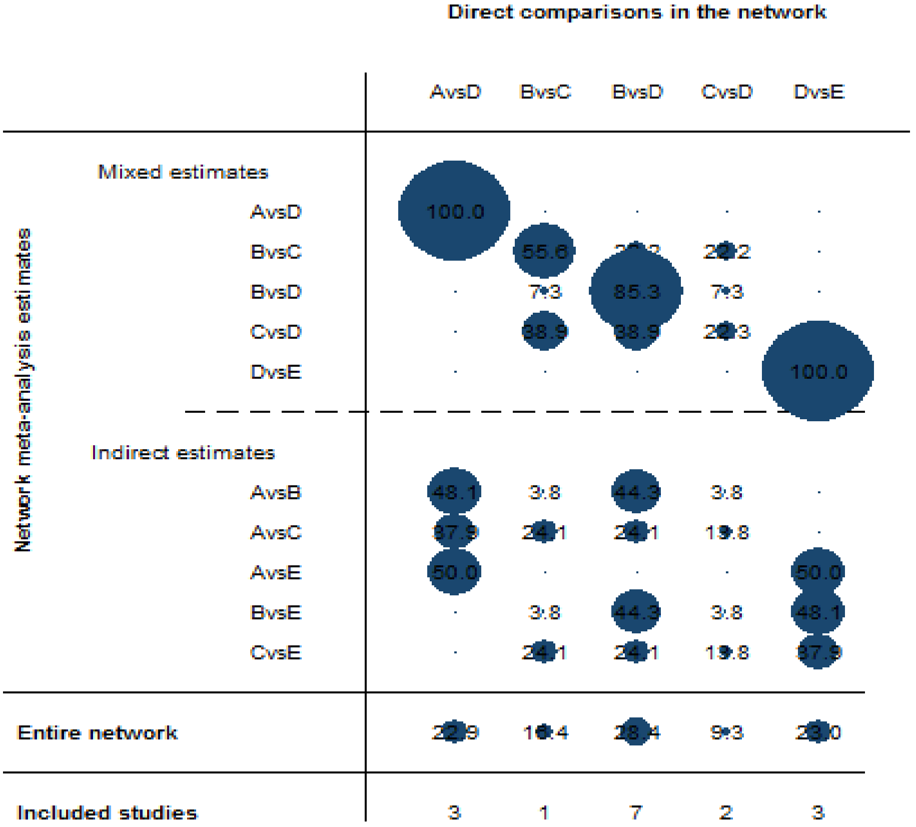

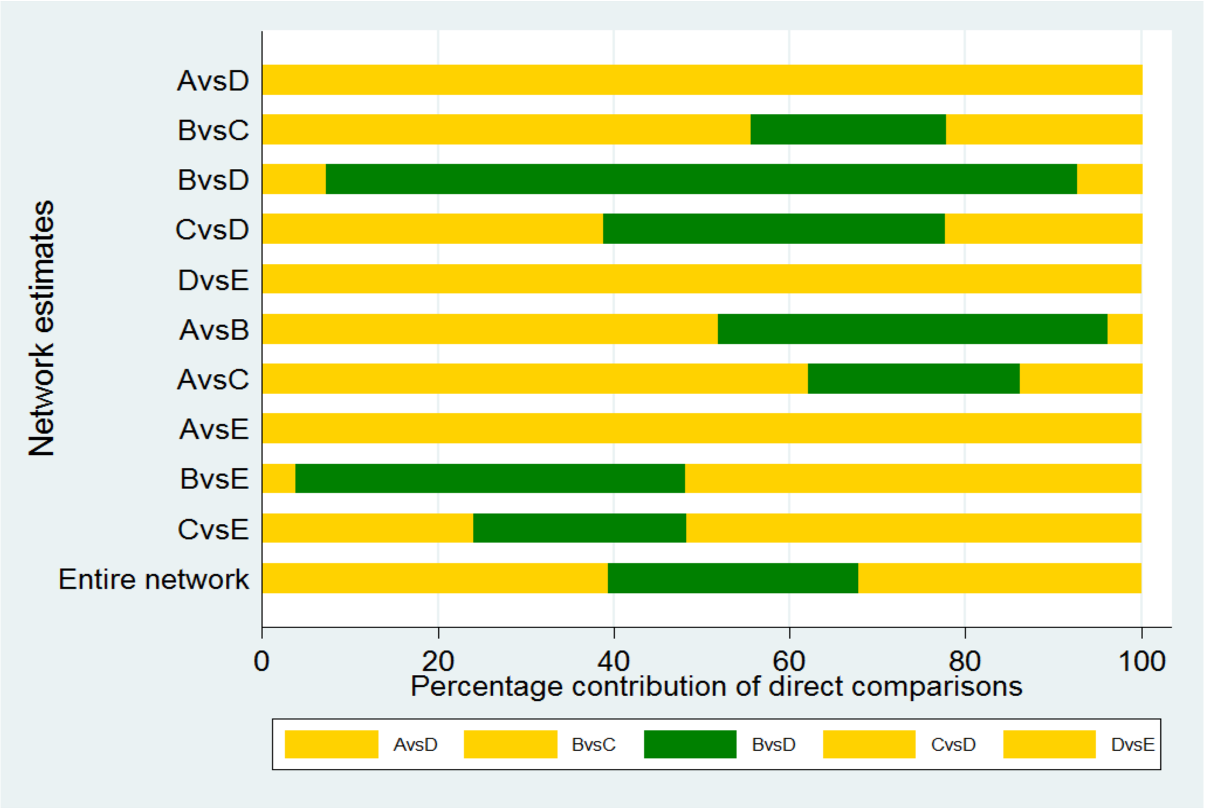

A: Synbiotics; B: Probiotics; C: Probiotics; D: EPN; E: TPN

Table S 11.3 Result of GRADE for VAP

	Nature of the evidence	Study limitations	Imprecision	Inconsistency	Indirectness	Publication bias	Confidence	Downgrading due to
A vs B	Indirect estimated	No downgrade	No downgrade	No downgrade	No downgrade	No downgrade	HIGH	-
A vs C	Indirect estimated	Downgrade because ＞70% contribution from moderate Rob comparisons	No downgrade	No downgrade	No downgrade	No downgrade	MODERATE	Study limitations
A vs D	Mixed estimated	Downgrade because ＞70% contribution from moderate Rob comparisons	Downgrade because point estimate < 1.0 but upper limit >1.25	Downgrade because pair heterogeneity I^2=62.6%	No downgrade	No downgrade	VERY LOW	Study limitations Imprecision Inconsistency
A vs E	Indirect estimated	Downgrade because ＞70% contribution from moderate Rob comparisons	Downgrade because point estimate >1.0 but lower limit<0.80	No downgrade	No downgrade	No downgrade	LOW	Study limitations Imprecision
B vs C	Mixed estimated	Downgrade because ＞70% contribution from moderate Rob comparisons	Downgrade because point estimate >1.0 but lower limit<0.80	No downgrade	No downgrade	No downgrade	LOW	Study limitations Imprecision
B vs D	Mixed estimated	No downgrade	Downgrade because point estimate >1.0 but lower limit<0.80	No downgrade	No downgrade	No downgrade	MODERATE	Imprecision
B vs E	Indirect estimated	No downgrade	Downgrade because point estimate >1.0 but lower limit<0.80	No downgrade	No downgrade	No downgrade	MODERATE	Imprecision
C vs D	Mixed estimated	No downgrade	Downgrade because point estimate >1.0 but lower limit<0.80	No downgrade	No downgrade	No downgrade	MODERATE	Imprecision
C vs E	Indirect estimated	Downgrade because ＞70% contribution from moderate Rob comparisons	Downgrade because point estimate >1.0 but lower limit<0.80	No downgrade	No downgrade	No downgrade	LOW	Study limitations Imprecision
D vs E	Mixed estimated	Downgrade because ＞70% contribution from moderate Rob comparisons	Downgrade because point estimate >1.0 but lower limit<0.80	No downgrade	No downgrade	No downgrade	LOW	Study limitations Imprecision
Ranking of treatments		Downgrade because ＞70% contribution from moderate Rob comparisons	No downgrade	Downgrade because global heterogeneity I^2=54.33%	No downgrade	No downgrade	LOW	Study limitations Inconsistency

A: Synbiotics; B: Probiotics; C: Probiotics; D: EPN; E: TPN

Figure S 11.4 Contribution plot for bloodstream infection and contribution of low or moderate RoB comparisons to each network estimate of bloodstream infection

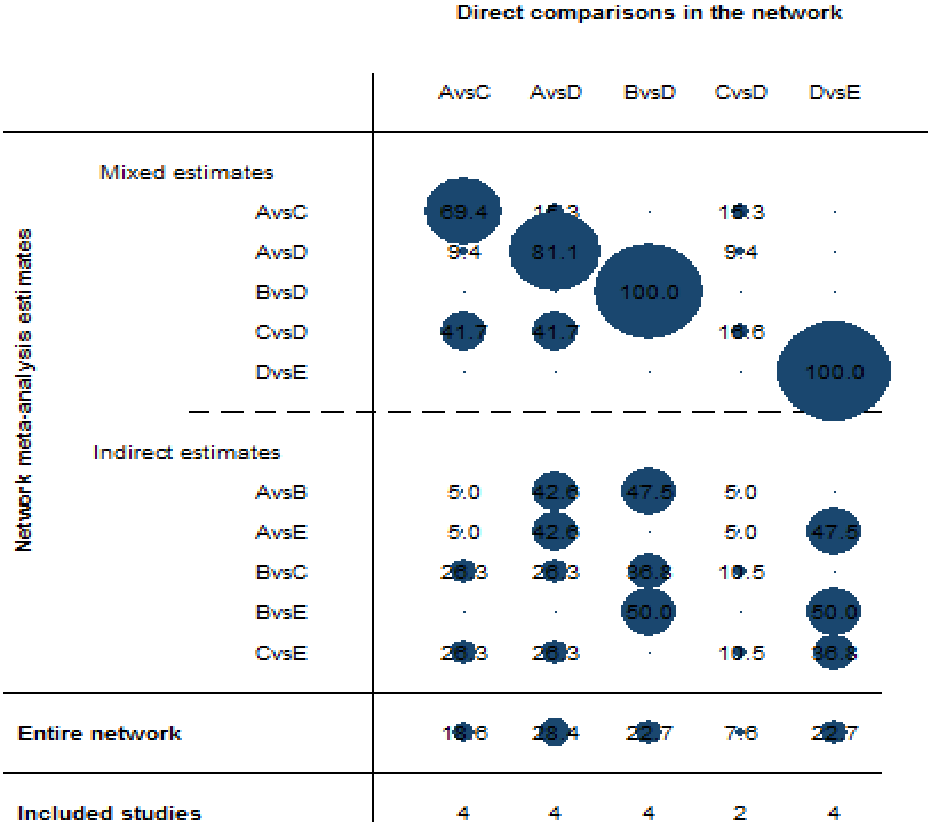

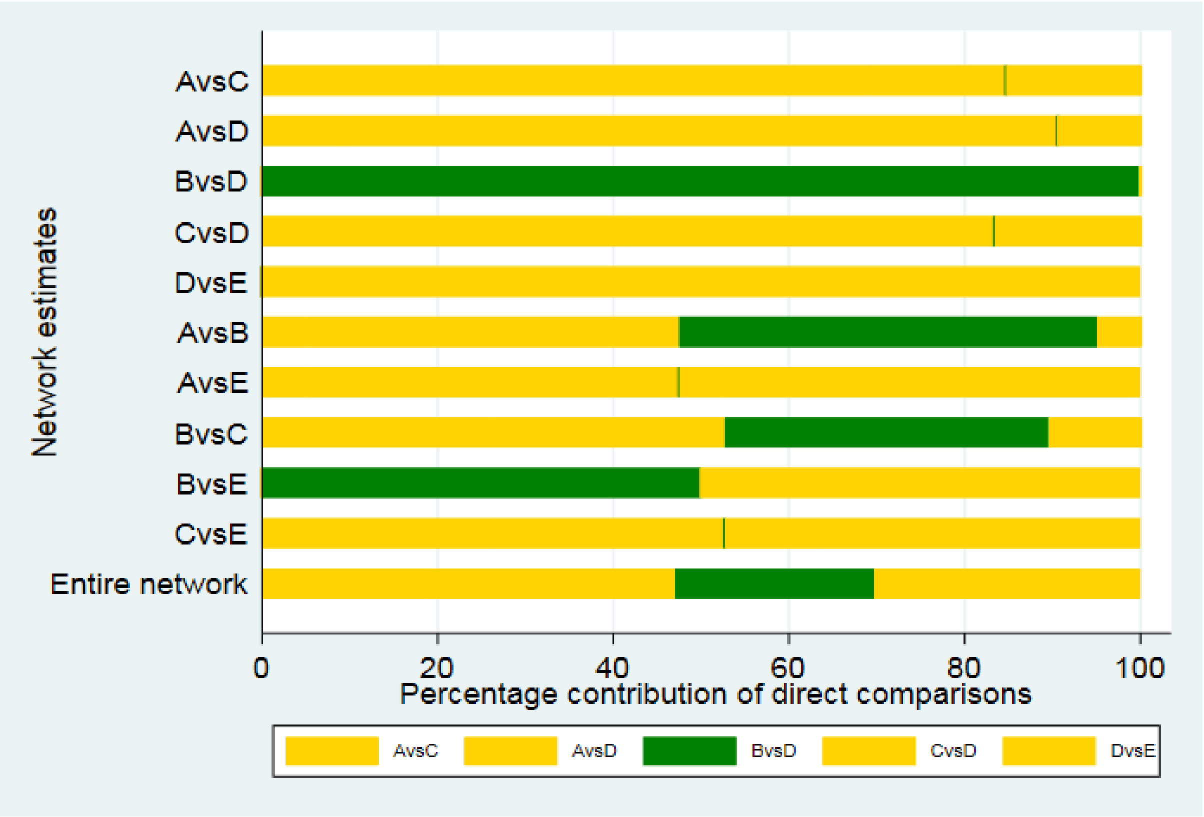

A: Synbiotics; B: Probiotics; C: Probiotics; D: EPN; E: TPN

Table S 11.4 Result of GRADE for bloodstream infection

	Nature of the evidence	Study limitations	Imprecision	Inconsistency	Indirectness	Publication bias	Confidence	Downgrading due to
A vs B	Indirect estimated	No downgrade	No downgrade	No downgrade	No downgrade	No downgrade	HIGH	-
A vs C	Mixed estimated	Downgrade because >70% contribution from moderate Rob comparisons	Downgrade because point estimate < 1.0 but upper limit >1.25	No downgrade	No downgrade	No downgrade	LOW	Study limitations Imprecision
A vs D	Mixed estimated	Downgrade because >70% contribution from moderate Rob comparisons	Downgrade because point estimate < 1.0 but upper limit >1.25	No downgrade	No downgrade	No downgrade	LOW	Study limitations Imprecision
A vs E	Indirect estimated	Downgrade because >70% contribution from moderate Rob comparisons	No downgrade	No downgrade	No downgrade	No downgrade	MODERATE	Study limitations Imprecision
B vs C	Indirect estimated	No downgrade	Downgrade because point estimate >1.0 but lower limit<0.80	No downgrade	No downgrade	No downgrade	MODERATE	Imprecision
B vs D	Mixed estimated	No downgrade	Downgrade because point estimate >1.0 but lower limit<0.80	Downgrade because pair heterogeneity I^2=75.1%	No downgrade	No downgrade	MODERATE	Imprecision Inconsistency
B vs E	Indirect estimated	No downgrade	No downgrade	No downgrade	No downgrade	No downgrade	HIGH	Imprecision
C vs D	Mixed estimated	Downgrade because >70% contribution from moderate Rob comparisons	Downgrade because point estimate < 1.0 but upper limit >1.25	Downgrade because pair heterogeneity I^2=51.1%	No downgrade	No downgrade	VERY LOW	Study limitations Imprecision Inconsistency
C vs E	Indirect estimated	Downgrade because >70% contribution from moderate Rob comparisons	Downgrade because point estimate >1.0 but lower limit<0.80	No downgrade	No downgrade	No downgrade	LOW	Study limitations Imprecision
D vs E	Mixed estimated	Downgrade because >70% contribution from moderate Rob comparisons	Downgrade because point estimate >1.0 but lower limit<0.80	Downgrade because pair heterogeneity I^2=63.5%	No downgrade	No downgrade	VERY LOW	Study limitations Imprecision Inconsistency
Ranking of treatments		Downgrade because >70% contribution from moderate Rob comparisons	No downgrade	No downgrade	No downgrade	No downgrade	MODERATE	Study limitations

A: Synbiotics; B: Probiotics; C: Probiotics; D: EPN; E: TPN

Figure S 11.5 Contribution plot for CRB and contribution of low or moderate RoB comparisons to each network estimate of CRB

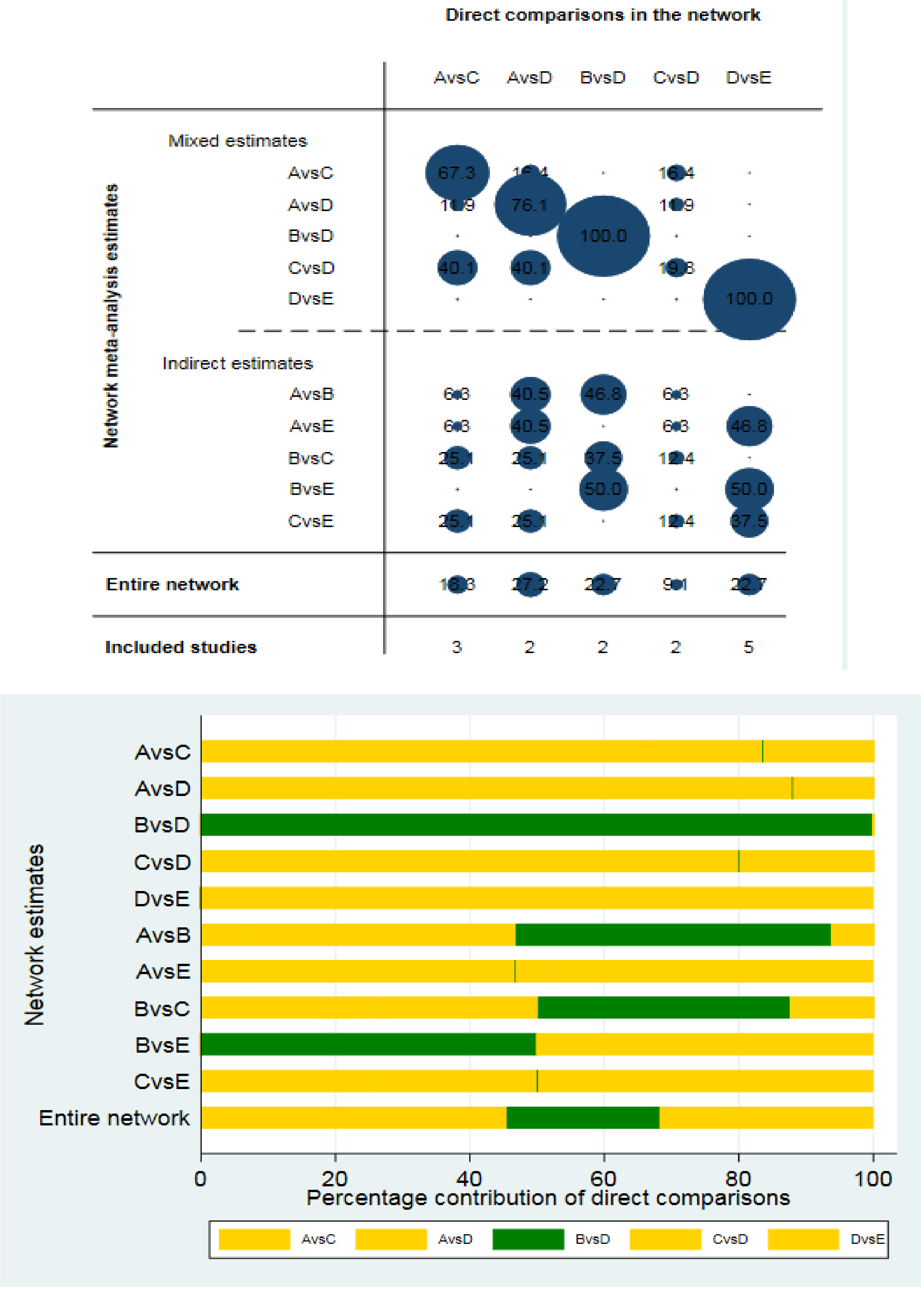

A: Synbiotics; B: Probiotics; C: Probiotics; D: EPN; E: TPN

Table S 11.5 Result of GRADE for CRBIS

	Nature of the evidence	Study limitations	Imprecision	Inconsistency	Indirectness	Publication bias	Confidence	Downgrading due to
A vs B	Indirect estimated	No downgrade	No downgrade	No downgrade	No downgrade	No downgrade	HIGH	-
A vs C	Mixed estimated	Downgrade because >70% contribution from moderate Rob comparisons	No downgrade	Downgrade because side splitting p=0.03569	No downgrade	No downgrade	LOW	Study limitations Inconsistency
A vs D	Mixed estimated	Downgrade because >70% contribution from moderate Rob comparisons	Downgrade because point estimate < 1.0 but upper limit >1.25	Downgrade because side splitting p=0.04404	No downgrade	No downgrade	VERY LOW	Study limitations Imprecision Inconsistency
A vs E	Indirect estimated	Downgrade because >70% contribution from moderate Rob comparisons	Downgrade because point estimate >1.0 but lower limit<0.80	No downgrade	No downgrade	No downgrade	LOW	Study limitations Imprecision
B vs C	Indirect estimated	No downgrade	Downgrade because point estimate >1.0 but lower limit<0.80	No downgrade	No downgrade	No downgrade	MODERATE	Imprecision
B vs D	Mixed estimated	No downgrade	Downgrade because point estimate >1.0 but lower limit<0.80	No downgrade	No downgrade	No downgrade	MODERATE	Imprecision
B vs E	Indirect estimated	No downgrade	No downgrade	No downgrade	No downgrade	No downgrade	MODERATE	Imprecision
C vs D	Mixed estimated	Downgrade because >70% contribution from moderate Rob comparisons	Downgrade because point estimate >1.0 but lower limit<0.80	Downgrade because pair heterogeneity I^2=71.0% Downgrade because side splitting p=0.02783	No downgrade	No downgrade	VERY LOW	Study limitations Imprecision Inconsistency
C vs E	Indirect estimated	Downgrade because >70% contribution from moderate Rob comparisons	No downgrade	No downgrade	No downgrade	No downgrade	MODERATE	Study limitations
D vs E	Mixed estimated	Downgrade because >70% contribution from moderate Rob comparisons	Downgrade because point estimate >1.0 but lower limit<0.80	Downgrade because pair heterogeneity I^2=92.4%	No downgrade	No downgrade	VERY LOW	Study limitations Imprecision Inconsistency
Ranking of treatments		Downgrade because >70% contribution from moderate Rob comparisons	No downgrade	Downgrade because global heterogeneity I^2=79.14%	No downgrade	No downgrade	LOW	Study limitations Inconsistency

A: Synbiotics; B: Probiotics; C: Probiotics; D: EPN; E: TPN

Figure S 11.6 Contribution plot for UTI and contribution of low or moderate RoB comparisons to each network estimate of UTI

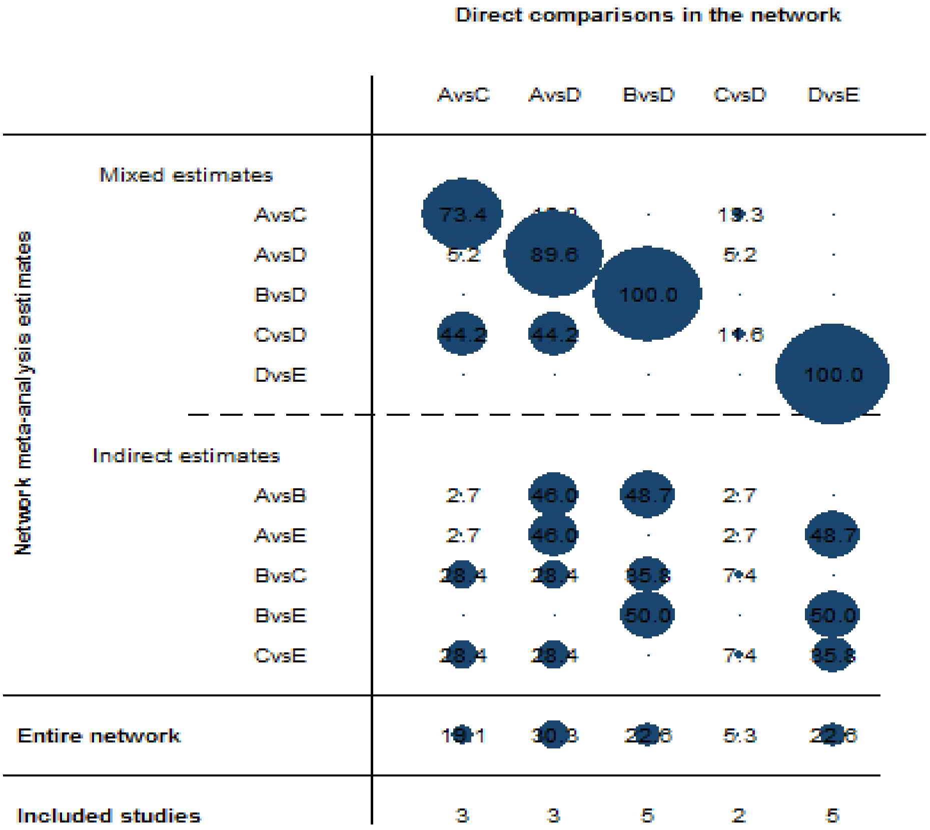

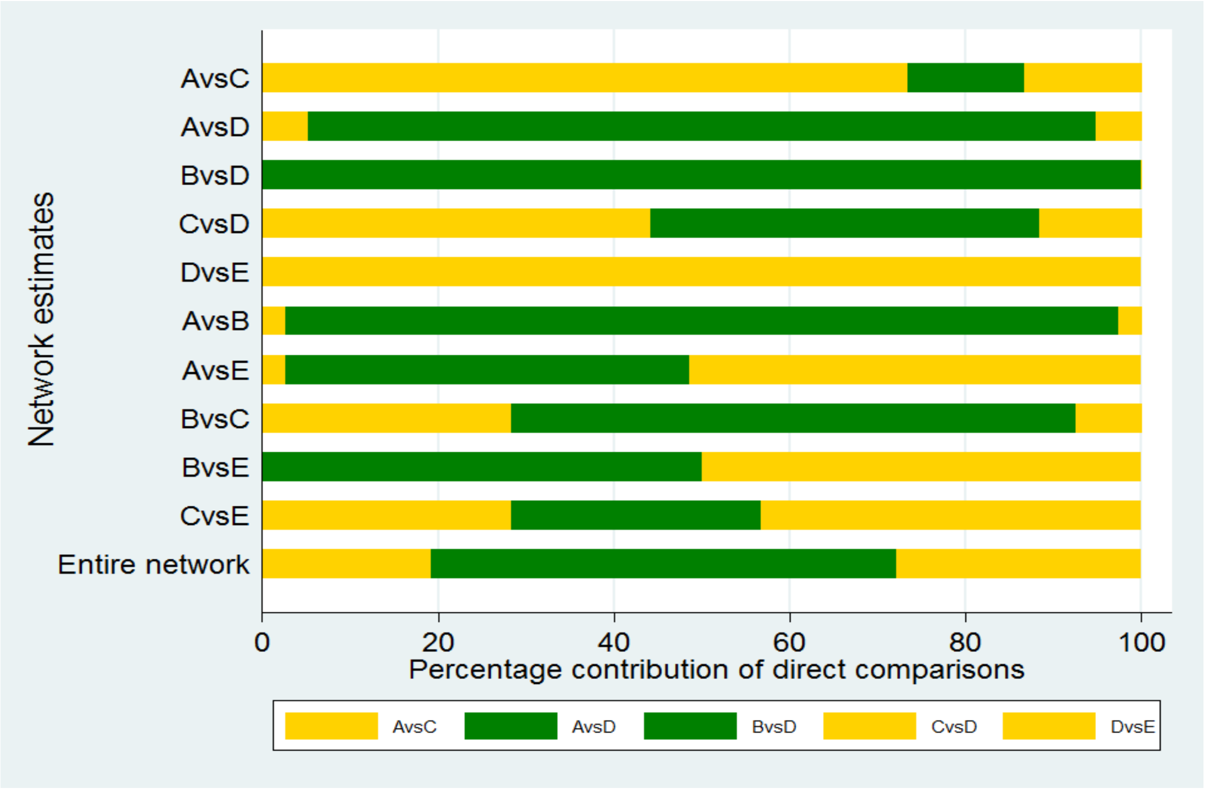

A: Synbiotics; B: Probiotics; C: Probiotics; D: EPN; E: TPN

Table S 11.6 Result of GRADE for UTI

	Nature of the evidence	Study limitations	Imprecision	Inconsistency	Indirectness	Publication bias	Confidence	Downgrading due to
A vs B	Indirect estimated	No downgrade	No downgrade	No downgrade	No downgrade	No downgrade	HIGH	-
A vs C	Mixed estimated	Downgrade because >70% contribution from moderate Rob comparisons	Downgrade because point estimate >1.0 but lower limit<0.80	Downgrade because side splitting p=0.04033	No downgrade	No downgrade	VERY LOW	Study limitations Imprecision Inconsistency
A vs D	Mixed estimated	No downgrade	Downgrade because point estimate >1.0 but lower limit<0.80	Downgrade because side splitting p=0.03591	No downgrade	No downgrade	LOW	Imprecision Inconsistency
A vs E	Indirect estimated	No downgrade	Downgrade because point estimate < 1.0 but upper limit >1.25	No downgrade	No downgrade	No downgrade	MODERATE	Imprecision
B vs C	Indirect estimated	No downgrade	Downgrade because point estimate >1.0 but lower limit<0.80	No downgrade	No downgrade	No downgrade	MODERATE	Imprecision
B vs D	Mixed estimated	No downgrade	Downgrade because point estimate < 1.0 but upper limit >1.25	No downgrade	No downgrade	No downgrade	MODERATE	Imprecision
B vs E	Indirect estimated	No downgrade	No downgrade	No downgrade	No downgrade	No downgrade	HIGH	-
C vs D	Mixed estimated	No downgrade	Downgrade because point estimate < 1.0 but upper limit >1.25	Downgrade because side splitting p=0.04071	No downgrade	No downgrade	LOW	Imprecision Inconsistency
C vs E	Indirect estimated	Downgrade because >70% contribution from moderate Rob comparisons	Downgrade because point estimate >1.0 but lower limit<0.80	No downgrade	No downgrade	No downgrade	LOW	Study limitations Imprecision
D vs E	Mixed estimated	Downgrade because >70% contribution from moderate Rob comparisons	Downgrade because point estimate >1.0 but lower limit<0.80	No downgrade	No downgrade	No downgrade	LOW	Study limitations Imprecision
Ranking of treatments		No downgrade	No downgrade	No downgrade	No downgrade	No downgrade	HIGH	-

A: Synbiotics; B: Probiotics; C: Probiotics; D: EPN; E: TPN

Figure S 11.7 Contribution plot for sepsis and contribution of low or moderate RoB comparisons to each network estimate of sepsis

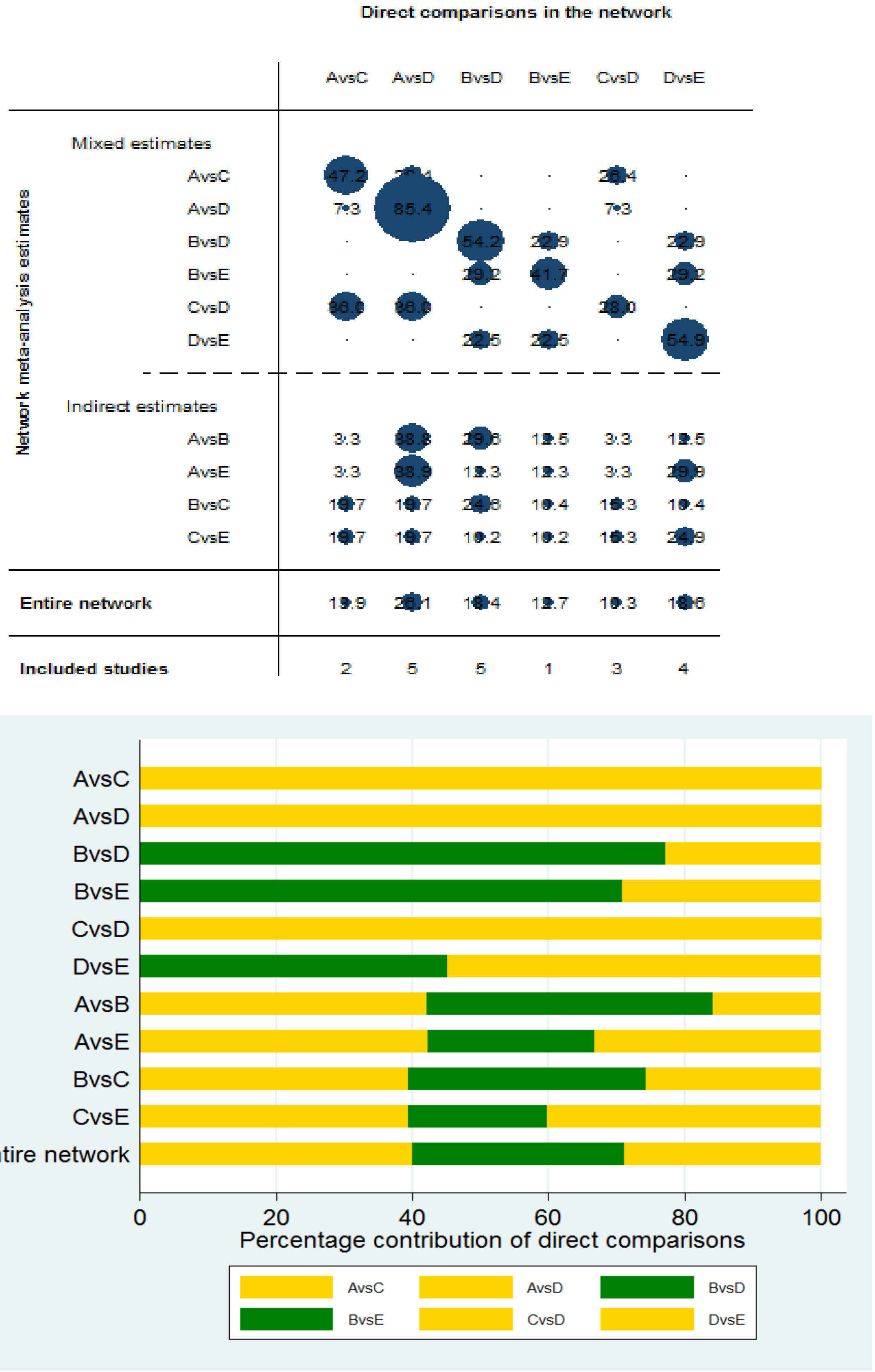

A: Synbiotics; B: Probiotics; C: Probiotics; D: EPN; E: TPN

Table S 11.7 Result of GRADE for sepsis

	Nature of the evidence	Study limitations	Imprecision	Inconsistency	Indirectness	Publication bias	Confidence	Downgrading due to
A vs B	Indirect estimated	No downgrade	No downgrade	No downgrade	No downgrade	No downgrade	HIGH	-
A vs C	Mixed estimated	Downgrade because >70% contribution from moderate Rob comparisons	No downgrade	No downgrade	No downgrade	No downgrade	MODERATE	Study limitations
A vs D	Mixed estimated	Downgrade because >70% contribution from moderate Rob comparisons	Downgrade because point estimate < 1.0 but upper limit >1.25	No downgrade	No downgrade	No downgrade	LOW	Study limitations Imprecision
A vs E	Indirect estimated	Downgrade because >70% contribution from moderate Rob comparisons	No downgrade	No downgrade	No downgrade	No downgrade	MODERATE	Study limitations
B vs C	Indirect estimated	No downgrade	Downgrade because point estimate >1.0 but lower limit<0.80	No downgrade	No downgrade	No downgrade	MODERATE	Imprecision
B vs D	Mixed estimated	No downgrade	Downgrade because point estimate >1.0 but lower limit<0.80	No downgrade	No downgrade	No downgrade	MODERATE	Imprecision
B vs E	Mixed estimated	No downgrade	No downgrade	No downgrade	No downgrade	No downgrade	HIGH	-
C vs D	Mixed estimated	Downgrade because >70% contribution from moderate Rob comparisons	Downgrade because point estimate >1.0 but lower limit<0.80	No downgrade	No downgrade	No downgrade	LOW	Study limitations Imprecision
C vs E	Indirect estimated	Downgrade because >70% contribution from moderate Rob comparisons	No downgrade	No downgrade	No downgrade	No downgrade	MODERATE	Study limitations
D vs E	Mixed estimated	No downgrade	No downgrade	No downgrade	No downgrade	No downgrade	HIGH	-
Ranking of treatments		No downgrade	No downgrade	No downgrade	No downgrade	No downgrade	HIGH	-

A: Synbiotics; B: Probiotics; C: Probiotics; D: EPN; E: TPN

Figure S 11.8 Contribution plot for diarrhea and contribution of low or moderate RoB comparisons to each network estimate of diarrhea

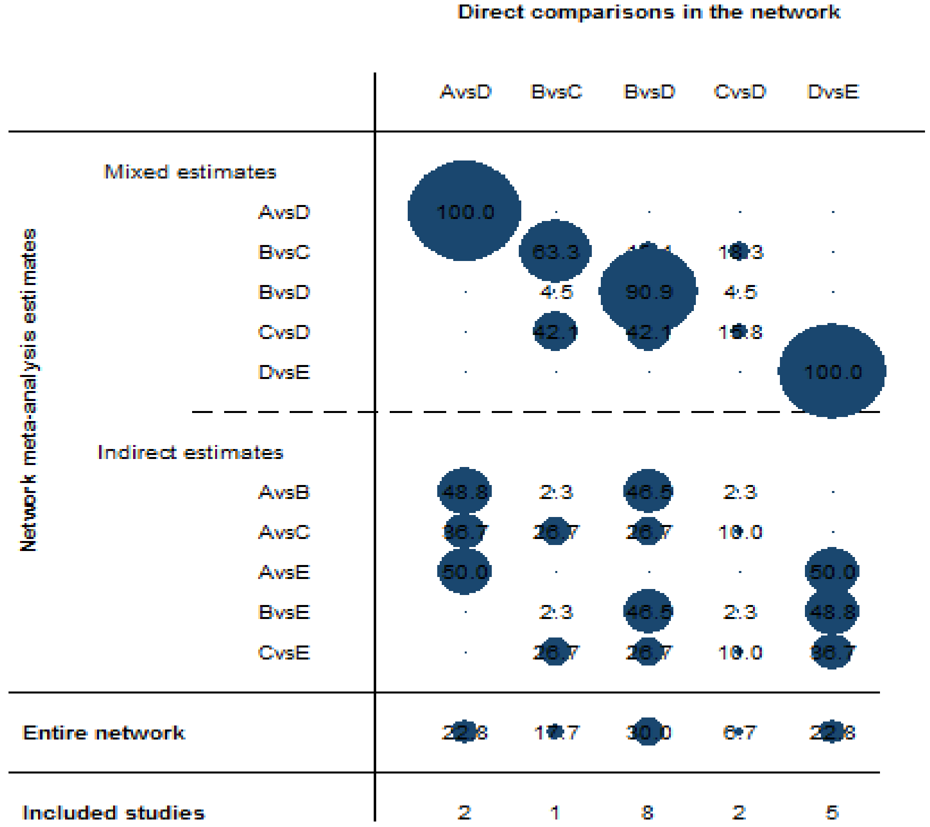

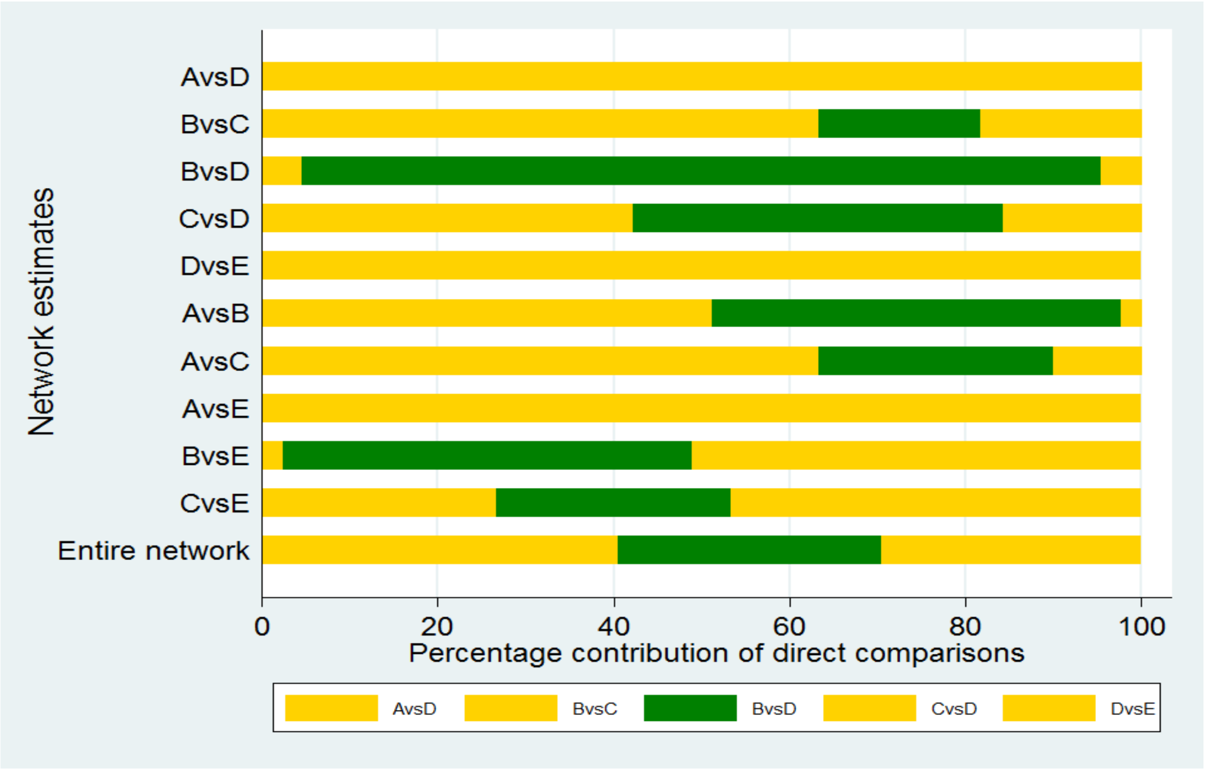

A: Synbiotics; B: Probiotics; C: Probiotics; D: EPN; E: TPN

Table S 11.8 Result of GRADE for diarrhea

	Nature of the evidence	Study limitations	Imprecision	Inconsistency	Indirectness	Publication bias	Confidence	Downgrading due to
A vs B	Indirect estimated	No downgrade	Downgrade because point estimate < 1.0 but upper limit >1.25	No downgrade	No downgrade	No downgrade	MODERATE	Imprecision
A vs C	Indirect estimated	Downgrade because ＞70% contribution from moderate Rob comparisons	No downgrade	No downgrade	No downgrade	No downgrade	MODERATE	Study limitations
A vs D	Mixed estimated	Downgrade because ＞70% contribution from moderate Rob comparisons	No downgrade	No downgrade	No downgrade	No downgrade	MODERATE	Study limitations
A vs E	Indirect estimated	Downgrade because ＞70% contribution from moderate Rob comparisons	No downgrade	No downgrade	No downgrade	No downgrade	LOW	Study limitations Imprecision
B vs C	Mixed estimated	Downgrade because ＞70% contribution from moderate Rob comparisons	Downgrade because point estimate >1.0 but lower limit<0.80	Downgrade because side splitting p=0.0103	No downgrade	No downgrade	LOW	Study limitations Imprecision Inconsistency
B vs D	Mixed estimated	No downgrade	Downgrade because point estimate < 1.0 but upper limit >1.25	Downgrade because side splitting p=0.01008	No downgrade	No downgrade	LOW	Imprecision Inconsistency
B vs E	Indirect estimated	No downgrade	Downgrade because point estimate < 1.0 but upper limit >1.25	No downgrade	No downgrade	No downgrade	MODERATE	Imprecision
C vs D	Mixed estimated	No downgrade	Downgrade because point estimate < 1.0 but upper limit >1.25	Downgrade because side splitting p=0.0106	No downgrade	No downgrade	LOW	Imprecision Inconsistency
C vs E	Indirect estimated	Downgrade because ＞70% contribution from moderate Rob comparisons	Downgrade because point estimate < 1.0 but upper limit >1.25	No downgrade	No downgrade	No downgrade	LOW	Study limitations Imprecision
D vs E	Mixed estimated	Downgrade because ＞70% contribution from moderate Rob comparisons	Downgrade because point estimate >1.0 but lower limit<0.80	Downgrade because pair heterogeneity I^2=95.9%	No downgrade	No downgrade	VERY LOW	Study limitations Imprecision Inconsistency
Ranking of treatments		No downgrade	No downgrade	Downgrade because global heterogeneity I^2=91.11% Downgrade because total inconsistency p=0.0018	No downgrade	No downgrade	MODERATE	Inconsistency

A: Synbiotics; B: Probiotics; C: Probiotics; D: EPN; E: TPN

Figure S 11.9 Contribution plot for hospital mortality and contribution of low or moderate RoB comparisons to each network estimate of hospital mortality

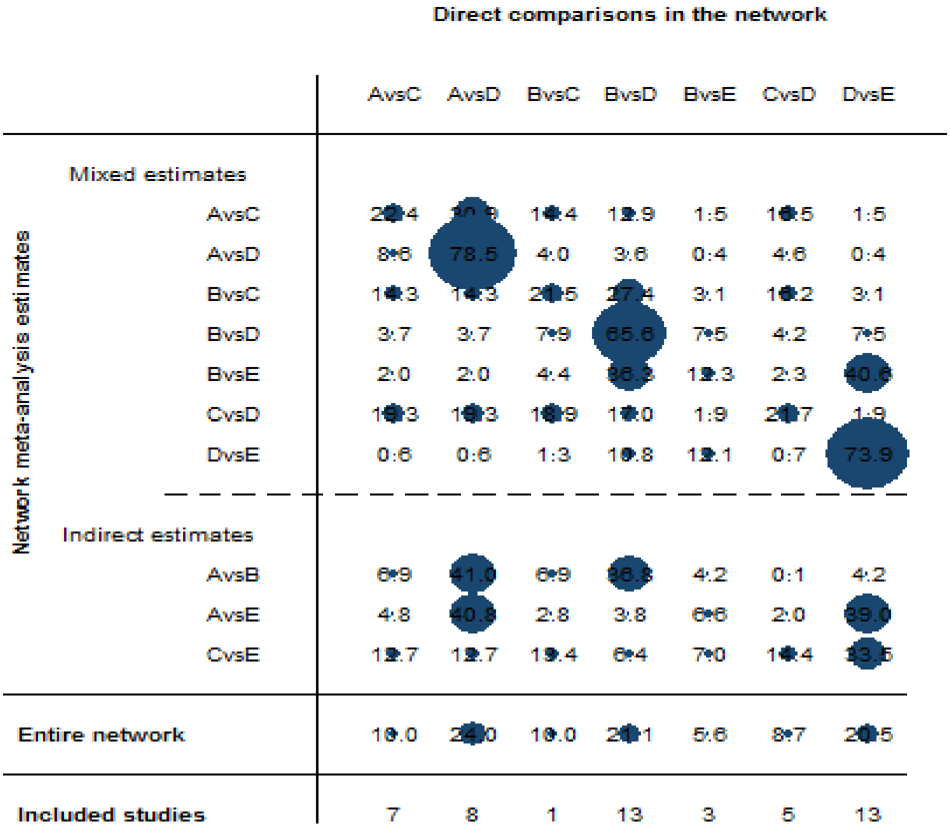

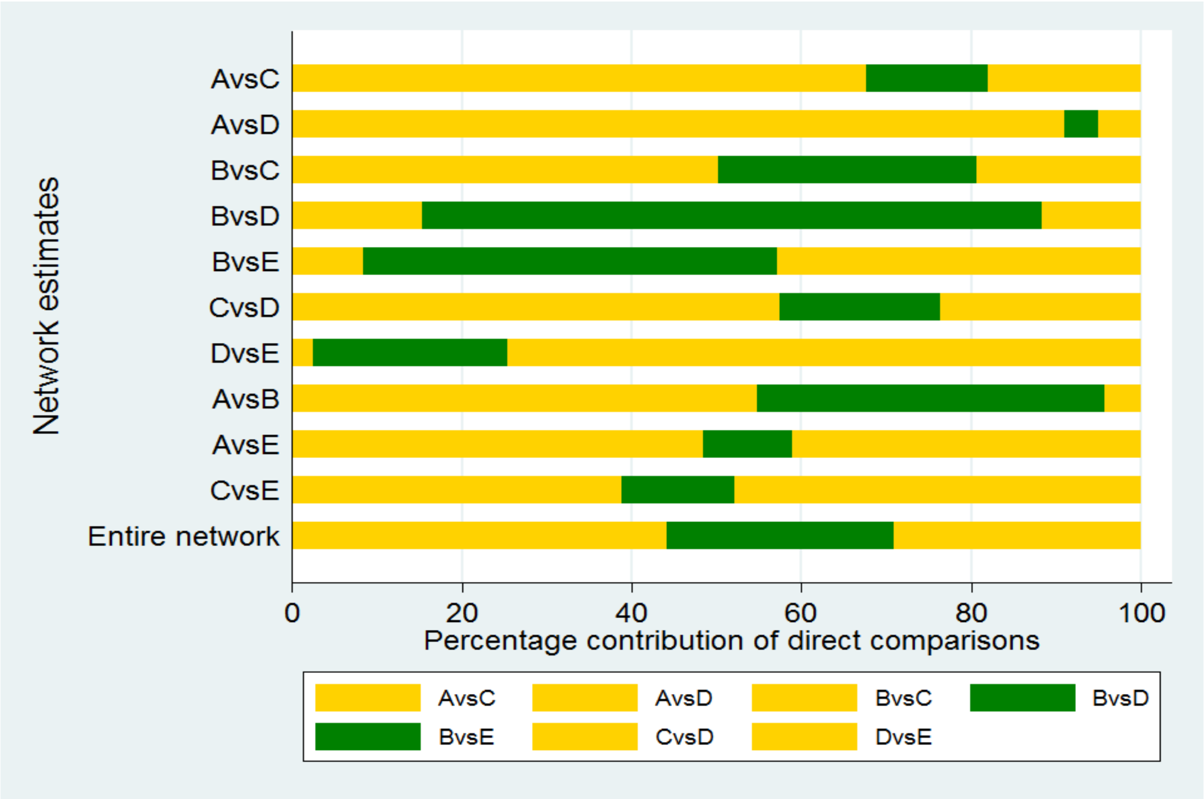

A: Synbiotics; B: Probiotics; C: Probiotics; D: EPN; E: TPN

Table S 11.9 Result of GRADE for hospital mortality

	Nature of the evidence	Study limitations	Imprecision	Inconsistency	Indirectness	Publication bias	Cinfidence	Downgrading due to
A vs B	Indirect estimated	No downgrade	No downgrade	No downgrade	No downgrade	No downgrade	HIGH	-
A vs C	Mixed estimated	Downgrade because > 70% contribution from moderate Rob comparisons	Downgrade because point estimate < 1.0 but upper limit >1.25	No downgrade	No downgrade	No downgrade	LOW	Study limitations Imprecision
A vs D	Mixed estimated	Downgrade because > 70% contribution from moderate Rob comparisons	Downgrade because point estimate < 1.0 but upper limit >1.25	No downgrade	No downgrade	No downgrade	LOW	Study limitations Imprecision
A vs E	Indirect estimated	Downgrade because > 70% contribution from moderate Rob comparisons	No downgrade	No downgrade	No downgrade	No downgrade	MODERATE	Study limitations
B vs C	Mixed estimated	No downgrade	Downgrade because point estimate >1.0 but lower limit<0.80	No downgrade	No downgrade	No downgrade	MODERATE	Imprecision
B vs D	Mixed estimated	No downgrade	Downgrade because point estimate >1.0 but lower limit<0.80	No downgrade	No downgrade	No downgrade	MODERATE	Imprecision
B vs E	Mixed estimated	No downgrade	No downgrade	No downgrade	No downgrade	No downgrade	MODERATE	-
C vs D	Mixed estimated	Downgrade because > 70% contribution from moderate Rob comparisons	Downgrade because point estimate < 1.0 but upper limit >1.25	No downgrade	No downgrade	No downgrade	LOW	Study limitations Imprecision
C vs E	Indirect estimated	Downgrade because > 70% contribution from moderate Rob comparisons	No downgrade	No downgrade	No downgrade	No downgrade	MODERATE	Study limitations
D vs E	Mixed estimated	Downgrade because > 70% contribution from moderate Rob comparisons	No downgrade	Downgrade because pair heterogeneity I^2=72.2%	No downgrade	No downgrade	LOW	Study limitations Inconsistency-
Ranking of treatments		Downgrade because > 70% contribution from moderate Rob comparisons	No downgrade	No downgrade	No downgrade	No downgrade	MODERATE	Study limitations

A: Synbiotics; B: Probiotics; C: Probiotics; D: EPN; E: TPN

Figure S 11.10 Contribution plot for ICU mortality and of low or moderate RoB comparisons to each network estimate of ICU mortality

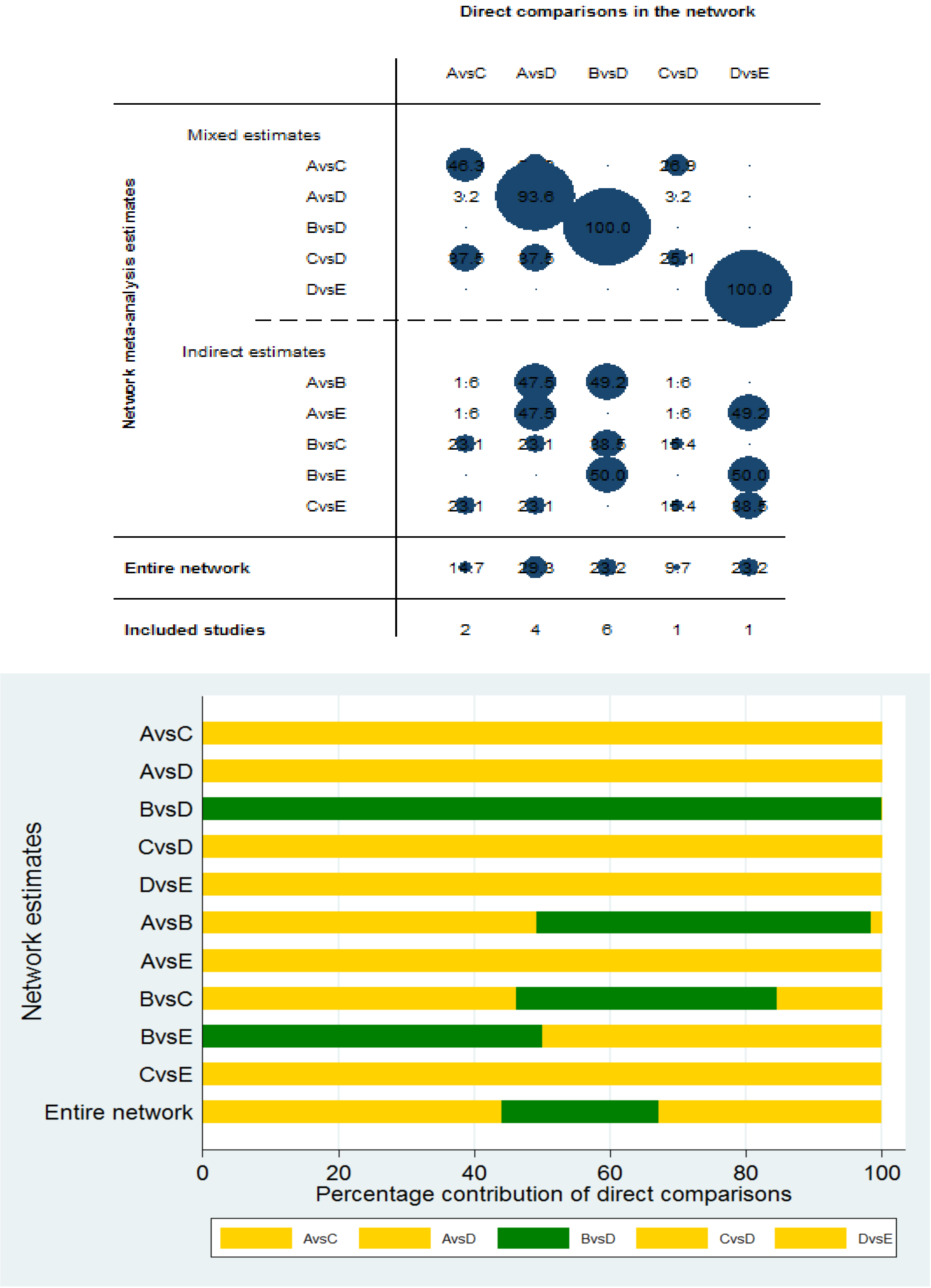

A: Synbiotics; B: Probiotics; C: Probiotics; D: EPN; E: TPN

Table S 11.10 Result of GRADE for ICU mortality

	Nature of the evidence	Study limitations	Imprecision	Inconsistency	Indirectness	Publication bias	Confidence	Downgrading due to
A vs B	Indirect estimated	No downgrade	No downgrade	No downgrade	No downgrade	No downgrade	MODERATE	Imprecision
A vs C	Mixed estimated	Downgrade because >70% contribution from moderate Rob comparisons	Downgrade because point estimate >1.0 but lower limit<0.80	No downgrade	No downgrade	No downgrade	LOW	Study limitations Imprecision
A vs D	Mixed estimated	Downgrade because >70% contribution from moderate Rob comparisons	Downgrade because point estimate < 1.0 but upper limit >1.25	No downgrade	No downgrade	No downgrade	LOW	Study limitations Imprecision
A vs E	Indirect estimated	Downgrade because >70% contribution from moderate Rob comparisons	No downgrade	No downgrade	No downgrade	No downgrade	MODERATE	Study limitations
B vs C	Indirect estimated	No downgrade	Downgrade because point estimate >1.0 but lower limit<0.80	No downgrade	No downgrade	No downgrade	MODERATE	Imprecision
B vs D	Mixed estimated	No downgrade	Downgrade because point estimate >1.0 but lower limit<0.80	No downgrade	No downgrade	No downgrade	MODERATE	Imprecision
B vs E	Indirect estimated	No downgrade	No downgrade	No downgrade	No downgrade	No downgrade	HIGH	-
C vs D	Mixed estimated	Downgrade because >70% contribution from moderate Rob comparisons	Downgrade because point estimate < 1.0 but upper limit >1.25	No downgrade	No downgrade	No downgrade	LOW	Study limitations Imprecision
C vs E	Indirect estimated	Downgrade because >70% contribution from moderate Rob comparisons	No downgrade	No downgrade	No downgrade	No downgrade	MODERATE	Study limitations
D vs E	Mixed estimated	Downgrade because >70% contribution from moderate Rob comparisons	No downgrade	No downgrade	No downgrade	No downgrade	MODERATE	Study limitations
Ranking of treatments		Downgrade because >70% contribution from moderate Rob comparisons	No downgrade	No downgrade	No downgrade	No downgrade	MODERATE	Study limitations

A: Synbiotics; B: Probiotics; C: Probiotics; D: EPN; E: TPN

Figure S 11.11 Contribution plot for hospital LOS and of low or moderate RoB comparisons to each network estimate of hospital LOS

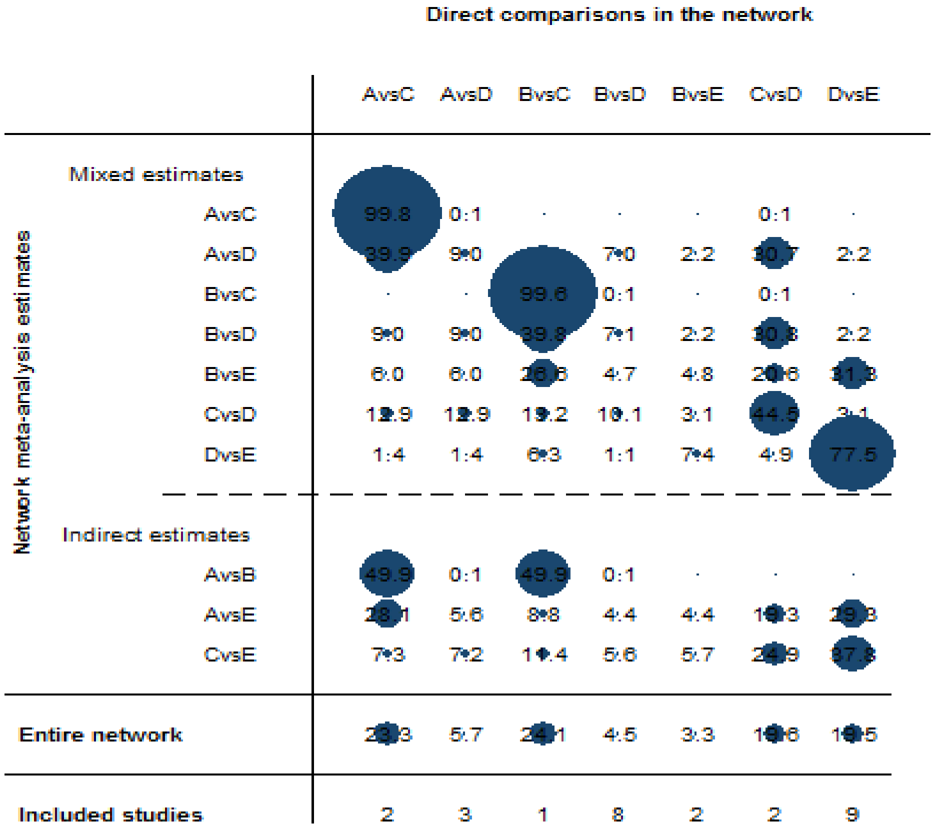

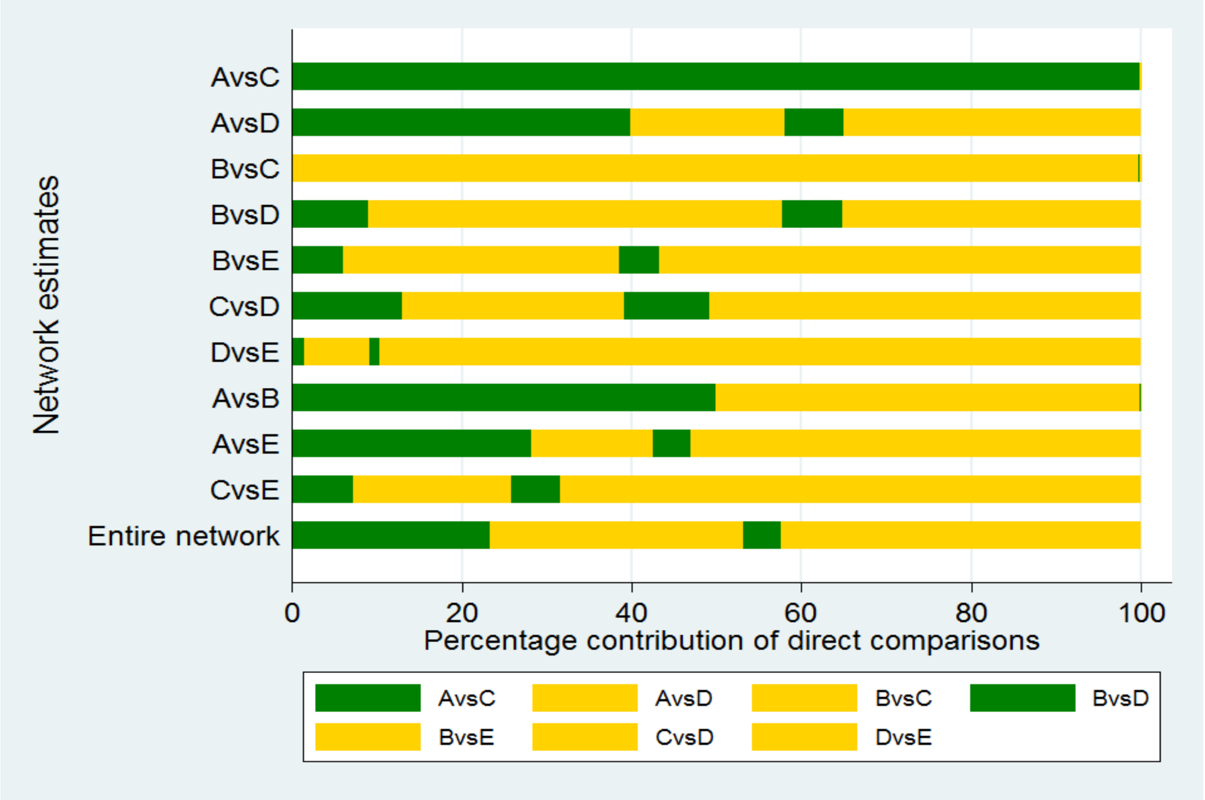

A: Synbiotics; B: Probiotics; C: Probiotics; D: EPN; E: TPN

Table S 11.11 Result of GRADE for hospital LOS

	Nature of the evidence	Study limitations	Imprecision	Inconsistency	Indirectness	Publication bias	Confidence	Downgrading due to
A vs B	Indirect estimated	No downgrade	Downgrade because confidence interval crosses null value or includes values favoring either treatment	No downgrade	No downgrade	No downgrade	MODERATE	Imprecision
A vs C	Mixed estimated	No downgrade	Downgrade because confidence interval crosses null value or includes values favoring either treatment	No downgrade	No downgrade	No downgrade	MODERATE	Imprecision
A vs D	Mixed estimated	No downgrade	Downgrade because confidence interval crosses null value or includes values favoring either treatment	No downgrade	No downgrade	No downgrade	MODERATE	Imprecision
A vs E	Indirect estimated	No downgrade	No downgrade	No downgrade	No downgrade	No downgrade	MODERATE	Study limitations
B vs C	Mixed estimated	Downgrade because >70% contribution from moderate Rob comparisons	Downgrade because confidence interval crosses null value or includes values favoring either treatment	No downgrade	No downgrade	No downgrade	LOW	Study limitations Imprecision
B vs D	Mixed estimated	Downgrade because >70% contribution from moderate Rob comparisons	Downgrade because confidence interval crosses null value or includes values favoring either treatment	Downgrade because pair heterogeneity I^2=76. 8%	No downgrade	No downgrade	VERY LOW	Study limitations Imprecision Inconsistency
B vs E	Mixed estimated	Downgrade because >70% contribution from moderate Rob comparisons	No downgrade	Downgrade because pair heterogeneity I^2=85.7% Downgrade because side splitting p=0.0452	No downgrade	No downgrade	VERY LOW	Study limitations Inconsistency
C vs D	Mixed estimated	Downgrade because >70% contribution from moderate Rob comparisons	Downgrade because confidence interval crosses null value or includes values favoring either treatment	No downgrade	No downgrade	No downgrade	LOW	Study limitations Imprecision
C vs E	Indirect estimated	Downgrade because >70% contribution from moderate Rob comparisons	Downgrade because confidence interval crosses null value or includes values favoring either treatment	No downgrade	No downgrade	No downgrade	LOW	Study limitations Imprecision
D vs E	Mixed estimated	Downgrade because >70% contribution from moderate Rob comparisons	No downgrade	Downgrade because pair heterogeneity I^2=99.1%	No downgrade	No downgrade	LOW	Study limitations Inconsistency
Ranking of treatments		Downgrade because >70% contribution from moderate Rob comparisons	Downgrade because probabilities are similarly distributed across the ranks	Downgrade because global heterogeneity I^2=98.56%	No downgrade	Downgrade because asymmetric comparison adjusted funnel plot	VERY LOW	Study limitations Imprecision Inconsistency Publication bias

A: Synbiotics; B: Probiotics; C: Probiotics; D: EPN; E: TPN

Figure S 11.12 Contribution plot for ICU LOS and of low or moderate RoB comparisons to each network estimate of ICU LOS

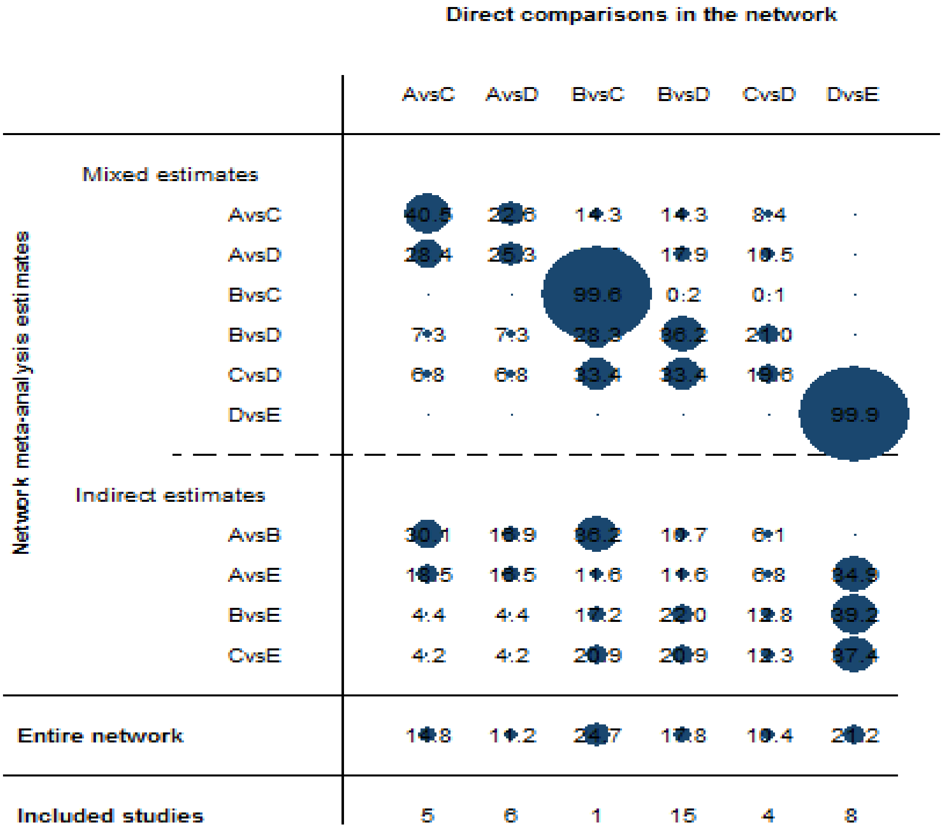

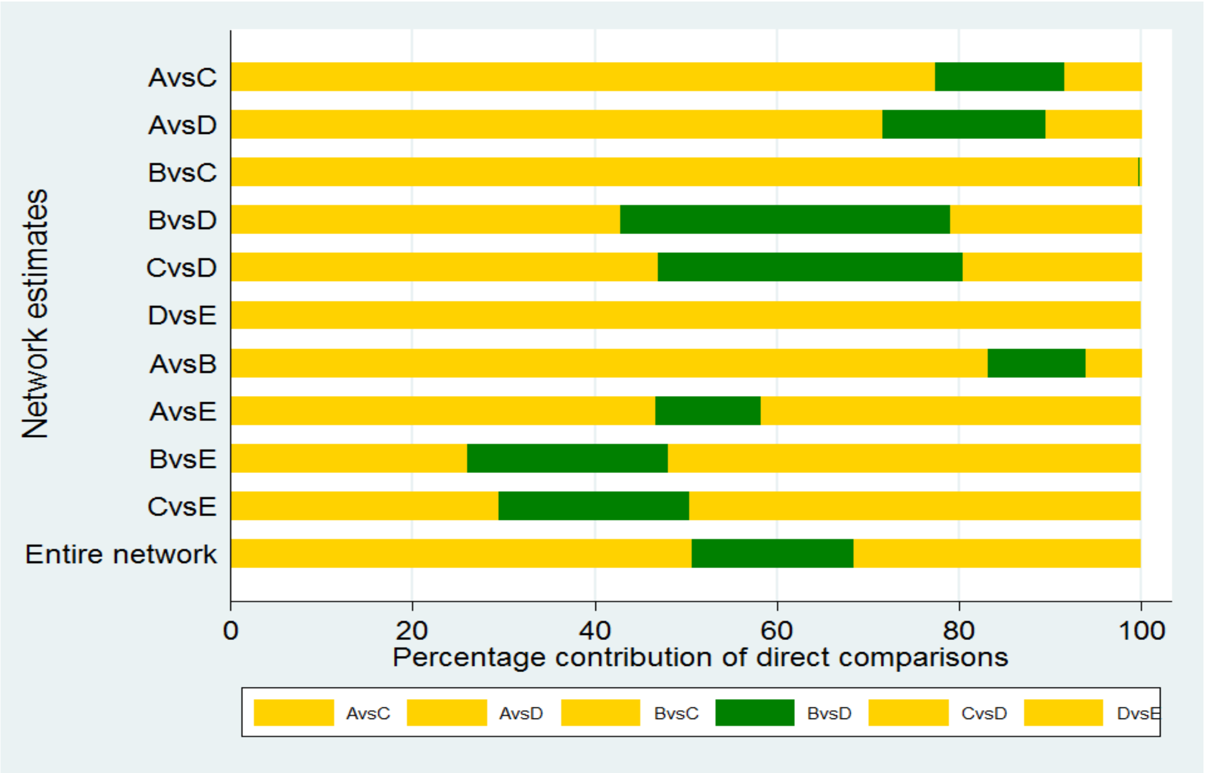

A: Synbiotics; B: Probiotics; C: Probiotics; D: EPN; E: TPN

Table S 11.12 Result of GRADE for ICU LOS

	Nature of the evidence	Study limitations	Imprecision	Inconsistency	Indirectness	Publication bias	Confidence	Downgrading due to
A vs B	Indirect estimated	Downgrade because ＞70% contribution from moderate Rob comparisons	Downgrade because confidence interval crosses null value or includes values favoring either treatment	No downgrade	No downgrade	No downgrade	LOW	Study limitations Imprecision
A vs C	Mixed estimated	Downgrade because ＞70% contribution from moderate Rob comparisons	Downgrade because confidence interval crosses null value or includes values favoring either treatment	No downgrade	No downgrade	No downgrade	LOW	Study limitations Imprecision
A vs D	Mixed estimated	Downgrade because ＞70% contribution from moderate Rob comparisons	Downgrade because confidence interval crosses null value or includes values favoring either treatment	Downgrade because pair heterogeneity I^2=76.3%	No downgrade	No downgrade	VERY LOW	Study limitations Imprecision Inconsistency
A vs E	Indirect estimated	Downgrade because ＞70% contribution from moderate Rob comparisons	Downgrade because confidence interval crosses null value or includes values favoring either treatment	No downgrade	No downgrade	No downgrade	LOW	Study limitations Imprecision
B vs C	Mixed estimated	Downgrade because ＞70% contribution from moderate Rob comparisons	Downgrade because confidence interval crosses null value or includes values favoring either treatment	No downgrade	No downgrade	No downgrade	LOW	Imprecision
B vs D	Mixed estimated	No downgrade	Downgrade because confidence interval crosses null value or includes values favoring either treatment	Downgrade because pair heterogeneity I^2=80.1%	No downgrade	No downgrade	LOW	Imprecision Inconsistency
B vs E	Indirect estimated	Downgrade because ＞70% contribution from moderate Rob comparisons	No downgrade	No downgrade	No downgrade	No downgrade	MODERATE	Study limitations
C vs D	Mixed estimated	No downgrade	Downgrade because confidence interval crosses null value or includes values favoring either treatment	Downgrade because pair heterogeneity I^2=60.9%	No downgrade	No downgrade	LOW	Imprecision Inconsistency
C vs E	Indirect estimated	Downgrade because ＞70% contribution from moderate Rob comparisons	Downgrade because confidence interval crosses null value or includes values favoring either treatment	No downgrade	No downgrade	No downgrade	LOW	Study limitations Imprecision
D vs E	Mixed estimated	Downgrade because ＞70% contribution from moderate Rob comparisons	Downgrade because confidence interval crosses null value or includes values favoring either treatment	Downgrade because pair heterogeneity I^2=89.7%	No downgrade	No downgrade	VERY LOW	Study limitations Inconsistency
Ranking of treatments		Downgrade because ＞70% contribution from moderate Rob comparisons	Downgrade because probabilities are similarly distributed across the ranks	Downgrade because global heterogeneity I^2=79.47%	No downgrade	Downgrade because asymmetric comparison adjusted funnel plot	VERY LOW	Study limitations Imprecision Inconsistency Publication bias

A: Synbiotics; B: Probiotics; C: Probiotics; D: EPN; E: TPN

Figure S 11.13 Contribution plot for MV and of low or moderate RoB comparisons to each network estimate of MV

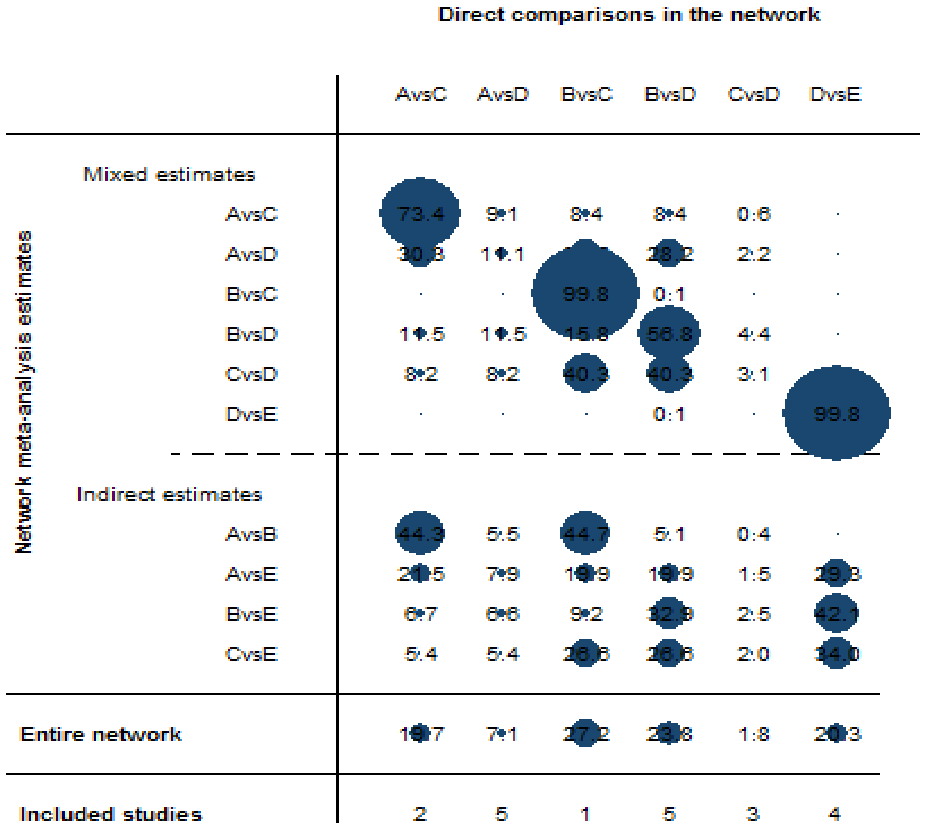

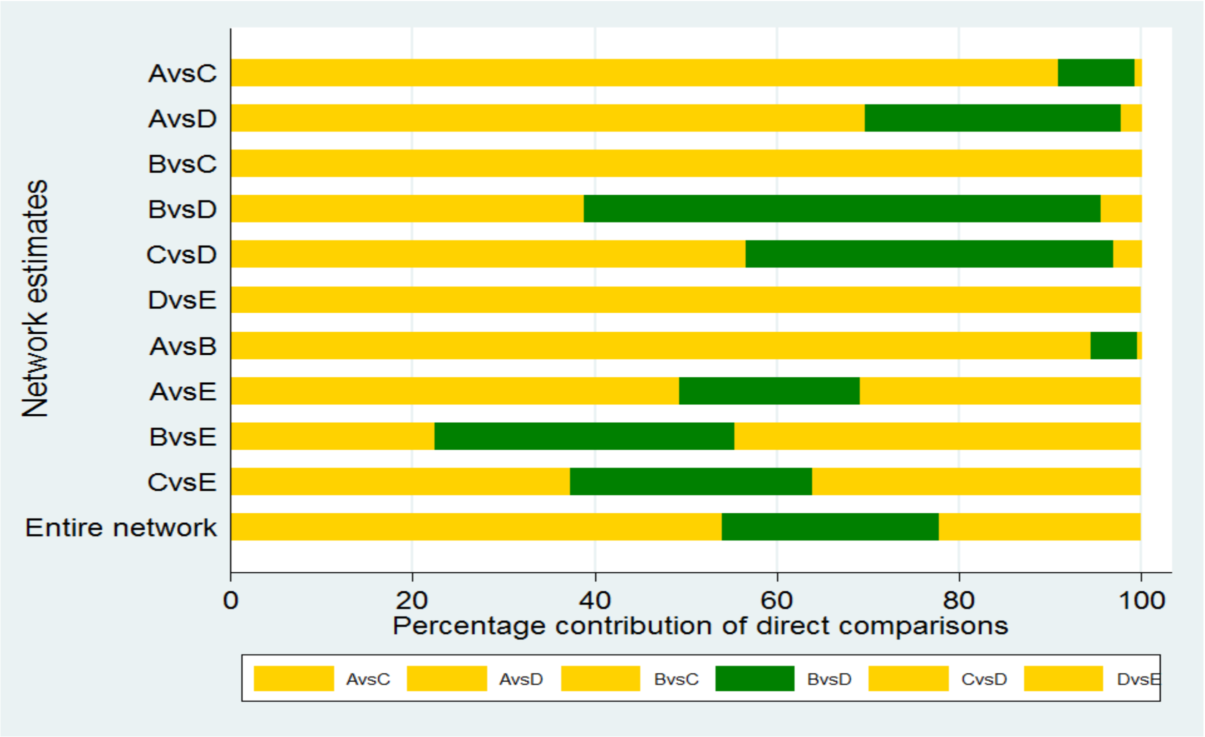

A: Synbiotics; B: Probiotics; C: Probiotics; D: EPN; E: TPN

Table S 11.13 Result of GRADE for MV

	Nature of the evidence	Study limitations	Imprecision	Inconsistency	Indirectness	Publication bias	Confidence	Downgrading due to
A vs B	Indirect estimated	Downgrade because > 70% contribution from moderate Rob comparisons	Downgrade because confidence interval crosses null value or includes values favoring either treatment	No downgrade	No downgrade	No downgrade	LOW	Study limitations Imprecision
A vs C	Mixed estimated	Downgrade because > 70% contribution from moderate Rob comparisons	Downgrade because confidence interval crosses null value or includes values favoring either treatment	No downgrade	No downgrade	No downgrade	LOW	Study limitations Imprecision
A vs D	Mixed estimated	Downgrade because > 70% contribution from moderate Rob comparisons	Downgrade because confidence interval crosses null value or includes values favoring either treatment	Downgrade because pair heterogeneity I^2=887%	No downgrade	No downgrade	VERY LOW	Study limitations Imprecision Inconsistency
A vs E	Indirect estimated	Downgrade because > 70% contribution from moderate Rob comparisons	No downgrade	No downgrade	No downgrade	No downgrade	MODERATE	Study limitations
B vs C	Mixed estimated	Downgrade because > 70% contribution from moderate Rob comparisons	Downgrade because confidence interval crosses null value or includes values favoring either treatment	No downgrade	No downgrade	No downgrade	MODERATE	Study limitations Imprecision
B vs D	Mixed estimated	No downgrade	No downgrade	No downgrade	No downgrade	No downgrade	HIGH	-
B vs E	Indirect estimated	No downgrade	No downgrade	No downgrade	No downgrade	No downgrade	HIGH	-
C vs D	Mixed estimated	No downgrade	Downgrade because confidence interval crosses null value or includes values favoring either treatment	Downgrade because pair heterogeneity I^2=89.5%	No downgrade	No downgrade	LOW	Imprecision Inconsistency
C vs E	Indirect estimated	Downgrade because > 70% contribution from moderate Rob comparisons	No downgrade	No downgrade	No downgrade	No downgrade	MODERATE	Study limitations
D vs E	Mixed estimated	Downgrade because > 70% contribution from moderate Rob comparisons	Downgrade because confidence interval crosses null value or includes values favoring either treatment	Downgrade because pair heterogeneity I^2=91.3%	No downgrade	No downgrade	VERY LOW	Study limitations Imprecision Inconsistency
Ranking of treatment s		Downgrade because > 70% contribution from moderate Rob comparisons	No downgrade	Downgrade because global heterogeneity I^2=86.1%	No downgrade	Downgrade because asymmetric comparison adjusted funnel plot	VERY LOW	Study limitations Inconsistency Publication bias

A: Synbiotics; B: Probiotics; C: Probiotics; D: EPN; E: TPN

Appendix file 12
Treatment ranking and surface under the cumulative ranking curves (SUCRA) for each outcome

Figure S 12.1 Treatment ranking and SUCRA ranking curve for nosocomial infection

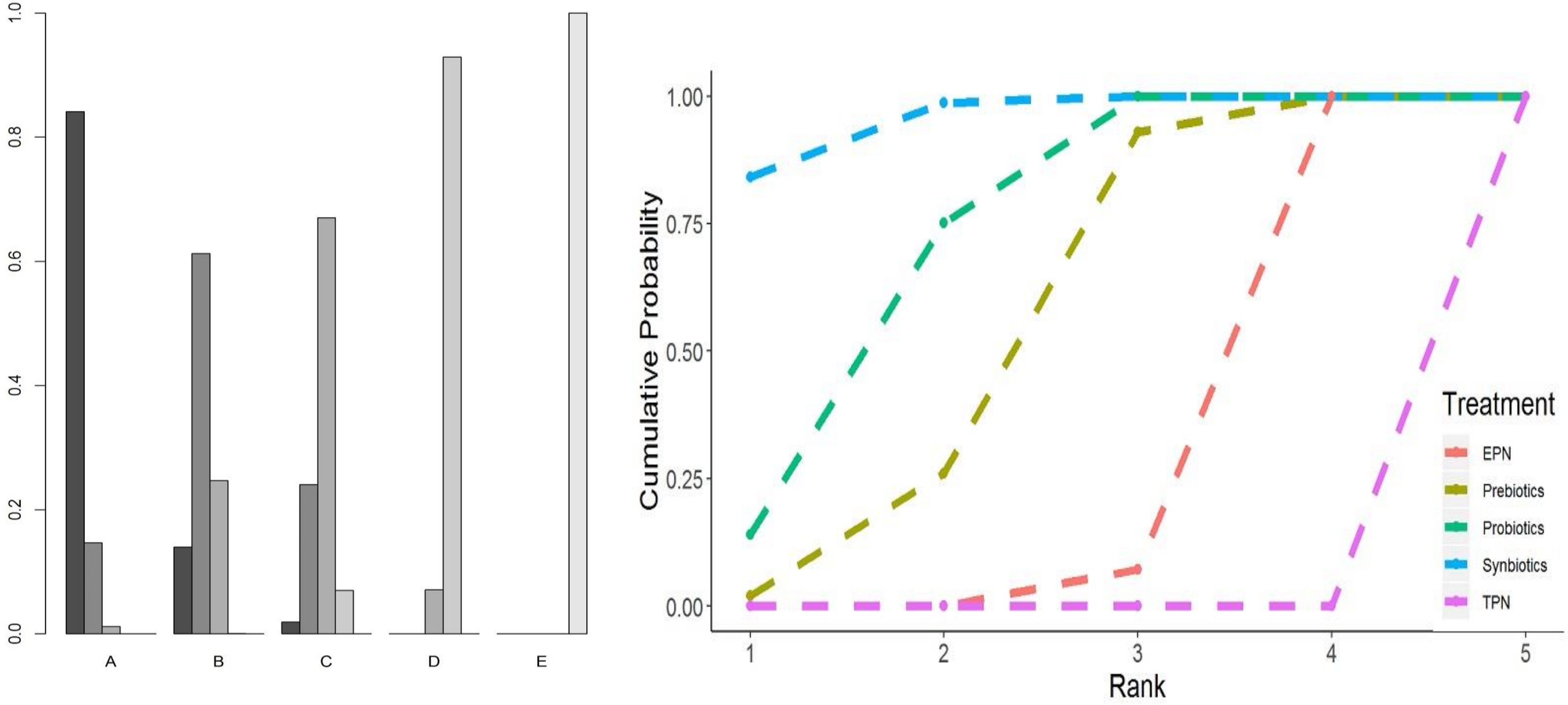

A: Synbiotics; B: Probiotics; C: Probiotics; D: EPN; E: TPN

Figure S 12.2 Treatment ranking and SUCRA ranking curve for hospital acquired pneumonia

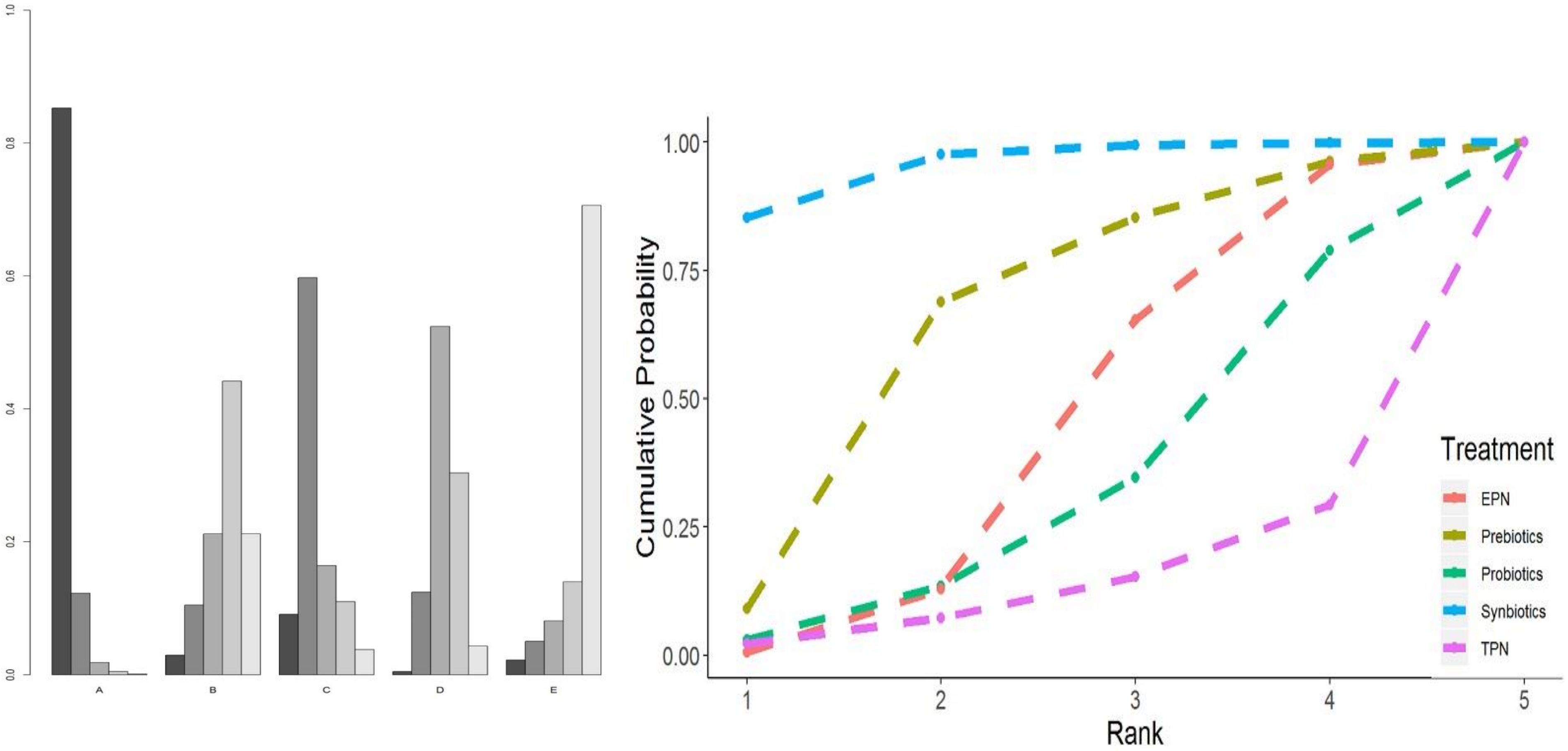

A: Synbiotics; B: Probiotics; C: Probiotics; D: EPN; E: TPN

Figure S 12.3 Treatment ranking and SUCRA ranking curve for ventilator-associated pneumonia

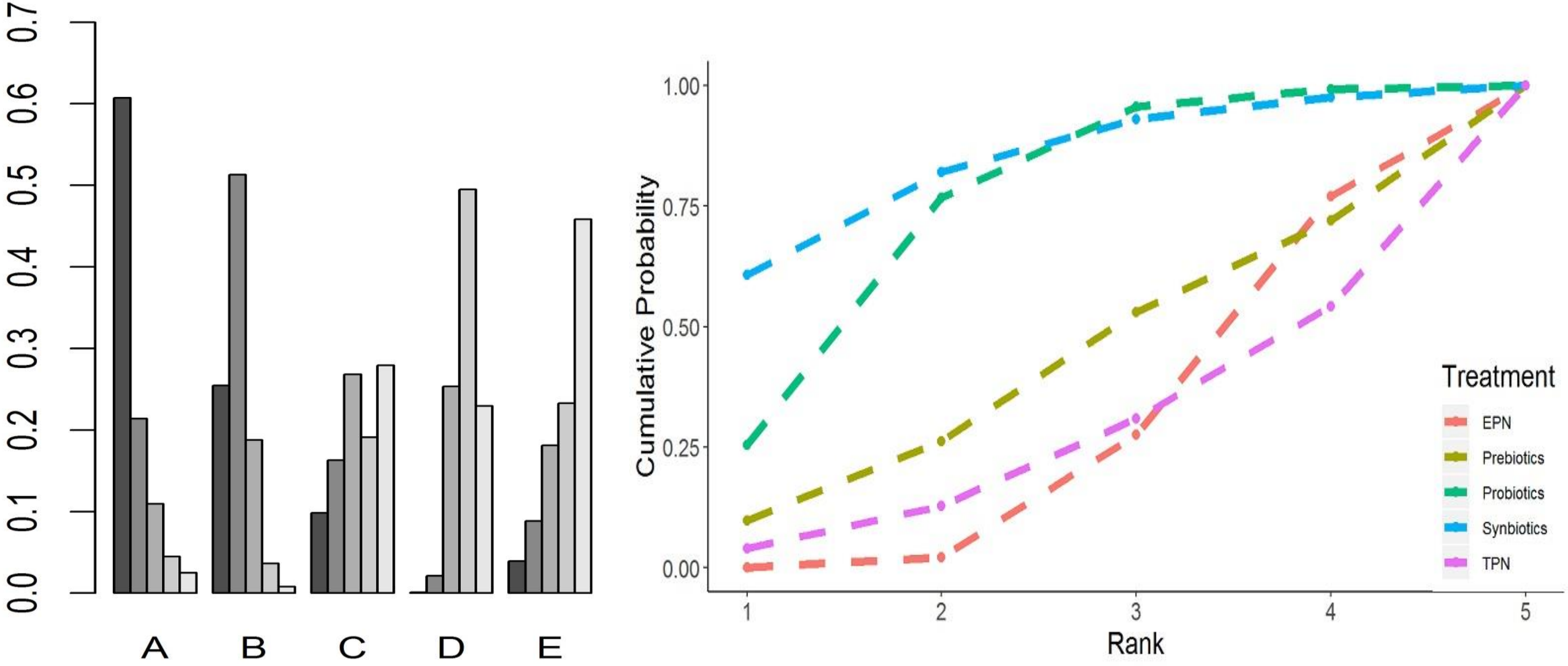

A: Synbiotics; B: Probiotics; C: Probiotics; D: EPN; E: TPN

Figure S 12.4 Treatment ranking and SUCRA ranking curve for bloodstream infection

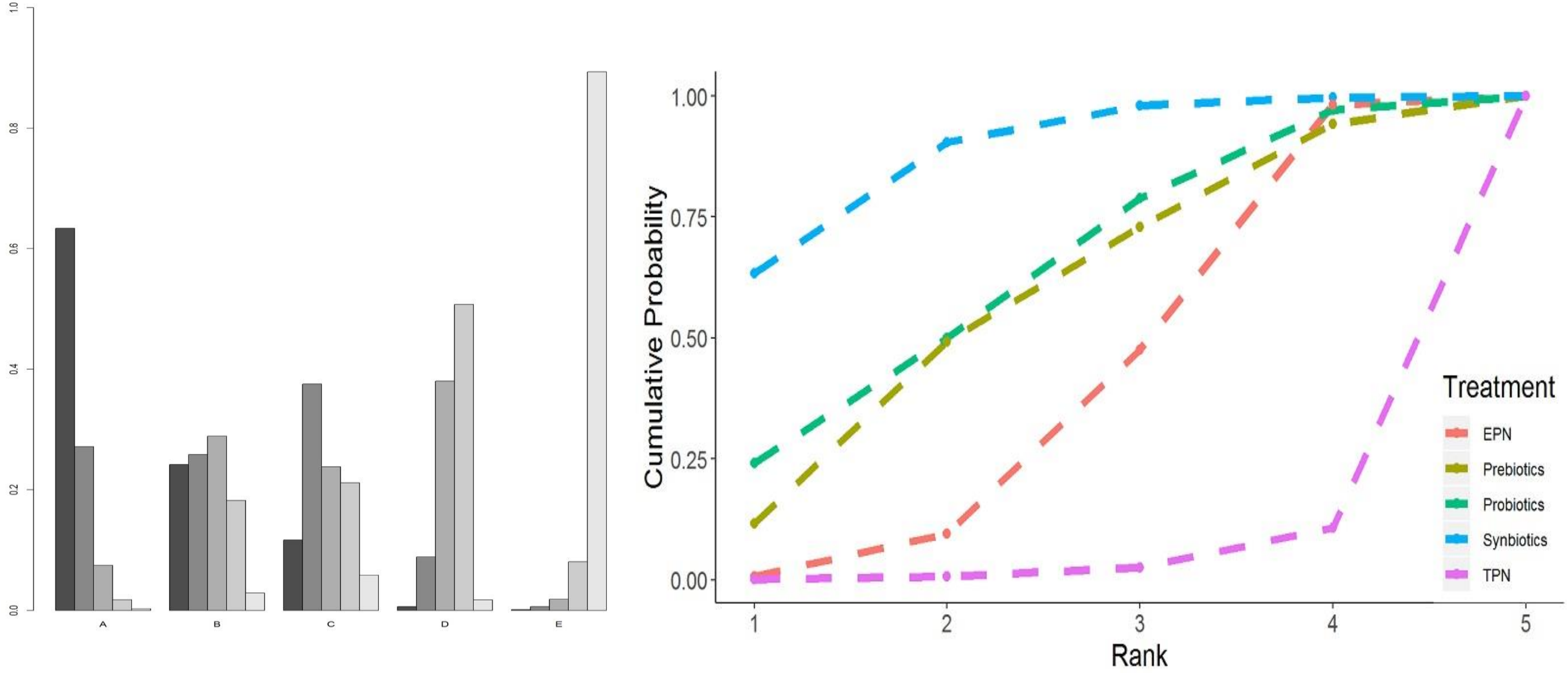

A: Synbiotics; B: Probiotics; C: Probiotics; D: EPN; E: TPN

Figure S 12.5 Treatment ranking and SUCRA ranking curve for catheter-related bloodstream infection

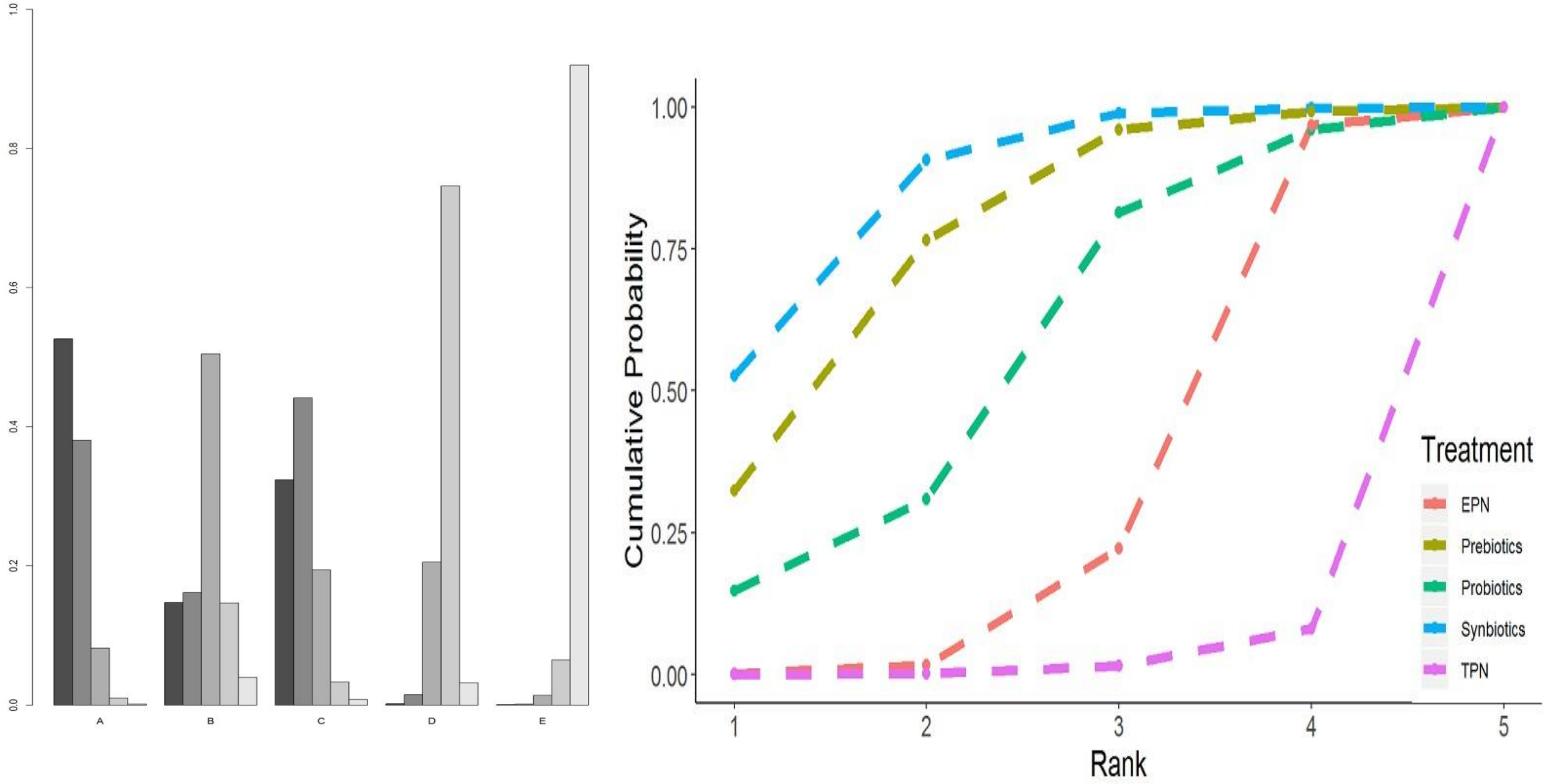

A: Synbiotics; B: Probiotics; C: Probiotics; D: EPN; E: TPN

Figure S 12.6 Treatment ranking and SUCRA ranking curve for urinary tract infections

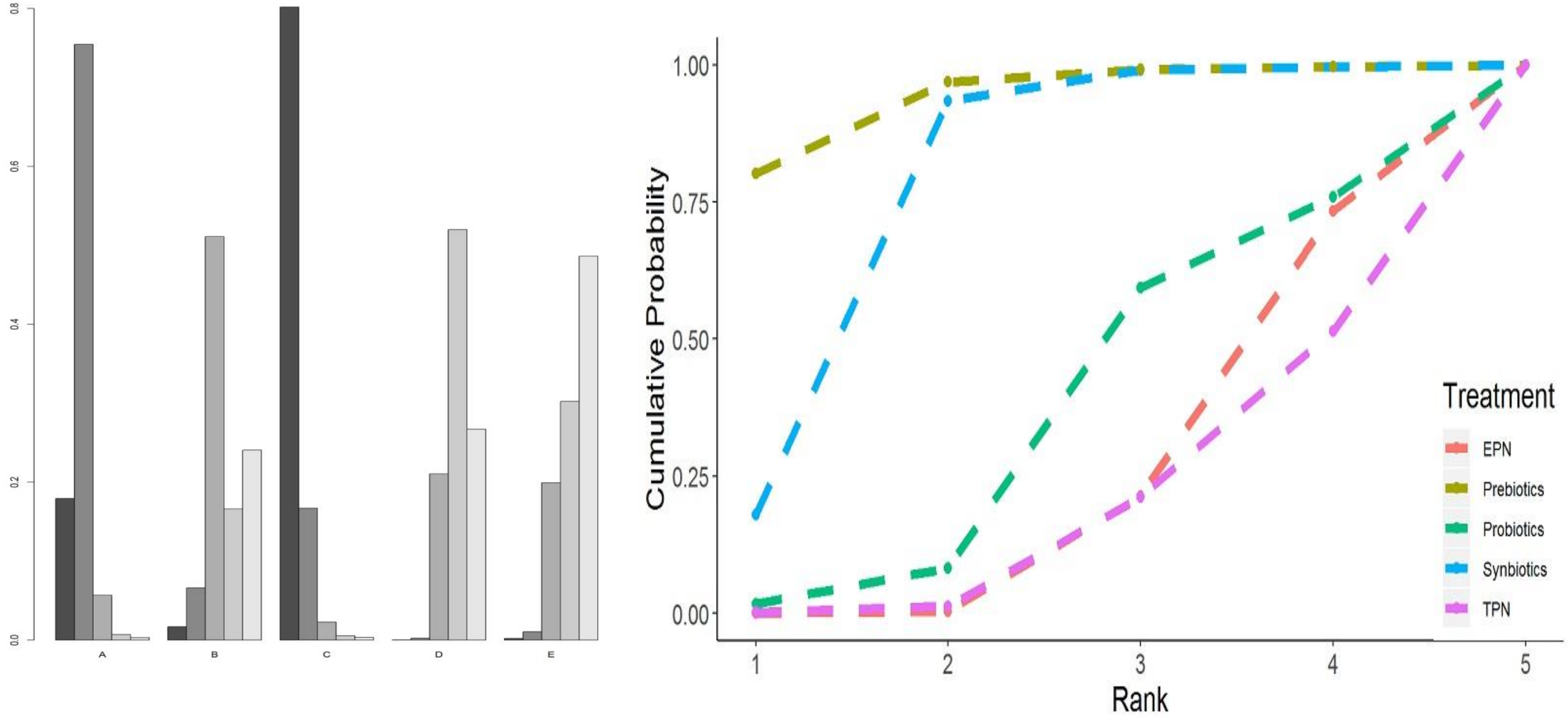

A: Synbiotics; B: Probiotics; C: Probiotics; D: EPN; E: TPN

Figure S 12.7 Treatment ranking and SUCRA ranking curve for sepsis

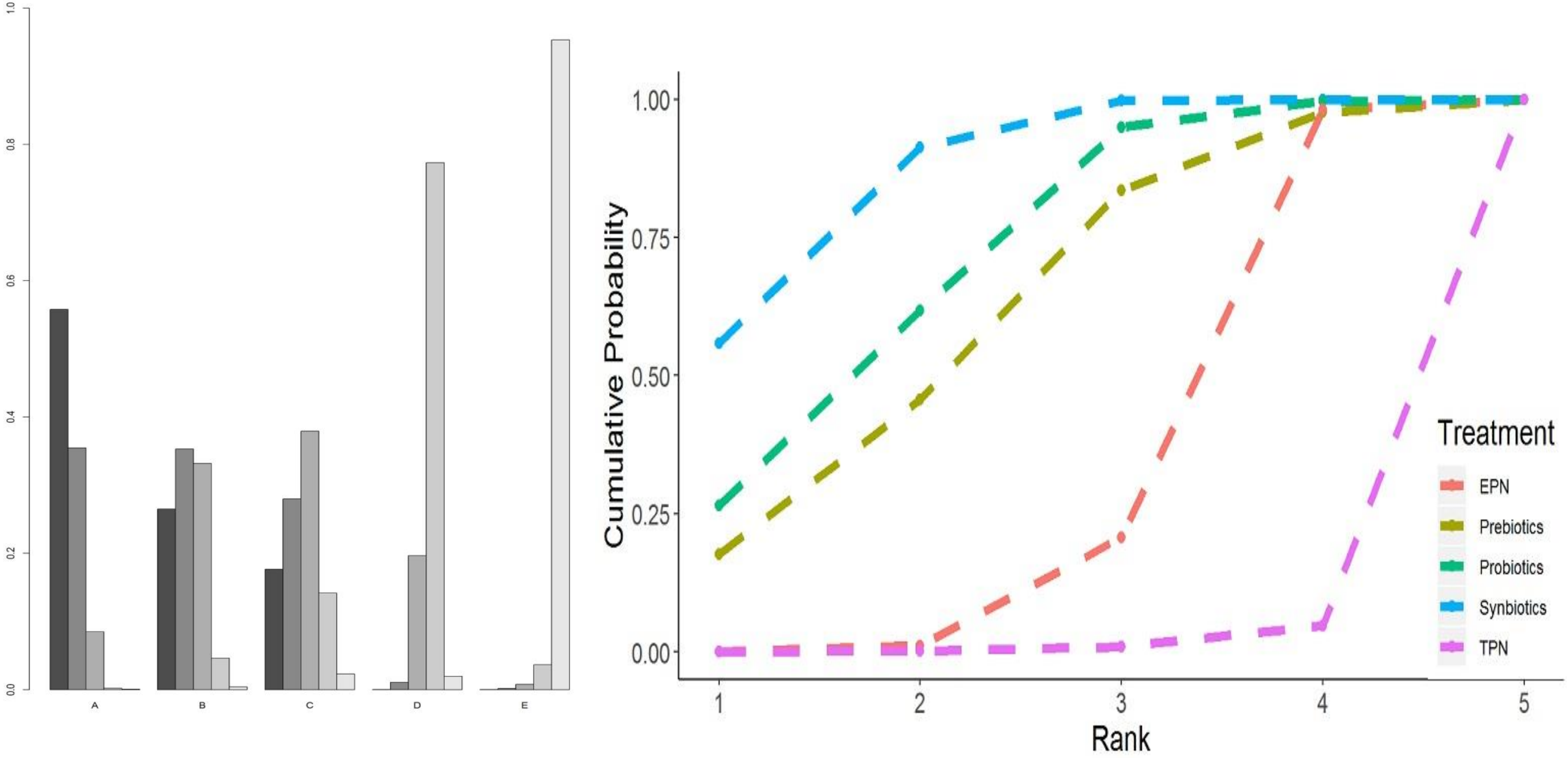

A: Synbiotics; B: Probiotics; C: Probiotics; D: EPN; E: TPN

Figure S 12.8 Treatment ranking and SUCRA ranking curve for diarrhea

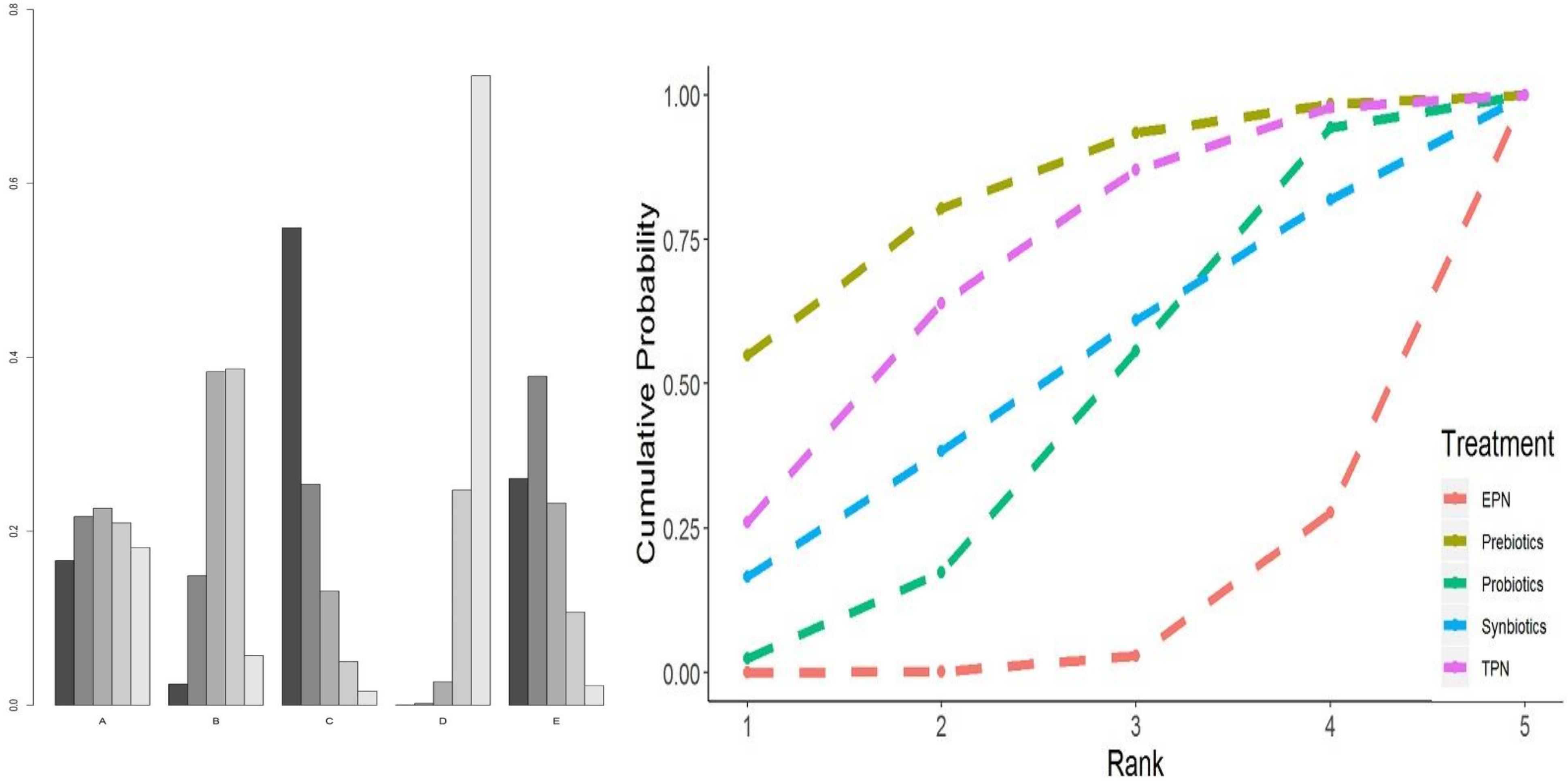

A: Synbiotics; B: Probiotics; C: Probiotics; D: EPN; E: TPN

Figure S 12.9 Treatment ranking and SUCRA ranking curve for hospital mortality

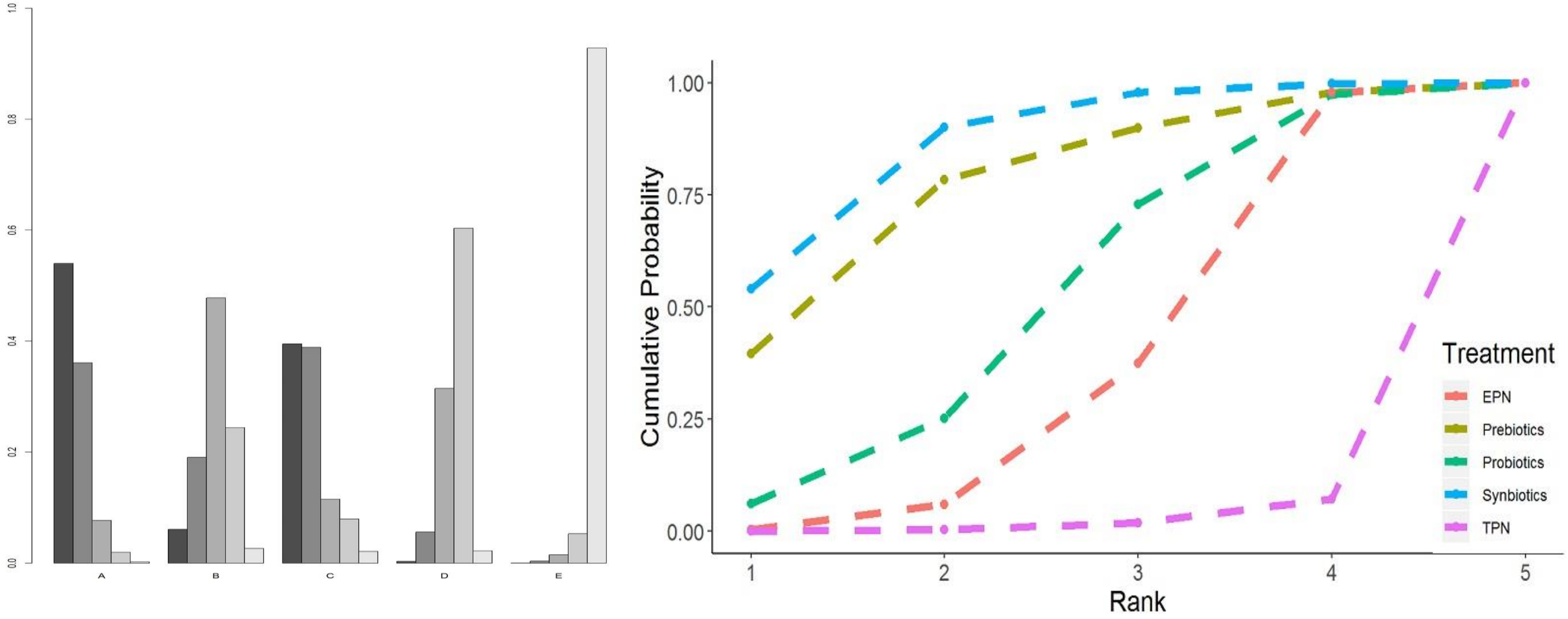

A: Synbiotics; B: Probiotics; C: Probiotics; D: EPN; E: TPN

Figure S 12.10 Treatment ranking and SUCRA ranking curve for ICU mortality

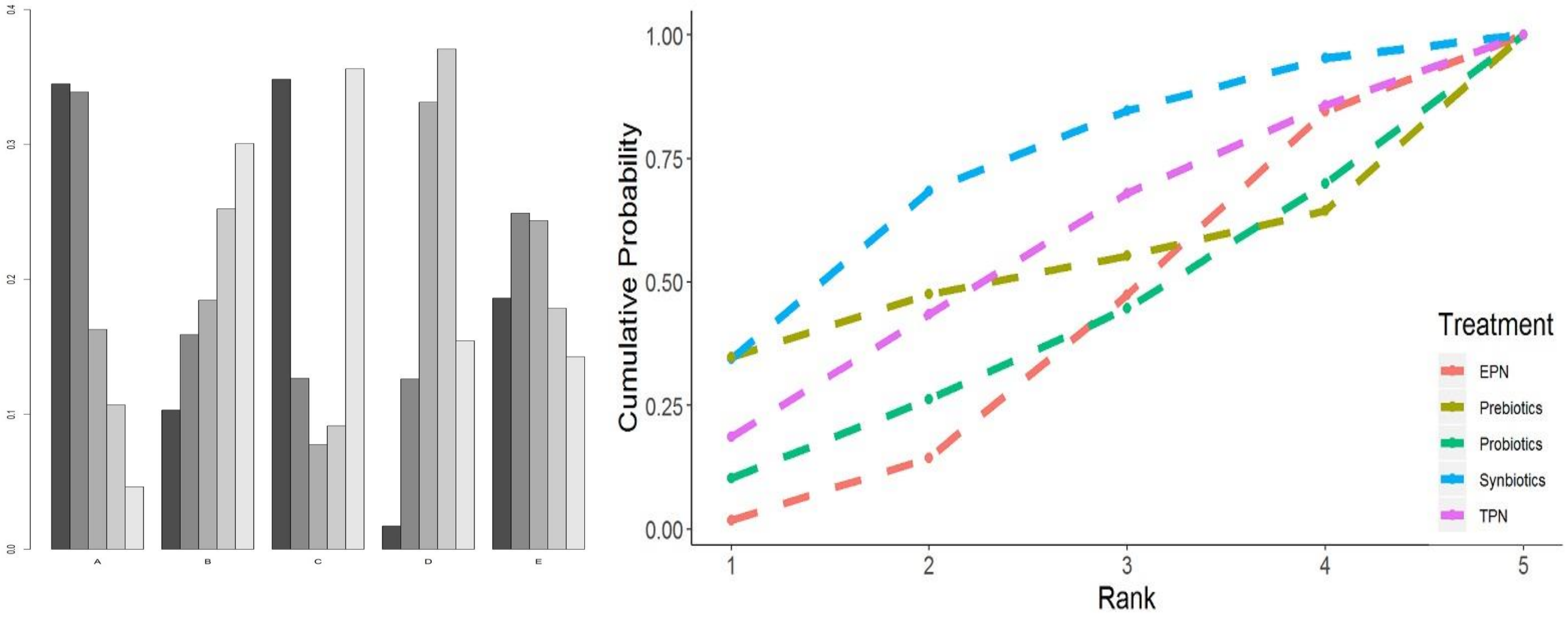

A: Synbiotics; B: Probiotics; C: Probiotics; D: EPN; E: TPN

Figure S 12.11 Treatment ranking and SUCRA ranking curve for hospital lengtn of stay

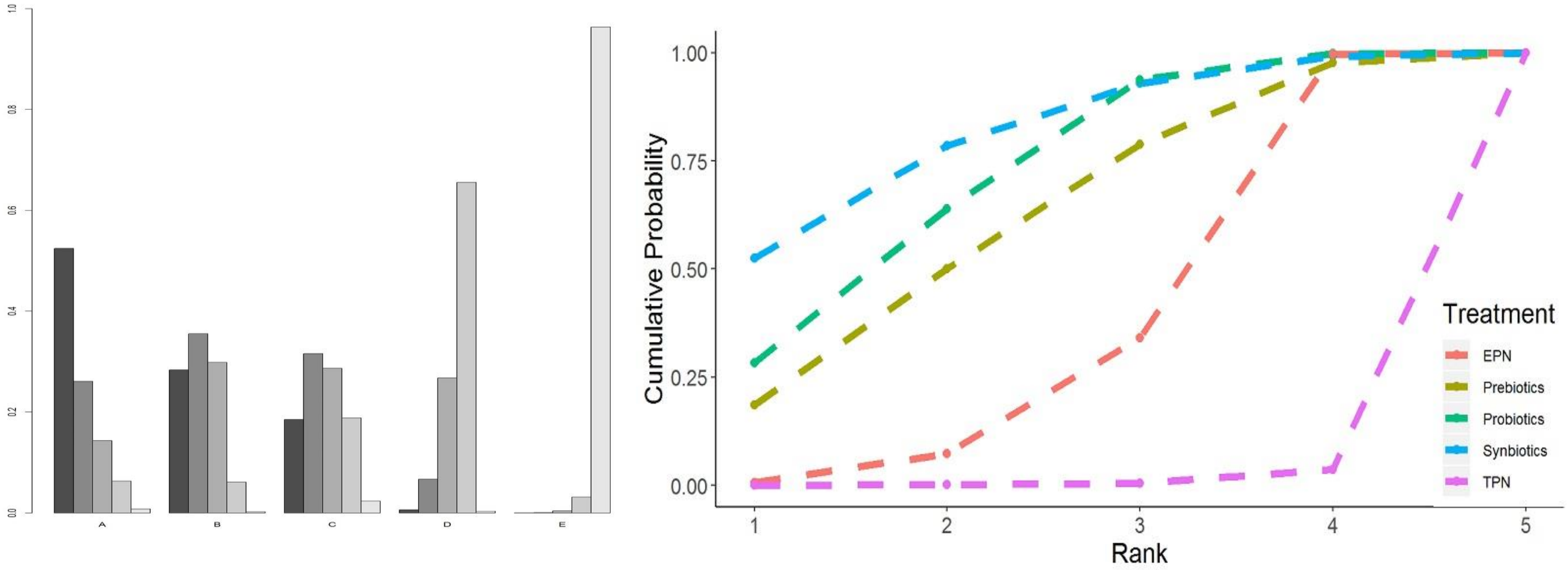

A: Synbiotics; B: Probiotics; C: Probiotics; D: EPN; E: TPN

Figure S 12.12 Treatment ranking and SUCRA ranking curve for ICU length of stay

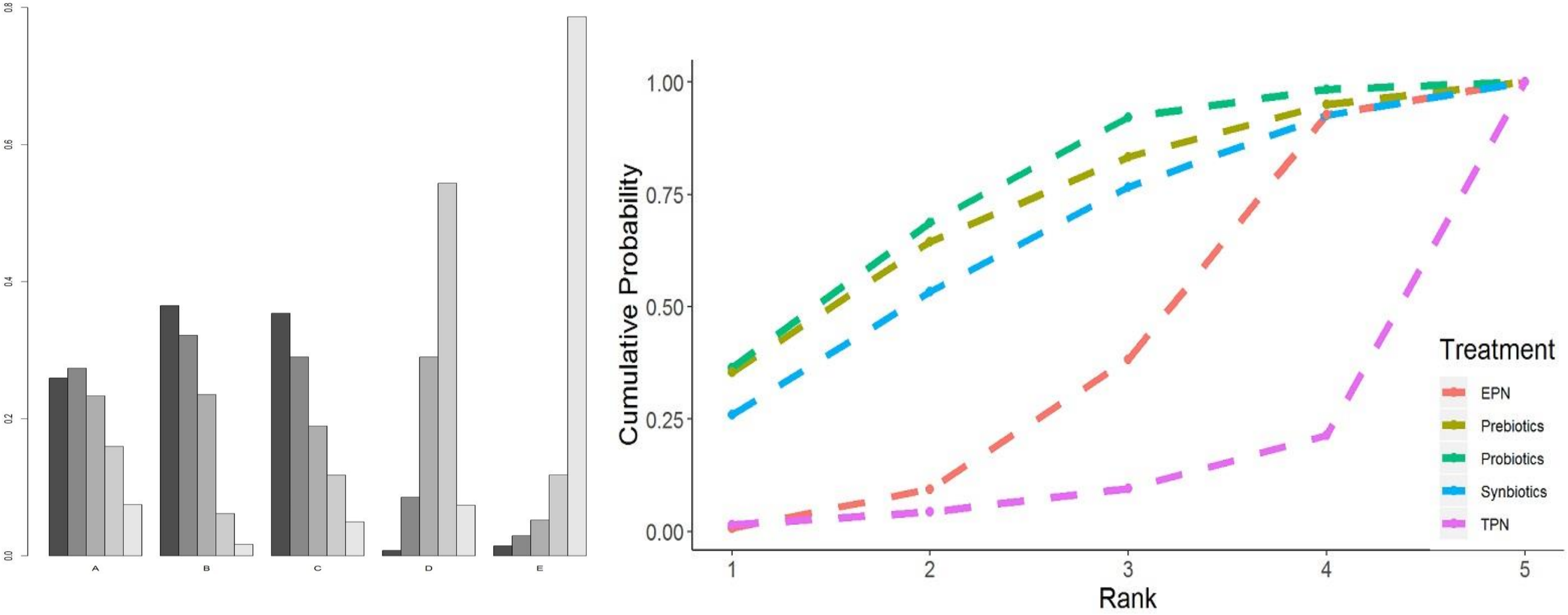

A: Synbiotics; B: Probiotics; C: Probiotics; D: EPN; E: TPN

Figure S 12.13 Treatment ranking and SUCRA ranking curve for the duration of mechanical ventilation

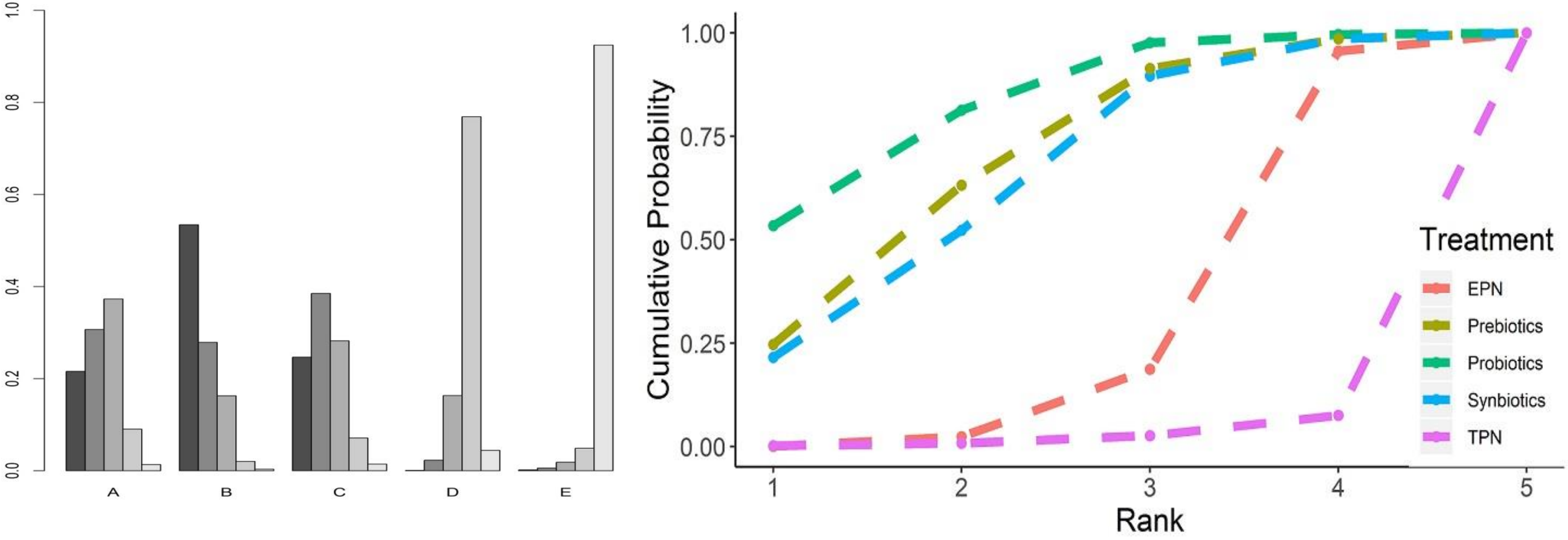

A: Synbiotics; B: Probiotics; C: Probiotics; D: EPN; E: TPN

Appendix 13
Subgroup analyses

Table S 13.1 Subgroup analyses for nosocomial infection in different times, quality and doses

Treatment	Overall patients		Studies over the last 10 years		High quality studies only (low risk of bias)		Low to moderate doses		High doses	
	OR (95% CrI)	Rank	OR (95% CrI)	Rank	OR (95% CrI)	Rank	OR (95% CrI)	Rank	OR (95% CrI)	Rank
**Synbiotics**	0.37 (0.22, 0.61)	1	0.33 (0.16, 0.64)	1	0.32 (0.13, 0.71)	1	0.36 (0.19, 0.66)	1	0.33 (0.12, 0.73)	1
**Probiotics**	0.52 (0.34, 0.77)	2	0.50 (0.31, 0.74)	2	0.42 (0.16, 1.10)	2	0.48 (0.30, 0.74)	3	0.43 (0.15, 1.20)	2
**Prebiotics**	0.65 (0.35, 1.15)	3	0.51 (0.24, 1.00)	3	0.71 (0.28, 1.60)	3	0.43 (0,19.0.94)	2	0.62 (0, 25.1.40)	3
**EPN**	Reference	4	Reference	4	Reference	4	Reference	4	Reference	4
**TPN**	2.29 (1.48, 3.67)	5	1.80 (1.10, 3.20)	5	2.20 (1.10, 4.60)	5	2.24 (1.44, 3.53)	5	2.50 (0.51, 12.00)	5
**Number of studies**	42		24		23		22		15	
**partcipants**	6215		5232		4468		2299		1102	

Abbreviations: CrI: credible interval; EPN: Enteral nutrition or adjuvant peripheral parenteral nutrition; OR: odds ratio; TPN: Total parenteral nutrition.

Table S 13.2 Subgroup analyses for hospital mortality and ICU mortality in the different initial time of nutrition therapy

	Hospital mortality						ICU mortality			
	Overall patients		Nutrition therapy within 48h		Nutrition therapy beyond 48h		Overall patients		Nutrition therapy within 48h	
	OR (95% CrI)	Rank	OR (95% CrI)	Rank	OR (95% CrI)	Rank	OR (95% CrI)	Rank	OR (95% CrI)	Rank
**Synbiotics**	0.64 (0.38, 1.02)	1	0.65 (0.37, 1.04)	1	0.76 (0.04, 12.57)	3	0.78 (0.43, 1.38)	1	0.79 (0.44, 1.39)	1
**Probiotics**	0.90 (0.60, 1.31)	3	0.91 (0.60, 1.31)	3	0.75 (0.01, 19.2)	2	1.03 (0.56, 1.81)	5	1.04 (0.57, 1.83)	5
**Prebiotics**	0.68 (0.35, 1.28)	2	0.73 (0.36, 1.40)	2	0.38 (0.02, 7.35)	1	0.91 (0.17.4.92)	3	0.94 (0.19.4.96)	3
**EPN**	Reference	4	Reference	4	Reference	4	Reference	4	Reference	4
**TPN**	1.49 (0.97, 2.36)	5	1.16 (0.72, 1.96)	5	3.02 (0.08, 1.11)	5	0.91 (0.42, 1.93)	2	0.95 (0.44, 1.99)	2
**Number of studies**	42		34		8		12		11	
**partcipants**	6217		5788		429		3559		3484	

Abbreviations: CrI: credible interval; EPN: Enteral nutrition or adjuvant peripheral parenteral nutrition; OR: odds ratio; TPN: Total parenteral nutrition.

Appendix 14
Sensitivity analyses

Table S 14.1 Sensitivity analyses for the risk of nosocomial infection

The following table shows the odds ratio and the rank order (SUCRA ranks) between overall patients and subgroup analyses.

Treatment	Overall patients		High quality studies only (low risk of bias)	
	OR (95% CrI)	Rank	OR (95% CrI)	Rank
Synbiotics	**0.37 (0.22, 0.61)**	**1**	**0.32 (0.13, 0.71)**	**1**
Probiotics	**0.52 (0.34, 0.77)**	**2**	0.42 (0.16, 1.10)	2
Prebiotics	0.65 (0.35, 1.15)	3	0.71 (0.28, 1.60)	3
EPN	reference	4	Reference	4
TPN	**2.29 (1.48, 3.67)**	**5**	**2.20 (1.10, 4.60)**	**5**
Number of studies	43		26	
partcipants	6215		4663	

Appendix 15

References of included studies

1. Braga M, Vignali A, Gianotti L, Cestari A, Profili M, Di Carlo V: Benefits of early postoperative enteral feeding in cancer patients. Infusionstherapie und Transfusionsmedizin 1995, 22(5):280-284.

2. Kudsk KA, Minard G, Croce MA, Brown RO, Lowrey TS, Pritchard FE, Dickerson RN, Fabian TC: A randomized trial of isonitrogenous enteral diets after severe trauma - An immune-enhancing diet reduces septic complications. Annals of surgery 1996, 224(4):531-540.

3. Bleichner G, Bléhaut H, Mentec H. Saccharomyces boulardii prevents diarrhea in critically ill tube-fed patients A multicenter, randomized, double-blind placebo-controlled trial .Intensive care medicine. 1997;23(5):517-523.

4. Falcão de Arruda IS. Benefits of early enteral nutrition with glutamine and probiotics in brain injury patients. Clinical science (London, England: 1979). 2004;106(3):287-292.

5. Jain PK, McNaught CE, Anderson AD, et al. Influence of synbiotic containing Lactobacillus acidophilus La5, Bifidobacterium lactis Bb 12, Streptococcus thermophilus, Lactobacillus bulgaricus and oligofructose on gut barrier function and sepsis in critically ill patients: a randomised controlled trial. Clinical nutrition (Edinburgh, Scotland). 2004;23(4):467-475.

6. Lu X, Han CM, Yu JX. Preliminary comparative study on the effects of early enteral supplementation of synbiotics on severely burned patients. Zhonghua shao shang za zhi. 2004;20(4):198-201.

7. Sun B, Gao Y, Xu J, Zhou XL, Zhou ZQ, Liu C, Jiang HC: Role of individually staged nutritional support in the management of severe acute pancreatitis. Hepatobiliary and Pancreatic Diseases International 2004, 3(3):458-463.

8. McNaught CE, Woodcock NP, Anderson AD. A prospective randomised trial of probiotics in critically ill patients. Clinical nutrition (Edinburgh, Scotland). 2005;24(2):211-219.

9. Klarin B, Johansson ML, Molin G, et al. Adhesion of the probiotic bacterium Lactobacillus plantarum 299v onto the gut mucosa in critically ill patients: a randomised open trial. Critical care (London, England). 2005;9(3):R285-93.

10. Lee E Morrow, Marin H Kollef, James B Bowers, et al. Probiotic manipulation of the native flora in critically ill patients: an opportunity for ventilatorassociated pneumonia prophylaxis?. Chest. 2005;128(4_MeetingAbstracts): 144S.

11. Kotzampassi K, Giamarellos-Bourboulis EJ, Voudouris A, et al. Benefits of a synbiotic formula (Synbiotic 2000Forte) in critically Ill trauma patients: early results of a randomized controlled trial. World journal of surgery. 2006;30(10):1848-1855.

12. Petrov MS, Kukosh MV, Emelyanov NV: A randomized controlled trial of enteral versus parenteral feeding in patients with predicted severe acute pancreatitis shows a significant reduction in mortality and in infected pancreatic complications with total enteral nutrition. Digestive Surgery 2006, 23(5-6):336-345.

13. Alberda C, Gramlich L, Meddings J, et al. Effects of probiotic therapy in critically ill patients: a randomized, double-blind, placebo-controlled trial. The American journal of clinical nutrition. 2007;85(3):816-823.

14. Abdulmeguid AM, Hassan A: Enteral versus parenteral nutrition in mechanically ventilated patients. Neurologia Croatica 2007, 56:15-24.

15. Karakan T, Ergun M, Dogan I, et al. Comparison of early enteral nutrition in severe acute pancreatitis with prebiotic fiber supplementation versus standard enteral solution: a prospective randomized double-blind study.World journal of gastroenterology. 2007;13(19):2733-2737.

16. Casas M, Mora J, Fort E, Aracil C, Busquets D, Galter S, Jáuregui CE, Ayala E, Cardona D, Gich I et al: Total enteral nutrition vs. total parenteral nutrition in patients with severe acute pancreatitis. Revista Espanola de Enfermedades Digestivas 2007, 99(5):264-269.

17. Oláh A, Belágyi T, Pótó L, et al. Synbiotic control of inflammation and infection in severe acute pancreatitis: a prospective, randomized, double blind study. Hepato-gastroenterology. 2007;54(74):590-594.

18. Spindler-Vesel A, Bengmark S, Vovk I, et al. Synbiotics, prebiotics, glutamine, or peptide in early enteral nutrition: a randomized study in trauma patients. JPEN J Parenter Enteral Nutr . 2007;31(2):119-126.

19. Sramek V, Dadak L, Stouracova M, et al. Impact of addition of synbiotics (Synbiotic 2000 Forte) to enteral nutrition on the course of MODS, occurrence of sepsis, immune status and gut function in long-term critically ill patients. Anesteziologie a intenzivni medicina. 2007;18(3):157-163

20. Besselink MG, van Santvoort HC, Buskens E, et al. Probiotic prophylaxis in predicted severe acute pancreatitis: a randomised, double-blind, placebo-controlled trial. Lancet (London, England). 2008;371(9613):651-659.

21. Forestier C, Guelon D, Cluytens V, et al. Oral probiotic and prevention of Pseudomonas aeruginosa infections: a randomized, double-blind, placebo-controlled pilot study in intensive care unit patients. Critical care (London, England). 2008;12(3):R69.

22. Klarin B, Wullt M, Palmquist I, et al. Lactobacillus plantarum 299v reduces colonisation of Clostridium difficile in critically ill patients treated with antibiotics. Acta anaesthesiologica Scandinavica. 2008;52(8):1096-1102.

23. Doley RP, Yadav TD, Wig JD, Kochhar R, Singh G, Bharathy KGS, Kudari A, Gupta R, Gupta V, Poornachandra KS et al: Enteral nutrition in severe acute pancreatitis. Journal of the Pancreas 2009, 10(2):157-162.

24. Giamarellos-Bourboulis EJ, Bengmark S, Kanellakopoulou K. Pro- and synbiotics to control inflammation and infection in patients with multiple injuries.The Journal of trauma. 2009;67(4):815-821.

25. Knight DJ, Gardiner D, Banks A, et al. Effect of synbiotic therapy on the incidence of ventilator associated pneumonia in critically ill patients: a randomised, double-blind, placebo-controlled trial. Intensive care medicine. 2009;35(5):854-861.

26. Moses V, Mahendri NV, John G, Peter JV, Ganesh A: Early hypocaloric enteral nutritional supplementation in acute organophosphate poisoning - A prospective randomized trial. Clinical Toxicology 2009, 47(5):419-424.

27. Barraud D, Blard C, Hein F, et al. Probiotics in the critically ill patient: a double blind, randomized, placebo-controlled trial. Intensive care medicine. 2010;36(9):1540-1547.

28. Frohmader TJ, Chaboyer WP, Robertson IK. Decrease in frequency of liquid stool in enterally fed critically ill patients given the multispecies probiotic VSL#3: a pilot trial. American journal of critical care: an official publication, American Association of Critical-Care Nurses. 2010;19(3):e1-11.

29. Morrow LE, Kollef MH. Probiotic prophylaxis of ventilator-associated pneumonia: a blinded, randomized, controlled trial. American journal of respiratory and critical care medicine. 2010;182(8):1058-1064.

30. Ferrie S. Lactobacillus GG as treatment for diarrhea during enteral feeding in critical illness: randomized controlled trial. JPEN J Parenter Enteral Nutr. 2011;35(1):43-49.

31. Tan M, Zhu JC, Du J, et al. Effects of probiotics on serum levels of Th1/Th2 cytokine and clinical outcomes in severe traumatic brain-injured patients: a prospective randomized pilot study. Critical care (London, England) . 2011; 15(6):R290.

32. Hayakawa M, Asahara T, Ishitani T, et al. Synbiotic therapy reduces the pathological gram-negative rods caused by an increased acetic acid concentration in the gut. Digestive diseases and sciences. 2012;57(10):2642-2649.

33. Malian M, Reichenbach R, PeckA, et al. Probiotic supplementation in critical care. Critical Care Medicine. 2012; 40:12 SUPPL 1:275.

34. Plaudis H, Pupelis G, Zeiza K, Boka V: Early Low Volume Oral Synbiotic/Prebiotic Supplemented Enteral Stimulation of the Gut in Patients with Severe Acute Pancreatitis: A Prospective Feasibility Study. Acta Chirurgica Belgica 2012, 112(2):131-138.

35. Cui LH, Wang XH, Peng LH, et al. The effects of early enteral nutrition with addition of probiotics on the prognosis of patients suffering from severe acute pancreatitis. Zhonghua wei zhong bing ji jiu yi xue. 2013; 25(4):224-228.

36. Elke G, Kuhnt E, Ragaller M, Schadler D, Frerichs I, Brunkhorst FM, Loffler M, Reinhart K, Weiler N, German Competence Network Sepsis S: Enteral nutrition is associated with improved outcome in patients with severe sepsis A secondary analysis of the VISEP trial. Medizinische Klinik-Intensivmedizin Und Notfallmedizin 2013, 108(3):223-233.

37. López de Toro Martín-Consuegra I, Sanchez-Casado M, Pérez-Pedrero Sánchez-Belmonte MJ, et al. The influence of symbiotics in multi-organ failure: randomised trial. Medicina clínica. 2014; 143(4):143-149.

38. Tan M, Lu XL, Duan JW, et al. Effects of probiotics on blood glucose levels and clinical outcomes in patients with severe craniocerebral trauma. Zhonghua wei zhong bing ji jiu yi xue. 2013;25(10):627-630.

39. Wang G, Wen J, Xu L, et al. Effect of enteral nutrition and ecoimmunonutrition on bacterial translocation and cytokine production in patients with severe acute pancreatitis. The Journal of surgical research. 2013;183(2):592-597.

40. Sanaie S, Ebrahimi-Mameghani M, Hamishehkar H, et al. Effect of a multispecies probiotic on inflammatory markers in critically ill patients: A randomized, double-blind, placebo-controlled trial. Journal of research in medical sciences: the official journal of Isfahan University of Medical Sciences. 2014;19(9):827-833.

41. Zhu YM, Lin S, Dang xw, Wang M, et al. Efeects of Probioties intreatment of severe acute Panereatitis. Shi jie hua ren xiao hua za zhi. 2014;

22(32):5013-5017.

42. Fu YH, Wen JB, Wang GL, Wen P, Gong M, Han M, Li X: Effect of enteral nutrition on cytokine production and plasma endotoxin in patients with severe acute pancreatitis. World Chinese Journal of Digestology 2015, 23(7):1174-1179.

43. Kim JM, Joh JW, Kim HJ, Kim SH, Rha M, Sinn DH, Choi GS, Kwon CHD, Cho YY, Suh JM et al: Early Enteral Feeding after Living Donor Liver Transplantation Prevents Infectious Complications. Medicine (United States) 2015, 94(44):e1771.

44. Yong Rongrungruang, Donnaya Krajangwittaya, Kittisak Pholtawornkulchai, et al. Randomized controlled study of probiotics containing Lactobacillus casei (Shirota strain) for prevention of ventilator-associated pneumonia. J Med Assoc Thai. 2015; 98(3):253-259.

45. Fan MC, Wang QL, Fang W, Jiang YX, Li LD, Sun P, Wang ZH: Early Enteral Combined with Parenteral Nutrition Treatment for Severe Traumatic Brain Injury: Effects on Immune Function, Nutritional Status and Outcomes. Chinese Medical Sciences Journal 2016, 31(4):213-220.

46. Malik AA, Rajandram R, Tah PC et al. Microbial cell preparation in enteral feeding in critically ill patients: A randomized, double-blind, placebo-controlled clinical trial. Journal of critical care. 2016;32:182-188.

47. Zarinfar N, harafkhah M, Amiri M, et al. Probiotic effects in prevention from ventilator-associated pneumonia. Koomesh. 2016;17 :4 (803-813).

48. Zeng J, Wang CT, Zhang FS et al. Effect of probiotics on the incidence of ventilator-associated pneumonia in critically ill patients: a randomized controlled multicenter trial. Intensive care medicine. 2016;42(6):1018-1028.

49. Alberda C, Marcushamer S, Hewer T, et al. Feasibility of a Drink in the Intensive Care Unit for Prevention of Antibiotic Associated Diarrhea and Clostridium difficile. Nutrients. 2018; 10(5).

50. Fazilaty Z, Chenari H, Shariatpanahi ZV: Effect of beta-glucan on serum levels of IL-12, hs-CRP, and clinical outcomes in multiple-trauma patients: a prospective randomized study. Ulusal Travma Ve Acil Cerrahi Dergisi-Turkish Journal of Trauma & Emergency Surgery 2018, 24(4):287-293.

51. Manzanares W, Lemieux M, Langlois PL. Probiotic and synbiotic therapy in critical illness: a systematic review and 75meta-analyses. Critical care (London, England) . 2015; 19:262.

52. Reignier J, Boisrame-Helms J, Brisard L, Lascarrou JB, Hssain AA, Anguel N, Argaud L, Asehnoune K, Asfar P, Bellec F et al: Enteral versus parenteral early nutrition in ventilated adults with shock: a randomised, controlled, multicentre, open-label, parallel-group study (NUTRIREA-2). Lancet (London, England) 2018, 391(10116):133-143.

53. Shimizu K, Yamada T, Ogura H, et al. Synbiotics modulate gut microbiota and reduce enteritis and ventilator-associated pneumonia in patients with sepsis: a randomized controlled trial. Critical care (London, England). 2018;22(1):239.

54. Tuncay P, Arpaci F, Doganay M, et al. Use of standard enteral formula versus enteric formula with prebiotic content in nutrition therapy: A randomized controlled study among neuro-critical care patients. Clinical nutrition ESPEN. 2018;25:26-36.

55. Mahmoodpoor A, Hamishehkar H, Asghari R, et al. Effect of a Probiotic Preparation on Ventilator-Associated Pneumonia in Critically Ill Patients Admitted to the Intensive Care Unit: A Prospective Double-Blind Randomized Controlled Trial. Nutrition in clinical practice: official publication of the American Society for Parenteral and Enteral Nutrition. 2019;34(1):156-162.
